# Risk prediction in people with acute myocardial infarction in England: a cohort study using data from 1521 general practices

**DOI:** 10.1136/bmjopen-2024-094961

**Published:** 2025-12-05

**Authors:** Evangelos Kontopantelis, Salwa S Zghebi, Corneliu T Arsene, Azfar G Zaman, Nicholas W S Chew, Harindra C Wijeysundera, Kamlesh Khunti, Darren M Ashcroft, Matthew Carr, Rosa Parisi, Mamas A Mamas

**Affiliations:** 1 Division of Informatics, Imaging and Data Sciences, School of Health Sciences, Faculty of Biology, Medicine and Health, The University of Manchester, Manchester, UK; 2 Division of Population Health, Health Services Research and Primary Care, School of Health Sciences, Faculty of Biology, Medicine and Health, The University of Manchester, Manchester, UK; 3 Freeman Hospital and Newcastle University, Newcastle upon Tyne, UK; 4 School of Vascular Biology and Medicine, Newcastle University, Newcastle Upon Tyne, UK; 5 Department of Cardiology, National University Heart Centre, National University Health System, Singapore; 6 Department of Medicine, Faculty of Medicine, University of Toronto, Toronto, Ontario, Canada; 7 Diabetes Research Centre, University of Leicester, Leicester, UK; 8 Centre for Pharmacoepidemiology and Drug Safety, Division of Pharmacy and Optometry, School of Health Sciences, Faculty of Biology, Medicine and Health, The University of Manchester, Manchester, UK; 9 National Institute for Health and Care Research (NIHR) Greater Manchester Patient Safety Research Collaboration (PSRC), The University of Manchester, Manchester, UK; 10 Keele Cardiovascular Research Group, Centre for Prognosis Research, Institute for Primary Care and Health Sciences, Keele University, Keele, UK

**Keywords:** Patients, Primary Care, CARDIOLOGY, Cardiovascular Disease, PUBLIC HEALTH, Myocardial infarction

## Abstract

**Abstract:**

**Objective:**

To develop prediction models for short-term outcomes following a first acute myocardial infarction (AMI) event (index) or for past AMI events (prevalent) in a national primary care cohort.

**Design:**

Retrospective cohort study using logistic regression models to estimate 1-year and 5-year risks of all-cause mortality and composite cardiovascular outcomes.

**Setting:**

Primary care practices in England contributing data to the Clinical Practice Research Datalink (CPRD) Aurum and CPRD GOLD databases between 2006 and 2019.

**Participants:**

Patients with an incident (index) or prevalent AMI event. Models were trained on a random 80% sample of CPRD Aurum (n=1018 practices), internally validated on the remaining 20% (n=255) and externally validated using CPRD GOLD (n=248).

**Outcome measures:**

Discrimination assessed using sensitivity, specificity and area under the receiver operating characteristic curve (AUC). Calibration assessed using calibration plots.

**Results:**

In the index (prevalent) cohorts, 94 241 (64 789) patients were included in the training and internal validation sets, and 16 832 (7479) in the external validation set. For the index cohort, AUCs for 1-year [5-year] all-cause mortality were 0.802 (95% CI 0.793 to 0.812) [0.847 (0.841 to 0.853)] internally and 0.800 (0.790 to 0.810) [0.841 (0.835 to 0.847)] externally. For the primary composite outcome (stroke, heart failure and all-cause death), AUCs were 0.763 (0.756 to 0.771) [0.824 (0.818 to 0.830)] internally and 0.748 (0.739 to 0.756) [0.808 (0.801 to 0.815)] externally. Discrimination was higher in the prevalent cohort, particularly for 1-year mortality (AUC: 0.896, 95% CI 0.887 to 0.904). Models excluding treatment variables showed slightly lower but comparable performance. Calibration was acceptable across models.

**Conclusions:**

These models can support clinicians in identifying patients at increased risk of short-term adverse outcomes following AMI, whether newly diagnosed or with a prior history. This can inform monitoring strategies and secondary prevention and guide patient counselling on modifiable risk factors.

**STRENGTHS AND LIMITATIONS OF THIS STUDY**

- This study used large, nationally representative primary care datasets (Clinical Practice Research Datalink (CPRD) Aurum and GOLD) with linkage to hospital and mortality records.
- Risk prediction models were developed using logistic regression and validated both internally and externally.
- Multiple imputation was applied to handle missing data, improving model reliability.
- The use of routinely collected clinical data may introduce coding inaccuracies and bias/poor performance due to omitted predictors.
- Time-to-event models were not used, which may limit performance in risk estimation.

## Introduction

 Cardiovascular disease (CVD) accounts for nearly 25% of all UK deaths^1^,^5^ and is the leading cause of death globally,^6^,^13^ representing 32% of all global deaths.^14^ People with existing CVD are estimated to be up to five times more likely to have a stroke and between 10 and 50% of individuals with a first acute myocardial infarction (AMI) develop a second AMI during their lifetime, compared with those without CVD.^15 16^ While several cardiovascular (CV) risk prediction tools exist for incident (new onset) CVD, like the QRISK,^17 18^ Framingham score,^19^ ASSIGN,^20^ SCORE^21^ and NORRISK,^22^ there is a paucity of predictive tools for people with new and existing CVD.^23^

Specific risk stratification tools for AMI survivors are limited, despite an estimated 1.4 million survivors after a first AMI in the UK in 2023.^24^ Following a first AMI event, 1-year mortality risk is 5.6% for males and 7.2% for females, while 7-year risk is 13.9% for males and 16.2% for females.^25^ Small differences have been reported in the risk factors for first and second AMIs, with females being more at risk for second AMI.^15^ The risk of a second AMI is the highest during the first year, in those who survive a first AMI, and the risk of all-cause death in survivors remains high for at least 7 years of follow-up compared with the general population without a history of AMI.^25^ Although these studies identified important risk factors and quantified risks for people who have had an AMI, they stopped short of delivering risk stratification tools that could be used in the primary care setting, where CVD is managed in most countries. Furthermore, only one of the previous studies used primary care data^26^ and examined important coronary risk factors such as smoking status, blood pressure and cholesterol levels.

We provide contemporary mortality and baseline characteristics for a large cohort of AMI patients in UK primary care, identified between 2006 and 2014 with follow-up data to 2019. Our study had three objectives: first, our aim was to develop 1-year and 5-year risk prediction models for all-cause mortality, and a composite of all-cause mortality, heart failure and stroke. Second, we report on factors associated with poor outcomes for this population and separate 1-year models for heart failure, stroke, recurrent AMI and CV death. Third, we developed models to be applied when a person first becomes at risk (first AMI event during the study period, or index date), and also prevalent models, which included all AMI cases active on an audit date (31 December 2014), to account for past and multiple events.

## Methods

### Data sources

The Clinical Practice Research Datalink (CPRD) provides anonymised longitudinal patient-level data on clinical diagnoses, prescribed treatments, patient demographics including comorbidities, ethnicity and deprivation, referrals and linkage to external datasets and disease registries. We analysed English primary care data from CPRD GOLD and CPRD Aurum, collecting data from general practices using the Vision and EMIS Web clinical computer systems, respectively.^27 28^ We used the July 2020 versions of both data resources, focusing on English practices eligible for data linkages, including 1273 general practices for CPRD Aurum and 248 for CPRD GOLD. The lower number for GOLD reflects the transition of many practices from GOLD to Aurum (due to a clinical computer system change), and these were included in Aurum alone so we could maximise their data contribution through a longer follow-up.

Both CPRD GOLD and Aurum databases were linked to data from the Hospital Episode Statistics (HES) Admitted Patient Care (APC), Office for National Statistics (ONS) death registration, ONS Index of Multiple Deprivation (IMD) and ONS 2011 rural-urban classification. HES APC and ONS death registration data are linked using the patient NHS identifier, primarily. The IMD incorporates routine administrative data covering a wider range of indicators, aggregated in seven domains (income, employment, education and skills, health and disability, crime, barriers to housing and services and living environment), using an area-based model at a low geography (Lower Super Output Areas (LSOAs), average of 1500 people).^29^ We obtained the latest available IMD information for the general practice location (2020) and patient residence location (2015) in quintiles; it is known that the measure has changed very little over time.^30^ The rural-urban classification is a simplified dichotomy of a much more detailed characterisation, at the LSOA level, for the general practice location.^31^ The quality of AMI recording in CPRD Aurum has been well validated.^32^

### Study design and study population

Retrospective cohort study, which included patients registered with a CPRD GOLD or Aurum English general practice that was eligible for data linkages, for at least 365 days before the index date, between 1 January 2006 and 31 December 2014, with a diagnosis of an AMI event in their clinical record during the study period (index date), and they were at least 35 years old at the time of that event. Patients could have had AMI events prior to the start of the study period. Eligible patients were followed-up until 31 December 2019 and censored at the earliest of outcome date, death date, transfer out of practice date, practice last collection date or end of study (31 December 2019). Importantly, patients whose follow-up ended (eg, were transferred out or had last collection date) before observing the outcome of interest had their respective outcome imputed (see the following section). The study is reported following the TRIPOD guidelines.^33^

### Outcomes and predictors

We focused on three outcomes, over 1 and 5 years, all-cause death and two composite outcomes of stroke, heart failure and all-cause death (‘composite outcome’), and of stroke, heart failure and CV-related death (‘composite CV outcome’). An alternative composite outcome also included recurrent MI, which was identified from HES data only to minimise misidentifying duplicate recording of MI events (related to index MI) on primary care data as an outcome. Baseline covariates were selected a priori as potentially relevant by the clinical expert in our team (MAM) and included: age, sex, patient-level IMD, ethnicity, body mass index (BMI), smoking, alcohol status and various comorbidities (stroke, diabetes type-1/type-2/other, chronic kidney disease (CKD), heart valve disease, heart failure, hypertension, hypothyroidism, atrial fibrillation, chronic pulmonary disease, cardiomyopathy, coronary heart disease, hyperlipidaemia, ventricular tachycardia/ventricular fibrillation, rheumatoid arthritis, peripheral vascular disease, percutaneous coronary intervention or coronary artery bypass grafting, menopause, systemic lupus erythematosus, erectile dysfunction, dementia, any tumour, HIV/AIDS and liver disease. A second set of covariates was considered (see the Statistical modelling section) that included the above predictors with addition of the following therapies: antihypertensives (including α- and β-adrenoceptor blockers, calcium channel blockers and all RAS acting agents), diuretics, lipid-regulating therapies (statins, fibrates, nicotinic acid), anti-platelets, anticoagulants and glucose lowering therapies (oral antidiabetics, eg, SGLT2is, injectables, eg, GLP-1 agonists and insulin). Additionally, for the prevalent models, we included: the number of AMI before the audit date (ie, in addition to the one observed during the study period). For covariates that change over time (eg, BMI), the information closest to index and audit dates was extracted, if within a year, otherwise set to missing.

Read and SNOMED code lists were used to identify condition diagnoses. The Read code lists were obtained both from the literature and then updated with the *pcdsearch* Stata command^34 35^ and reviewed by researchers and consultant cardiologists in our team. Therapies were identified from relevant product codes in prescriptions. The outcomes from linked HES APC and ONS data were identified using the 10th revision of ICD-10 codes. If two or more codes of the same diagnosis type were recorded in the same day, we assumed they pertained to the same event. For heart failure and stroke, we also included events identified from HES data. For recurrent AMI, there exists a risk of delayed recording and perhaps a primary care entry may relate to a previous event recorded in secondary care^36 37^; however, to overcome this, we only used AMI events from HES data.

### Training and validation cohorts

A random selection of 80% of general practices in CPRD Aurum database (n=1018 general practices) was selected for the training of all models. Internal validation was conducted in the remaining 20% of CPRD Aurum practices (n=255). External validation was conducted in the CPRD GOLD database (n=248 general practices). Model development was not limited to complete cases, but instead we used multiple imputation with chained equations (*mi* suite of commands in Stata), before developing the model on the training cohort.^38 39^ All covariates and all outcomes were included in the imputation model.^40^ For simplicity, only one imputed dataset per outcome per timepoint was created. Variables with imputed data included outcomes, ethnicity, IMD, BMI, smoking status and alcohol consumption. The same imputation model was then applied to the internal and external validation cohorts, to obtain a complete imputed dataset for each cohort.^41^

### Statistical modelling

Both the index models, which focused on new AMI cases over the study period, and the prevalent models, which included all AMI cases active on 31 December 2014, were developed using logistic regression. The covariates previously described were included in all models, with the addition of previous AMI and time since latest AMI for the prevalent models. Both discrimination and calibration of all models, across the three databases, were evaluated. Discrimination was evaluated through the area under the receiver operating characteristic (AUC). Also, for a probability threshold of 0.5 (equal to or higher indicating a positive classification), we report on accuracy, sensitivity, specificity, positive predictive value, negative predictive value, true positives, true negatives, false positives and false negatives (onlinesupplemental files 1  2). Calibration was evaluated through calibration plots and the calibration slope. We obtained CIs for the AUCs and the calibration slopes through bootstraps of 1000 replicates. All model creation steps used are explicitly stated in this section; no additional procedures were applied.

Although therapies are often markers of poorer outcomes, we did not use them in the above models to avoid a potential ‘feedback loop’, that is, patients considered to be at higher risk, prescribed medication, leading to further increased risk. However, in a second set of models, we explored the inclusion of the therapies covariates previously described, in addition to the covariates included in the previously described models. All analyses were conducted in Stata V.17 and an alpha level of 0.05 was used throughout.

### Patient and public involvement

Throughout the course of the study, we engaged with PRIMER (Primary Care Research in Manchester Engagement Resource), a diverse group of patients, carers and members of the public with an interest in primary care research, based at the Centre for Primary Care and Health Services Research at The University of Manchester. Our preliminary findings were presented to PRIMER members, and feedback was requested on the readability of outputs, the predictors used in the modelling and the dissemination of results.

## Results

Cohort sizes varied across outcomes, due to the slightly different inclusion and exclusion criteria. Here, we report on the details of the training and external validation cohorts for the index models and 1-year all-cause mortality, with information on all cohorts presented in table 1 and in online supplemental table S1. In 75 930 people in the Aurum training cohort, mean age was 69.5 years (SD=13.7) and 69.9 years (SD=13.8) in the GOLD external validation cohort, with similar sex distributions in the two databases (37.67% and 37.40% females, respectively). Most participants were of White ethnicity, current consumers of alcohol and current or ex-smokers. Population characteristics in the prior history of AMI cohorts were similar.

**Table 1:** Outcomes and cohort characteristics, index models*

	CPRD aurum		CPRD GOLD (external validation)
	Training dataset (80%)	Validation dataset (20%) (internal validation)	
Number of patients, N (%)	75 930 (80.57)	18 311 (19.43)	16 832 (100)
Number of general practices	1018 (80)	255 (20)	248 (100)
1-year all-cause death	8171 (10.76)	1886 (10.30)	1845 (10.96)
5-year all-cause death	23 259 (30.63)	5536 (30.23)	5937 (35.27)
1-year composite outcome†	20 137 (26.52)	4774 (26.07)	4579 (27.20)
5-year composite outcome†	37 049 (48.79)	8971 (48.99)	9263 (55.03)
1-year composite CV outcome‡	17 989 (23.69)	4301 (23.49)	4108 (24.41)
5-year composite CV outcome‡	33 953 (44.72)	8265 (45.14)	8590 (51.03)
Age, mean (SD)	69.5 (13.7)	69.4 (13.8)	69.9 (13.8)
Gender, N (%)			
Female	28 603 (37.67)	6811 (37.20)	6295 (37.40)
IMD, N (%)			
Q1 (least deprived)	15 267 (20.11)	3729 (20.36)	3051 (18.13)
Q2	15 430 (20.32)	3969 (21.68)	3408 (20.25)
Q3	15 208 (20.03)	3587 (19.59)	3774 (22.42)
Q4	15 060 (19.83)	3353 (18.31)	3602 (21.40)
Q5 (most deprived)	14 965 (19.71)	3673 (20.06)	2997 (17.81)
Ethnicity, N (%)			
White	70 708 (93.12)	16 853 (92.04)	16 062 (95.43)
Non-White	5222 (6.88)	1458 (7.96)	770 (4.57)
Alcohol consumption status, N (%)			
Non/former drinker	13 286 (17.50)	3410 (18.62)	4506 (26.77)
Current drinker	62 644 (82.50)	14 901 (81.38)	12 326 (73.23)
Smoking status, N (%)			
Current smoker	24 476 (32.23)	5907 (32.26)	4868 (28.92)
Ex-smoker	37 295 (49.12)	8962 (48.94)	7520 (44.68)
Never smoker	14 159 (18.65)	3442 (18.80)	4444 (26.40)
BMI (kg/m^2^), mean (SD)	28 (5.5)	28 (5.5)	28 (5.3)
Comorbidities, N (%)			
Hypertension	31 381 (41.33)	7304 (39.89)	8403 (49.92)
Hyperlipidaemia	14 892 (19.61)	3394 (18.54)	3171 (18.84)
Heart failure	4706 (6.20)	1119 (6.11)	1162 (6.90)
Atrial fibrillation	6968 (9.18)	1675 (9.15)	1429 (8.49)
Heart valve disease	867 (1.14)	186 (1.02)	233 (1.38)
Chronic pulmonary disease	9993 (13.16)	2349 (12.83)	1556 (9.24)
Cardiomyopathy	372 (0.49)	99 (0.54)	86 (0.51)
CHD	20 542 (27.05)	4884 (26.67)	6164 (36.62)
Ventricular tachycardia/fibrillation	287 (0.38)	75 (0.41)	34 (0.20)
PVD	4854 (6.39)	1170 (6.39)	1075 (6.39)
Stroke+TIA	7718 (10.16)	1836 (10.03)	1523 (9.05)
CV procedures	7492 (9.87)	1830 (9.99)	1493 (8.87)
Chronic kidney disease	14 215 (18.72)	3380 (18.46)	2731 (16.23)
Diabetes			
T1DM	592 (0.78)	134 (0.73)	136 (0.81)
T2DM	12 462 (16.41)	3107 (16.97)	2514 (14.94)
Unspecified	1007 (1.33)	236 (1.29)	213 (1.27)
All	14 061 (18.5)	3477 (19)	2863 (17)
Hypothyroidism	5903 (7.77)	1463 (7.99)	1339 (7.96)
Liver disease	153 (0.20)	41 (0.22)	109 (0.65)
Lupus	244 (0.32)	51 (0.28)	40 (0.24)
Erectile dysfunction	7054 (9.29)	1774 (9.69)	1689 (10.03)
HIV_AIDS	61 (0.08)	14 (0.08)	7 (0.04)
Menopause	6620 (8.72)	1651 (9.02)	1807 (10.74)
Rheumatoid arthritis	2464 (3.25)	583 (3.18)	394 (2.34)
Dementia	1669 (2.20)	415 (2.27)	360 (2.14)
Any tumour	11 608 (15.29)	2886 (15.76)	2353 (13.98)
Medications, N (%)			
Lipid-lowering agents	36 525 (48.10)	8772 (47.91)	7713 (45.82)
Diuretics	34 564 (45.52)	8359 (45.65)	7601 (45.16)
Antihypertensives	52 777 (69.51)	12 796 (69.88)	11 510 (68.38)
Glucose lowering therapies	11 516 (15.17)	2913 (15.91)	2386 (14.18)
Anticoagulants	5157 (6.79)	1263 (6.90)	1403 (8.34)
Antiplatelets	35 002 (46.10)	8425 (46.01)	7653 (45.47)

* Includes imputed values for outcomes, ethnicity, deprivation, BMI, smoking status and alcohol consumption.

† Stroke, heart failure and all-cause death.

‡ Stroke, heart failure and CV-related death.

BMI, body mass index; CHD, coronary heart disease ; CPRD, Clinical Practice Research Datalink; CV, cardiovascular; IMD, Index of Multiple Deprivation; PVD, Peripheral Vascular Disease; T1DM, type 1 diabetes mellitus; T2DM, type 2 diabetes mellitus; TIA, Transient Ischaemic Attack.

In the index cohorts, 8171 (10.76%) patients in the Aurum training cohort and 1845 (10.96%) in the GOLD external validation cohort died within 1 year, while 23 259 (30.63%) and 5937 (35.27%) died within 5 years, respectively. The 1-year and 5-year composite outcomes were higher and ranged from over 23% for the 1-year composite CV outcome to over 55% for the 5-year composite outcome (table 1). In the prior history of AMI cohorts, 5529 (10.74%) patients in the Aurum training cohort and 887 (11.86%) in the GOLD external validation cohort died within 1 year, while 13 306 (25.84%) and 2345 (31.35%) died within 5 years, respectively. The 1-year and 5-year composite outcomes ranged from 12.89% for the 1-year composite CV outcome to over 48% for the 5-year composite CV outcome (online supplemental table S1).

### Index model performance

Index model performance is summarised in table 2 (and online supplemental table S2). Overall, in the internal validation cohorts, performance was good to very good, ranging from an AUC of 0.758 (95% CI 0.750 to 0.766) for the 1-year composite CV outcome to an AUC of 0.847 (95% CI 0.841 to 0.853) for 5-year all-cause mortality, with over 72% of subjects correctly classified in all models. Performance in the external validation cohorts was slightly lower for most outcomes and AUC ranged from 0.741 (95% CI 0.731 to 0.750) for the 1-year composite CV outcome to 0.841 (95% CI 0.835 to 0.847) for 5-year all-cause mortality. Calibration was also affected in the external validation cohorts, especially for the composite outcomes.

**Table 2:** Performance summary, index models including therapies

Outcome	Internal validation (Aurum)			External validation (GOLD)		
	AUC (95% CI)	Correctly classified (%)	Calibration slope	AUC (95% CI)	Correctly classified (%)	Calibration slope
1-year all-cause mortality	0.802 (0.793 to 0.812)	77.34	0.973	0.800 (0.790 to 0.810)	77.48	0.964
1-year composite outcome*	0.763 (0.756 to 0.771)	72.99	0.947	0.748 (0.739 to 0.756)	71.94	0.860
1-year composite CV outcome†	0.758 (0.750 to 0.766)	73.50	0.957	0.741 (0.731 to 0.750)	72.28	0.860
5-year all-cause mortality	0.847 (0.841 to 0.853)	77.96	0.959	0.841 (0.835 to 0.847)	77.01	0.949
5-year composite outcome*	0.824 (0.818 to 0.830)	74.95	0.967	0.808 (0.801 to 0.815)	73.25	0.919
5-year composite CV outcome†	0.818 (0.812 to 0.825)	75.03	0.965	0.794 (0.787 to 0.801)	72.46	0.865

* Stroke, heart failure and all-cause death.

† Stroke, heart failure and CV-related death.

AUC, area under the receiver operating characteristic curve; CV, cardiovascular.

### Prior history of AMI model performance

**Figure 1:**
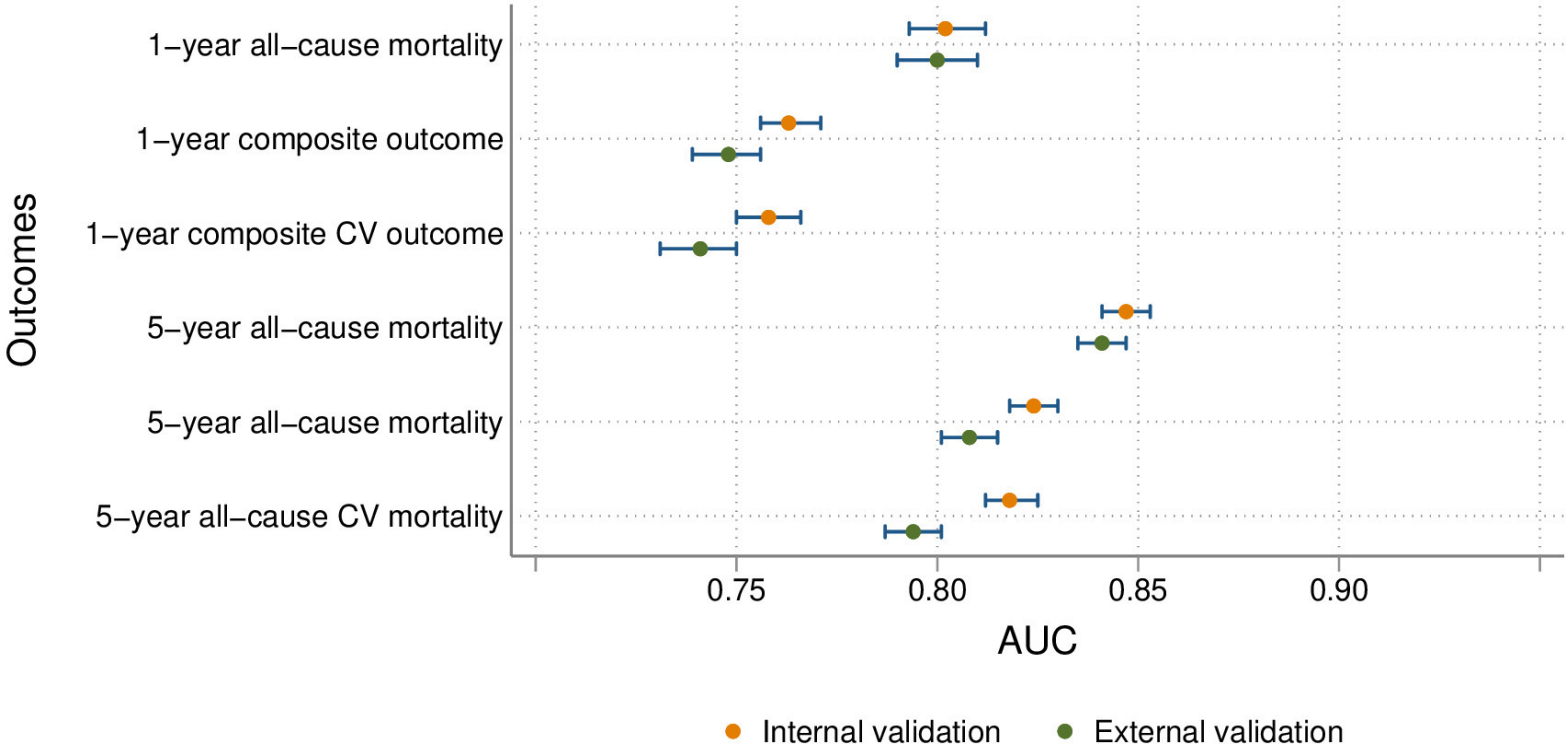
Discrimination performance summary for index models, including therapies. AUC, area under the receiver operating characteristic curve; CV, cardiovascular.

**Figure 2:**
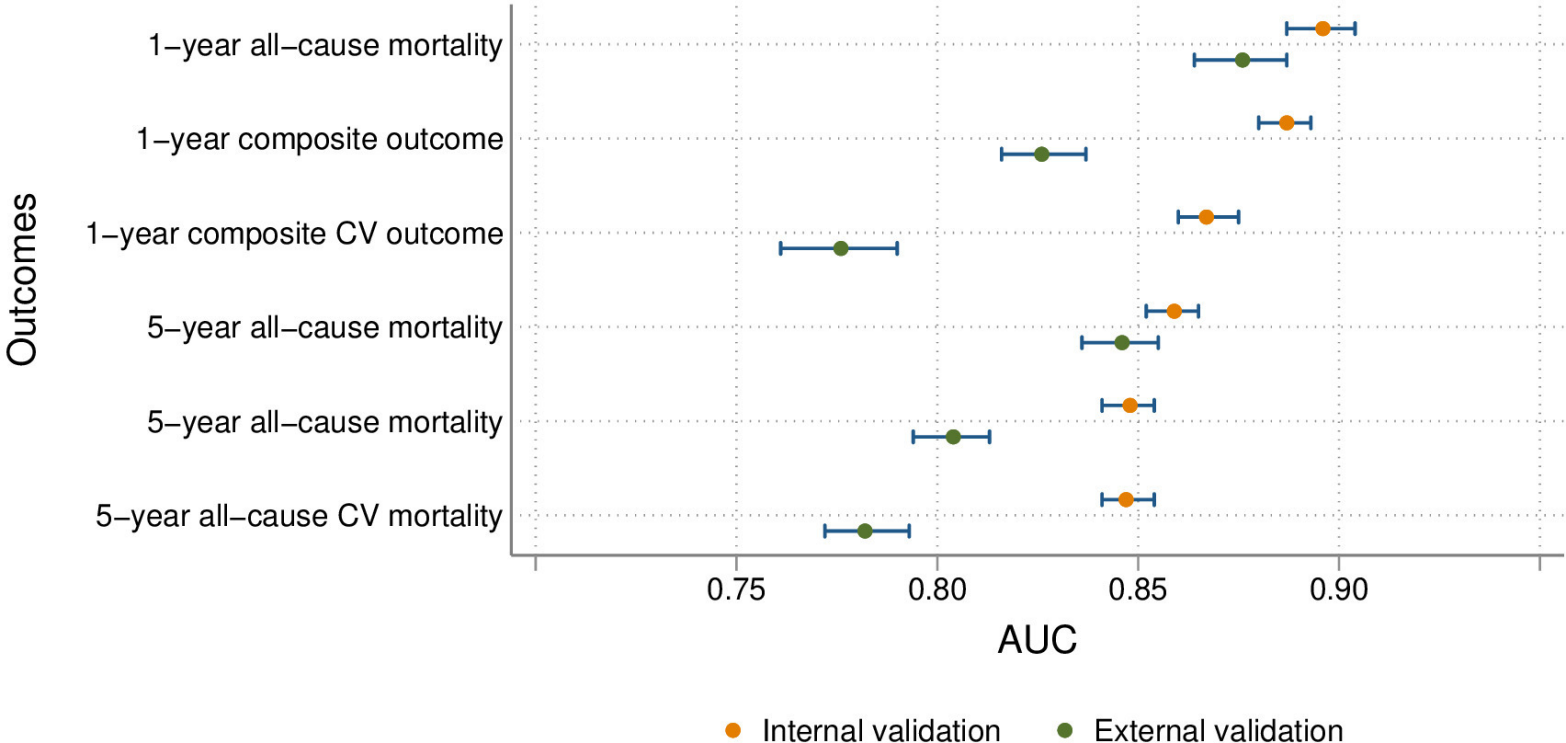
Discrimination performance summary for prevalent models, including therapies. AUC, area under the receiver operating characteristic curve; CV, cardiovascular.

The prior history of AMI model performance is summarised in table 3 (and online supplemental table S3). Performance was higher than in the index AMI models, especially in the internal validation cohorts, ranging from an AUC of 0.847 (95% CI 0.841 to 0.854) for the 5-year composite CV outcome to an AUC of 0.896 (95% CI 0.887 to 0.904) for 1-year all-cause mortality, with correct classification exceeding 82% for the 1-year outcomes. However, performance was slightly lower in the external validation cohorts, especially for the composite CV outcomes, with the AUC ranging from 0.776 (95% CI 0.761 to 0.790) for the 1-year composite CV outcome to 0.876 (95% CI 0.864 to 0.887) for 1-year all-cause mortality. In addition, for the composite CV outcomes, calibration was severely affected. All model AUCs are summarised in figure 1 (index models) and figure 2 (prevalent models).

**Table 3:** Performance summary, prevalent models including therapies

Outcome	Internal validation (Aurum)			External validation (GOLD)		
	AUC (95% CI)	Correctly classified (%)	Calibration slope	AUC (95% CI)	Correctly classified (%)	Calibration slope
1-year all-cause mortality	0.896 (0.887 to 0.904)	86.28	1.050	0.876 (0.864 to 0.887)	85.16	0.949
1-year composite outcome*	0.887 (0.880 to 0.893)	82.83	0.995	0.826 (0.816 to 0.837)	77.39	0.668
1-year composite CV outcome†	0.867 (0.860 to 0.875)	82.44	0.964	0.776 (0.761 to 0.790)	76.67	0.555
5-year all-cause mortality	0.859 (0.852 to 0.865)	79.73	1.045	0.846 (0.836 to 0.855)	78.53	1.010
5-year composite outcome*	0.848 (0.841 to 0.854)	77.16	1.049	0.804 (0.794 to 0.813)	72.87	0.812
5-year composite CV outcome†	0.847 (0.841 to 0.854)	77.77	1.042	0.782 (0.772 to 0.793)	71.95	0.708

* Stroke, heart failure and all-cause death.

† Stroke, heart failure and CV-related death.

AUC, area under the receiver operating characteristic curve; CV, cardiovascular.

### Additional models

The alternative composite score models, which included recurrent MI, performed considerably worse and were not pursued further. Models for predicting recurrent MI were not much better than chance. The models that included therapies performed slightly better than the models not including them; hence, we prioritised the former. All these models, with therapies and without, as well as the associations between covariates of interest and outcomes, are presented in full within online supplemental file 1 (primary outcomes only) and online supplemental file 2 (all outcomes).

## Discussion

The findings of this study demonstrate good or very good discrimination for the developed risk prediction models following incident AMI, but also for prevalent AMI, focusing on 1-year and 5-year mortality and composite CVD outcomes. The results confirmed that up to 18% of all the AMI index cohort experienced a heart failure event, 7% had a stroke and 6% died of CVD by 1 year. Time since index data was very important for the combination of secondary outcomes, with very high risk during the first year following AMI diagnosis.

Multiple contemporary CV risk stratification tools have been developed to be used in clinical practice to predict incident CV events. In contrast, there are limited clinical tools to predict prognosis in patients with established CVD, representing a significant barrier in the management of such patients in clinical practice. Our study is novel in using data from primary care, where the majority of post AMI patients are managed and adds to the existing literature where there is a paucity of predictive tools based on contemporary data of primary care cohorts with a past history of AMI.

### Findings in context

Implementing risk prediction tools is preferable in primary care, where CV risk management routinely takes place. A 2016 study developed a prediction model for in-hospital mortality in patients with AMI and reported an AUC of 0.88 in internal validation.^42^ More recently, Wu *et al* developed a risk prediction model for in-hospital major CV events in patients hospitalised for AMI in China, reporting good discrimination in two external validation samples (AUCs of 0.74 and 0.80).^43^ Other recent studies have focused on readmission and predicting major adverse cardiac events after hospital discharge. Dreyer *et al* reported modest discrimination in internal validation (AUC=0.67), predicting readmission over the first year following hospitalisation for AMI in younger adults (≤55 years).^44^ Korean registry data were used to develop a machine learning (deep learning) model to predict occurrences of major adverse cardiac events during 1-year follow-up after hospital discharge in patients with AMI, with the authors reporting excellent discrimination (AUC=0.96) in the validation sample.^45^

In terms of the identified input covariates with prognostic importance, these were in agreement with those reported in other international primary studies, in the US, Canada and Switzerland, and in what has been reported in systematic reviews. Diabetes and age of AMI diagnosis are known risk factors for stroke^46 47^; atrial fibrillation, age of first AMI diagnosis, COPD (due to cigarette smoking) and CKD are risk factors for heart failure^48 49^; heart failure and CKD are risk factors for CV death^50 51^; hypertension and dyslipidaemia are risk factors for recurrent MI.^52 53^ In our models, we included additional clinically relevant covariates, diabetes and stroke for heart failure, cardiac surgery for recurrent MI, age of AMI diagnosis, diabetes, stroke and cardiac surgery for CV death. In addition, we also included a measure of socioeconomic deprivation, which is an important and often ignored determinant of CVD outcomes and mortality.^54 55^ We also developed two sets of models (index and prevalent), with a slight variation in their included components, with the prevalent models additionally including the number of previous AMI events. In both Aurum and GOLD, we observed high risk for both composite outcomes in the first year following an AMI diagnosis (approximately 1 in 4), confirming the results of previous studies in England^25^ and Sweden,^15^ and reinforcing the importance of short-term clinical care, following AMI.

In spite of nearly 7 million people in the UK with pre-existing CVD, there is a paucity of tools to support clinicians in risk stratifying these patients in primary care. Our proposed CV risk stratification tool, derived from primary care data, can provide actionable information to help healthcare professionals managing patients in primary care to identify patients with existing CVD at higher risk for future adverse events, giving clinicians the option of greater risk factor control and implementation of more optimal secondary care interventions.

In addition, the algorithms will enable primary care practitioners to discuss the impact of CV risk factor modification on future CV events, identify patients that may benefit from more aggressive secondary prevention management strategies such as longer and/or more potent antiplatelet regimes, prescribing additional lipid lowering medication or even be used in clinical trials to identify patients at higher risk of CV events to evaluate novel therapies. We also developed two sets of models, index and prevalent, to provide more tailored decision support for AMI management during the patients’ life course.

Several chronic conditions, including CVD, are increasingly managed in primary care,^56^ and validated risk stratification algorithms are important not only on an individual level but also on a health system level. Despite the evidence on the efficacy of interventions for secondary prevention of CVD, guidelines-based recommendations are sub-optimally implemented in clinical practice.^57^ Through identification of primary care practices with a high proportion of high-risk individuals, healthcare resources can be proportionately allocated to affected primary care practices. This includes use of organisational^58^ and internet-based interventions to improve risk factor management.^59^

### Strengths and limitations

Our study has several strengths. First, by using the Electronic Health Records (EHRs) available in the CPRD medical databases, we obtained a large sample size with excellent national representativeness. Another advantage was the linkage to HES and other sources of information which augmented the validity of the patient records thereby providing a contemporary and longitudinal epidemiological analysis of incident and prevalent AMI in England. Second, we studied all-cause mortality, CV death and several outcomes of interest, before training and validating 1-year and 5-year risk models on two large databases. In addition, the predictors used herein were based on data routinely collected in general practice visits, making the models likely to be generalisable in countries with similar primary care settings.

Our study also has several limitations. By using administrative data, we are dependent on the accurate recording of diagnoses and biological measurements in patients’ EHRs, although recent work has highlighted that the accuracy of an AMI diagnosis in the CPRD is high.^60^ Missing data is a common problem with data of this type, and we addressed this using advanced longitudinal imputation methods. Many more covariates would be relevant to accurate risk prediction (eg, troponin levels, ECG findings) but we aimed to develop a risk prediction model for use in primary care, where such information is not routinely available. Multimorbidity and frailty will be driving 1- and 5-year mortality; however, we opted not to use composite scores. Instead, we included a wide range of individual comorbidities to better capture distinct predictive contributions. Including both aggregate scores and their component conditions would introduce redundancy, and we anticipated that dimensionality reduction could compromise model performance. We did not differentiate between AMI subtypes (eg, Type 1 due to atherosclerotic plaque rupture vs Type 2 due to mismatch between myocardial oxygen supply and demand), as this information is not routinely available in primary care records. However, given that Type 1 is the predominant form captured in secondary care, we expect most cases to reflect this subtype. We acknowledge that competing risks, particularly from non-CV causes, may be more prominent in other AMI subtypes and could influence risk prediction. Finally, we decided not to use time-to-event models, so that we would be able to provide clear prediction metrics for specific time points (1 year and 5 years), while employing simpler and less computationally demanding models. However, while logistic regression offers simplicity and interpretability, it does not account for censoring or varying follow-up times, which may introduce bias in longer-term predictions, leading to poor performance. On the other hand, this approach will allow us to directly compare with machine learning methods in future work, while making it much easier to implement the models in primary care.

## Conclusions

We developed and validated prognostic models of future adverse outcomes using a large cohort of patients with AMI in primary care. This paper provides contemporary information on the clinical profile of patients with existing AMI, and the short- and medium-term risks. This addresses an important gap, since most of the literature focuses on either the risk of a first AMI or utilises secondary care data for risk prediction for a subsequent event. The proposed CV prediction tools, derived from primary care data, can provide actionable information to help primary care physicians identify patients with existing CVD, who are at higher risk for future adverse events. Thus, they can prove useful in guiding more aggressive secondary care interventions and/or risk factor management, enabling primary care physicians to discuss the impact of CV risk-factor modification on future CV events, as well as identify patients that may benefit from more aggressive secondary management. One other benefit of using validated risk stratification tools in primary care is the potential to identify patients at higher risk of CV events for enrolment in clinical trials to evaluate novel therapies.

## Supplementary material

10.1136/bmjopen-2024-094961 online supplemental file 1

10.1136/bmjopen-2024-094961 online supplemental file 2

## Data Availability

Data may be obtained from a third party and are not publicly available.
