# Supplementary material for: Risk prediction in people with acute myocardial infarction in England: a cohort study using data from 1521 general practices
Source: BMJ Open. 2025 Dec 5;15(12):e094961. doi: 10.1136/bmjopen-2024-094961 (PMC12684221; doi:10.1136/bmjopen-2024-094961)
Supplement: online supplemental file 2 [file bmjopen-15-12-s002.docx]

**Supplementary file 2: Results for all outcomes**

**for “Epidemiology and risk prediction in people with Acute Myocardial Infarction in England, UK: cohort study, 2006 to 2019”**

Contents

[Baseline characteristics 7](#_Toc144304698)

[Internal and external validation - Index models 13](#_Toc144304699)

[Internal validation (CPRD Aurum) - without therapies 13](#_Toc144304700)

[1) 1-year all-cause mortality 13](#_Toc144304701)

[2) 1-year Stroke 15](#_Toc144304702)

[3) 1- year heart failure (HF) 16](#_Toc144304703)

[4) 1-year Recurrent MI 18](#_Toc144304704)

[5) 1-year CV mortality 20](#_Toc144304705)

[6) 1-year Composite 1 outcome (HF, stroke, recurrent MI, & all-cause mortality) 22](#_Toc144304706)

[7) 1-year Composite 1 CV outcome (HF, stroke, recurrent MI, & CV mortality) 24](#_Toc144304707)

[8) 1-year Composite 2 outcome (HF, stroke, & all-cause mortality) 26](#_Toc144304708)

[9) 1-year Composite 2 CV outcome (HF, stroke, & CV mortality) 28](#_Toc144304709)

[External validation (CPRD GOLD) - without therapies 31](#_Toc144304710)

[1) 1-year all-cause mortality 31](#_Toc144304711)

[2) 1-year Stroke 32](#_Toc144304712)

[3) 1-year heart failure (HF) 34](#_Toc144304713)

[4) 1-year Recurrent MI 36](#_Toc144304714)

[5) 1-year CV mortality 38](#_Toc144304715)

[6) 1-year Composite 1 outcome (HF, stroke, recurrent MI, & all-cause mortality) 40](#_Toc144304716)

[7) 1-year Composite 1 CV outcome (HF, stroke, recurrent MI, & CV mortality) 42](#_Toc144304717)

[8) 1-year Composite 2 outcome (HF, stroke, & all-cause mortality) 44](#_Toc144304718)

[9) 1-year Composite 2 CV outcome (HF, stroke, & CV mortality) 46](#_Toc144304719)

[Internal validation (CPRD Aurum) - with therapies 49](#_Toc144304720)

[1) 1-year all-cause mortality 49](#_Toc144304721)

[2) 1-year Stroke 51](#_Toc144304722)

[3) 1-year heart failure (HF) 53](#_Toc144304723)

[4) 1-year Recurrent MI 55](#_Toc144304724)

[5) 1-year CV mortality 57](#_Toc144304725)

[6) 1-year Composite 1 outcome (HF, stroke, recurrent MI, & all-cause mortality) 59](#_Toc144304726)

[7) 1-year Composite 1 CV outcome (HF, stroke, recurrent MI, & CV mortality) 61](#_Toc144304727)

[8) 1-year Composite 2 outcome (HF, stroke, & all-cause mortality) 63](#_Toc144304728)

[9) 1-year Composite 2 CV outcome (HF, stroke, & CV mortality) 65](#_Toc144304729)

[External validation (CPRD GOLD) - with therapies 67](#_Toc144304730)

[1) 1-year all-cause mortality 67](#_Toc144304731)

[2) 1-year Stroke 69](#_Toc144304732)

[3) 1-year heart failure (HF) 71](#_Toc144304733)

[4) 1-year Recurrent MI 73](#_Toc144304734)

[5) 1-year CV mortality 75](#_Toc144304735)

[6) 1-year Composite 1 outcome (HF, stroke, recurrent MI, & all-cause mortality) 77](#_Toc144304736)

[7) 1-year Composite 1 CV outcome (HF, stroke, recurrent MI, & CV mortality) 79](#_Toc144304737)

[8) 1-year Composite 2 outcome (HF, stroke, & all-cause mortality) 81](#_Toc144304738)

[9) 1-year Composite 2 CV outcome (HF, stroke, & CV mortality) 83](#_Toc144304739)

[Internal validation (CPRD Aurum) - without therapies 85](#_Toc144304740)

[1) 5-year all-cause mortality 85](#_Toc144304741)

[2) 5-year Stroke 86](#_Toc144304742)

[3) 5-year heart failure (HF) 88](#_Toc144304743)

[4) 5- year Recurrent MI outcome 90](#_Toc144304744)

[5) 5-year CV mortality 92](#_Toc144304745)

[6) 5-year Composite 1 outcome (HF, stroke, recurrent MI, & all-cause mortality) 94](#_Toc144304746)

[7) 5-year Composite 1 CV outcome (HF, stroke, recurrent MI, & CV mortality) 96](#_Toc144304747)

[8) 5-year Composite 2 outcome (HF, stroke, & all-cause mortality) 98](#_Toc144304748)

[9) 5-year Composite 2 CV outcome (HF, stroke, & CV mortality) 100](#_Toc144304749)

[External validation (CPRD GOLD) - without therapies 103](#_Toc144304750)

[1) 5-year all-cause mortality 103](#_Toc144304751)

[2) 5-year Stroke 105](#_Toc144304752)

[3) 5-year heart failure (HF) 106](#_Toc144304753)

[4) 5-year Recurrent MI 108](#_Toc144304754)

[5) 5-year CV mortality 110](#_Toc144304755)

[6) 5-year Composite 1 outcome (HF, stroke, recurrent MI, & all-cause mortality) 112](#_Toc144304756)

[7) 5-year Composite 1 CV outcome (HF, stroke, recurrent MI, & CV mortality) 114](#_Toc144304757)

[8) 5-year Composite 2 outcome (HF, stroke, & all-cause mortality) 116](#_Toc144304758)

[9) 5-year Composite 2 CV outcome (HF, stroke, & CV mortality) 118](#_Toc144304759)

[Internal validation (CPRD Aurum) - with therapies 121](#_Toc144304760)

[1) 5-year all-cause mortality 121](#_Toc144304761)

[2) 5-year Stroke 123](#_Toc144304762)

[3) 5-year heart failure (HF) 125](#_Toc144304763)

[4) 5-year Recurrent MI 127](#_Toc144304764)

[5) 5-year CV mortality 129](#_Toc144304765)

[6) 5-year Composite 1 outcome (HF, stroke, recurrent MI, & all-cause mortality) 131](#_Toc144304766)

[7) 5-year Composite 1 CV outcome (HF, stroke, recurrent MI, & CV mortality) 133](#_Toc144304767)

[8) 5-year Composite 2 outcome (HF, stroke, & all-cause mortality) 135](#_Toc144304768)

[9) 5-year Composite 2 CV outcome (HF, stroke, & CV mortality) 137](#_Toc144304769)

[External validation (CPRD GOLD) - with therapies 140](#_Toc144304770)

[1) 5-year all-cause mortality 140](#_Toc144304771)

[2) 5-year Stroke 142](#_Toc144304772)

[3) 5-year heart failure (HF) 144](#_Toc144304773)

[4) 5-year Recurrent MI 146](#_Toc144304774)

[5) 5-year CV mortality 148](#_Toc144304775)

[6) 5-year Composite 1 outcome (HF, stroke, recurrent MI, & all-cause mortality) 150](#_Toc144304776)

[7) 5-year Composite 1 CV outcome (HF, stroke, recurrent MI, & CV mortality) 152](#_Toc144304777)

[8) 5-year Composite 2 outcome (HF, stroke, & all-cause mortality) 154](#_Toc144304778)

[9) 5-year Composite 2 CV outcome (HF, stroke, & CV mortality) 156](#_Toc144304779)

[Internal and external validation - Audit models 158](#_Toc144304780)

[Internal validation (CPRD Aurum) - without therapies 158](#_Toc144304781)

[1) 1-year all-cause mortality 158](#_Toc144304782)

[2) 1-year Stroke 160](#_Toc144304783)

[3) 1-year heart failure (HF) 162](#_Toc144304784)

[4) 1-year Recurrent MI 164](#_Toc144304785)

[5) 1-year CV mortality 166](#_Toc144304786)

[6) 1-year Composite 1 outcome (HF, stroke, recurrent MI, & all-cause mortality) 168](#_Toc144304787)

[7) 1-year Composite 1 CV outcome (HF, stroke, recurrent MI, & CV mortality) 170](#_Toc144304788)

[8) 1-year Composite 2 outcome (HF, stroke, & all-cause mortality) 172](#_Toc144304789)

[9) 1-year Composite 2 CV outcome (HF, stroke, & CV mortality) 174](#_Toc144304790)

[External validation (CPRD GOLD) - without therapies 176](#_Toc144304791)

[1) 1-year all-cause mortality 176](#_Toc144304792)

[2) 1-year Stroke 178](#_Toc144304793)

[3) 1-year heart failure (HF) 180](#_Toc144304794)

[4) 1-year Recurrent MI 182](#_Toc144304795)

[5) 1-year CV mortality 184](#_Toc144304796)

[6) 1-year Composite 1 outcome (HF, stroke, recurrent MI, & all-cause mortality) 186](#_Toc144304797)

[7) 1-year Composite 1 CV outcome (HF, stroke, recurrent MI, & CV mortality) 188](#_Toc144304798)

[8) 1-year Composite 2 outcome (HF, stroke, & all-cause mortality) 190](#_Toc144304799)

[9) 1-year Composite 2 CV outcome (HF, stroke, & CV mortality) 192](#_Toc144304800)

[Internal validation (CPRD Aurum) - with therapies 194](#_Toc144304801)

[1) 1-year all-cause mortality 194](#_Toc144304802)

[2) 1-year Stroke 195](#_Toc144304803)

[3) 1-year heart failure (HF) 197](#_Toc144304804)

[4) 1-year Recurrent MI 200](#_Toc144304805)

[5) 1-year CV mortality 202](#_Toc144304806)

[6) 1-year Composite 1 outcome (HF, stroke, recurrent MI, & all-cause mortality) 204](#_Toc144304807)

[7) 1-year Composite 1 CV outcome (HF, stroke, recurrent MI, & CV mortality) 206](#_Toc144304808)

[8) 1-year Composite 2 outcome (HF, stroke, & all-cause mortality) 209](#_Toc144304809)

[9) 1-year Composite 2 CV outcome (HF, stroke, & CV mortality) 211](#_Toc144304810)

[External validation (CPRD GOLD) - with therapies 214](#_Toc144304811)

[1) 1-year all-cause mortality 214](#_Toc144304812)

[2) 1-year Stroke 217](#_Toc144304813)

[3) 1-year heart failure (HF) 219](#_Toc144304814)

[4) 1-year Recurrent MI 221](#_Toc144304815)

[5) 1-year CV mortality 223](#_Toc144304816)

[6) 1-year Composite 1 outcome (HF, stroke, recurrent MI, & all-cause mortality) 226](#_Toc144304817)

[7) 1-year Composite 1 CV outcome (HF, stroke, recurrent MI, & CV mortality) 228](#_Toc144304818)

[8) 1-year Composite 2 outcome (HF, stroke, & all-cause mortality) 230](#_Toc144304819)

[9) 1-year Composite 2 CV outcome (HF, stroke, & CV mortality) 232](#_Toc144304820)

[Internal validation (CPRD Aurum) - without therapies 235](#_Toc144304821)

[1) 5-year all-cause mortality 235](#_Toc144304822)

[2) 5-year Stroke 237](#_Toc144304823)

[3) 5-year heart failure (HF) 239](#_Toc144304824)

[4) 5-year Recurrent MI 241](#_Toc144304825)

[5) 5-year CV mortality 243](#_Toc144304826)

[6) 5-year Composite 1 outcome (HF, stroke, recurrent MI, & all-cause mortality) 245](#_Toc144304827)

[7) 5-year Composite 1 CV outcome (HF, stroke, recurrent MI, & CV mortality) 246](#_Toc144304828)

[8) 5-year Composite 2 outcome (HF, stroke, & all-cause mortality) 248](#_Toc144304829)

[9) 5-year Composite 2 CV outcome (HF, stroke, & CV mortality) 250](#_Toc144304830)

[External validation (CPRD GOLD) - without therapies 252](#_Toc144304831)

[1) 5-year all-cause mortality 252](#_Toc144304832)

[2) 5-year Stroke 254](#_Toc144304833)

[3) 5-year heart failure (HF) 256](#_Toc144304834)

[4) 5-year Recurrent MI 258](#_Toc144304835)

[5) 5-year CV mortality 260](#_Toc144304836)

[6) 5-year Composite 1 outcome (HF, stroke, recurrent MI, & all-cause mortality) 262](#_Toc144304837)

[7) 5-year Composite 1 CV outcome (HF, stroke, recurrent MI, & CV mortality) 264](#_Toc144304838)

[8) 5-year Composite 2 outcome (HF, stroke, & all-cause mortality) 266](#_Toc144304839)

[9) 5-year Composite 2 CV outcome (HF, stroke, & CV mortality) 268](#_Toc144304840)

[Internal validation (CPRD Aurum) - with therapies 271](#_Toc144304841)

[1) 5-year all-cause mortality 271](#_Toc144304842)

[2) 5-year Stroke 273](#_Toc144304843)

[3) 5-year heart failure (HF) 275](#_Toc144304844)

[4) 5-year Recurrent MI 277](#_Toc144304845)

[5) 5-year CV mortality 279](#_Toc144304846)

[6) 5-year Composite 1 outcome (HF, stroke, recurrent MI, & all-cause mortality) 281](#_Toc144304847)

[7) 5-year Composite 1 CV outcome (HF, stroke, recurrent MI, & CV mortality) 283](#_Toc144304848)

[8) 5-year Composite 2 outcome (HF, stroke, & all-cause mortality) 285](#_Toc144304849)

[9) 5-year Composite 2 CV outcome (HF, stroke, & CV mortality) 287](#_Toc144304850)

[External validation (CPRD GOLD) - with therapies 289](#_Toc144304851)

[1) 5-year all-cause mortality 289](#_Toc144304852)

[2) 5-year Stroke 291](#_Toc144304853)

[3) 5-year heart failure (HF) 294](#_Toc144304854)

[4) 5-year Recurrent MI 296](#_Toc144304855)

[5) 5-year CV mortality 298](#_Toc144304856)

[6) 5-year Composite 1 outcome (HF, stroke, recurrent MI, & all-cause mortality) 301](#_Toc144304857)

[7) 5-year Composite 1 CV outcome (HF, stroke, recurrent MI, & CV mortality) 303](#_Toc144304858)

[8) 5-year Composite 2 outcome (HF, stroke, & all-cause mortality) 305](#_Toc144304859)

[9) 5-year Composite 2 CV outcome (HF, stroke, & CV mortality) 307](#_Toc144304860)

# Baseline characteristics

Table S1. Outcomes and cohort characteristics, Audit models*

|  | **CPRD Aurum** | | **CPRD GOLD (external validation)** |
| --- | --- | --- | --- |
|  | **Training dataset (80%)** | **Validation dataset (20%) (internal validation)** |  |
| **Number of Patients, N** | 51,485 (79.45%) | 13,313 (20.55%) | 7,479 |
| **Number of general practices** | 1,011 (80%) | 253 (20%) | 122 |
| **1-year all-cause death** | 5,529 (10.74 %) | 1,519 (11.41 %) | 887 (11.86 %) |
| **5-year all-cause death** | 13,306 (25.84 %) | 3,491 (26.22 %) | 2,345 (31.35 %) |
| **1-year stroke** | 3,329 (6.47%) | 790 (5.93 %) | 396 (5.29 %) |
| **5-year stroke** | 11,199 (21.75 %) | 2,820 (21.18 %) | 1,858 (24.84 %) |
| **1-year heart failure (HF)** | 7,351 (14.28 %) | 1,860 (13.97%) | 500 (6.69 %) |
| **5-year heart failure (HF)** | 16,122 (31.31 %) | 4,282 (32.16 %) | 2,303 (30.79 %) |
| **1-year CV mortality** | 1,191 (2.31 %) | 310 (2.33 %) | 193 (2.58 %) |
| **5-year CV mortality** | 6,124 (11.89 %) | 1,748 (13.13 %) | 1,204 (16.10 %) |
| **1-year recurrent MI** | 1,553 (3.02 %) | 428 (3.21 %) | 285 (3.81%) |
| **5-year recurrent MI** | 7,181 (13.95 %) | 1,920 (14.42 %) | 1,611 (21.54%) |
| **1-year composite outcome†** | 13,716 (26.64 %) | 3,544 (26.62 %) | 1,586 (21.21 %) |
| **5-year composite outcome†** | 22,920 (44.52%) | 5,928 (44.53 %) | 3,628 (48.51%) |
| **1-year composite CV outcome§** | 10,826 (21.03 %) | 2,702 (20.30 %) | 964 (12.89 %) |
| **5-year composite CV outcome§** | 20,960 (40.71 %) | 5,413 (40.66 %) | 3,096 (41.40%) |
| **1-year composite MI outcome&** | 14,705 (28.56 %) | 3,799 (28.54 %) | 1,800 (24.07 %) |
| **5-year composite MI outcome&** | 24,301 (47.20 %) | 6,296 (47.29 %) | 3,790 (50.68 %) |
| **1-year composite MI CV outcome¥** | 11,826 (22.97%) | 3,015 (22.65 %) | 1,195 (15.98 %) |
| **5-year composite MI CV outcome¥** | 22,483 (43.67 %) | 5,894 (44.27 %) | 3,531 (47.21%) |
| **Age, mean (SD)** | 69.9 (12.6) | 70.0 (12.5) | 70.1 (12.6) |
| **Gender, N (%)**   - Female | 16,980 (32.98) | 4,337 (32.58) | 2,419 (32.34) |
| **IMD, N (%)**   - Q1 (Least deprived) - Q2 - Q3 - Q4 - Q5 (Most deprived) | 10,578 (20.55)  10,486 (20.37)  10,004 (19.43)  10,214 (19.84)  10,203 (19.82) | 2,827 (21.23)  2,946 (22.13)  2,843 (21.36)  2,389 (17.94)  2,308 (17.34) | 1,429 (19.11)  1,366 (18.26)  1,720 (23.00)  1,673 (22.37)  1,291 (17.26) |
| **Ethnicity, N (%)**   - White   Other than White | 47,388 (92.04)  4,097 (7.96) | 12,276 (92.21)  1,037 (7.79) | 7,106 (95.01)  373 (4.99) |
| **Alcohol consumption status, N (%)**   - Non/former drinker - Drinker | 8,019 (15.58)  43,466 (84.42) | 2,072 (15.56)  11,241 (84.44) | 1,858 (24.84)  5,621 (75.16) |
| **Smoking status, N (%)**   - Current smoker - Ex-smoker - Never smoker | 16,885 (32.80)  25,656 (49.83)  8,944 (17.37) | 4,352 (32.69)  6,811 (51.16)  2,150 (16.15) | 2,213 (29.59)  3,364 (44.98)  1,902 (25.43) |
| **BMI** (kg/m^2^) **, mean (SD)** | 28 (5.2) | 28 (5.3) | 31 (5.7) |
| **Duration from last AMI event, year mean (SD)** | 3.1 (3) | 3.2 (3) | 3.7 (3) |
| **≥1 previous AMI events, N (%)** | 51,453 (99.94) | 13,307 (99.95) | 7,476 (99.96) |
| **Comorbidities, N (%)**   - Hypertension | 22,279 (43.27) | 5,905 (44.36) | 4,126 (55.17) |
| - Hyperlipidaemia | 15,009 (29.15) | 4,177 (31.38) | 2,106 (28.16) |
| - Heart Failure | 7,177 (13.94) | 1,856 (13.94) | 1,376 (18.40) |
| - Atrial Fibrillation | 7,137 (13.86) | 1,890 (14.20) | 997 (13.33) |
| - Heart Valve Disease | 626 (1.22) | 166 (1.25) | 179 (2.39) |
| - Chronic Pulmonary Disease | 3,579 (6.95) | 904 (6.79) | 796 (10.64) |
| - Cardiomyopathy | 573 (1.11) | 147 (1.10) | 97 (1.30) |
| - Coronary Heart Disease (CHD) | 51,445 (99.92) | 13,308 (99.96) | 7,368 (98.52) |
| - VT/VF | 692 (1.34) | 174 (1.31) | 75 (1.00) |
| - PVD | 3,579 (6.95) | 904 (6.79) | 522 (6.98) |
| - Stroke + TIA | 5,901 (11.46) | 1,521 (11.42) | 786(10.51) |
| - CV procedures | 24,675 (47.93) | 6,468 (48.58) | 3,525 (47.13) |
| - Chronic Kidney Disease | 12,572 (24.42) | 3,339 (25.08) | 1,742 (23.29) |
| - Diabetes   - T1DM   - T2DM   - Unspecified | 315 (0.61)  11,377 (22.10)  638 (1.24) | 90 (0.68)  2,914 (21.89)  206 (1.55) | 61 (0.82)  1,568 (20.97)  103 (1.38) |
| - Hypothyroidism | 4,650 (9.03) | 1,194 (8.97) | 706 (9.44) |
| - Liver disease | 130 (0.25) | 34 (0.26) | 51 (0.68) |
| - Lupus | 172 (0.33) | 41 (0.31) | 19 (0.25) |
| - Erectile dysfunction | 8,814 (17.12) | 2,233 (16.77) | 1,345 (17.98) |
| - HIV_AIDS | 58 (0.11) | 17 (0.13) | <5 |
| - Menopause | 5,253 (10.20) | 1,288 (9.67) | 876 (11.71) |
| - Rheumatoid arthritis | 1,861 (3.61) | 450 (3.38) | 147 (1.97) |
| - Dementia | 1,702 (3.31) | 459 (3.45) | 253 (3.38) |
| - Any tumour | 8,974 (17.43) | 2,280 (17.13) | 1,242 (16.61) |
| **Medications, N (%)**   - Lipid-reg agents | 50,266 (97.63) | 13,013 (97.75) | 7,288 (97.45) |
| - Diuretics | 26,733 (51.92) | 7,095 (53.29) | 4,023 (53.79) |
| - Antihypertensives | 50,970 (99.00) | 13,202 (99.17) | 7,389 (98.80) |
| - Antidiabetic drugs | 10,035 (19.49) | 2,586 (19.42) | 1,472 (19.68) |
| - Anticoagulants | 6,555 (12.73) | 1,730 (12.99) | 1,288 (17.22) |
| - Antiplatelets | 50,381 (97.86) | 13,071 (98.18) | 7,320 (97.87) |

* includes imputed values for outcomes, ethnicity, deprivation, BMI, smoking status, and alcohol consumption

† stroke, heart failure and all-cause death

§ stroke, heart failure and cardiovascular-related death

& stroke, heart failure, recurrent MI, and all-cause death

**¥** stroke, heart failure, recurrent MI, and cardiovascular-related death

Table S2: Performance summary, Index models not including therapies

|  | **Internal validation (Aurum)** | | | **External validation (GOLD)** | | |
| --- | --- | --- | --- | --- | --- | --- |
| **Outcome** | **AUROC**  **(95% CI)** | **Correctly classified** | **Calibration slope** | **AUROC**  **(95% CI)** | **Correctly classified** | **Calibration slope** |
| 1-year all-cause mortality | 0.799 (0.790; 0.809) | 77.07% | 0.980 | 0.793 (0.782; 0.803) | 77.19% | 0.946 |
| 5-year all-cause mortality | 0.843 (0.837; 0.849) | 77.83% | 0.973 | 0.837 (0.831; 0.843) | 76.91% | 0.967 |
| 1-year stroke | 0.800 (0.787; 0.813) | 87.41% | 0.972 | 0.776 (0.761; 0.791) | 85.53% | 0.847 |
| 5-year stroke | 0.831 (0.824; 0.838) | 79.02% | 0.974 | 0.827 (0.820; 0.834) | 77.95% | 0.974 |
| 1-year heart failure | 0.728 (0.718; 0.739) | 74.34% | 0.955 | 0.705 (0.694; 0.715) | 73.24% | 0.794 |
| 5-year heart failure | 0.793 (0.786; 0.800) | 73.68% | 0.947 | 0.776 (0.769; 0.783) | 71.50% | 0.886 |
| 1-year recurrent MI | 0.568 (0.555; 0.581) | 60.12% | 0.840 | 0.567 (0.558; 0.577) | 57.22% | 0.827 |
| 5-year recurrent MI | 0.675 (0.665; 0.684) | 66.83% | 0.971 | 0.646 (0.638; 0.654) | 58.04% | 0.840 |
| 1-year CV mortality | 0.794 (0.780; 0.807) | 78.62% | 0.955 | 0.792 (0.778; 0.806) | 78.45% | 0.923 |
| 5-year CV mortality | 0.854 (0.847; 0.860) | 80.95% | 0.984 | 0.851 (0.843; 0.857) | 80.05% | 0.979 |
| 1-year composite outcome† | 0.760 (0.752; 0.768) | 72.64% | 0.944 | 0.744 (0.736; 0.753) | 71.54% | 0.862 |
| 1-year composite CV outcome§ | 0.756 (0.748; 0.765) | 73.35% | 0.964 | 0.734 (0.725; 0.743) | 71.85% | 0.843 |
| 5-year composite outcome† | 0.821 (0.815; 0.827) | 74.70% | 0.971 | 0.805 (0.799; 0.812) | 73.18% | 0.927 |
| 5-year composite CV outcome§ | 0.812 (0.806; 0.818) | 74.40% | 0.958 | 0.795 (0.788; 0.802) | 72.28% | 0.898 |
| 1-year composite MI outcome& | 0.707 (0.699; 0.715) | 67.87% | 0.933 | 0.657 (0.649; 0.665) | 61.70% | 0.708 |
| 5-year composite MI outcome& | 0.780 (0.774; 0.787) | 71.41% | 0.940 | 0.724 (0.715; 0.733) | 64.27% | 0.742 |
| 1-year composite MI CV outcome¥ | 0.697 (0.689; 0.706) | 67.92% | 0.945 | 0.648 (0.641; 0.656) | 61.28% | 0.715 |
| 5-year composite MI CV outcome¥ | 0.774 (0.767; 0.781) | 71.04% | 0.963 | 0.716 (0.707; 0.724) | 63.21% | 0.747 |

† stroke, heart failure and all-cause death

§ stroke, heart failure and cardiovascular-related death

& stroke, heart failure, recurrent MI, and all-cause death

**¥** stroke, heart failure, recurrent MI, and cardiovascular-related death

Table S3: Performance summary, Audit models not including therapies

|  | **Internal validation (Aurum)** | | | **External validation (GOLD)** | | |
| --- | --- | --- | --- | --- | --- | --- |
| **Outcome** | **AUROC**  **(95% CI)** | **Correctly classified** | **Calibration slope** | **AUROC**  **(95% CI)** | **Correctly classified** | **Calibration slope** |
| 1-year all-cause mortality | 0.886  (0.878; 0.895) | 85.91% | 0.997 | 0.870  (0.858; 0.882) | 85.15% | 0.933 |
| 5-year all-cause mortality | 0.851  (0.844; 0.858) | 79.14% | 1.030 | 0.840  (0.830; 0.849) | 77.68% | 0.999 |
| 1-year stroke | 0.855  (0.841; 0.869) | 89.33% | 0.994 | 0.804  (0.782; 0.826) | 88.29% | 0.827 |
| 5-year stroke | 0.834  (0.826; 0.843) | 80.18% | 0.997 | 0.816  (0.805; 0.826) | 77.08 % | 0.966 |
| 1-year heart failure | 0.871  (0.861; 0.880) | 86.23% | 0.959 | 0.718  (0.699; 0.741) | 78.91% | 0.414 |
| 5-year heart failure | 0.846  (0.839; 0.854) | 78.75% | 1.053 | 0.771  (0.760; 0.782) | 72.70 % | 0.643 |
| 1-year recurrent MI | 0.688  (0.661; 0.716) | 75.29% | 1.022 | 0.671  (0.637; 0.705) | 73.85% | 0.887 |
| 5-year recurrent MI | 0.716  (0.704; 0.729) | 72.63% | 1.062 | 0.713  (0.698; 0.727) | 71.07 % | 1.119 |
| 1-year CV mortality | 0.825  (0.802; 0.849) | 85.38% | 1.012 | 0.817  (0.787; 0.847) | 84.28% | 0.963 |
| 5-year CV mortality | 0.855  (0.845; 0.865) | 82.73% | 1.049 | 0.854  (0.843; 0.866) | 81.88 % | 1.086 |
| 1-year composite outcome† | 0.883  (0.876; 0.890) | 82.46% | 1.017 | 0.828  (0.816; 0.840) | 78.09% | 0.698 |
| 5-year composite outcome† | 0.840  (0.833; 0.847) | 76.79% | 1.051 | 0.793  (0.783; 0.803) | 72.42% | 0.795 |
| 1-year composite CV outcome§ | 0.862  (0.854; 0.870) | 82.54% | 0.963 | 0.764  (0.748; 0.779) | 76.40% | 0.526 |
| 5-year composite CV outcome§ | 0.834  (0.828; 0.841) | 76.45% | 1.027 | 0.789  (0.779; 0.799) | 72.48% | 0.763 |
| 1-year composite MI outcome& | 0.865  (0.858; 0.872) | 80.84% | 1.001 | 0.813  (0.801; 0.825) | 76.67% | 0.712 |
| 5-year composite MI outcome& | 0.819  (0.812; 0.825) | 74.83% | 1.034 | 0.775  (0.766; 0.785) | 70.38% | 0.800 |
| 1-year composite MI CV outcome¥ | 0.843  (0.835; 0.851) | 80.85% | 0.970 | 0.757  (0.743; 0.772) | 75.30% | 0.583 |
| 5-year composite MI CV outcome¥ | 0.815  (0.808; 0.822) | 74.82% | 1.037 | 0.759  (0.748; 0.771) | 69.84% | 0.736 |

† stroke, heart failure and all-cause death

§ stroke, heart failure and cardiovascular-related death

& stroke, heart failure, recurrent MI, and all-cause death

**¥** stroke, heart failure, recurrent MI, and cardiovascular-related death

# Internal and external validation - Index models

## Internal validation (CPRD Aurum) - without therapies

### 1) 1-year all-cause mortality

| _1year_All_cause_death | Odds ratio | Std. err. | z | P>|z| | [95% conf. interval] | |
| --- | --- | --- | --- | --- | --- | --- |
| age | 1.076 | 0.002 | 50.90 | 0.000 | 1.073 | 1.079 |
|  |  |  |  |  |  |  |
| gender |  |  |  |  |  |  |
| Female | 0.947 | 0.028 | -1.83 | 0.067 | 0.893 | 1.004 |
|  |  |  |  |  |  |  |
| IMD |  |  |  |  |  |  |
| Q2 | 1.081 | 0.043 | 1.95 | 0.051 | 1.000 | 1.170 |
| Q3 | 1.189 | 0.048 | 4.30 | 0.000 | 1.099 | 1.286 |
| Q4 | 1.260 | 0.051 | 5.69 | 0.000 | 1.164 | 1.364 |
| Q5-most deprived | 1.334 | 0.055 | 6.95 | 0.000 | 1.230 | 1.447 |
|  |  |  |  |  |  |  |
| Other than White | 0.842 | 0.052 | -2.76 | 0.006 | 0.746 | 0.951 |
| Drinker | 0.810 | 0.025 | -6.71 | 0.000 | 0.762 | 0.861 |
|  |  |  |  |  |  |  |
| Smoking_status |  |  |  |  |  |  |
| current smoker | 1.227 | 0.047 | 5.40 | 0.000 | 1.139 | 1.322 |
| ex-smoker | 0.875 | 0.029 | -4.06 | 0.000 | 0.820 | 0.933 |
|  |  |  |  |  |  |  |
| BMI | 0.972 | 0.003 | -10.60 | 0.000 | 0.967 | 0.977 |
| HT_b | 0.983 | 0.026 | -0.65 | 0.513 | 0.933 | 1.035 |
| Hyperlipidaemia_b | 0.909 | 0.029 | -2.95 | 0.003 | 0.853 | 0.969 |
|  |  |  |  |  |  |  |
| Diabetes_type_b |  |  |  |  |  |  |
| T1DM | 3.391 | 0.427 | 9.71 | 0.000 | 2.650 | 4.340 |
| T2DM | 1.419 | 0.047 | 10.53 | 0.000 | 1.330 | 1.515 |
| DM - nos | 2.047 | 0.188 | 7.82 | 0.000 | 1.711 | 2.450 |
|  |  |  |  |  |  |  |
| HF_b | 1.897 | 0.074 | 16.46 | 0.000 | 1.757 | 2.047 |
| AF_b | 1.296 | 0.045 | 7.40 | 0.000 | 1.210 | 1.388 |
| Heart_valve_dis_b | 1.396 | 0.122 | 3.80 | 0.000 | 1.175 | 1.657 |
| VT_VF_b | 0.839 | 0.158 | -0.93 | 0.351 | 0.580 | 1.213 |
| Cardiomyopathy_b | 1.231 | 0.193 | 1.33 | 0.185 | 0.906 | 1.673 |
| CV_procedures_b | 0.812 | 0.037 | -4.63 | 0.000 | 0.744 | 0.887 |
| TIA_stroke_b | 1.340 | 0.045 | 8.68 | 0.000 | 1.254 | 1.432 |
| PVD_b | 1.666 | 0.069 | 12.29 | 0.000 | 1.536 | 1.808 |
| CKD_b | 1.320 | 0.038 | 9.63 | 0.000 | 1.247 | 1.397 |
| Hypothyroidism_b | 1.025 | 0.043 | 0.59 | 0.556 | 0.944 | 1.112 |
| Liver_dis_b | 1.694 | 0.503 | 1.77 | 0.076 | 0.946 | 3.032 |
| Lupus_b | 1.412 | 0.299 | 1.63 | 0.103 | 0.932 | 2.138 |
| Erectile_dysfunction_b | 0.844 | 0.044 | -3.23 | 0.001 | 0.761 | 0.936 |
| Any_tumour_b | 1.502 | 0.044 | 13.78 | 0.000 | 1.418 | 1.592 |
| Menopause_b | 0.819 | 0.041 | -3.95 | 0.000 | 0.741 | 0.904 |
| Dementia_b | 1.650 | 0.095 | 8.66 | 0.000 | 1.473 | 1.848 |
| RA_b | 1.305 | 0.082 | 4.24 | 0.000 | 1.154 | 1.477 |
| _cons | 0.001 | 0.000 | -47.63 | 0.000 | 0.001 | 0.001 |

**Performance in validation dataset (20% of practices) - Internal validation - Index AMI cases**

| AUROC (95% CI): 0.799 (0.790; 0.809) |  |
| --- | --- |
| 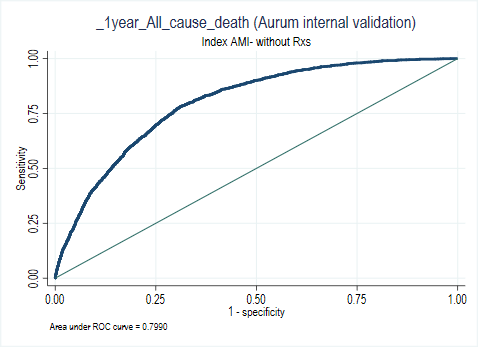 | 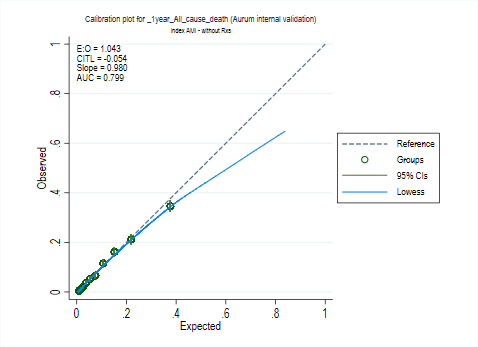 |
| 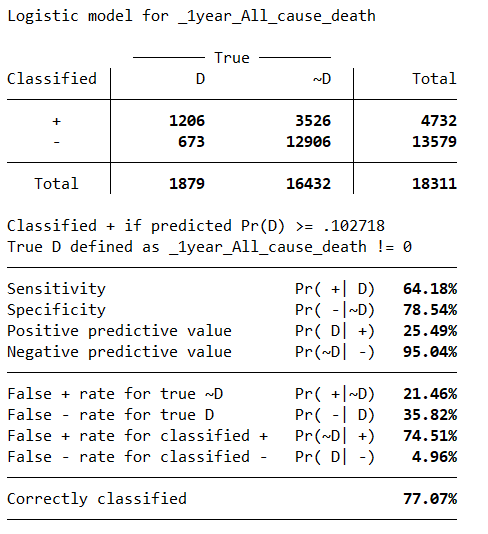 | % Correctly classified: 77.07 |

### 2) 1-year Stroke

| _1year_Stroke | Odds ratio | Std. err. | z | P>|z| | [95% conf. interval] | |
| --- | --- | --- | --- | --- | --- | --- |
| age | 1.040 | 0.002 | 23.61 | 0.000 | 1.037 | 1.043 |
|  |  |  |  |  |  |  |
| gender |  |  |  |  |  |  |
| Female | 1.119 | 0.042 | 3.02 | 0.003 | 1.040 | 1.203 |
|  |  |  |  |  |  |  |
| IMD |  |  |  |  |  |  |
| Q2 | 0.929 | 0.046 | -1.49 | 0.136 | 0.843 | 1.023 |
| Q3 | 1.009 | 0.050 | 0.17 | 0.863 | 0.915 | 1.111 |
| Q4 | 1.097 | 0.054 | 1.87 | 0.062 | 0.995 | 1.208 |
| Q5-most deprived | 1.125 | 0.057 | 2.35 | 0.019 | 1.020 | 1.242 |
|  |  |  |  |  |  |  |
| Other than White | 0.958 | 0.067 | -0.62 | 0.536 | 0.835 | 1.098 |
| Drinker | 0.856 | 0.033 | -4.01 | 0.000 | 0.793 | 0.923 |
|  |  |  |  |  |  |  |
| Smoking_status |  |  |  |  |  |  |
| current smoker | 1.104 | 0.052 | 2.10 | 0.036 | 1.007 | 1.211 |
| ex-smoker | 0.927 | 0.038 | -1.84 | 0.066 | 0.855 | 1.005 |
|  |  |  |  |  |  |  |
| BMI | 0.989 | 0.003 | -3.68 | 0.000 | 0.983 | 0.995 |
| HT_b | 1.202 | 0.039 | 5.63 | 0.000 | 1.127 | 1.281 |
| Hyperlipidaemia_b | 1.127 | 0.042 | 3.25 | 0.001 | 1.049 | 1.212 |
|  |  |  |  |  |  |  |
| Diabetes_type_b |  |  |  |  |  |  |
| T1DM | 2.927 | 0.411 | 7.65 | 0.000 | 2.223 | 3.854 |
| T2DM | 1.399 | 0.055 | 8.51 | 0.000 | 1.295 | 1.511 |
| DM - nos | 1.579 | 0.172 | 4.19 | 0.000 | 1.275 | 1.955 |
|  |  |  |  |  |  |  |
| HF_b | 1.247 | 0.062 | 4.42 | 0.000 | 1.131 | 1.375 |
| AF_b | 1.455 | 0.061 | 8.98 | 0.000 | 1.341 | 1.580 |
| Heart_valve_dis_b | 1.221 | 0.129 | 1.88 | 0.060 | 0.992 | 1.502 |
| VT_VF_b | 1.086 | 0.223 | 0.40 | 0.689 | 0.725 | 1.625 |
| Cardiomyopathy_b | 1.702 | 0.292 | 3.10 | 0.002 | 1.216 | 2.382 |
| CV_procedures_b | 0.857 | 0.045 | -2.95 | 0.003 | 0.773 | 0.949 |
| TIA_stroke_b | 6.857 | 0.227 | 58.08 | 0.000 | 6.426 | 7.317 |
| PVD_b | 1.488 | 0.074 | 7.97 | 0.000 | 1.349 | 1.640 |
| CKD_b | 1.072 | 0.039 | 1.92 | 0.054 | 0.999 | 1.150 |
| Hypothyroidism_b | 1.023 | 0.053 | 0.45 | 0.653 | 0.925 | 1.132 |
| Liver_dis_b | 0.867 | 0.361 | -0.34 | 0.731 | 0.383 | 1.962 |
| Lupus_b | 1.096 | 0.282 | 0.36 | 0.722 | 0.662 | 1.816 |
| Erectile_dysfunction_b | 1.057 | 0.062 | 0.95 | 0.341 | 0.943 | 1.186 |
| Any_tumour_b | 1.057 | 0.041 | 1.41 | 0.159 | 0.979 | 1.141 |
| Menopause_b | 0.904 | 0.053 | -1.70 | 0.089 | 0.805 | 1.015 |
| Dementia_b | 1.265 | 0.094 | 3.16 | 0.002 | 1.093 | 1.463 |
| RA_b | 1.000 | 0.082 | -0.00 | 0.999 | 0.851 | 1.175 |
| _cons | 0.003 | 0.000 | -33.60 | 0.000 | 0.002 | 0.004 |

**Performance in validation dataset (20% of practices) - Internal validation - Index AMI cases**

| AUROC (95% CI): 0.800 (0.787; 0.813) |  |
| --- | --- |
| 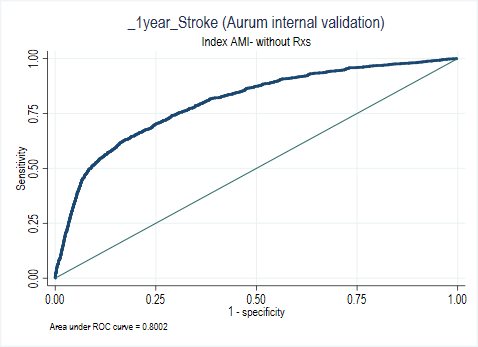 | 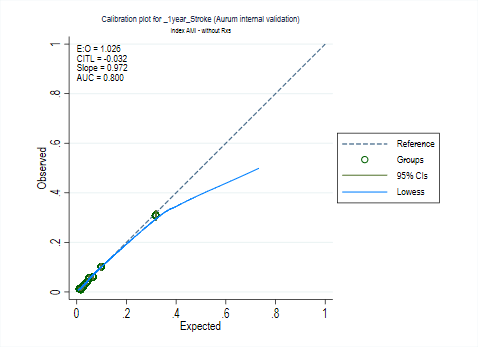 |
| 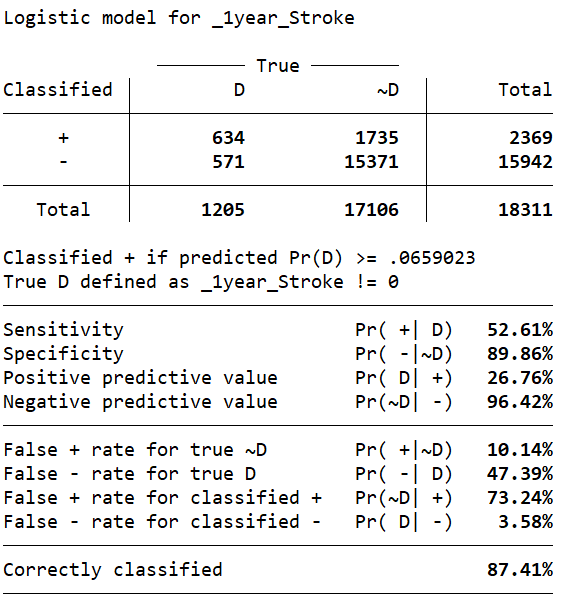 | % Correctly classified: 87.41 |

### 3) 1- year heart failure (HF)

| _1year_HF | Odds ratio | Std. err. | z | P>|z| | [95% conf. interval] | |
| --- | --- | --- | --- | --- | --- | --- |
| age | 1.041 | 0.001 | 37.98 | 0.000 | 1.039 | 1.043 |
|  |  |  |  |  |  |  |
| gender |  |  |  |  |  |  |
| Female | 0.946 | 0.024 | -2.16 | 0.031 | 0.900 | 0.995 |
|  |  |  |  |  |  |  |
| IMD |  |  |  |  |  |  |
| Q2 | 1.107 | 0.037 | 3.03 | 0.002 | 1.037 | 1.182 |
| Q3 | 1.091 | 0.037 | 2.55 | 0.011 | 1.020 | 1.165 |
| Q4 | 1.183 | 0.040 | 4.95 | 0.000 | 1.107 | 1.265 |
| Q5-most deprived | 1.350 | 0.046 | 8.78 | 0.000 | 1.263 | 1.444 |
|  |  |  |  |  |  |  |
| Other than White | 1.045 | 0.047 | 0.98 | 0.327 | 0.957 | 1.141 |
| Drinker | 0.837 | 0.023 | -6.58 | 0.000 | 0.794 | 0.882 |
|  |  |  |  |  |  |  |
| Smoking_status |  |  |  |  |  |  |
| current smoker | 1.160 | 0.037 | 4.60 | 0.000 | 1.089 | 1.236 |
| ex-smoker | 1.016 | 0.029 | 0.57 | 0.569 | 0.961 | 1.075 |
|  |  |  |  |  |  |  |
| BMI | 1.005 | 0.002 | 2.63 | 0.009 | 1.001 | 1.009 |
| HT_b | 1.098 | 0.024 | 4.19 | 0.000 | 1.051 | 1.146 |
| Hyperlipidaemia_b | 0.938 | 0.025 | -2.40 | 0.016 | 0.890 | 0.988 |
|  |  |  |  |  |  |  |
| Diabetes_type_b |  |  |  |  |  |  |
| T1DM | 2.332 | 0.249 | 7.95 | 0.000 | 1.893 | 2.874 |
| T2DM | 1.423 | 0.039 | 12.76 | 0.000 | 1.348 | 1.502 |
| DM - nos | 2.091 | 0.160 | 9.63 | 0.000 | 1.799 | 2.429 |
|  |  |  |  |  |  |  |
| HF_b | 5.248 | 0.178 | 48.82 | 0.000 | 4.910 | 5.610 |
| AF_b | 1.420 | 0.045 | 11.15 | 0.000 | 1.335 | 1.510 |
| Heart_valve_dis_b | 1.381 | 0.112 | 3.99 | 0.000 | 1.178 | 1.617 |
| VT_VF_b | 1.102 | 0.166 | 0.64 | 0.520 | 0.820 | 1.479 |
| Cardiomyopathy_b | 1.815 | 0.217 | 4.98 | 0.000 | 1.436 | 2.295 |
| CV_procedures_b | 0.830 | 0.030 | -5.15 | 0.000 | 0.773 | 0.891 |
| TIA_stroke_b | 1.089 | 0.034 | 2.73 | 0.006 | 1.024 | 1.157 |
| PVD_b | 1.473 | 0.055 | 10.33 | 0.000 | 1.369 | 1.586 |
| CKD_b | 1.256 | 0.032 | 8.90 | 0.000 | 1.194 | 1.320 |
| Hypothyroidism_b | 1.022 | 0.038 | 0.59 | 0.557 | 0.951 | 1.098 |
| Liver_dis_b | 1.235 | 0.301 | 0.86 | 0.388 | 0.765 | 1.992 |
| Lupus_b | 1.568 | 0.267 | 2.65 | 0.008 | 1.124 | 2.189 |
| Erectile_dysfunction_b | 0.982 | 0.038 | -0.46 | 0.648 | 0.911 | 1.060 |
| Any_tumour_b | 1.116 | 0.030 | 4.03 | 0.000 | 1.058 | 1.177 |
| Menopause_b | 0.801 | 0.034 | -5.25 | 0.000 | 0.738 | 0.870 |
| Dementia_b | 0.833 | 0.052 | -2.91 | 0.004 | 0.737 | 0.942 |
| RA_b | 1.106 | 0.062 | 1.82 | 0.069 | 0.992 | 1.234 |
| _cons | 0.006 | 0.001 | -45.20 | 0.000 | 0.005 | 0.008 |

**Performance in validation dataset (20% of practices) - Internal validation - Index AMI cases**

| AUROC (95% CI): 0.728 (0.718; 0.739) |  |
| --- | --- |
| 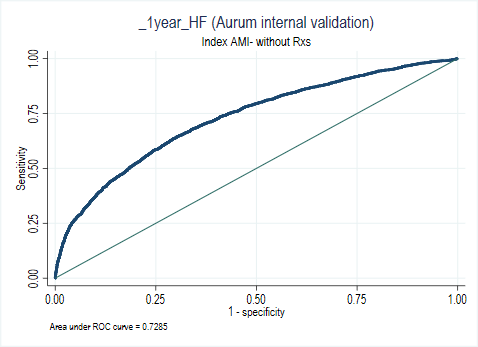 | 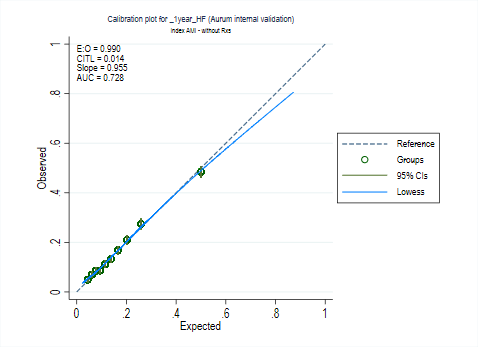 |
| 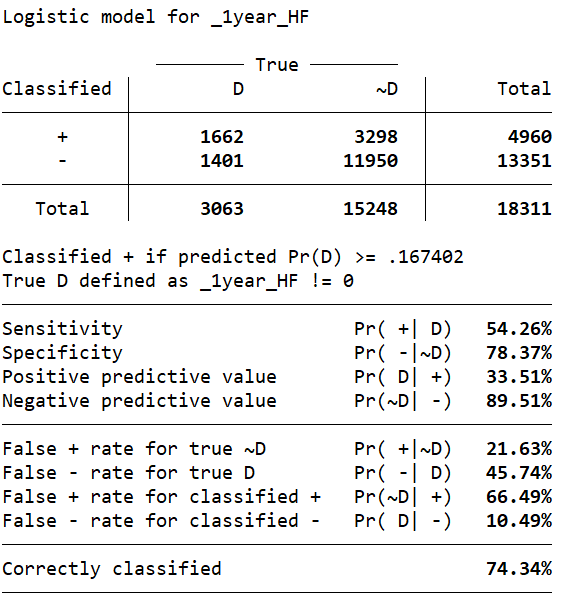 | % Correctly classified: 74.34 |

### 4) 1-year Recurrent MI

| _1year_Recurrent_MI | Odds ratio | Std. err. | z | P>|z| | [95% conf. interval] | |
| --- | --- | --- | --- | --- | --- | --- |
| age | 1.012 | 0.001 | 11.27 | 0.000 | 1.010 | 1.014 |
|  |  |  |  |  |  |  |
| gender |  |  |  |  |  |  |
| Female | 1.013 | 0.028 | 0.46 | 0.642 | 0.959 | 1.070 |
|  |  |  |  |  |  |  |
| IMD |  |  |  |  |  |  |
| Q2 | 1.039 | 0.037 | 1.06 | 0.290 | 0.968 | 1.114 |
| Q3 | 1.003 | 0.036 | 0.07 | 0.942 | 0.934 | 1.077 |
| Q4 | 1.002 | 0.037 | 0.06 | 0.952 | 0.933 | 1.077 |
| Q5-most deprived | 1.062 | 0.039 | 1.62 | 0.106 | 0.987 | 1.141 |
|  |  |  |  |  |  |  |
| Other than White | 1.076 | 0.051 | 1.56 | 0.120 | 0.981 | 1.180 |
| Drinker | 0.971 | 0.030 | -0.97 | 0.330 | 0.914 | 1.031 |
|  |  |  |  |  |  |  |
| Smoking_status |  |  |  |  |  |  |
| current smoker | 1.048 | 0.036 | 1.36 | 0.173 | 0.980 | 1.122 |
| ex-smoker | 0.962 | 0.030 | -1.25 | 0.211 | 0.905 | 1.022 |
|  |  |  |  |  |  |  |
| BMI | 0.995 | 0.002 | -2.13 | 0.033 | 0.991 | 1.000 |
| HT_b | 1.054 | 0.026 | 2.16 | 0.031 | 1.005 | 1.106 |
| Hyperlipidaemia_b | 0.977 | 0.029 | -0.81 | 0.421 | 0.922 | 1.035 |
|  |  |  |  |  |  |  |
| Diabetes_type_b |  |  |  |  |  |  |
| T1DM | 1.329 | 0.161 | 2.34 | 0.019 | 1.047 | 1.686 |
| T2DM | 1.118 | 0.036 | 3.51 | 0.000 | 1.050 | 1.190 |
| DM - nos | 1.367 | 0.122 | 3.50 | 0.000 | 1.148 | 1.629 |
|  |  |  |  |  |  |  |
| HF_b | 1.193 | 0.053 | 3.98 | 0.000 | 1.094 | 1.301 |
| AF_b | 1.013 | 0.039 | 0.32 | 0.748 | 0.938 | 1.093 |
| Heart_valve_dis_b | 1.149 | 0.112 | 1.43 | 0.152 | 0.950 | 1.390 |
| VT_VF_b | 0.575 | 0.129 | -2.47 | 0.013 | 0.371 | 0.892 |
| Cardiomyopathy_b | 0.765 | 0.134 | -1.53 | 0.126 | 0.542 | 1.078 |
| CV_procedures_b | 0.735 | 0.031 | -7.31 | 0.000 | 0.676 | 0.798 |
| TIA_stroke_b | 1.099 | 0.040 | 2.62 | 0.009 | 1.024 | 1.179 |
| PVD_b | 1.368 | 0.059 | 7.31 | 0.000 | 1.258 | 1.489 |
| CKD_b | 1.151 | 0.034 | 4.70 | 0.000 | 1.085 | 1.221 |
| Hypothyroidism_b | 1.058 | 0.044 | 1.36 | 0.174 | 0.975 | 1.148 |
| Liver_dis_b | 1.596 | 0.358 | 2.09 | 0.037 | 1.029 | 2.477 |
| Lupus_b | 1.414 | 0.255 | 1.92 | 0.055 | 0.993 | 2.013 |
| Erectile_dysfunction_b | 0.968 | 0.041 | -0.76 | 0.445 | 0.892 | 1.051 |
| Any_tumour_b | 1.037 | 0.032 | 1.17 | 0.244 | 0.975 | 1.103 |
| Menopause_b | 0.865 | 0.039 | -3.19 | 0.001 | 0.792 | 0.946 |
| Dementia_b | 0.983 | 0.071 | -0.24 | 0.811 | 0.853 | 1.132 |
| RA_b | 0.844 | 0.057 | -2.53 | 0.011 | 0.740 | 0.963 |
| _cons | 0.058 | 0.007 | -24.44 | 0.000 | 0.046 | 0.073 |

**Performance in validation dataset (20% of practices) - Internal validation - Index AMI cases**

| AUROC (95% CI): 0.568 (0.555; 0.581) |  |
| --- | --- |
| 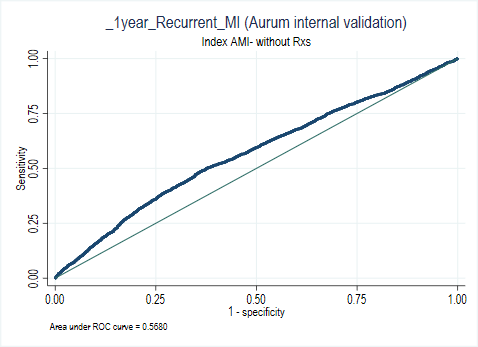 | 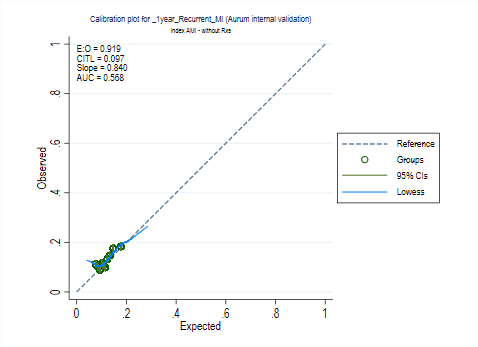 |
| 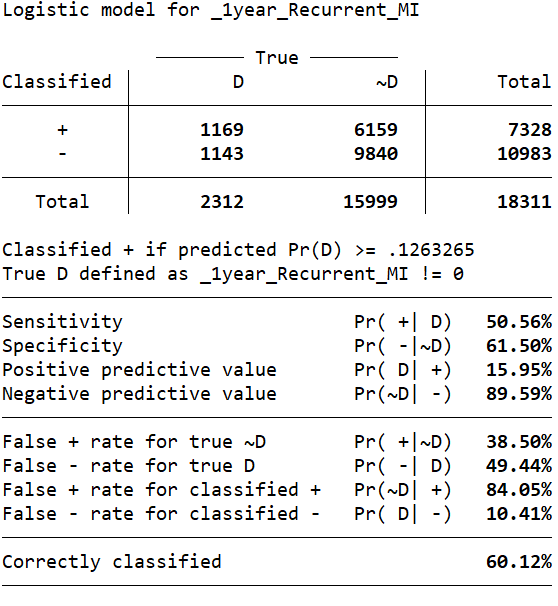 | % Correctly classified: 60.12 |

### 5) 1-year CV mortality

| _1year_CV_death | Odds ratio | Std. err. | z | P>|z| | [95% conf. interval] | |
| --- | --- | --- | --- | --- | --- | --- |
| age | 1.077 | 0.002 | 37.86 | 0.000 | 1.073 | 1.081 |
|  |  |  |  |  |  |  |
| gender |  |  |  |  |  |  |
| Female | 0.900 | 0.035 | -2.66 | 0.008 | 0.834 | 0.973 |
|  |  |  |  |  |  |  |
| IMD |  |  |  |  |  |  |
| Q2 | 1.012 | 0.054 | 0.22 | 0.829 | 0.911 | 1.123 |
| Q3 | 1.107 | 0.059 | 1.92 | 0.055 | 0.998 | 1.229 |
| Q4 | 1.174 | 0.063 | 3.00 | 0.003 | 1.057 | 1.304 |
| Q5-most deprived | 1.174 | 0.065 | 2.89 | 0.004 | 1.053 | 1.308 |
|  |  |  |  |  |  |  |
| Other than White | 0.838 | 0.070 | -2.10 | 0.035 | 0.711 | 0.988 |
| Drinker | 0.804 | 0.033 | -5.32 | 0.000 | 0.742 | 0.871 |
|  |  |  |  |  |  |  |
| Smoking_status |  |  |  |  |  |  |
| current smoker | 1.080 | 0.054 | 1.55 | 0.122 | 0.980 | 1.191 |
| ex-smoker | 0.798 | 0.034 | -5.25 | 0.000 | 0.734 | 0.868 |
|  |  |  |  |  |  |  |
| BMI | 0.985 | 0.003 | -4.45 | 0.000 | 0.978 | 0.991 |
| HT_b | 1.078 | 0.038 | 2.13 | 0.033 | 1.006 | 1.155 |
| Hyperlipidaemia_b | 0.953 | 0.041 | -1.12 | 0.262 | 0.877 | 1.036 |
|  |  |  |  |  |  |  |
| Diabetes_type_b |  |  |  |  |  |  |
| T1DM | 4.240 | 0.628 | 9.76 | 0.000 | 3.172 | 5.668 |
| T2DM | 1.415 | 0.061 | 8.02 | 0.000 | 1.300 | 1.540 |
| DM - nos | 1.999 | 0.231 | 5.98 | 0.000 | 1.593 | 2.508 |
|  |  |  |  |  |  |  |
| HF_b | 2.070 | 0.097 | 15.57 | 0.000 | 1.889 | 2.269 |
| AF_b | 1.443 | 0.063 | 8.41 | 0.000 | 1.325 | 1.572 |
| Heart_valve_dis_b | 1.508 | 0.158 | 3.92 | 0.000 | 1.228 | 1.851 |
| VT_VF_b | 1.032 | 0.233 | 0.14 | 0.890 | 0.663 | 1.606 |
| Cardiomyopathy_b | 1.252 | 0.244 | 1.15 | 0.249 | 0.855 | 1.834 |
| CV_procedures_b | 0.891 | 0.052 | -1.97 | 0.049 | 0.795 | 1.000 |
| TIA_stroke_b | 1.271 | 0.055 | 5.54 | 0.000 | 1.168 | 1.384 |
| PVD_b | 1.753 | 0.091 | 10.82 | 0.000 | 1.584 | 1.941 |
| CKD_b | 1.261 | 0.047 | 6.17 | 0.000 | 1.171 | 1.357 |
| Hypothyroidism_b | 0.995 | 0.055 | -0.09 | 0.932 | 0.894 | 1.108 |
| Liver_dis_b | 1.562 | 0.640 | 1.09 | 0.276 | 0.700 | 3.485 |
| Lupus_b | 2.150 | 0.530 | 3.10 | 0.002 | 1.326 | 3.486 |
| Erectile_dysfunction_b | 0.814 | 0.059 | -2.85 | 0.004 | 0.706 | 0.938 |
| Any_tumour_b | 1.019 | 0.042 | 0.45 | 0.650 | 0.940 | 1.104 |
| Menopause_b | 0.715 | 0.052 | -4.63 | 0.000 | 0.620 | 0.824 |
| Dementia_b | 1.361 | 0.100 | 4.18 | 0.000 | 1.178 | 1.573 |
| RA_b | 1.210 | 0.103 | 2.25 | 0.025 | 1.025 | 1.429 |
| _cons | 0.000 | 0.000 | -40.07 | 0.000 | 0.000 | 0.000 |

**Performance in validation dataset (20% of practices) - Internal validation - Index AMI cases**

| AUROC (95% CI): 0.794 (0.780; 0.807) |  |
| --- | --- |
| 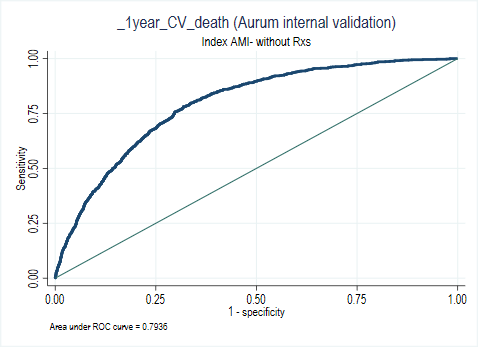 | 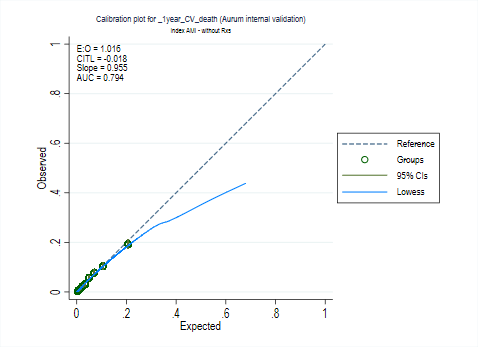 |
| 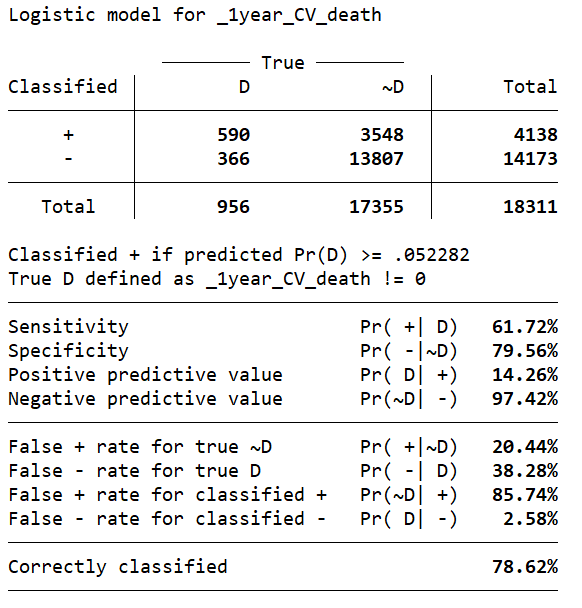 | % Correctly classified: 78.62 |

### 6) 1-year Composite 1 outcome (HF, stroke, recurrent MI, & all-cause mortality)

|  | | | | | | |
| --- | --- | --- | --- | --- | --- | --- |
| _1yr_Comp1_outcome | Odds ratio | Std. err. | z | P>|z| | [95% conf. interval] | |
| age | 1.037 | 0.001 | 44.57 | 0.000 | 1.035 | 1.038 |
|  |  |  |  |  |  |  |
| gender |  |  |  |  |  |  |
| Female | 1.013 | 0.021 | 0.62 | 0.535 | 0.973 | 1.054 |
|  |  |  |  |  |  |  |
| IMD |  |  |  |  |  |  |
| Q2 | 1.067 | 0.028 | 2.46 | 0.014 | 1.013 | 1.123 |
| Q3 | 1.089 | 0.029 | 3.22 | 0.001 | 1.034 | 1.147 |
| Q4 | 1.171 | 0.031 | 5.91 | 0.000 | 1.111 | 1.234 |
| Q5-most deprived | 1.273 | 0.035 | 8.89 | 0.000 | 1.207 | 1.342 |
|  |  |  |  |  |  |  |
| Other than White | 0.975 | 0.035 | -0.70 | 0.484 | 0.910 | 1.046 |
| Drinker | 0.929 | 0.021 | -3.27 | 0.001 | 0.889 | 0.971 |
|  |  |  |  |  |  |  |
| Smoking_status |  |  |  |  |  |  |
| current smoker | 1.118 | 0.028 | 4.38 | 0.000 | 1.063 | 1.175 |
| ex-smoker | 0.934 | 0.021 | -3.00 | 0.003 | 0.893 | 0.976 |
|  |  |  |  |  |  |  |
| BMI | 0.994 | 0.002 | -3.78 | 0.000 | 0.991 | 0.997 |
| HT_b | 1.049 | 0.019 | 2.66 | 0.008 | 1.013 | 1.086 |
| Hyperlipidaemia_b | 0.952 | 0.020 | -2.29 | 0.022 | 0.913 | 0.993 |
|  |  |  |  |  |  |  |
| Diabetes_type_b |  |  |  |  |  |  |
| T1DM | 2.221 | 0.201 | 8.80 | 0.000 | 1.860 | 2.654 |
| T2DM | 1.373 | 0.032 | 13.61 | 0.000 | 1.312 | 1.437 |
| DM - nos | 1.894 | 0.132 | 9.15 | 0.000 | 1.652 | 2.171 |
|  |  |  |  |  |  |  |
| HF_b | 3.349 | 0.120 | 33.66 | 0.000 | 3.121 | 3.593 |
| AF_b | 1.376 | 0.039 | 11.22 | 0.000 | 1.302 | 1.455 |
| Heart_valve_dis_b | 1.394 | 0.106 | 4.38 | 0.000 | 1.201 | 1.617 |
| VT_VF_b | 0.961 | 0.128 | -0.29 | 0.768 | 0.741 | 1.248 |
| Cardiomyopathy_b | 1.602 | 0.185 | 4.07 | 0.000 | 1.277 | 2.010 |
| CV_procedures_b | 0.719 | 0.021 | -11.21 | 0.000 | 0.678 | 0.761 |
| TIA_stroke_b | 2.040 | 0.054 | 26.81 | 0.000 | 1.937 | 2.150 |
| PVD_b | 1.574 | 0.052 | 13.64 | 0.000 | 1.475 | 1.680 |
| CKD_b | 1.246 | 0.027 | 10.00 | 0.000 | 1.193 | 1.301 |
| Hypothyroidism_b | 1.041 | 0.032 | 1.32 | 0.188 | 0.980 | 1.106 |
| Liver_dis_b | 1.516 | 0.275 | 2.29 | 0.022 | 1.062 | 2.163 |
| Lupus_b | 1.372 | 0.195 | 2.22 | 0.027 | 1.037 | 1.813 |
| Erectile_dysfunction_b | 0.963 | 0.029 | -1.25 | 0.212 | 0.907 | 1.022 |
| Any_tumour_b | 1.191 | 0.027 | 7.75 | 0.000 | 1.140 | 1.245 |
| Menopause_b | 0.798 | 0.026 | -6.95 | 0.000 | 0.748 | 0.850 |
| Dementia_b | 1.383 | 0.074 | 6.06 | 0.000 | 1.245 | 1.536 |
| RA_b | 1.075 | 0.049 | 1.58 | 0.114 | 0.983 | 1.175 |
| _cons | 0.030 | 0.003 | -40.27 | 0.000 | 0.026 | 0.036 |

**Performance in validation dataset (20% of practices) - Internal validation - Index AMI cases**

| AUROC (95% CI): 0.707 (0.699; 0.715) |  |
| --- | --- |
| 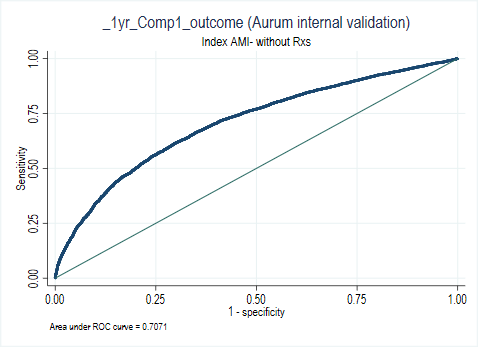 | 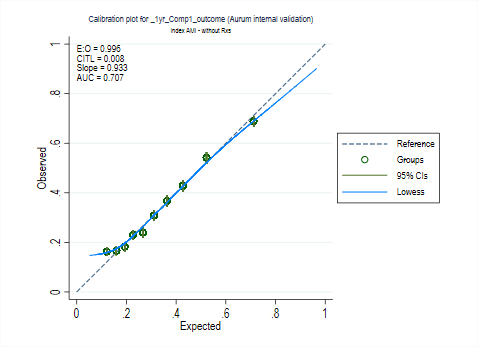 |
| 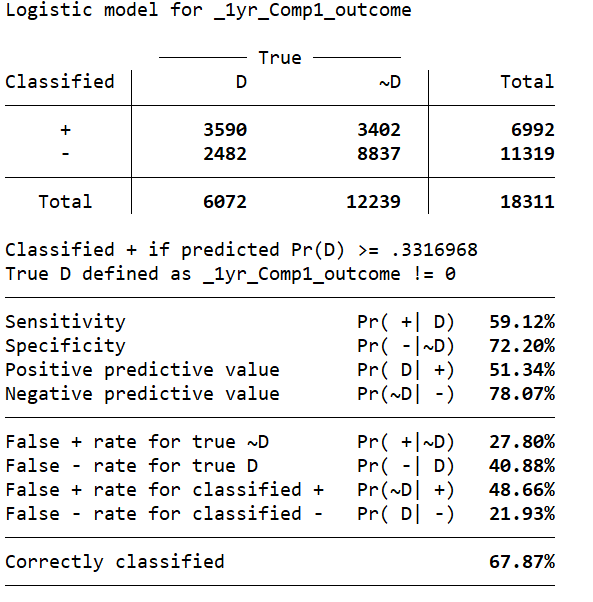 | % Correctly classified: 67.87 |

### 7) 1-year Composite 1 CV outcome (HF, stroke, recurrent MI, & CV mortality)

| _1yr_Comp1_CV_outcome - without therapies | | | | | | |
| --- | --- | --- | --- | --- | --- | --- |
| _1yr_Comp1_CV_outcome | Odds ratio | Std. err. | z | P>|z| | [95% conf. interval] | |
| age | 1.033 | 0.001 | 40.38 | 0.000 | 1.032 | 1.035 |
|  |  |  |  |  |  |  |
| gender |  |  |  |  |  |  |
| Female | 1.023 | 0.021 | 1.09 | 0.275 | 0.982 | 1.065 |
|  |  |  |  |  |  |  |
| IMD |  |  |  |  |  |  |
| Q2 | 1.031 | 0.027 | 1.16 | 0.248 | 0.979 | 1.087 |
| Q3 | 1.058 | 0.028 | 2.12 | 0.034 | 1.004 | 1.115 |
| Q4 | 1.153 | 0.031 | 5.27 | 0.000 | 1.093 | 1.215 |
| Q5-most deprived | 1.231 | 0.034 | 7.60 | 0.000 | 1.167 | 1.299 |
|  |  |  |  |  |  |  |
| Other than White | 0.990 | 0.035 | -0.28 | 0.779 | 0.923 | 1.062 |
| Drinker | 0.918 | 0.021 | -3.77 | 0.000 | 0.879 | 0.960 |
|  |  |  |  |  |  |  |
| Smoking_status |  |  |  |  |  |  |
| current smoker | 1.095 | 0.028 | 3.53 | 0.000 | 1.041 | 1.151 |
| ex-smoker | 0.946 | 0.022 | -2.42 | 0.015 | 0.904 | 0.989 |
|  |  |  |  |  |  |  |
| BMI | 0.999 | 0.002 | -0.31 | 0.753 | 0.996 | 1.003 |
| HT_b | 1.053 | 0.019 | 2.87 | 0.004 | 1.017 | 1.091 |
| Hyperlipidaemia_b | 0.972 | 0.021 | -1.33 | 0.182 | 0.931 | 1.014 |
|  |  |  |  |  |  |  |
| Diabetes_type_b |  |  |  |  |  |  |
| T1DM | 2.172 | 0.197 | 8.56 | 0.000 | 1.818 | 2.594 |
| T2DM | 1.347 | 0.031 | 12.74 | 0.000 | 1.287 | 1.410 |
| DM - nos | 1.838 | 0.128 | 8.77 | 0.000 | 1.605 | 2.106 |
|  |  |  |  |  |  |  |
| HF_b | 3.391 | 0.119 | 34.86 | 0.000 | 3.166 | 3.632 |
| AF_b | 1.375 | 0.039 | 11.23 | 0.000 | 1.301 | 1.454 |
| Heart_valve_dis_b | 1.368 | 0.103 | 4.17 | 0.000 | 1.181 | 1.584 |
| VT_VF_b | 1.162 | 0.153 | 1.14 | 0.255 | 0.898 | 1.504 |
| Cardiomyopathy_b | 1.682 | 0.193 | 4.53 | 0.000 | 1.343 | 2.107 |
| CV_procedures_b | 0.736 | 0.022 | -10.31 | 0.000 | 0.694 | 0.780 |
| TIA_stroke_b | 2.105 | 0.056 | 28.23 | 0.000 | 1.999 | 2.217 |
| PVD_b | 1.542 | 0.051 | 13.05 | 0.000 | 1.445 | 1.646 |
| CKD_b | 1.200 | 0.027 | 8.23 | 0.000 | 1.149 | 1.253 |
| Hypothyroidism_b | 1.010 | 0.031 | 0.33 | 0.742 | 0.951 | 1.073 |
| Liver_dis_b | 1.495 | 0.273 | 2.20 | 0.028 | 1.045 | 2.139 |
| Lupus_b | 1.277 | 0.185 | 1.69 | 0.091 | 0.962 | 1.696 |
| Erectile_dysfunction_b | 1.014 | 0.031 | 0.47 | 0.639 | 0.956 | 1.077 |
| Any_tumour_b | 1.044 | 0.024 | 1.87 | 0.062 | 0.998 | 1.092 |
| Menopause_b | 0.792 | 0.026 | -7.07 | 0.000 | 0.742 | 0.845 |
| Dementia_b | 1.110 | 0.059 | 1.96 | 0.050 | 1.000 | 1.231 |
| RA_b | 1.072 | 0.049 | 1.52 | 0.128 | 0.980 | 1.174 |
| _cons | 0.031 | 0.003 | -39.83 | 0.000 | 0.026 | 0.036 |

**Performance in validation dataset (20% of practices) - Internal validation - Index AMI cases**

| AUROC (95% CI): 0.697 (0.689; 0.706) |  |
| --- | --- |
| 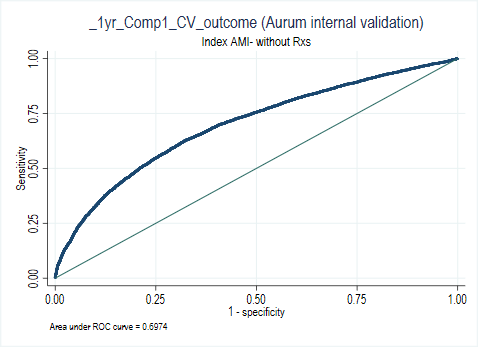 | 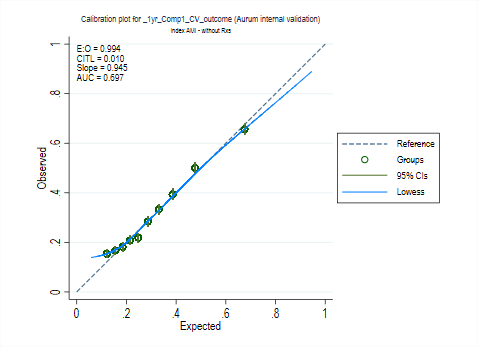 |
| 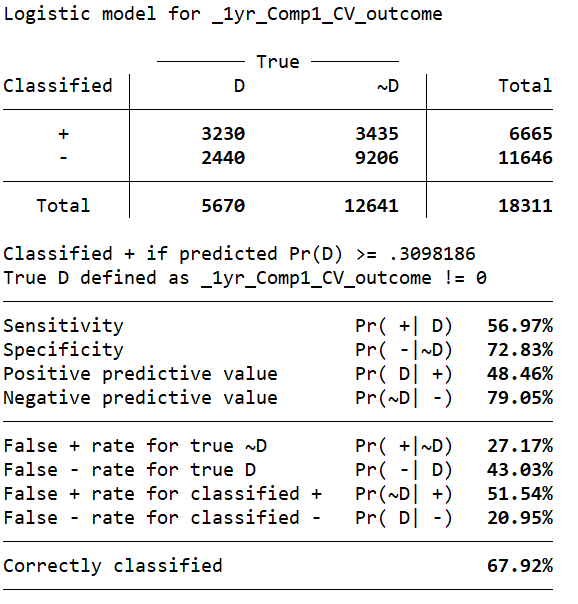 | % Correctly classified: 67.92 |

### 8) 1-year Composite 2 outcome (HF, stroke, & all-cause mortality)

|  | | | | | | |
| --- | --- | --- | --- | --- | --- | --- |
| _1yr_Comp2_outcome | Odds ratio | Std. err. | z | P>|z| | [95% conf. interval] | |
| age | 1.052 | 0.001 | 54.42 | 0.000 | 1.050 | 1.054 |
|  |  |  |  |  |  |  |
| gender |  |  |  |  |  |  |
| Female | 1.013 | 0.022 | 0.58 | 0.564 | 0.970 | 1.058 |
|  |  |  |  |  |  |  |
| IMD |  |  |  |  |  |  |
| Q2 | 1.075 | 0.031 | 2.47 | 0.014 | 1.015 | 1.138 |
| Q3 | 1.144 | 0.033 | 4.60 | 0.000 | 1.080 | 1.212 |
| Q4 | 1.231 | 0.036 | 7.04 | 0.000 | 1.162 | 1.304 |
| Q5-most deprived | 1.394 | 0.042 | 11.11 | 0.000 | 1.314 | 1.478 |
|  |  |  |  |  |  |  |
| Other than White | 0.977 | 0.039 | -0.59 | 0.554 | 0.903 | 1.056 |
| Drinker | 0.869 | 0.021 | -5.82 | 0.000 | 0.829 | 0.911 |
|  |  |  |  |  |  |  |
| Smoking_status |  |  |  |  |  |  |
| current smoker | 1.194 | 0.033 | 6.34 | 0.000 | 1.131 | 1.262 |
| ex-smoker | 0.958 | 0.024 | -1.72 | 0.085 | 0.912 | 1.006 |
|  |  |  |  |  |  |  |
| BMI | 0.994 | 0.002 | -3.43 | 0.001 | 0.990 | 0.997 |
| HT_b | 1.066 | 0.021 | 3.31 | 0.001 | 1.027 | 1.108 |
| Hyperlipidaemia_b | 0.968 | 0.023 | -1.39 | 0.165 | 0.925 | 1.013 |
|  |  |  |  |  |  |  |
| Diabetes_type_b |  |  |  |  |  |  |
| T1DM | 2.680 | 0.261 | 10.14 | 0.000 | 2.215 | 3.243 |
| T2DM | 1.422 | 0.035 | 14.12 | 0.000 | 1.354 | 1.493 |
| DM - nos | 2.066 | 0.151 | 9.95 | 0.000 | 1.791 | 2.384 |
|  |  |  |  |  |  |  |
| HF_b | 3.917 | 0.141 | 37.94 | 0.000 | 3.651 | 4.204 |
| AF_b | 1.474 | 0.043 | 13.26 | 0.000 | 1.392 | 1.561 |
| Heart_valve_dis_b | 1.303 | 0.102 | 3.40 | 0.001 | 1.119 | 1.518 |
| VT_VF_b | 1.080 | 0.152 | 0.55 | 0.583 | 0.820 | 1.422 |
| Cardiomyopathy_b | 1.794 | 0.214 | 4.91 | 0.000 | 1.421 | 2.266 |
| CV_procedures_b | 0.793 | 0.025 | -7.28 | 0.000 | 0.745 | 0.844 |
| TIA_stroke_b | 2.319 | 0.063 | 30.95 | 0.000 | 2.199 | 2.446 |
| PVD_b | 1.651 | 0.057 | 14.56 | 0.000 | 1.543 | 1.766 |
| CKD_b | 1.243 | 0.029 | 9.47 | 0.000 | 1.188 | 1.301 |
| Hypothyroidism_b | 1.001 | 0.033 | 0.03 | 0.974 | 0.939 | 1.067 |
| Liver_dis_b | 1.006 | 0.225 | 0.03 | 0.980 | 0.649 | 1.559 |
| Lupus_b | 1.384 | 0.215 | 2.09 | 0.036 | 1.021 | 1.876 |
| Erectile_dysfunction_b | 0.979 | 0.033 | -0.62 | 0.534 | 0.916 | 1.046 |
| Any_tumour_b | 1.252 | 0.030 | 9.41 | 0.000 | 1.195 | 1.312 |
| Menopause_b | 0.782 | 0.028 | -6.85 | 0.000 | 0.729 | 0.839 |
| Dementia_b | 1.426 | 0.077 | 6.54 | 0.000 | 1.282 | 1.585 |
| RA_b | 1.174 | 0.057 | 3.30 | 0.001 | 1.067 | 1.291 |
| _cons | 0.007 | 0.001 | -50.75 | 0.000 | 0.005 | 0.008 |

**Performance in validation dataset (20% of practices) - Internal validation - Index AMI cases**

| AUROC (95% CI): 0.760 (0.752; 0.768) |  |
| --- | --- |
| 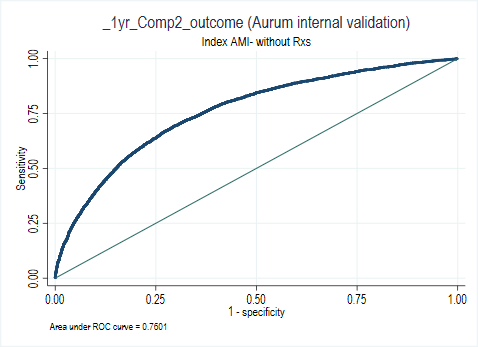 | 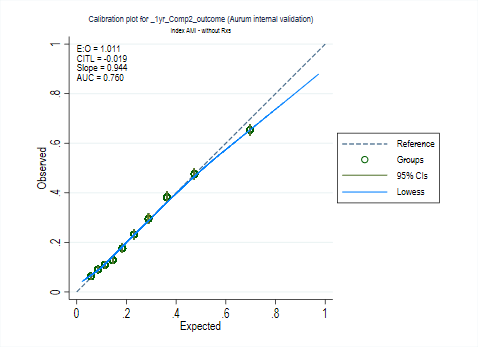 |
| 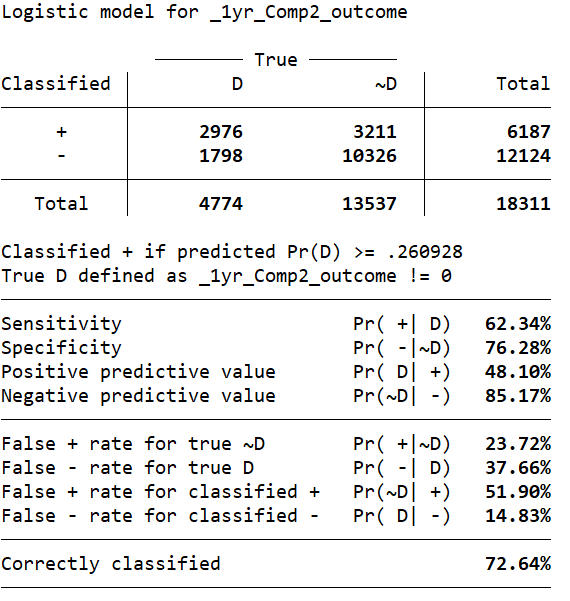 | % Correctly classified: 72.64 |

### 9) 1-year Composite 2 CV outcome (HF, stroke, & CV mortality)

| _1yr_Comp2_CV_outcome - without therapies | | | | | | |
| --- | --- | --- | --- | --- | --- | --- |
| _1yr_Comp2_CV_outcome | Odds ratio | Std. err. | z | P>|z| | [95% conf. interval] | |
| age | 1.047 | 0.001 | 48.02 | 0.000 | 1.045 | 1.049 |
|  |  |  |  |  |  |  |
| gender |  |  |  |  |  |  |
| Female | 0.974 | 0.022 | -1.15 | 0.250 | 0.931 | 1.019 |
|  |  |  |  |  |  |  |
| IMD |  |  |  |  |  |  |
| Q2 | 1.057 | 0.032 | 1.85 | 0.064 | 0.997 | 1.122 |
| Q3 | 1.124 | 0.034 | 3.88 | 0.000 | 1.060 | 1.193 |
| Q4 | 1.242 | 0.038 | 7.14 | 0.000 | 1.170 | 1.318 |
| Q5-most deprived | 1.397 | 0.043 | 10.89 | 0.000 | 1.316 | 1.484 |
|  |  |  |  |  |  |  |
| Other than White | 0.947 | 0.039 | -1.31 | 0.189 | 0.874 | 1.027 |
| Drinker | 0.890 | 0.022 | -4.70 | 0.000 | 0.848 | 0.935 |
|  |  |  |  |  |  |  |
| Smoking_status |  |  |  |  |  |  |
| current smoker | 1.120 | 0.032 | 3.95 | 0.000 | 1.059 | 1.185 |
| ex-smoker | 0.926 | 0.024 | -3.00 | 0.003 | 0.881 | 0.974 |
|  |  |  |  |  |  |  |
| BMI | 1.000 | 0.002 | -0.19 | 0.846 | 0.996 | 1.003 |
| HT_b | 1.103 | 0.022 | 4.93 | 0.000 | 1.061 | 1.147 |
| Hyperlipidaemia_b | 0.987 | 0.024 | -0.55 | 0.579 | 0.942 | 1.034 |
|  |  |  |  |  |  |  |
| Diabetes_type_b |  |  |  |  |  |  |
| T1DM | 2.580 | 0.254 | 9.63 | 0.000 | 2.127 | 3.128 |
| T2DM | 1.436 | 0.036 | 14.28 | 0.000 | 1.366 | 1.509 |
| DM - nos | 2.075 | 0.152 | 9.97 | 0.000 | 1.798 | 2.395 |
|  |  |  |  |  |  |  |
| HF_b | 4.137 | 0.146 | 40.30 | 0.000 | 3.861 | 4.433 |
| AF_b | 1.457 | 0.043 | 12.75 | 0.000 | 1.375 | 1.543 |
| Heart_valve_dis_b | 1.408 | 0.110 | 4.40 | 0.000 | 1.209 | 1.640 |
| VT_VF_b | 1.083 | 0.154 | 0.56 | 0.576 | 0.820 | 1.430 |
| Cardiomyopathy_b | 1.838 | 0.218 | 5.13 | 0.000 | 1.457 | 2.319 |
| CV_procedures_b | 0.809 | 0.026 | -6.51 | 0.000 | 0.759 | 0.862 |
| TIA_stroke_b | 2.516 | 0.068 | 34.03 | 0.000 | 2.386 | 2.654 |
| PVD_b | 1.625 | 0.056 | 13.98 | 0.000 | 1.518 | 1.740 |
| CKD_b | 1.274 | 0.030 | 10.35 | 0.000 | 1.217 | 1.334 |
| Hypothyroidism_b | 0.973 | 0.033 | -0.81 | 0.420 | 0.911 | 1.040 |
| Liver_dis_b | 1.100 | 0.247 | 0.42 | 0.672 | 0.708 | 1.708 |
| Lupus_b | 1.476 | 0.233 | 2.47 | 0.013 | 1.084 | 2.010 |
| Erectile_dysfunction_b | 0.970 | 0.034 | -0.86 | 0.387 | 0.906 | 1.039 |
| Any_tumour_b | 1.061 | 0.026 | 2.40 | 0.017 | 1.011 | 1.114 |
| Menopause_b | 0.792 | 0.029 | -6.28 | 0.000 | 0.736 | 0.851 |
| Dementia_b | 1.081 | 0.060 | 1.42 | 0.156 | 0.970 | 1.205 |
| RA_b | 1.115 | 0.056 | 2.16 | 0.031 | 1.010 | 1.230 |
| _cons | 0.007 | 0.001 | -48.86 | 0.000 | 0.006 | 0.008 |

**Performance in validation dataset (20% of practices) - Internal validation - Index AMI cases**

| AUROC (95% CI): 0.756 (0.748; 0.765) |  |
| --- | --- |
| 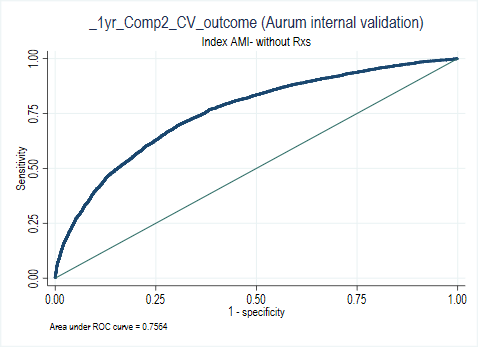 | 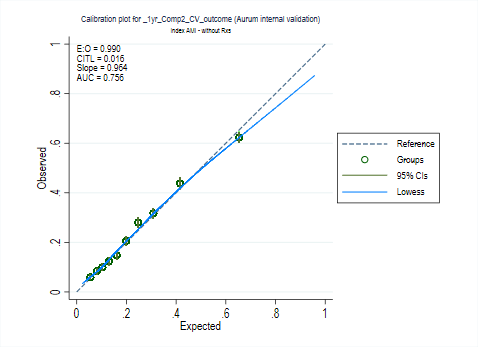 |
| 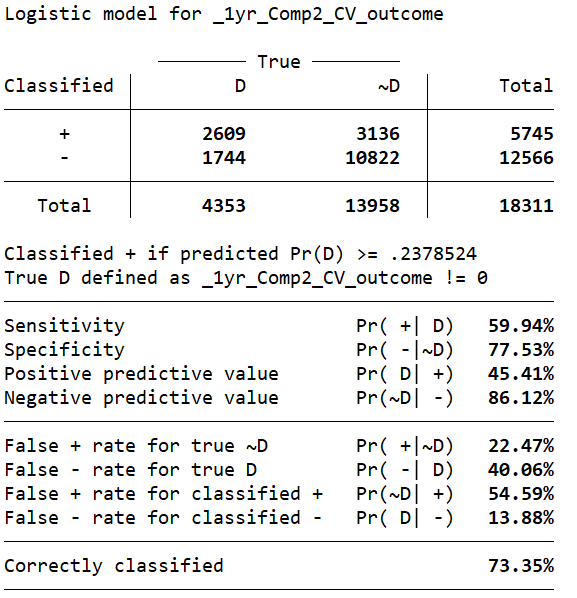 | % Correctly classified: 73.35 |

## External validation (CPRD GOLD) - without therapies

### 1) 1-year all-cause mortality

|  | | | | | | |
| --- | --- | --- | --- | --- | --- | --- |
| _1year_All_cause_death | Odds ratio | Std. err. | z | P>|z| | [95% conf. interval] | |
| age | 1.077 | 0.002 | 51.96 | 0.000 | 1.074 | 1.080 |
|  |  |  |  |  |  |  |
| gender |  |  |  |  |  |  |
| Female | 0.974 | 0.029 | -0.89 | 0.373 | 0.920 | 1.032 |
|  |  |  |  |  |  |  |
| IMD |  |  |  |  |  |  |
| Q2 | 1.064 | 0.043 | 1.55 | 0.120 | 0.984 | 1.151 |
| Q3 | 1.162 | 0.047 | 3.76 | 0.000 | 1.075 | 1.257 |
| Q4 | 1.206 | 0.049 | 4.62 | 0.000 | 1.114 | 1.305 |
| Q5-most deprived | 1.284 | 0.053 | 6.06 | 0.000 | 1.184 | 1.392 |
| unknown | 0.538 | 0.301 | -1.11 | 0.268 | 0.180 | 1.611 |
|  |  |  |  |  |  |  |
| Other than White | 1.454 | 0.070 | 7.82 | 0.000 | 1.324 | 1.597 |
| Drinker | 0.996 | 0.013 | -0.33 | 0.740 | 0.971 | 1.021 |
|  |  |  |  |  |  |  |
| Smoking_status |  |  |  |  |  |  |
| current smoker | 1.261 | 0.048 | 6.12 | 0.000 | 1.171 | 1.359 |
| ex-smoker | 0.880 | 0.029 | -3.86 | 0.000 | 0.825 | 0.939 |
| unknown | 2.330 | 0.356 | 5.53 | 0.000 | 1.727 | 3.144 |
|  |  |  |  |  |  |  |
| BMI | 0.973 | 0.003 | -10.25 | 0.000 | 0.968 | 0.978 |
| HT_b | 0.981 | 0.026 | -0.74 | 0.459 | 0.931 | 1.033 |
| Hyperlipidaemia_b | 0.893 | 0.029 | -3.48 | 0.000 | 0.838 | 0.952 |
|  |  |  |  |  |  |  |
| Diabetes_type_b |  |  |  |  |  |  |
| T1DM | 3.264 | 0.416 | 9.27 | 0.000 | 2.542 | 4.191 |
| T2DM | 1.400 | 0.046 | 10.14 | 0.000 | 1.312 | 1.494 |
| DM - nos | 2.136 | 0.194 | 8.37 | 0.000 | 1.788 | 2.552 |
|  |  |  |  |  |  |  |
| HF_b | 1.889 | 0.074 | 16.33 | 0.000 | 1.750 | 2.038 |
| AF_b | 1.328 | 0.047 | 8.11 | 0.000 | 1.240 | 1.423 |
| Heart_valve_dis_b | 1.442 | 0.126 | 4.19 | 0.000 | 1.215 | 1.712 |
| VT_VF_b | 0.832 | 0.157 | -0.98 | 0.328 | 0.575 | 1.203 |
| Cardiomyopathy_b | 1.210 | 0.190 | 1.21 | 0.225 | 0.890 | 1.644 |
| CV_procedures_b | 0.812 | 0.037 | -4.63 | 0.000 | 0.743 | 0.887 |
| TIA_stroke_b | 1.343 | 0.045 | 8.74 | 0.000 | 1.257 | 1.435 |
| PVD_b | 1.689 | 0.070 | 12.59 | 0.000 | 1.557 | 1.833 |
| CKD_b | 1.319 | 0.038 | 9.59 | 0.000 | 1.246 | 1.395 |
| Hypothyroidism_b | 1.032 | 0.043 | 0.75 | 0.450 | 0.951 | 1.120 |
| Liver_dis_b | 1.837 | 0.530 | 2.11 | 0.035 | 1.043 | 3.234 |
| Lupus_b | 1.327 | 0.285 | 1.32 | 0.188 | 0.871 | 2.020 |
| Erectile_dysfunction_b | 0.811 | 0.043 | -3.95 | 0.000 | 0.731 | 0.900 |
| Any_tumour_b | 1.543 | 0.046 | 14.70 | 0.000 | 1.457 | 1.635 |
| Menopause_b | 0.824 | 0.042 | -3.81 | 0.000 | 0.746 | 0.910 |
| Dementia_b | 1.803 | 0.103 | 10.30 | 0.000 | 1.612 | 2.017 |
| RA_b | 1.355 | 0.085 | 4.85 | 0.000 | 1.199 | 1.533 |
| _cons | 0.001 | 0.000 | -50.65 | 0.000 | 0.000 | 0.001 |

**Performance in validation dataset (CPRD GOLD) - External validation - Index AMI cases**

| AUROC (95% CI): 0.793 (0.782; 0.803) |  |
| --- | --- |
| 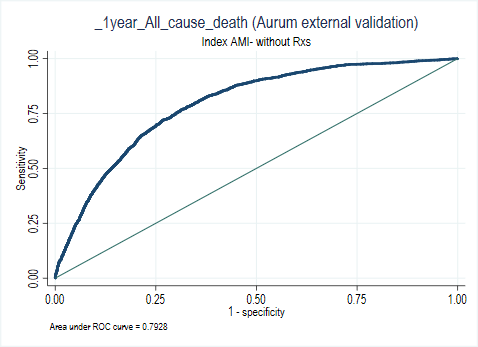 | 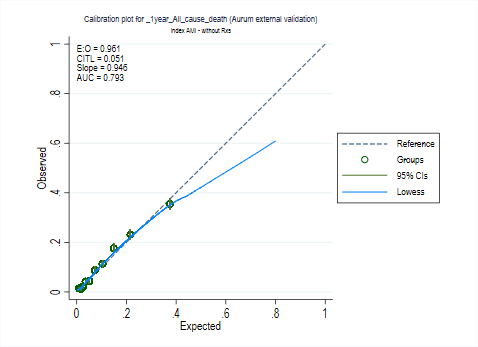 |
| 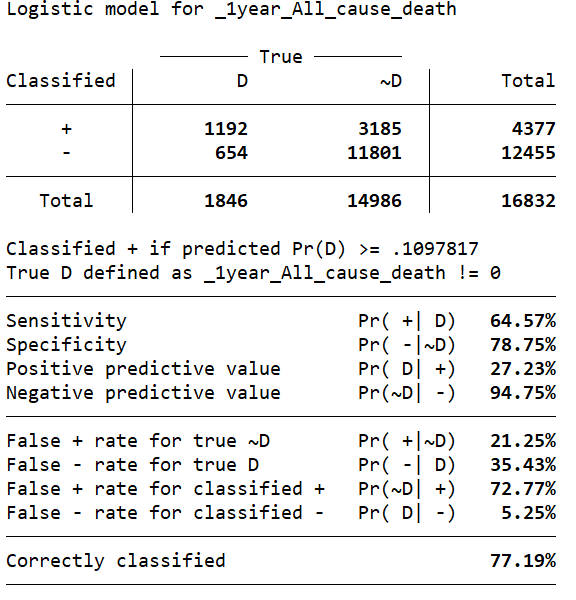 | % Correctly classified: 77.19 |

### 2) 1-year Stroke

| _1year_Stroke | Odds ratio | Std. err. | z | P>|z| | [95% conf. interval] | |
| --- | --- | --- | --- | --- | --- | --- |
| age | 1.040 | 0.002 | 23.61 | 0.000 | 1.037 | 1.043 |
|  |  |  |  |  |  |  |
| gender |  |  |  |  |  |  |
| Female | 1.119 | 0.042 | 3.02 | 0.003 | 1.040 | 1.203 |
|  |  |  |  |  |  |  |
| IMD |  |  |  |  |  |  |
| Q2 | 0.929 | 0.046 | -1.49 | 0.136 | 0.843 | 1.023 |
| Q3 | 1.009 | 0.050 | 0.17 | 0.863 | 0.915 | 1.111 |
| Q4 | 1.097 | 0.054 | 1.87 | 0.062 | 0.995 | 1.208 |
| Q5-most deprived | 1.125 | 0.057 | 2.35 | 0.019 | 1.020 | 1.242 |
|  |  |  |  |  |  |  |
| Other than White | 0.958 | 0.067 | -0.62 | 0.536 | 0.835 | 1.098 |
| Drinker | 0.856 | 0.033 | -4.01 | 0.000 | 0.793 | 0.923 |
|  |  |  |  |  |  |  |
| Smoking_status |  |  |  |  |  |  |
| current smoker | 1.104 | 0.052 | 2.10 | 0.036 | 1.007 | 1.211 |
| ex-smoker | 0.927 | 0.038 | -1.84 | 0.066 | 0.855 | 1.005 |
|  |  |  |  |  |  |  |
| BMI | 0.989 | 0.003 | -3.68 | 0.000 | 0.983 | 0.995 |
| HT_b | 1.202 | 0.039 | 5.63 | 0.000 | 1.127 | 1.281 |
| Hyperlipidaemia_b | 1.127 | 0.042 | 3.25 | 0.001 | 1.049 | 1.212 |
|  |  |  |  |  |  |  |
| Diabetes_type_b |  |  |  |  |  |  |
| T1DM | 2.927 | 0.411 | 7.65 | 0.000 | 2.223 | 3.854 |
| T2DM | 1.399 | 0.055 | 8.51 | 0.000 | 1.295 | 1.511 |
| DM - nos | 1.579 | 0.172 | 4.19 | 0.000 | 1.275 | 1.955 |
|  |  |  |  |  |  |  |
| HF_b | 1.247 | 0.062 | 4.42 | 0.000 | 1.131 | 1.375 |
| AF_b | 1.455 | 0.061 | 8.98 | 0.000 | 1.341 | 1.580 |
| Heart_valve_dis_b | 1.221 | 0.129 | 1.88 | 0.060 | 0.992 | 1.502 |
| VT_VF_b | 1.086 | 0.223 | 0.40 | 0.689 | 0.725 | 1.625 |
| Cardiomyopathy_b | 1.702 | 0.292 | 3.10 | 0.002 | 1.216 | 2.382 |
| CV_procedures_b | 0.857 | 0.045 | -2.95 | 0.003 | 0.773 | 0.949 |
| TIA_stroke_b | 6.857 | 0.227 | 58.08 | 0.000 | 6.426 | 7.317 |
| PVD_b | 1.488 | 0.074 | 7.97 | 0.000 | 1.349 | 1.640 |
| CKD_b | 1.072 | 0.039 | 1.92 | 0.054 | 0.999 | 1.150 |
| Hypothyroidism_b | 1.023 | 0.053 | 0.45 | 0.653 | 0.925 | 1.132 |
| Liver_dis_b | 0.867 | 0.361 | -0.34 | 0.731 | 0.383 | 1.962 |
| Lupus_b | 1.096 | 0.282 | 0.36 | 0.722 | 0.662 | 1.816 |
| Erectile_dysfunction_b | 1.057 | 0.062 | 0.95 | 0.341 | 0.943 | 1.186 |
| Any_tumour_b | 1.057 | 0.041 | 1.41 | 0.159 | 0.979 | 1.141 |
| Menopause_b | 0.904 | 0.053 | -1.70 | 0.089 | 0.805 | 1.015 |
| Dementia_b | 1.265 | 0.094 | 3.16 | 0.002 | 1.093 | 1.463 |
| RA_b | 1.000 | 0.082 | -0.00 | 0.999 | 0.851 | 1.175 |
| _cons | 0.003 | 0.000 | -33.60 | 0.000 | 0.002 | 0.004 |

**Performance in validation dataset (CPRD GOLD) - External validation - Index AMI cases**

| AUROC (95% CI): 0.776 (0.761; 0.791) |  |
| --- | --- |
| 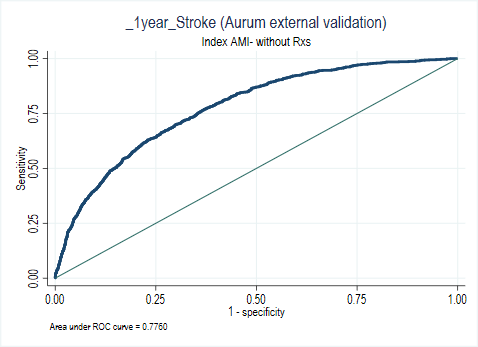 | 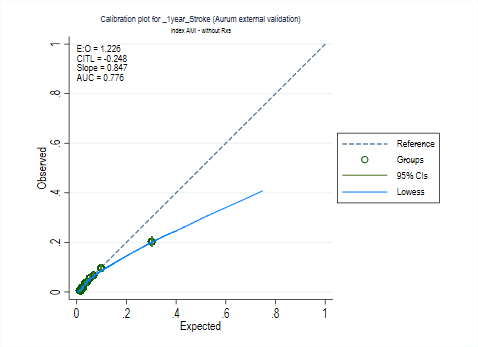 |
| 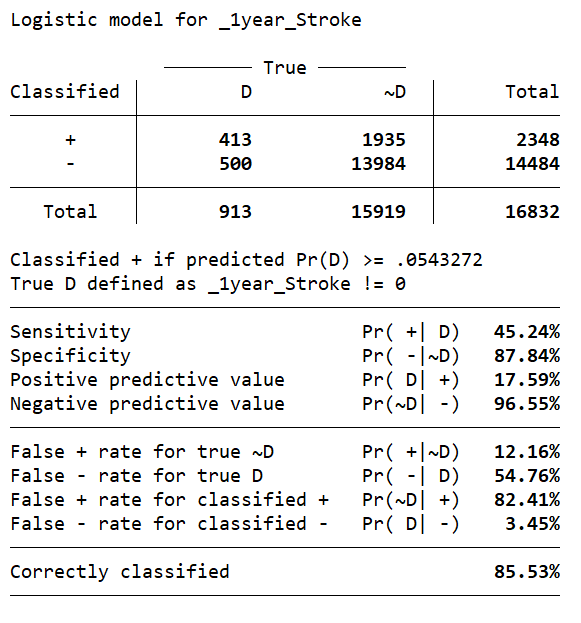 | % Correctly classified: 85.53 |

### 3) 1-year heart failure (HF)

|  | | | | | | |
| --- | --- | --- | --- | --- | --- | --- |
| _1year_HF | Odds ratio | Std. err. | z | P>|z| | [95% conf. interval] | |
| age | 1.041 | 0.001 | 37.98 | 0.000 | 1.039 | 1.043 |
|  |  |  |  |  |  |  |
| gender |  |  |  |  |  |  |
| Female | 0.946 | 0.024 | -2.16 | 0.031 | 0.900 | 0.995 |
|  |  |  |  |  |  |  |
| IMD |  |  |  |  |  |  |
| Q2 | 1.107 | 0.037 | 3.03 | 0.002 | 1.037 | 1.182 |
| Q3 | 1.091 | 0.037 | 2.55 | 0.011 | 1.020 | 1.165 |
| Q4 | 1.183 | 0.040 | 4.95 | 0.000 | 1.107 | 1.265 |
| Q5-most deprived | 1.350 | 0.046 | 8.78 | 0.000 | 1.263 | 1.444 |
|  |  |  |  |  |  |  |
| Other than White | 1.045 | 0.047 | 0.98 | 0.327 | 0.957 | 1.141 |
| Drinker | 0.837 | 0.023 | -6.58 | 0.000 | 0.794 | 0.882 |
|  |  |  |  |  |  |  |
| Smoking_status |  |  |  |  |  |  |
| current smoker | 1.160 | 0.037 | 4.60 | 0.000 | 1.089 | 1.236 |
| ex-smoker | 1.016 | 0.029 | 0.57 | 0.569 | 0.961 | 1.075 |
|  |  |  |  |  |  |  |
| BMI | 1.005 | 0.002 | 2.63 | 0.009 | 1.001 | 1.009 |
| HT_b | 1.098 | 0.024 | 4.19 | 0.000 | 1.051 | 1.146 |
| Hyperlipidaemia_b | 0.938 | 0.025 | -2.40 | 0.016 | 0.890 | 0.988 |
|  |  |  |  |  |  |  |
| Diabetes_type_b |  |  |  |  |  |  |
| T1DM | 2.332 | 0.249 | 7.95 | 0.000 | 1.893 | 2.874 |
| T2DM | 1.423 | 0.039 | 12.76 | 0.000 | 1.348 | 1.502 |
| DM - nos | 2.091 | 0.160 | 9.63 | 0.000 | 1.799 | 2.429 |
|  |  |  |  |  |  |  |
| HF_b | 5.248 | 0.178 | 48.82 | 0.000 | 4.910 | 5.610 |
| AF_b | 1.420 | 0.045 | 11.15 | 0.000 | 1.335 | 1.510 |
| Heart_valve_dis_b | 1.381 | 0.112 | 3.99 | 0.000 | 1.178 | 1.617 |
| VT_VF_b | 1.102 | 0.166 | 0.64 | 0.520 | 0.820 | 1.479 |
| Cardiomyopathy_b | 1.815 | 0.217 | 4.98 | 0.000 | 1.436 | 2.295 |
| CV_procedures_b | 0.830 | 0.030 | -5.15 | 0.000 | 0.773 | 0.891 |
| TIA_stroke_b | 1.089 | 0.034 | 2.73 | 0.006 | 1.024 | 1.157 |
| PVD_b | 1.473 | 0.055 | 10.33 | 0.000 | 1.369 | 1.586 |
| CKD_b | 1.256 | 0.032 | 8.90 | 0.000 | 1.194 | 1.320 |
| Hypothyroidism_b | 1.022 | 0.038 | 0.59 | 0.557 | 0.951 | 1.098 |
| Liver_dis_b | 1.235 | 0.301 | 0.86 | 0.388 | 0.765 | 1.992 |
| Lupus_b | 1.568 | 0.267 | 2.65 | 0.008 | 1.124 | 2.189 |
| Erectile_dysfunction_b | 0.982 | 0.038 | -0.46 | 0.648 | 0.911 | 1.060 |
| Any_tumour_b | 1.116 | 0.030 | 4.03 | 0.000 | 1.058 | 1.177 |
| Menopause_b | 0.801 | 0.034 | -5.25 | 0.000 | 0.738 | 0.870 |
| Dementia_b | 0.833 | 0.052 | -2.91 | 0.004 | 0.737 | 0.942 |
| RA_b | 1.106 | 0.062 | 1.82 | 0.069 | 0.992 | 1.234 |
| _cons | 0.006 | 0.001 | -45.20 | 0.000 | 0.005 | 0.008 |

**Performance in validation dataset (CPRD GOLD) - External validation - Index AMI cases**

| AUROC (95% CI): 0.705 (0.694; 0.715) |  |
| --- | --- |
| 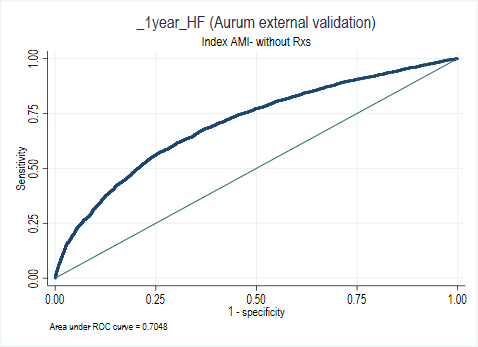 | 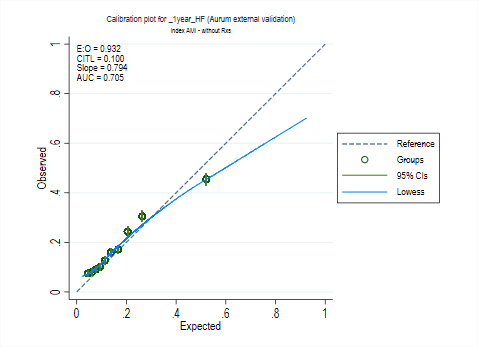 |
| 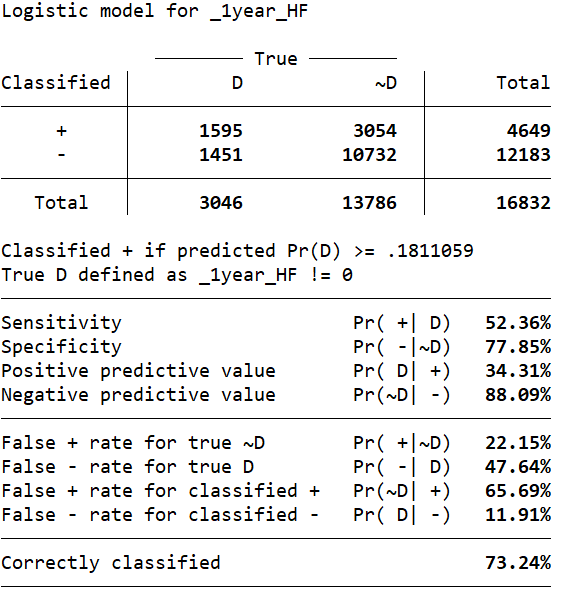 | % Correctly classified: 73.24 |

### 4) 1-year Recurrent MI

| _1year_Recurrent_MI | Odds ratio | Std. err. | z | P>|z| | [95% conf. interval] | |
| --- | --- | --- | --- | --- | --- | --- |
| age | 1.012 | 0.001 | 11.27 | 0.000 | 1.010 | 1.014 |
|  |  |  |  |  |  |  |
| gender |  |  |  |  |  |  |
| Female | 1.013 | 0.028 | 0.46 | 0.642 | 0.959 | 1.070 |
|  |  |  |  |  |  |  |
| IMD |  |  |  |  |  |  |
| Q2 | 1.039 | 0.037 | 1.06 | 0.290 | 0.968 | 1.114 |
| Q3 | 1.003 | 0.036 | 0.07 | 0.942 | 0.934 | 1.077 |
| Q4 | 1.002 | 0.037 | 0.06 | 0.952 | 0.933 | 1.077 |
| Q5-most deprived | 1.062 | 0.039 | 1.62 | 0.106 | 0.987 | 1.141 |
|  |  |  |  |  |  |  |
| Other than White | 1.076 | 0.051 | 1.56 | 0.120 | 0.981 | 1.180 |
| Drinker | 0.971 | 0.030 | -0.97 | 0.330 | 0.914 | 1.031 |
|  |  |  |  |  |  |  |
| Smoking_status |  |  |  |  |  |  |
| current smoker | 1.048 | 0.036 | 1.36 | 0.173 | 0.980 | 1.122 |
| ex-smoker | 0.962 | 0.030 | -1.25 | 0.211 | 0.905 | 1.022 |
|  |  |  |  |  |  |  |
| BMI | 0.995 | 0.002 | -2.13 | 0.033 | 0.991 | 1.000 |
| HT_b | 1.054 | 0.026 | 2.16 | 0.031 | 1.005 | 1.106 |
| Hyperlipidaemia_b | 0.977 | 0.029 | -0.81 | 0.421 | 0.922 | 1.035 |
|  |  |  |  |  |  |  |
| Diabetes_type_b |  |  |  |  |  |  |
| T1DM | 1.329 | 0.161 | 2.34 | 0.019 | 1.047 | 1.686 |
| T2DM | 1.118 | 0.036 | 3.51 | 0.000 | 1.050 | 1.190 |
| DM - nos | 1.367 | 0.122 | 3.50 | 0.000 | 1.148 | 1.629 |
|  |  |  |  |  |  |  |
| HF_b | 1.193 | 0.053 | 3.98 | 0.000 | 1.094 | 1.301 |
| AF_b | 1.013 | 0.039 | 0.32 | 0.748 | 0.938 | 1.093 |
| Heart_valve_dis_b | 1.149 | 0.112 | 1.43 | 0.152 | 0.950 | 1.390 |
| VT_VF_b | 0.575 | 0.129 | -2.47 | 0.013 | 0.371 | 0.892 |
| Cardiomyopathy_b | 0.765 | 0.134 | -1.53 | 0.126 | 0.542 | 1.078 |
| CV_procedures_b | 0.735 | 0.031 | -7.31 | 0.000 | 0.676 | 0.798 |
| TIA_stroke_b | 1.099 | 0.040 | 2.62 | 0.009 | 1.024 | 1.179 |
| PVD_b | 1.368 | 0.059 | 7.31 | 0.000 | 1.258 | 1.489 |
| CKD_b | 1.151 | 0.034 | 4.70 | 0.000 | 1.085 | 1.221 |
| Hypothyroidism_b | 1.058 | 0.044 | 1.36 | 0.174 | 0.975 | 1.148 |
| Liver_dis_b | 1.596 | 0.358 | 2.09 | 0.037 | 1.029 | 2.477 |
| Lupus_b | 1.414 | 0.255 | 1.92 | 0.055 | 0.993 | 2.013 |
| Erectile_dysfunction_b | 0.968 | 0.041 | -0.76 | 0.445 | 0.892 | 1.051 |
| Any_tumour_b | 1.037 | 0.032 | 1.17 | 0.244 | 0.975 | 1.103 |
| Menopause_b | 0.865 | 0.039 | -3.19 | 0.001 | 0.792 | 0.946 |
| Dementia_b | 0.983 | 0.071 | -0.24 | 0.811 | 0.853 | 1.132 |
| RA_b | 0.844 | 0.057 | -2.53 | 0.011 | 0.740 | 0.963 |
| _cons | 0.058 | 0.007 | -24.44 | 0.000 | 0.046 | 0.073 |

**Performance in validation dataset (CPRD GOLD) - External validation - Index AMI cases**

| AUROC (95% CI): 0.567 (0.558; 0.577) |  |
| --- | --- |
| 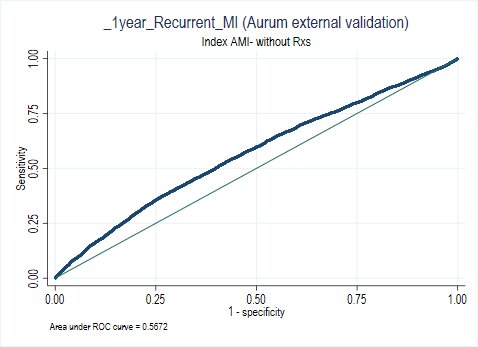 | 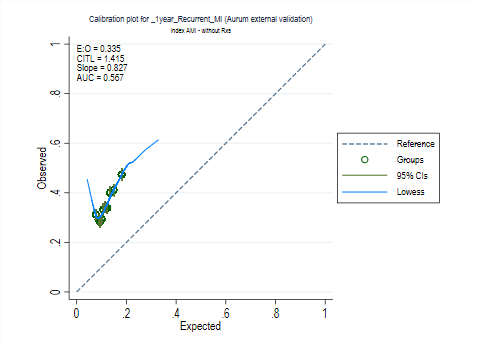 |
| 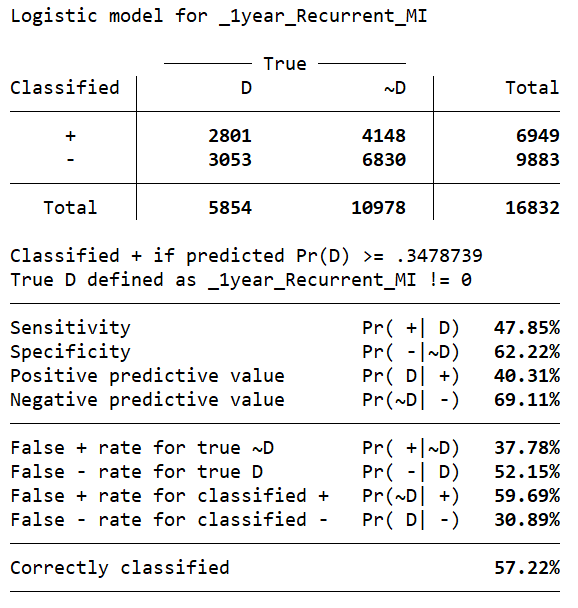 | % Correctly classified: 57.22 |

### 5) 1-year CV mortality

| _1year_CV_death | Odds ratio | Std. err. | z | P>|z| | [95% conf. interval] | |
| --- | --- | --- | --- | --- | --- | --- |
| age | 1.077 | 0.002 | 37.86 | 0.000 | 1.073 | 1.081 |
|  |  |  |  |  |  |  |
| gender |  |  |  |  |  |  |
| Female | 0.900 | 0.035 | -2.66 | 0.008 | 0.834 | 0.973 |
|  |  |  |  |  |  |  |
| IMD |  |  |  |  |  |  |
| Q2 | 1.012 | 0.054 | 0.22 | 0.829 | 0.911 | 1.123 |
| Q3 | 1.107 | 0.059 | 1.92 | 0.055 | 0.998 | 1.229 |
| Q4 | 1.174 | 0.063 | 3.00 | 0.003 | 1.057 | 1.304 |
| Q5-most deprived | 1.174 | 0.065 | 2.89 | 0.004 | 1.053 | 1.308 |
|  |  |  |  |  |  |  |
| Other than White | 0.838 | 0.070 | -2.10 | 0.035 | 0.711 | 0.988 |
| Drinker | 0.804 | 0.033 | -5.32 | 0.000 | 0.742 | 0.871 |
|  |  |  |  |  |  |  |
| Smoking_status |  |  |  |  |  |  |
| current smoker | 1.080 | 0.054 | 1.55 | 0.122 | 0.980 | 1.191 |
| ex-smoker | 0.798 | 0.034 | -5.25 | 0.000 | 0.734 | 0.868 |
|  |  |  |  |  |  |  |
| BMI | 0.985 | 0.003 | -4.45 | 0.000 | 0.978 | 0.991 |
| HT_b | 1.078 | 0.038 | 2.13 | 0.033 | 1.006 | 1.155 |
| Hyperlipidaemia_b | 0.953 | 0.041 | -1.12 | 0.262 | 0.877 | 1.036 |
|  |  |  |  |  |  |  |
| Diabetes_type_b |  |  |  |  |  |  |
| T1DM | 4.240 | 0.628 | 9.76 | 0.000 | 3.172 | 5.668 |
| T2DM | 1.415 | 0.061 | 8.02 | 0.000 | 1.300 | 1.540 |
| DM - nos | 1.999 | 0.231 | 5.98 | 0.000 | 1.593 | 2.508 |
|  |  |  |  |  |  |  |
| HF_b | 2.070 | 0.097 | 15.57 | 0.000 | 1.889 | 2.269 |
| AF_b | 1.443 | 0.063 | 8.41 | 0.000 | 1.325 | 1.572 |
| Heart_valve_dis_b | 1.508 | 0.158 | 3.92 | 0.000 | 1.228 | 1.851 |
| VT_VF_b | 1.032 | 0.233 | 0.14 | 0.890 | 0.663 | 1.606 |
| Cardiomyopathy_b | 1.252 | 0.244 | 1.15 | 0.249 | 0.855 | 1.834 |
| CV_procedures_b | 0.891 | 0.052 | -1.97 | 0.049 | 0.795 | 1.000 |
| TIA_stroke_b | 1.271 | 0.055 | 5.54 | 0.000 | 1.168 | 1.384 |
| PVD_b | 1.753 | 0.091 | 10.82 | 0.000 | 1.584 | 1.941 |
| CKD_b | 1.261 | 0.047 | 6.17 | 0.000 | 1.171 | 1.357 |
| Hypothyroidism_b | 0.995 | 0.055 | -0.09 | 0.932 | 0.894 | 1.108 |
| Liver_dis_b | 1.562 | 0.640 | 1.09 | 0.276 | 0.700 | 3.485 |
| Lupus_b | 2.150 | 0.530 | 3.10 | 0.002 | 1.326 | 3.486 |
| Erectile_dysfunction_b | 0.814 | 0.059 | -2.85 | 0.004 | 0.706 | 0.938 |
| Any_tumour_b | 1.019 | 0.042 | 0.45 | 0.650 | 0.940 | 1.104 |
| Menopause_b | 0.715 | 0.052 | -4.63 | 0.000 | 0.620 | 0.824 |
| Dementia_b | 1.361 | 0.100 | 4.18 | 0.000 | 1.178 | 1.573 |
| RA_b | 1.210 | 0.103 | 2.25 | 0.025 | 1.025 | 1.429 |
| _cons | 0.000 | 0.000 | -40.07 | 0.000 | 0.000 | 0.000 |

**Performance in validation dataset (CPRD GOLD) - External validation - Index AMI cases**

| AUROC (95% CI): 0.792 (0.778; 0.806) |  |
| --- | --- |
| 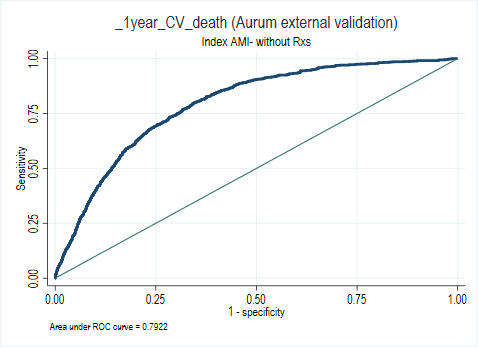 | 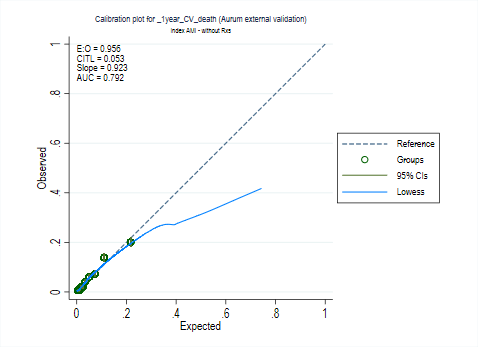 |
| 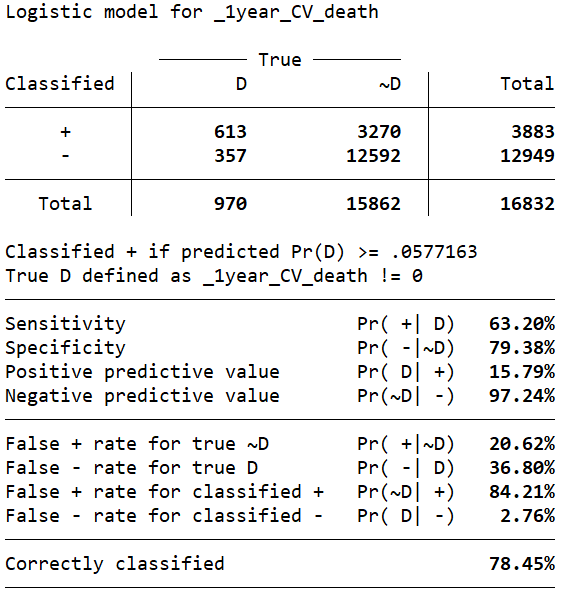 | % Correctly classified: 78.45 |

### 6) 1-year Composite 1 outcome (HF, stroke, recurrent MI, & all-cause mortality)

| _1yr_Comp1_outcome | Odds ratio | Std. err. | z | P>|z| | [95% conf. interval] | |
| --- | --- | --- | --- | --- | --- | --- |
| age | 1.037 | 0.001 | 44.57 | 0.000 | 1.035 | 1.038 |
|  |  |  |  |  |  |  |
| gender |  |  |  |  |  |  |
| Female | 1.013 | 0.021 | 0.62 | 0.535 | 0.973 | 1.054 |
|  |  |  |  |  |  |  |
| IMD |  |  |  |  |  |  |
| Q2 | 1.067 | 0.028 | 2.46 | 0.014 | 1.013 | 1.123 |
| Q3 | 1.089 | 0.029 | 3.22 | 0.001 | 1.034 | 1.147 |
| Q4 | 1.171 | 0.031 | 5.91 | 0.000 | 1.111 | 1.234 |
| Q5-most deprived | 1.273 | 0.035 | 8.89 | 0.000 | 1.207 | 1.342 |
|  |  |  |  |  |  |  |
| Other than White | 0.975 | 0.035 | -0.70 | 0.484 | 0.910 | 1.046 |
| Drinker | 0.929 | 0.021 | -3.27 | 0.001 | 0.889 | 0.971 |
|  |  |  |  |  |  |  |
| Smoking_status |  |  |  |  |  |  |
| current smoker | 1.118 | 0.028 | 4.38 | 0.000 | 1.063 | 1.175 |
| ex-smoker | 0.934 | 0.021 | -3.00 | 0.003 | 0.893 | 0.976 |
|  |  |  |  |  |  |  |
| BMI | 0.994 | 0.002 | -3.78 | 0.000 | 0.991 | 0.997 |
| HT_b | 1.049 | 0.019 | 2.66 | 0.008 | 1.013 | 1.086 |
| Hyperlipidaemia_b | 0.952 | 0.020 | -2.29 | 0.022 | 0.913 | 0.993 |
|  |  |  |  |  |  |  |
| Diabetes_type_b |  |  |  |  |  |  |
| T1DM | 2.221 | 0.201 | 8.80 | 0.000 | 1.860 | 2.654 |
| T2DM | 1.373 | 0.032 | 13.61 | 0.000 | 1.312 | 1.437 |
| DM - nos | 1.894 | 0.132 | 9.15 | 0.000 | 1.652 | 2.171 |
|  |  |  |  |  |  |  |
| HF_b | 3.349 | 0.120 | 33.66 | 0.000 | 3.121 | 3.593 |
| AF_b | 1.376 | 0.039 | 11.22 | 0.000 | 1.302 | 1.455 |
| Heart_valve_dis_b | 1.394 | 0.106 | 4.38 | 0.000 | 1.201 | 1.617 |
| VT_VF_b | 0.961 | 0.128 | -0.29 | 0.768 | 0.741 | 1.248 |
| Cardiomyopathy_b | 1.602 | 0.185 | 4.07 | 0.000 | 1.277 | 2.010 |
| CV_procedures_b | 0.719 | 0.021 | -11.21 | 0.000 | 0.678 | 0.761 |
| TIA_stroke_b | 2.040 | 0.054 | 26.81 | 0.000 | 1.937 | 2.150 |
| PVD_b | 1.574 | 0.052 | 13.64 | 0.000 | 1.475 | 1.680 |
| CKD_b | 1.246 | 0.027 | 10.00 | 0.000 | 1.193 | 1.301 |
| Hypothyroidism_b | 1.041 | 0.032 | 1.32 | 0.188 | 0.980 | 1.106 |
| Liver_dis_b | 1.516 | 0.275 | 2.29 | 0.022 | 1.062 | 2.163 |
| Lupus_b | 1.372 | 0.195 | 2.22 | 0.027 | 1.037 | 1.813 |
| Erectile_dysfunction_b | 0.963 | 0.029 | -1.25 | 0.212 | 0.907 | 1.022 |
| Any_tumour_b | 1.191 | 0.027 | 7.75 | 0.000 | 1.140 | 1.245 |
| Menopause_b | 0.798 | 0.026 | -6.95 | 0.000 | 0.748 | 0.850 |
| Dementia_b | 1.383 | 0.074 | 6.06 | 0.000 | 1.245 | 1.536 |
| RA_b | 1.075 | 0.049 | 1.58 | 0.114 | 0.983 | 1.175 |
| _cons | 0.030 | 0.003 | -40.27 | 0.000 | 0.026 | 0.036 |

**Performance in validation dataset (CPRD GOLD) - External validation - Index AMI cases**

| AUROC (95% CI): 0.657 (0.649; 0.665) |  |
| --- | --- |
| 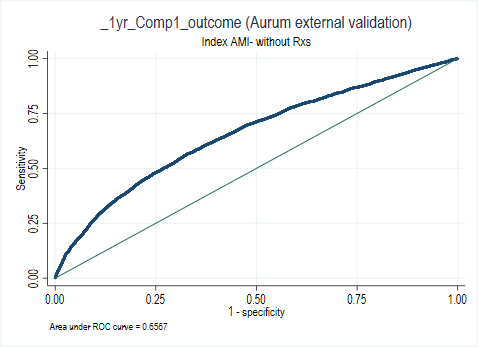 | 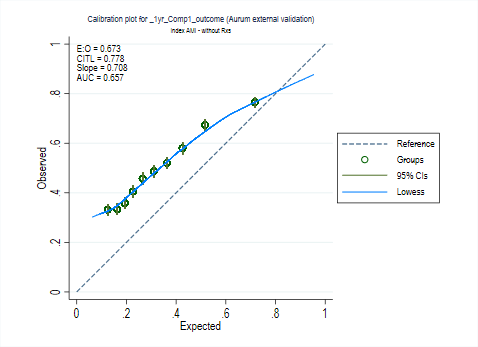 |
| 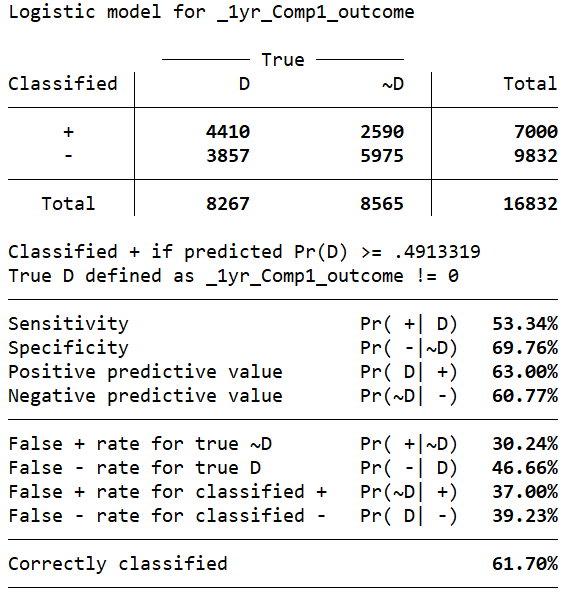 | % Correctly classified: 61.70 |

### 7) 1-year Composite 1 CV outcome (HF, stroke, recurrent MI, & CV mortality)

| _1yr_Comp1_CV_outcome | Odds ratio | Std. err. | z | P>|z| | [95% conf. interval] | |
| --- | --- | --- | --- | --- | --- | --- |
| age | 1.033 | 0.001 | 40.38 | 0.000 | 1.032 | 1.035 |
|  |  |  |  |  |  |  |
| gender |  |  |  |  |  |  |
| Female | 1.023 | 0.021 | 1.09 | 0.275 | 0.982 | 1.065 |
|  |  |  |  |  |  |  |
| IMD |  |  |  |  |  |  |
| Q2 | 1.031 | 0.027 | 1.16 | 0.248 | 0.979 | 1.087 |
| Q3 | 1.058 | 0.028 | 2.12 | 0.034 | 1.004 | 1.115 |
| Q4 | 1.153 | 0.031 | 5.27 | 0.000 | 1.093 | 1.215 |
| Q5-most deprived | 1.231 | 0.034 | 7.60 | 0.000 | 1.167 | 1.299 |
|  |  |  |  |  |  |  |
| Other than White | 0.990 | 0.035 | -0.28 | 0.779 | 0.923 | 1.062 |
| Drinker | 0.918 | 0.021 | -3.77 | 0.000 | 0.879 | 0.960 |
|  |  |  |  |  |  |  |
| Smoking_status |  |  |  |  |  |  |
| current smoker | 1.095 | 0.028 | 3.53 | 0.000 | 1.041 | 1.151 |
| ex-smoker | 0.946 | 0.022 | -2.42 | 0.015 | 0.904 | 0.989 |
|  |  |  |  |  |  |  |
| BMI | 0.999 | 0.002 | -0.31 | 0.753 | 0.996 | 1.003 |
| HT_b | 1.053 | 0.019 | 2.87 | 0.004 | 1.017 | 1.091 |
| Hyperlipidaemia_b | 0.972 | 0.021 | -1.33 | 0.182 | 0.931 | 1.014 |
|  |  |  |  |  |  |  |
| Diabetes_type_b |  |  |  |  |  |  |
| T1DM | 2.172 | 0.197 | 8.56 | 0.000 | 1.818 | 2.594 |
| T2DM | 1.347 | 0.031 | 12.74 | 0.000 | 1.287 | 1.410 |
| DM - nos | 1.838 | 0.128 | 8.77 | 0.000 | 1.605 | 2.106 |
|  |  |  |  |  |  |  |
| HF_b | 3.391 | 0.119 | 34.86 | 0.000 | 3.166 | 3.632 |
| AF_b | 1.375 | 0.039 | 11.23 | 0.000 | 1.301 | 1.454 |
| Heart_valve_dis_b | 1.368 | 0.103 | 4.17 | 0.000 | 1.181 | 1.584 |
| VT_VF_b | 1.162 | 0.153 | 1.14 | 0.255 | 0.898 | 1.504 |
| Cardiomyopathy_b | 1.682 | 0.193 | 4.53 | 0.000 | 1.343 | 2.107 |
| CV_procedures_b | 0.736 | 0.022 | -10.31 | 0.000 | 0.694 | 0.780 |
| TIA_stroke_b | 2.105 | 0.056 | 28.23 | 0.000 | 1.999 | 2.217 |
| PVD_b | 1.542 | 0.051 | 13.05 | 0.000 | 1.445 | 1.646 |
| CKD_b | 1.200 | 0.027 | 8.23 | 0.000 | 1.149 | 1.253 |
| Hypothyroidism_b | 1.010 | 0.031 | 0.33 | 0.742 | 0.951 | 1.073 |
| Liver_dis_b | 1.495 | 0.273 | 2.20 | 0.028 | 1.045 | 2.139 |
| Lupus_b | 1.277 | 0.185 | 1.69 | 0.091 | 0.962 | 1.696 |
| Erectile_dysfunction_b | 1.014 | 0.031 | 0.47 | 0.639 | 0.956 | 1.077 |
| Any_tumour_b | 1.044 | 0.024 | 1.87 | 0.062 | 0.998 | 1.092 |
| Menopause_b | 0.792 | 0.026 | -7.07 | 0.000 | 0.742 | 0.845 |
| Dementia_b | 1.110 | 0.059 | 1.96 | 0.050 | 1.000 | 1.231 |
| RA_b | 1.072 | 0.049 | 1.52 | 0.128 | 0.980 | 1.174 |
| _cons | 0.031 | 0.003 | -39.83 | 0.000 | 0.026 | 0.036 |

**Performance in validation dataset (CPRD GOLD) - External validation - Index AMI cases**

| AUROC (95% CI): 0.648 (0.641; 0.656) |  |
| --- | --- |
| 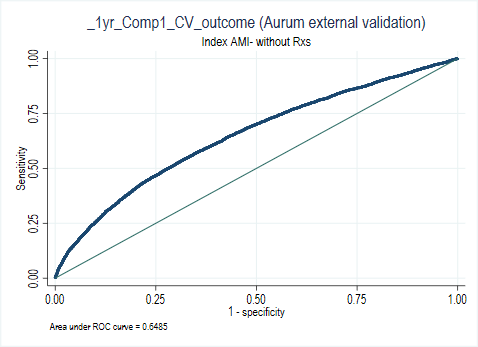 | 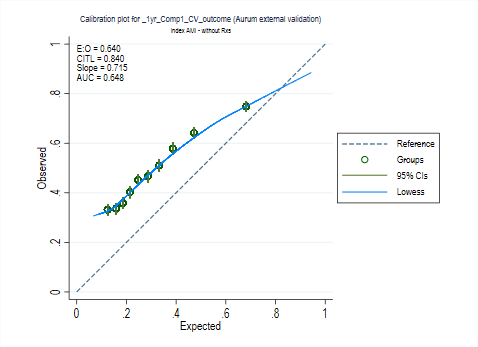 |
| 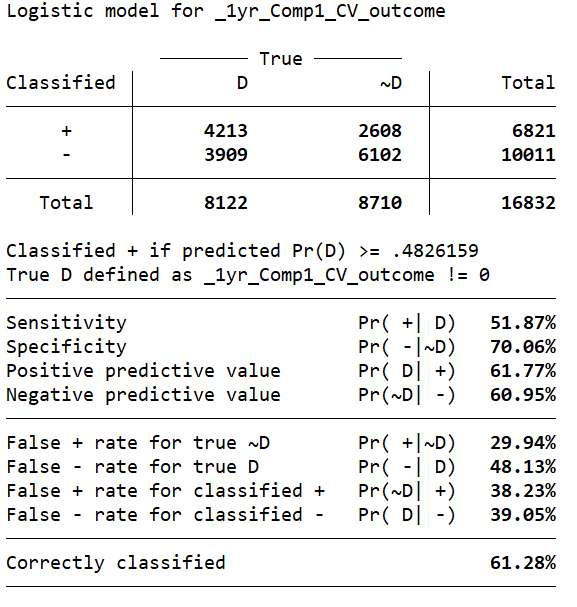 | % Correctly classified: 61.28 |

### 8) 1-year Composite 2 outcome (HF, stroke, & all-cause mortality)

|  | | | | | | |
| --- | --- | --- | --- | --- | --- | --- |
| _1yr_Comp2_outcome | Odds ratio | Std. err. | z | P>|z| | [95% conf. interval] | |
| age | 1.052 | 0.001 | 54.42 | 0.000 | 1.050 | 1.054 |
|  |  |  |  |  |  |  |
| gender |  |  |  |  |  |  |
| Female | 1.013 | 0.022 | 0.58 | 0.564 | 0.970 | 1.058 |
|  |  |  |  |  |  |  |
| IMD |  |  |  |  |  |  |
| Q2 | 1.075 | 0.031 | 2.47 | 0.014 | 1.015 | 1.138 |
| Q3 | 1.144 | 0.033 | 4.60 | 0.000 | 1.080 | 1.212 |
| Q4 | 1.231 | 0.036 | 7.04 | 0.000 | 1.162 | 1.304 |
| Q5-most deprived | 1.394 | 0.042 | 11.11 | 0.000 | 1.314 | 1.478 |
|  |  |  |  |  |  |  |
| Other than White | 0.977 | 0.039 | -0.59 | 0.554 | 0.903 | 1.056 |
| Drinker | 0.869 | 0.021 | -5.82 | 0.000 | 0.829 | 0.911 |
|  |  |  |  |  |  |  |
| Smoking_status |  |  |  |  |  |  |
| current smoker | 1.194 | 0.033 | 6.34 | 0.000 | 1.131 | 1.262 |
| ex-smoker | 0.958 | 0.024 | -1.72 | 0.085 | 0.912 | 1.006 |
|  |  |  |  |  |  |  |
| BMI | 0.994 | 0.002 | -3.43 | 0.001 | 0.990 | 0.997 |
| HT_b | 1.066 | 0.021 | 3.31 | 0.001 | 1.027 | 1.108 |
| Hyperlipidaemia_b | 0.968 | 0.023 | -1.39 | 0.165 | 0.925 | 1.013 |
|  |  |  |  |  |  |  |
| Diabetes_type_b |  |  |  |  |  |  |
| T1DM | 2.680 | 0.261 | 10.14 | 0.000 | 2.215 | 3.243 |
| T2DM | 1.422 | 0.035 | 14.12 | 0.000 | 1.354 | 1.493 |
| DM - nos | 2.066 | 0.151 | 9.95 | 0.000 | 1.791 | 2.384 |
|  |  |  |  |  |  |  |
| HF_b | 3.917 | 0.141 | 37.94 | 0.000 | 3.651 | 4.204 |
| AF_b | 1.474 | 0.043 | 13.26 | 0.000 | 1.392 | 1.561 |
| Heart_valve_dis_b | 1.303 | 0.102 | 3.40 | 0.001 | 1.119 | 1.518 |
| VT_VF_b | 1.080 | 0.152 | 0.55 | 0.583 | 0.820 | 1.422 |
| Cardiomyopathy_b | 1.794 | 0.214 | 4.91 | 0.000 | 1.421 | 2.266 |
| CV_procedures_b | 0.793 | 0.025 | -7.28 | 0.000 | 0.745 | 0.844 |
| TIA_stroke_b | 2.319 | 0.063 | 30.95 | 0.000 | 2.199 | 2.446 |
| PVD_b | 1.651 | 0.057 | 14.56 | 0.000 | 1.543 | 1.766 |
| CKD_b | 1.243 | 0.029 | 9.47 | 0.000 | 1.188 | 1.301 |
| Hypothyroidism_b | 1.001 | 0.033 | 0.03 | 0.974 | 0.939 | 1.067 |
| Liver_dis_b | 1.006 | 0.225 | 0.03 | 0.980 | 0.649 | 1.559 |
| Lupus_b | 1.384 | 0.215 | 2.09 | 0.036 | 1.021 | 1.876 |
| Erectile_dysfunction_b | 0.979 | 0.033 | -0.62 | 0.534 | 0.916 | 1.046 |
| Any_tumour_b | 1.252 | 0.030 | 9.41 | 0.000 | 1.195 | 1.312 |
| Menopause_b | 0.782 | 0.028 | -6.85 | 0.000 | 0.729 | 0.839 |
| Dementia_b | 1.426 | 0.077 | 6.54 | 0.000 | 1.282 | 1.585 |
| RA_b | 1.174 | 0.057 | 3.30 | 0.001 | 1.067 | 1.291 |
| _cons | 0.007 | 0.001 | -50.75 | 0.000 | 0.005 | 0.008 |

**Performance in validation dataset (CPRD GOLD) - External validation - Index AMI cases**

| AUROC (95% CI): 0.744 (0.736; 0.753) |  |
| --- | --- |
| 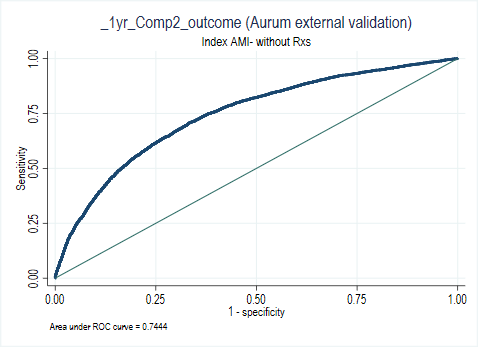 | 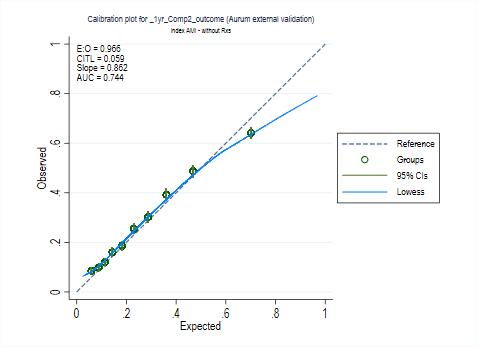 |
| 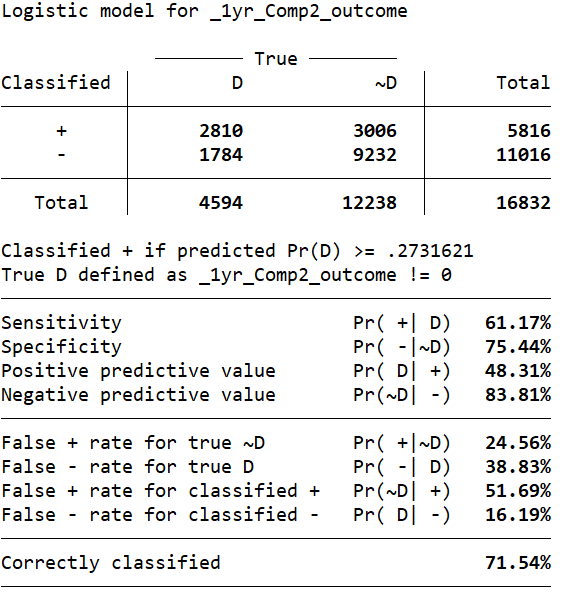 | % Correctly classified: 71.54 |

### 9) 1-year Composite 2 CV outcome (HF, stroke, & CV mortality)

|  | | | | | | |
| --- | --- | --- | --- | --- | --- | --- |
| _1yr_Comp2_CV_outcome | Odds ratio | Std. err. | z | P>|z| | [95% conf. interval] | |
| age | 1.047 | 0.001 | 48.02 | 0.000 | 1.045 | 1.049 |
|  |  |  |  |  |  |  |
| gender |  |  |  |  |  |  |
| Female | 0.974 | 0.022 | -1.15 | 0.250 | 0.931 | 1.019 |
|  |  |  |  |  |  |  |
| IMD |  |  |  |  |  |  |
| Q2 | 1.057 | 0.032 | 1.85 | 0.064 | 0.997 | 1.122 |
| Q3 | 1.124 | 0.034 | 3.88 | 0.000 | 1.060 | 1.193 |
| Q4 | 1.242 | 0.038 | 7.14 | 0.000 | 1.170 | 1.318 |
| Q5-most deprived | 1.397 | 0.043 | 10.89 | 0.000 | 1.316 | 1.484 |
|  |  |  |  |  |  |  |
| Other than White | 0.947 | 0.039 | -1.31 | 0.189 | 0.874 | 1.027 |
| Drinker | 0.890 | 0.022 | -4.70 | 0.000 | 0.848 | 0.935 |
|  |  |  |  |  |  |  |
| Smoking_status |  |  |  |  |  |  |
| current smoker | 1.120 | 0.032 | 3.95 | 0.000 | 1.059 | 1.185 |
| ex-smoker | 0.926 | 0.024 | -3.00 | 0.003 | 0.881 | 0.974 |
|  |  |  |  |  |  |  |
| BMI | 1.000 | 0.002 | -0.19 | 0.846 | 0.996 | 1.003 |
| HT_b | 1.103 | 0.022 | 4.93 | 0.000 | 1.061 | 1.147 |
| Hyperlipidaemia_b | 0.987 | 0.024 | -0.55 | 0.579 | 0.942 | 1.034 |
|  |  |  |  |  |  |  |
| Diabetes_type_b |  |  |  |  |  |  |
| T1DM | 2.580 | 0.254 | 9.63 | 0.000 | 2.127 | 3.128 |
| T2DM | 1.436 | 0.036 | 14.28 | 0.000 | 1.366 | 1.509 |
| DM - nos | 2.075 | 0.152 | 9.97 | 0.000 | 1.798 | 2.395 |
|  |  |  |  |  |  |  |
| HF_b | 4.137 | 0.146 | 40.30 | 0.000 | 3.861 | 4.433 |
| AF_b | 1.457 | 0.043 | 12.75 | 0.000 | 1.375 | 1.543 |
| Heart_valve_dis_b | 1.408 | 0.110 | 4.40 | 0.000 | 1.209 | 1.640 |
| VT_VF_b | 1.083 | 0.154 | 0.56 | 0.576 | 0.820 | 1.430 |
| Cardiomyopathy_b | 1.838 | 0.218 | 5.13 | 0.000 | 1.457 | 2.319 |
| CV_procedures_b | 0.809 | 0.026 | -6.51 | 0.000 | 0.759 | 0.862 |
| TIA_stroke_b | 2.516 | 0.068 | 34.03 | 0.000 | 2.386 | 2.654 |
| PVD_b | 1.625 | 0.056 | 13.98 | 0.000 | 1.518 | 1.740 |
| CKD_b | 1.274 | 0.030 | 10.35 | 0.000 | 1.217 | 1.334 |
| Hypothyroidism_b | 0.973 | 0.033 | -0.81 | 0.420 | 0.911 | 1.040 |
| Liver_dis_b | 1.100 | 0.247 | 0.42 | 0.672 | 0.708 | 1.708 |
| Lupus_b | 1.476 | 0.233 | 2.47 | 0.013 | 1.084 | 2.010 |
| Erectile_dysfunction_b | 0.970 | 0.034 | -0.86 | 0.387 | 0.906 | 1.039 |
| Any_tumour_b | 1.061 | 0.026 | 2.40 | 0.017 | 1.011 | 1.114 |
| Menopause_b | 0.792 | 0.029 | -6.28 | 0.000 | 0.736 | 0.851 |
| Dementia_b | 1.081 | 0.060 | 1.42 | 0.156 | 0.970 | 1.205 |
| RA_b | 1.115 | 0.056 | 2.16 | 0.031 | 1.010 | 1.230 |
| _cons | 0.007 | 0.001 | -48.86 | 0.000 | 0.006 | 0.008 |

**Performance in validation dataset (CPRD GOLD) - External validation - Index AMI cases**

| AUROC (95% CI): 0.734 (0.725; 0.743) |  |
| --- | --- |
| 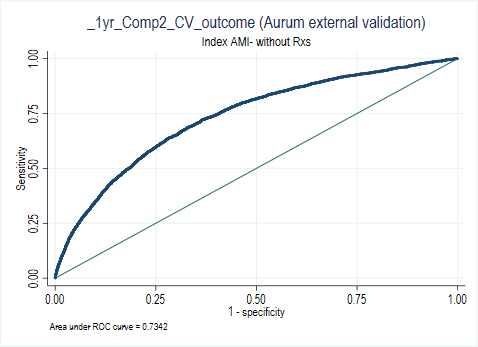 | 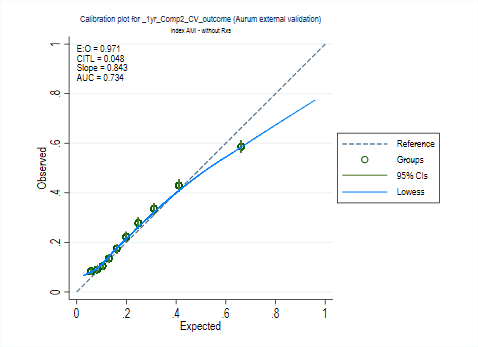 |
| 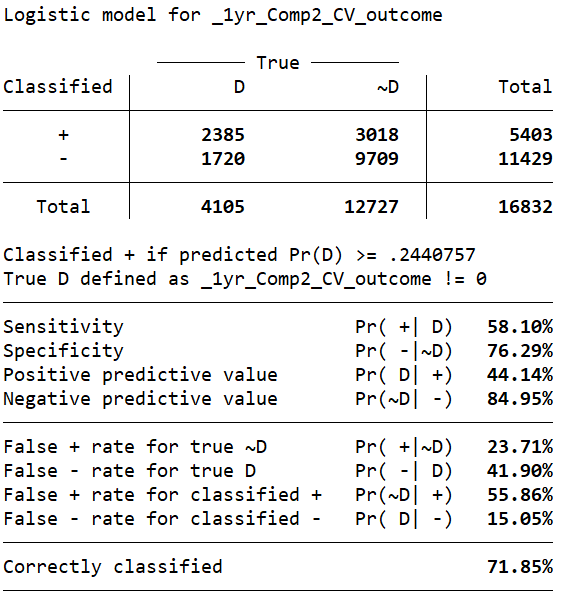 | % Correctly classified: 71.85 |

## Internal validation (CPRD Aurum) - with therapies

### 1) 1-year all-cause mortality

|  | | | | | | |
| --- | --- | --- | --- | --- | --- | --- |
| _1year_All_cause_death | Odds ratio | Std. err. | z | P>|z| | [95% conf. interval] | |
| age | 1.068 | 0.002 | 44.26 | 0.000 | 1.065 | 1.072 |
|  |  |  |  |  |  |  |
| gender |  |  |  |  |  |  |
| Female | 0.901 | 0.027 | -3.47 | 0.001 | 0.850 | 0.956 |
|  |  |  |  |  |  |  |
| IMD |  |  |  |  |  |  |
| Q2 | 1.069 | 0.043 | 1.67 | 0.095 | 0.988 | 1.157 |
| Q3 | 1.144 | 0.046 | 3.35 | 0.001 | 1.057 | 1.239 |
| Q4 | 1.252 | 0.051 | 5.54 | 0.000 | 1.157 | 1.356 |
| Q5-most deprived | 1.298 | 0.054 | 6.28 | 0.000 | 1.196 | 1.408 |
|  |  |  |  |  |  |  |
| Other than White |  |  |  |  |  |  |
| non-White | 0.843 | 0.052 | -2.74 | 0.006 | 0.746 | 0.952 |
|  |  |  |  |  |  |  |
| Drinker |  |  |  |  |  |  |
| drinker | 0.850 | 0.027 | -5.13 | 0.000 | 0.799 | 0.905 |
|  |  |  |  |  |  |  |
| Smoking_status |  |  |  |  |  |  |
| current smoker | 1.231 | 0.047 | 5.46 | 0.000 | 1.142 | 1.326 |
| ex-smoker | 0.867 | 0.029 | -4.30 | 0.000 | 0.813 | 0.926 |
|  |  |  |  |  |  |  |
| BMI | 0.968 | 0.003 | -12.40 | 0.000 | 0.962 | 0.973 |
| HT_b | 0.865 | 0.024 | -5.14 | 0.000 | 0.818 | 0.914 |
| Antihypertensives_b | 1.173 | 0.057 | 3.26 | 0.001 | 1.066 | 1.291 |
| Hyperlipidaemia_b | 0.909 | 0.031 | -2.80 | 0.005 | 0.851 | 0.972 |
| Lipid_reg_treatment_b | 0.840 | 0.028 | -5.33 | 0.000 | 0.787 | 0.895 |
|  |  |  |  |  |  |  |
| Diabetes_type_b |  |  |  |  |  |  |
| T1DM | 2.383 | 0.336 | 6.16 | 0.000 | 1.808 | 3.142 |
| T2DM | 1.103 | 0.069 | 1.58 | 0.114 | 0.977 | 1.246 |
| DM - nos | 1.667 | 0.173 | 4.93 | 0.000 | 1.360 | 2.042 |
|  |  |  |  |  |  |  |
| Antidiabetics_b | 1.371 | 0.090 | 4.78 | 0.000 | 1.204 | 1.560 |
| Diuretics_b | 1.505 | 0.055 | 11.26 | 0.000 | 1.402 | 1.616 |
| Anticoagulants_b | 1.204 | 0.054 | 4.11 | 0.000 | 1.102 | 1.315 |
| Antiplatelets_b | 1.158 | 0.038 | 4.50 | 0.000 | 1.086 | 1.234 |
| HF_b | 1.756 | 0.069 | 14.36 | 0.000 | 1.626 | 1.896 |
| AF_b | 1.143 | 0.045 | 3.43 | 0.001 | 1.059 | 1.234 |
| Heart_valve_dis_b | 1.368 | 0.120 | 3.58 | 0.000 | 1.153 | 1.623 |
| VT_VF_b | 0.748 | 0.144 | -1.51 | 0.131 | 0.513 | 1.090 |
| Cardiomyopathy_b | 1.147 | 0.178 | 0.88 | 0.377 | 0.846 | 1.556 |
| CV_procedures_b | 0.766 | 0.035 | -5.83 | 0.000 | 0.700 | 0.838 |
| TIA_stroke_b | 1.301 | 0.045 | 7.56 | 0.000 | 1.215 | 1.393 |
| PVD_b | 1.626 | 0.068 | 11.62 | 0.000 | 1.498 | 1.765 |
| CKD_b | 1.257 | 0.036 | 7.88 | 0.000 | 1.188 | 1.331 |
| Hypothyroidism_b | 1.015 | 0.043 | 0.36 | 0.715 | 0.935 | 1.102 |
| Liver_dis_b | 1.688 | 0.499 | 1.77 | 0.077 | 0.945 | 3.013 |
| Lupus_b | 1.412 | 0.296 | 1.65 | 0.099 | 0.937 | 2.129 |
| Erectile_dysfunction_b | 0.815 | 0.043 | -3.87 | 0.000 | 0.735 | 0.904 |
| Any_tumour_b | 1.481 | 0.044 | 13.25 | 0.000 | 1.398 | 1.570 |
| Menopause_b | 0.806 | 0.041 | -4.25 | 0.000 | 0.729 | 0.890 |
| Dementia_b | 1.768 | 0.102 | 9.90 | 0.000 | 1.579 | 1.979 |
| RA_b | 1.243 | 0.079 | 3.42 | 0.001 | 1.098 | 1.408 |
| _cons | 0.001 | 0.000 | -44.35 | 0.000 | 0.001 | 0.002 |

**Performance in validation dataset (20% of practices) - Internal validation - Index AMI cases**

| AUROC (95% CI): 0.802 (0.793; 0.812) |  |
| --- | --- |
| 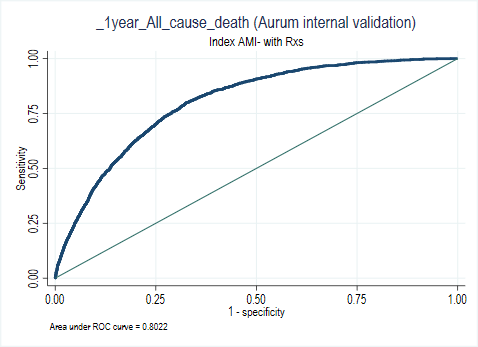 | 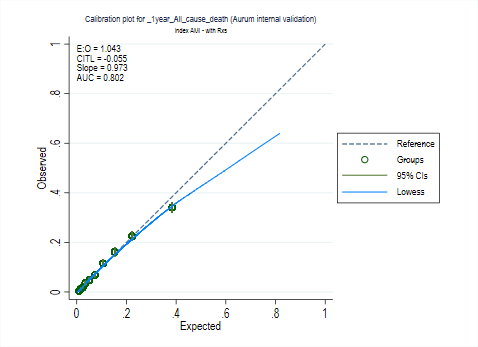 |
| 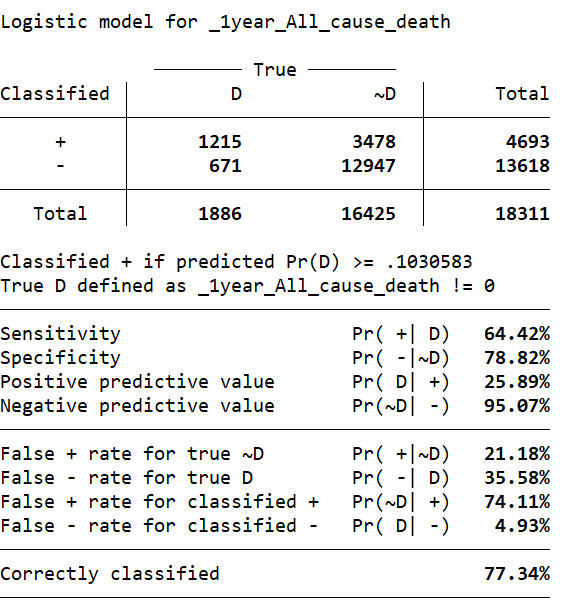 | % Correctly classified: 77.34 |

### 2) 1-year Stroke

|  | | | | | | |
| --- | --- | --- | --- | --- | --- | --- |
| _1year_Stroke | Odds ratio | Std. err. | z | P>|z| | [95% conf. interval] | |
| age | 1.036 | 0.002 | 20.11 | 0.000 | 1.032 | 1.039 |
|  |  |  |  |  |  |  |
| gender |  |  |  |  |  |  |
| Female | 1.036 | 0.039 | 0.96 | 0.339 | 0.963 | 1.115 |
|  |  |  |  |  |  |  |
| IMD |  |  |  |  |  |  |
| Q2 | 0.956 | 0.047 | -0.91 | 0.365 | 0.868 | 1.054 |
| Q3 | 1.063 | 0.052 | 1.24 | 0.216 | 0.965 | 1.171 |
| Q4 | 1.125 | 0.055 | 2.38 | 0.017 | 1.021 | 1.239 |
| Q5-most deprived | 1.089 | 0.055 | 1.69 | 0.092 | 0.986 | 1.203 |
|  |  |  |  |  |  |  |
| Other than White |  |  |  |  |  |  |
| non-White | 0.981 | 0.068 | -0.27 | 0.784 | 0.857 | 1.124 |
|  |  |  |  |  |  |  |
| Drinker |  |  |  |  |  |  |
| drinker | 0.867 | 0.033 | -3.71 | 0.000 | 0.804 | 0.935 |
|  |  |  |  |  |  |  |
| Smoking_status |  |  |  |  |  |  |
| current smoker | 1.132 | 0.053 | 2.63 | 0.009 | 1.032 | 1.242 |
| ex-smoker | 0.932 | 0.039 | -1.70 | 0.090 | 0.860 | 1.011 |
|  |  |  |  |  |  |  |
| BMI | 0.988 | 0.003 | -3.77 | 0.000 | 0.982 | 0.994 |
| HT_b | 1.064 | 0.037 | 1.79 | 0.074 | 0.994 | 1.139 |
| Antihypertensives_b | 1.343 | 0.083 | 4.78 | 0.000 | 1.190 | 1.517 |
| Hyperlipidaemia_b | 1.128 | 0.043 | 3.18 | 0.001 | 1.048 | 1.216 |
| Lipid_reg_treatment_b | 1.001 | 0.042 | 0.03 | 0.978 | 0.923 | 1.086 |
|  |  |  |  |  |  |  |
| Diabetes_type_b |  |  |  |  |  |  |
| T1DM | 1.749 | 0.286 | 3.42 | 0.001 | 1.269 | 2.410 |
| T2DM | 1.016 | 0.076 | 0.21 | 0.832 | 0.877 | 1.177 |
| DM - nos | 1.341 | 0.164 | 2.41 | 0.016 | 1.056 | 1.704 |
|  |  |  |  |  |  |  |
| Antidiabetics_b | 1.304 | 0.103 | 3.35 | 0.001 | 1.117 | 1.523 |
| Diuretics_b | 1.148 | 0.049 | 3.27 | 0.001 | 1.057 | 1.248 |
| Anticoagulants_b | 1.035 | 0.056 | 0.63 | 0.531 | 0.930 | 1.151 |
| Antiplatelets_b | 1.220 | 0.052 | 4.63 | 0.000 | 1.121 | 1.326 |
| HF_b | 1.111 | 0.056 | 2.09 | 0.036 | 1.007 | 1.227 |
| AF_b | 1.390 | 0.064 | 7.11 | 0.000 | 1.270 | 1.523 |
| Heart_valve_dis_b | 1.123 | 0.120 | 1.08 | 0.280 | 0.910 | 1.384 |
| VT_VF_b | 0.886 | 0.190 | -0.57 | 0.572 | 0.583 | 1.348 |
| Cardiomyopathy_b | 1.381 | 0.246 | 1.81 | 0.070 | 0.974 | 1.958 |
| CV_procedures_b | 0.786 | 0.041 | -4.59 | 0.000 | 0.709 | 0.871 |
| TIA_stroke_b | 6.237 | 0.219 | 52.19 | 0.000 | 5.822 | 6.680 |
| PVD_b | 1.471 | 0.073 | 7.83 | 0.000 | 1.336 | 1.620 |
| CKD_b | 1.105 | 0.040 | 2.78 | 0.005 | 1.030 | 1.185 |
| Hypothyroidism_b | 1.023 | 0.052 | 0.45 | 0.649 | 0.926 | 1.131 |
| Liver_dis_b | 0.714 | 0.315 | -0.76 | 0.445 | 0.300 | 1.696 |
| Lupus_b | 1.188 | 0.294 | 0.70 | 0.486 | 0.732 | 1.930 |
| Erectile_dysfunction_b | 1.003 | 0.059 | 0.05 | 0.962 | 0.894 | 1.125 |
| Any_tumour_b | 1.008 | 0.039 | 0.20 | 0.843 | 0.933 | 1.088 |
| Menopause_b | 0.942 | 0.055 | -1.03 | 0.305 | 0.840 | 1.056 |
| Dementia_b | 1.326 | 0.098 | 3.84 | 0.000 | 1.148 | 1.532 |
| RA_b | 1.042 | 0.084 | 0.51 | 0.610 | 0.890 | 1.221 |
| _cons | 0.003 | 0.000 | -32.91 | 0.000 | 0.002 | 0.004 |

**Performance in validation dataset (20% of practices) - Internal validation - Index AMI cases**

| AUROC (95% CI): 0.806 (0.793; 0.820) |  |
| --- | --- |
| 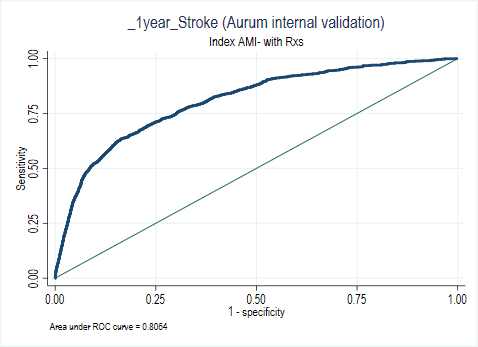 | 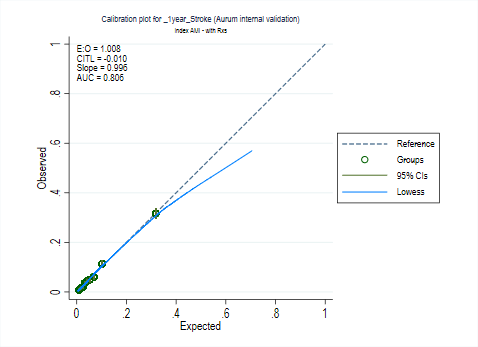 |
| 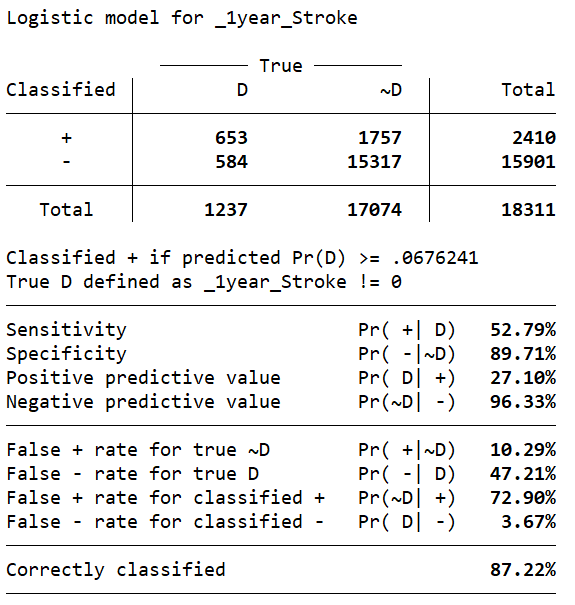 | % Correctly classified: 87.22 |

### 3) 1-year heart failure (HF)

|  | | | | | | |
| --- | --- | --- | --- | --- | --- | --- |
| _1year_HF | Odds ratio | Std. err. | z | P>|z| | [95% conf. interval] | |
| age | 1.038 | 0.001 | 33.25 | 0.000 | 1.036 | 1.040 |
|  |  |  |  |  |  |  |
| gender |  |  |  |  |  |  |
| Female | 0.897 | 0.023 | -4.21 | 0.000 | 0.853 | 0.944 |
|  |  |  |  |  |  |  |
| IMD |  |  |  |  |  |  |
| Q2 | 1.099 | 0.037 | 2.81 | 0.005 | 1.029 | 1.174 |
| Q3 | 1.041 | 0.035 | 1.19 | 0.233 | 0.974 | 1.113 |
| Q4 | 1.177 | 0.040 | 4.80 | 0.000 | 1.101 | 1.258 |
| Q5-most deprived | 1.322 | 0.045 | 8.17 | 0.000 | 1.237 | 1.414 |
|  |  |  |  |  |  |  |
| Other than White |  |  |  |  |  |  |
| non-White | 1.004 | 0.046 | 0.08 | 0.939 | 0.918 | 1.097 |
|  |  |  |  |  |  |  |
| Drinker |  |  |  |  |  |  |
| drinker | 0.870 | 0.024 | -5.11 | 0.000 | 0.825 | 0.918 |
|  |  |  |  |  |  |  |
| Smoking_status |  |  |  |  |  |  |
| current smoker | 1.115 | 0.036 | 3.39 | 0.001 | 1.047 | 1.188 |
| ex-smoker | 0.962 | 0.027 | -1.37 | 0.171 | 0.910 | 1.017 |
|  |  |  |  |  |  |  |
| BMI | 1.000 | 0.002 | 0.17 | 0.866 | 0.996 | 1.004 |
| HT_b | 0.997 | 0.024 | -0.12 | 0.902 | 0.950 | 1.046 |
| Antihypertensives_b | 0.905 | 0.034 | -2.67 | 0.007 | 0.842 | 0.974 |
| Hyperlipidaemia_b | 0.995 | 0.028 | -0.19 | 0.848 | 0.942 | 1.051 |
| Lipid_reg_treatment_b | 0.907 | 0.025 | -3.48 | 0.001 | 0.859 | 0.958 |
|  |  |  |  |  |  |  |
| Diabetes_type_b |  |  |  |  |  |  |
| T1DM | 1.839 | 0.218 | 5.13 | 0.000 | 1.457 | 2.320 |
| T2DM | 1.132 | 0.060 | 2.34 | 0.019 | 1.021 | 1.256 |
| DM - nos | 1.612 | 0.143 | 5.40 | 0.000 | 1.356 | 1.917 |
|  |  |  |  |  |  |  |
| Antidiabetics_b | 1.359 | 0.076 | 5.49 | 0.000 | 1.218 | 1.516 |
| Diuretics_b | 1.481 | 0.044 | 13.10 | 0.000 | 1.397 | 1.571 |
| Anticoagulants_b | 1.230 | 0.049 | 5.19 | 0.000 | 1.137 | 1.330 |
| Antiplatelets_b | 1.009 | 0.028 | 0.32 | 0.746 | 0.956 | 1.064 |
| HF_b | 4.688 | 0.161 | 44.95 | 0.000 | 4.383 | 5.015 |
| AF_b | 1.321 | 0.046 | 7.95 | 0.000 | 1.234 | 1.415 |
| Heart_valve_dis_b | 1.311 | 0.106 | 3.35 | 0.001 | 1.119 | 1.536 |
| VT_VF_b | 1.004 | 0.153 | 0.03 | 0.980 | 0.745 | 1.353 |
| Cardiomyopathy_b | 1.247 | 0.153 | 1.80 | 0.072 | 0.980 | 1.588 |
| CV_procedures_b | 0.836 | 0.031 | -4.87 | 0.000 | 0.778 | 0.899 |
| TIA_stroke_b | 1.070 | 0.034 | 2.10 | 0.036 | 1.004 | 1.139 |
| PVD_b | 1.445 | 0.055 | 9.71 | 0.000 | 1.341 | 1.556 |
| CKD_b | 1.194 | 0.031 | 6.86 | 0.000 | 1.135 | 1.257 |
| Hypothyroidism_b | 0.954 | 0.036 | -1.27 | 0.203 | 0.886 | 1.026 |
| Liver_dis_b | 1.018 | 0.262 | 0.07 | 0.946 | 0.614 | 1.687 |
| Lupus_b | 1.864 | 0.303 | 3.83 | 0.000 | 1.356 | 2.563 |
| Erectile_dysfunction_b | 1.019 | 0.039 | 0.50 | 0.619 | 0.945 | 1.099 |
| Any_tumour_b | 1.101 | 0.030 | 3.52 | 0.000 | 1.043 | 1.161 |
| Menopause_b | 0.802 | 0.034 | -5.22 | 0.000 | 0.738 | 0.871 |
| Dementia_b | 0.762 | 0.049 | -4.24 | 0.000 | 0.672 | 0.864 |
| RA_b | 1.208 | 0.066 | 3.46 | 0.001 | 1.086 | 1.345 |
| _cons | 0.009 | 0.001 | -41.27 | 0.000 | 0.007 | 0.011 |

**Performance in validation dataset (20% of practices) - Internal validation - Index AMI cases**

| AUROC (95% CI): 0.732 (0.722; 0.742) |  |
| --- | --- |
| 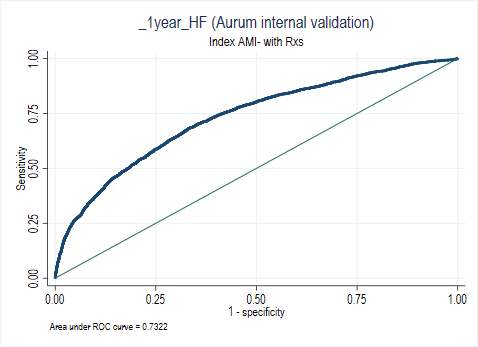 | 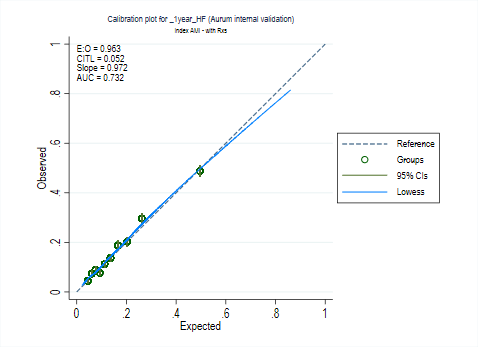 |
| 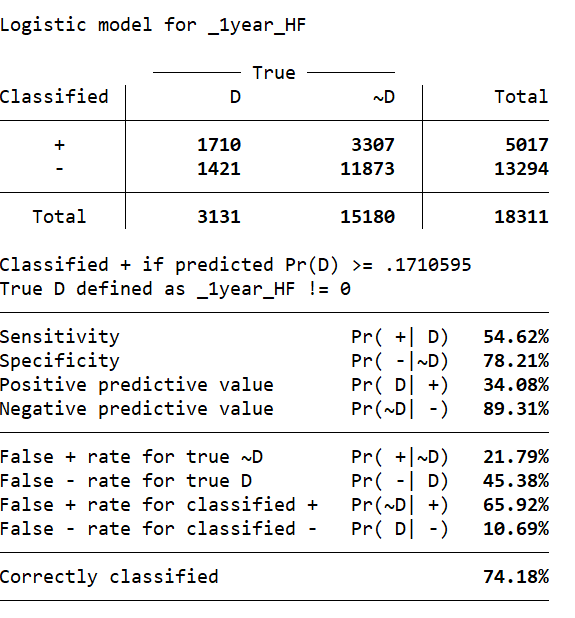 | % Correctly classified: 74.18 |

### 4) 1-year Recurrent MI

|  | | | | | | |
| --- | --- | --- | --- | --- | --- | --- |
| _1year_Recurrent_MI | Odds ratio | Std. err. | z | P>|z| | [95% conf. interval] | |
| age | 1.011 | 0.001 | 9.81 | 0.000 | 1.009 | 1.014 |
|  |  |  |  |  |  |  |
| gender |  |  |  |  |  |  |
| Female | 0.981 | 0.028 | -0.69 | 0.488 | 0.928 | 1.036 |
|  |  |  |  |  |  |  |
| IMD |  |  |  |  |  |  |
| Q2 | 1.016 | 0.036 | 0.45 | 0.652 | 0.948 | 1.090 |
| Q3 | 1.009 | 0.036 | 0.24 | 0.812 | 0.940 | 1.082 |
| Q4 | 0.986 | 0.036 | -0.37 | 0.710 | 0.918 | 1.060 |
| Q5-most deprived | 1.052 | 0.039 | 1.38 | 0.166 | 0.979 | 1.131 |
|  |  |  |  |  |  |  |
| Other than White |  |  |  |  |  |  |
| non-White | 1.047 | 0.050 | 0.97 | 0.332 | 0.954 | 1.149 |
|  |  |  |  |  |  |  |
| Drinker |  |  |  |  |  |  |
| drinker | 0.986 | 0.030 | -0.45 | 0.655 | 0.929 | 1.047 |
|  |  |  |  |  |  |  |
| Smoking_status |  |  |  |  |  |  |
| current smoker | 1.045 | 0.036 | 1.29 | 0.198 | 0.977 | 1.118 |
| ex-smoker | 0.957 | 0.030 | -1.43 | 0.153 | 0.900 | 1.017 |
|  |  |  |  |  |  |  |
| BMI | 0.995 | 0.002 | -2.40 | 0.017 | 0.990 | 0.999 |
| HT_b | 1.021 | 0.028 | 0.77 | 0.443 | 0.968 | 1.077 |
| Antihypertensives_b | 0.941 | 0.035 | -1.64 | 0.101 | 0.876 | 1.012 |
| Hyperlipidaemia_b | 0.991 | 0.031 | -0.29 | 0.776 | 0.933 | 1.053 |
| Lipid_reg_treatment_b | 0.914 | 0.028 | -2.96 | 0.003 | 0.861 | 0.970 |
|  |  |  |  |  |  |  |
| Diabetes_type_b |  |  |  |  |  |  |
| T1DM | 1.187 | 0.161 | 1.26 | 0.206 | 0.910 | 1.547 |
| T2DM | 1.022 | 0.062 | 0.35 | 0.725 | 0.907 | 1.150 |
| DM - nos | 1.321 | 0.132 | 2.78 | 0.005 | 1.085 | 1.607 |
|  |  |  |  |  |  |  |
| Antidiabetics_b | 1.170 | 0.075 | 2.46 | 0.014 | 1.032 | 1.327 |
| Diuretics_b | 1.138 | 0.036 | 4.04 | 0.000 | 1.069 | 1.211 |
| Anticoagulants_b | 0.974 | 0.048 | -0.55 | 0.585 | 0.885 | 1.071 |
| Antiplatelets_b | 1.075 | 0.032 | 2.42 | 0.015 | 1.014 | 1.141 |
| HF_b | 1.140 | 0.051 | 2.93 | 0.003 | 1.044 | 1.245 |
| AF_b | 0.981 | 0.042 | -0.45 | 0.652 | 0.902 | 1.067 |
| Heart_valve_dis_b | 1.300 | 0.121 | 2.81 | 0.005 | 1.083 | 1.562 |
| VT_VF_b | 0.598 | 0.131 | -2.35 | 0.019 | 0.389 | 0.919 |
| Cardiomyopathy_b | 0.803 | 0.138 | -1.28 | 0.201 | 0.573 | 1.124 |
| CV_procedures_b | 0.737 | 0.031 | -7.17 | 0.000 | 0.678 | 0.801 |
| TIA_stroke_b | 1.059 | 0.039 | 1.53 | 0.126 | 0.984 | 1.139 |
| PVD_b | 1.372 | 0.059 | 7.35 | 0.000 | 1.261 | 1.493 |
| CKD_b | 1.145 | 0.034 | 4.49 | 0.000 | 1.079 | 1.214 |
| Hypothyroidism_b | 1.059 | 0.044 | 1.38 | 0.167 | 0.976 | 1.148 |
| Liver_dis_b | 1.482 | 0.338 | 1.72 | 0.085 | 0.948 | 2.317 |
| Lupus_b | 1.172 | 0.224 | 0.83 | 0.406 | 0.805 | 1.706 |
| Erectile_dysfunction_b | 0.950 | 0.040 | -1.21 | 0.226 | 0.875 | 1.032 |
| Any_tumour_b | 1.064 | 0.033 | 2.00 | 0.046 | 1.001 | 1.130 |
| Menopause_b | 0.902 | 0.040 | -2.31 | 0.021 | 0.826 | 0.984 |
| Dementia_b | 1.131 | 0.078 | 1.79 | 0.074 | 0.988 | 1.295 |
| RA_b | 0.914 | 0.059 | -1.38 | 0.167 | 0.805 | 1.038 |
| _cons | 0.064 | 0.008 | -23.21 | 0.000 | 0.051 | 0.081 |

**Performance in validation dataset (20% of practices) - Internal validation - Index AMI cases**

| AUROC (95% CI): 0.570 (0.557; 0.583) |  |
| --- | --- |
| 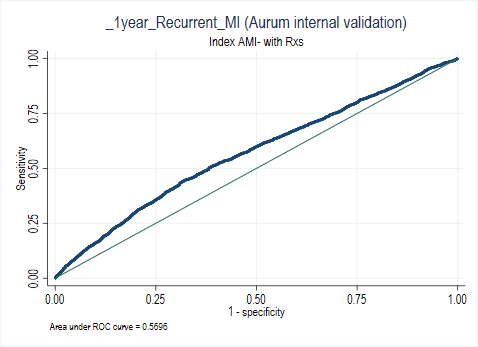 | 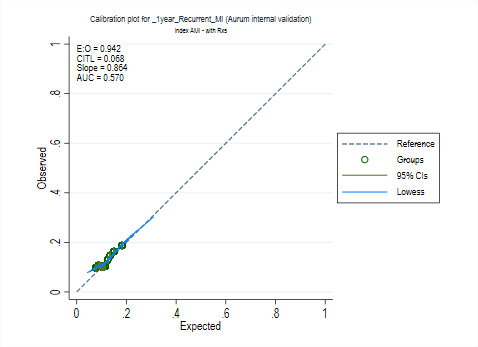 |
| 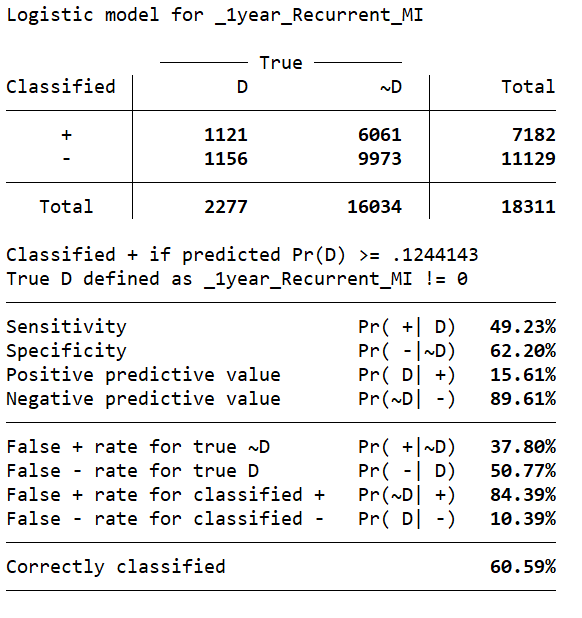 | % Correctly classified: 60.59 |

### 5) 1-year CV mortality

|  | | | | | | |
| --- | --- | --- | --- | --- | --- | --- |
| _1year_CV_death | Odds ratio | Std. err. | z | P>|z| | [95% conf. interval] | |
| age | 1.070 | 0.002 | 32.95 | 0.000 | 1.065 | 1.074 |
|  |  |  |  |  |  |  |
| gender |  |  |  |  |  |  |
| Female | 0.847 | 0.033 | -4.21 | 0.000 | 0.784 | 0.915 |
|  |  |  |  |  |  |  |
| IMD |  |  |  |  |  |  |
| Q2 | 0.973 | 0.052 | -0.51 | 0.607 | 0.876 | 1.080 |
| Q3 | 1.102 | 0.058 | 1.84 | 0.066 | 0.993 | 1.222 |
| Q4 | 1.175 | 0.062 | 3.04 | 0.002 | 1.059 | 1.304 |
| Q5-most deprived | 1.167 | 0.064 | 2.81 | 0.005 | 1.048 | 1.299 |
|  |  |  |  |  |  |  |
| Other than White |  |  |  |  |  |  |
| non-White | 0.776 | 0.066 | -2.98 | 0.003 | 0.657 | 0.917 |
|  |  |  |  |  |  |  |
| Drinker |  |  |  |  |  |  |
| drinker | 0.821 | 0.033 | -4.83 | 0.000 | 0.758 | 0.890 |
|  |  |  |  |  |  |  |
| Smoking_status |  |  |  |  |  |  |
| current smoker | 1.047 | 0.052 | 0.92 | 0.359 | 0.949 | 1.154 |
| ex-smoker | 0.785 | 0.033 | -5.67 | 0.000 | 0.722 | 0.854 |
|  |  |  |  |  |  |  |
| BMI | 0.981 | 0.003 | -5.65 | 0.000 | 0.974 | 0.987 |
| HT_b | 0.932 | 0.034 | -1.90 | 0.057 | 0.867 | 1.002 |
| Antihypertensives_b | 1.099 | 0.078 | 1.33 | 0.183 | 0.957 | 1.262 |
| Hyperlipidaemia_b | 0.920 | 0.041 | -1.87 | 0.062 | 0.844 | 1.004 |
| Lipid_reg_treatment_b | 0.871 | 0.037 | -3.22 | 0.001 | 0.800 | 0.947 |
|  |  |  |  |  |  |  |
| Diabetes_type_b |  |  |  |  |  |  |
| T1DM | 2.950 | 0.494 | 6.46 | 0.000 | 2.125 | 4.096 |
| T2DM | 1.064 | 0.086 | 0.76 | 0.445 | 0.907 | 1.247 |
| DM - nos | 1.416 | 0.190 | 2.60 | 0.009 | 1.089 | 1.841 |
|  |  |  |  |  |  |  |
| Antidiabetics_b | 1.439 | 0.123 | 4.25 | 0.000 | 1.217 | 1.701 |
| Diuretics_b | 1.591 | 0.080 | 9.19 | 0.000 | 1.441 | 1.756 |
| Anticoagulants_b | 1.099 | 0.063 | 1.65 | 0.099 | 0.982 | 1.229 |
| Antiplatelets_b | 1.237 | 0.054 | 4.87 | 0.000 | 1.135 | 1.347 |
| HF_b | 1.969 | 0.092 | 14.56 | 0.000 | 1.797 | 2.157 |
| AF_b | 1.294 | 0.062 | 5.35 | 0.000 | 1.177 | 1.422 |
| Heart_valve_dis_b | 1.554 | 0.159 | 4.31 | 0.000 | 1.272 | 1.899 |
| VT_VF_b | 1.133 | 0.244 | 0.58 | 0.561 | 0.743 | 1.728 |
| Cardiomyopathy_b | 1.066 | 0.210 | 0.32 | 0.746 | 0.724 | 1.569 |
| CV_procedures_b | 0.790 | 0.047 | -3.96 | 0.000 | 0.703 | 0.887 |
| TIA_stroke_b | 1.230 | 0.054 | 4.68 | 0.000 | 1.128 | 1.341 |
| PVD_b | 1.723 | 0.089 | 10.52 | 0.000 | 1.557 | 1.907 |
| CKD_b | 1.174 | 0.044 | 4.28 | 0.000 | 1.091 | 1.264 |
| Hypothyroidism_b | 1.020 | 0.055 | 0.37 | 0.715 | 0.917 | 1.134 |
| Liver_dis_b | 1.566 | 0.637 | 1.10 | 0.270 | 0.706 | 3.477 |
| Lupus_b | 1.567 | 0.425 | 1.65 | 0.098 | 0.920 | 2.667 |
| Erectile_dysfunction_b | 0.834 | 0.059 | -2.56 | 0.010 | 0.726 | 0.958 |
| Any_tumour_b | 1.054 | 0.043 | 1.30 | 0.193 | 0.974 | 1.141 |
| Menopause_b | 0.700 | 0.051 | -4.94 | 0.000 | 0.608 | 0.807 |
| Dementia_b | 1.528 | 0.109 | 5.94 | 0.000 | 1.328 | 1.757 |
| RA_b | 1.214 | 0.102 | 2.31 | 0.021 | 1.030 | 1.431 |
| _cons | 0.000 | 0.000 | -37.71 | 0.000 | 0.000 | 0.001 |

**Performance in validation dataset (20% of practices) - Internal validation - Index AMI cases**

| AUROC (95% CI): 0.807 (0.793; 0.821) |  |
| --- | --- |
| 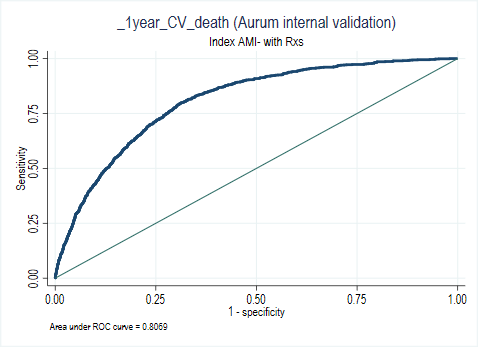 | 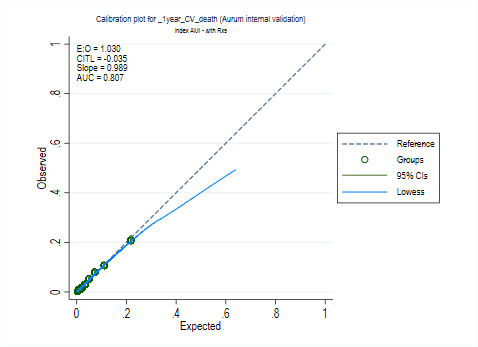 |
| 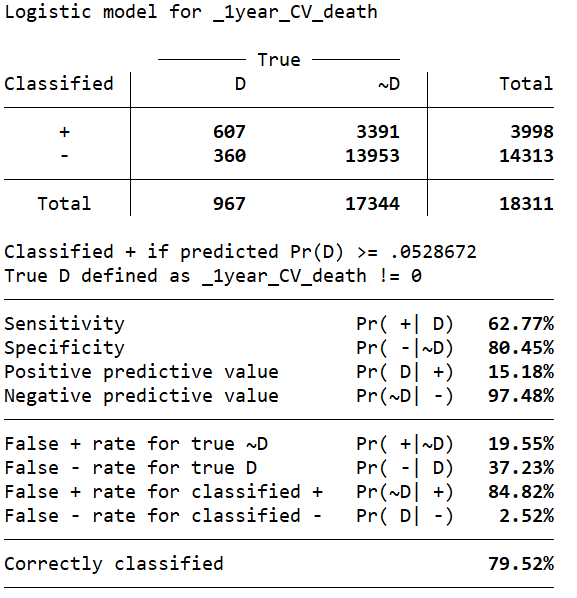 | % Correctly classified: 79.52 |

### 6) 1-year Composite 1 outcome (HF, stroke, recurrent MI, & all-cause mortality)

|  | | | | | | |
| --- | --- | --- | --- | --- | --- | --- |
| _1yr_Comp1_outcome | Odds ratio | Std. err. | z | P>|z| | [95% conf. interval] | |
| age | 1.034 | 0.001 | 39.21 | 0.000 | 1.032 | 1.036 |
|  |  |  |  |  |  |  |
| gender |  |  |  |  |  |  |
| Female | 0.977 | 0.020 | -1.11 | 0.267 | 0.939 | 1.018 |
|  |  |  |  |  |  |  |
| IMD |  |  |  |  |  |  |
| Q2 | 1.060 | 0.028 | 2.22 | 0.026 | 1.007 | 1.117 |
| Q3 | 1.070 | 0.028 | 2.55 | 0.011 | 1.016 | 1.127 |
| Q4 | 1.180 | 0.032 | 6.18 | 0.000 | 1.119 | 1.243 |
| Q5-most deprived | 1.251 | 0.034 | 8.25 | 0.000 | 1.186 | 1.320 |
|  |  |  |  |  |  |  |
| Other than White |  |  |  |  |  |  |
| non-White | 0.934 | 0.033 | -1.90 | 0.058 | 0.871 | 1.002 |
|  |  |  |  |  |  |  |
| Drinker |  |  |  |  |  |  |
| drinker | 0.924 | 0.021 | -3.53 | 0.000 | 0.884 | 0.965 |
|  |  |  |  |  |  |  |
| Smoking_status |  |  |  |  |  |  |
| current smoker | 1.112 | 0.028 | 4.18 | 0.000 | 1.058 | 1.169 |
| ex-smoker | 0.920 | 0.021 | -3.64 | 0.000 | 0.880 | 0.962 |
|  |  |  |  |  |  |  |
| BMI | 0.992 | 0.002 | -4.85 | 0.000 | 0.989 | 0.995 |
| HT_b | 0.961 | 0.019 | -1.99 | 0.046 | 0.924 | 0.999 |
| Antihypertensives_b | 0.980 | 0.026 | -0.74 | 0.459 | 0.930 | 1.033 |
| Hyperlipidaemia_b | 1.007 | 0.023 | 0.29 | 0.773 | 0.963 | 1.052 |
| Lipid_reg_treatment_b | 0.864 | 0.019 | -6.53 | 0.000 | 0.827 | 0.903 |
|  |  |  |  |  |  |  |
| Diabetes_type_b |  |  |  |  |  |  |
| T1DM | 1.689 | 0.172 | 5.16 | 0.000 | 1.384 | 2.061 |
| T2DM | 1.083 | 0.048 | 1.81 | 0.071 | 0.993 | 1.182 |
| DM - nos | 1.531 | 0.120 | 5.43 | 0.000 | 1.313 | 1.785 |
|  |  |  |  |  |  |  |
| Antidiabetics_b | 1.367 | 0.064 | 6.63 | 0.000 | 1.246 | 1.499 |
| Diuretics_b | 1.342 | 0.031 | 12.86 | 0.000 | 1.283 | 1.403 |
| Anticoagulants_b | 1.149 | 0.041 | 3.91 | 0.000 | 1.072 | 1.232 |
| Antiplatelets_b | 1.075 | 0.023 | 3.34 | 0.001 | 1.031 | 1.122 |
| HF_b | 3.108 | 0.113 | 31.30 | 0.000 | 2.895 | 3.337 |
| AF_b | 1.257 | 0.040 | 7.27 | 0.000 | 1.182 | 1.337 |
| Heart_valve_dis_b | 1.309 | 0.099 | 3.55 | 0.000 | 1.128 | 1.519 |
| VT_VF_b | 1.011 | 0.134 | 0.08 | 0.932 | 0.780 | 1.311 |
| Cardiomyopathy_b | 1.330 | 0.153 | 2.47 | 0.013 | 1.061 | 1.668 |
| CV_procedures_b | 0.711 | 0.021 | -11.38 | 0.000 | 0.670 | 0.754 |
| TIA_stroke_b | 2.045 | 0.056 | 25.94 | 0.000 | 1.937 | 2.158 |
| PVD_b | 1.544 | 0.052 | 12.91 | 0.000 | 1.445 | 1.649 |
| CKD_b | 1.199 | 0.027 | 8.18 | 0.000 | 1.148 | 1.253 |
| Hypothyroidism_b | 1.022 | 0.031 | 0.70 | 0.485 | 0.962 | 1.085 |
| Liver_dis_b | 1.382 | 0.253 | 1.77 | 0.077 | 0.966 | 1.978 |
| Lupus_b | 1.382 | 0.196 | 2.28 | 0.023 | 1.046 | 1.825 |
| Erectile_dysfunction_b | 0.962 | 0.029 | -1.27 | 0.205 | 0.906 | 1.021 |
| Any_tumour_b | 1.193 | 0.027 | 7.79 | 0.000 | 1.141 | 1.247 |
| Menopause_b | 0.793 | 0.026 | -7.13 | 0.000 | 0.744 | 0.845 |
| Dementia_b | 1.332 | 0.071 | 5.37 | 0.000 | 1.200 | 1.480 |
| RA_b | 1.077 | 0.049 | 1.62 | 0.105 | 0.985 | 1.177 |
| _cons | 0.038 | 0.003 | -37.00 | 0.000 | 0.032 | 0.046 |

**Performance in validation dataset (20% of practices) - Internal validation - Index AMI cases**

| AUROC (95% CI): 0.711 (0.704; 0.719) |  |
| --- | --- |
| 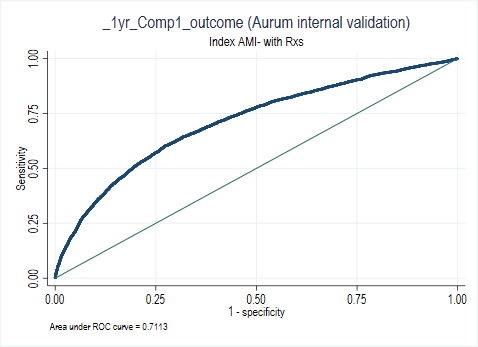 | 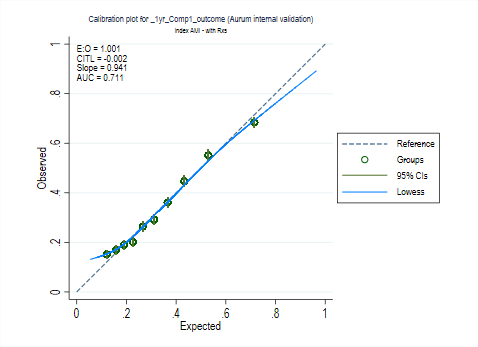 |
| 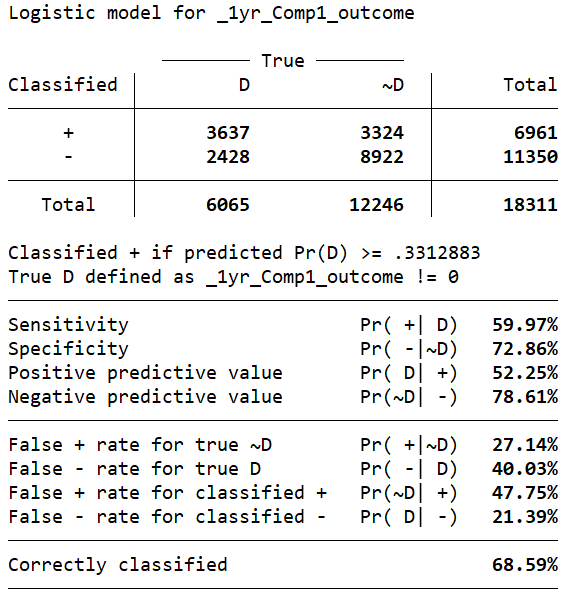 | % Correctly classified: 68.59 |

### 7) 1-year Composite 1 CV outcome (HF, stroke, recurrent MI, & CV mortality)

| _1yr_Comp1_CV_outcome | Odds ratio | Std. err. | z | P>|z| | [95% conf. interval] | |
| --- | --- | --- | --- | --- | --- | --- |
| age | 1.030 | 0.001 | 34.73 | 0.000 | 1.029 | 1.032 |
|  |  |  |  |  |  |  |
| gender |  |  |  |  |  |  |
| Female | 0.970 | 0.020 | -1.44 | 0.149 | 0.931 | 1.011 |
|  |  |  |  |  |  |  |
| IMD |  |  |  |  |  |  |
| Q2 | 1.020 | 0.027 | 0.76 | 0.448 | 0.968 | 1.075 |
| Q3 | 1.033 | 0.028 | 1.22 | 0.222 | 0.980 | 1.089 |
| Q4 | 1.134 | 0.031 | 4.65 | 0.000 | 1.075 | 1.195 |
| Q5-most deprived | 1.197 | 0.033 | 6.54 | 0.000 | 1.134 | 1.263 |
|  |  |  |  |  |  |  |
| Other than White |  |  |  |  |  |  |
| non-White | 0.996 | 0.036 | -0.12 | 0.905 | 0.928 | 1.068 |
|  |  |  |  |  |  |  |
| Drinker |  |  |  |  |  |  |
| drinker | 0.929 | 0.021 | -3.26 | 0.001 | 0.888 | 0.971 |
|  |  |  |  |  |  |  |
| Smoking_status |  |  |  |  |  |  |
| current smoker | 1.076 | 0.028 | 2.86 | 0.004 | 1.023 | 1.132 |
| ex-smoker | 0.937 | 0.022 | -2.81 | 0.005 | 0.896 | 0.981 |
|  |  |  |  |  |  |  |
| BMI | 0.997 | 0.002 | -1.74 | 0.083 | 0.994 | 1.000 |
| HT_b | 1.014 | 0.020 | 0.70 | 0.485 | 0.975 | 1.055 |
| Antihypertensives_b | 0.964 | 0.026 | -1.32 | 0.187 | 0.914 | 1.018 |
| Hyperlipidaemia_b | 0.997 | 0.023 | -0.15 | 0.879 | 0.953 | 1.042 |
| Lipid_reg_treatment_b | 0.896 | 0.020 | -4.85 | 0.000 | 0.857 | 0.937 |
|  |  |  |  |  |  |  |
| Diabetes_type_b |  |  |  |  |  |  |
| T1DM | 1.504 | 0.154 | 4.00 | 0.000 | 1.231 | 1.837 |
| T2DM | 1.035 | 0.046 | 0.77 | 0.439 | 0.948 | 1.130 |
| DM - nos | 1.386 | 0.109 | 4.16 | 0.000 | 1.189 | 1.617 |
|  |  |  |  |  |  |  |
| Antidiabetics_b | 1.416 | 0.067 | 7.33 | 0.000 | 1.291 | 1.554 |
| Diuretics_b | 1.317 | 0.030 | 11.89 | 0.000 | 1.259 | 1.378 |
| Anticoagulants_b | 1.102 | 0.039 | 2.73 | 0.006 | 1.028 | 1.182 |
| Antiplatelets_b | 1.056 | 0.023 | 2.47 | 0.013 | 1.011 | 1.103 |
| HF_b | 3.180 | 0.113 | 32.69 | 0.000 | 2.967 | 3.408 |
| AF_b | 1.297 | 0.041 | 8.28 | 0.000 | 1.220 | 1.379 |
| Heart_valve_dis_b | 1.223 | 0.092 | 2.67 | 0.008 | 1.055 | 1.417 |
| VT_VF_b | 0.934 | 0.125 | -0.51 | 0.610 | 0.719 | 1.214 |
| Cardiomyopathy_b | 1.573 | 0.181 | 3.94 | 0.000 | 1.256 | 1.971 |
| CV_procedures_b | 0.739 | 0.022 | -10.02 | 0.000 | 0.696 | 0.784 |
| TIA_stroke_b | 2.125 | 0.058 | 27.56 | 0.000 | 2.014 | 2.242 |
| PVD_b | 1.504 | 0.050 | 12.18 | 0.000 | 1.409 | 1.607 |
| CKD_b | 1.185 | 0.026 | 7.62 | 0.000 | 1.135 | 1.238 |
| Hypothyroidism_b | 1.000 | 0.031 | 0.02 | 0.987 | 0.941 | 1.063 |
| Liver_dis_b | 1.283 | 0.240 | 1.33 | 0.182 | 0.890 | 1.850 |
| Lupus_b | 1.364 | 0.195 | 2.16 | 0.030 | 1.030 | 1.805 |
| Erectile_dysfunction_b | 0.976 | 0.030 | -0.78 | 0.435 | 0.919 | 1.037 |
| Any_tumour_b | 1.070 | 0.025 | 2.95 | 0.003 | 1.023 | 1.120 |
| Menopause_b | 0.786 | 0.026 | -7.24 | 0.000 | 0.737 | 0.839 |
| Dementia_b | 1.100 | 0.059 | 1.79 | 0.074 | 0.991 | 1.221 |
| RA_b | 1.038 | 0.048 | 0.80 | 0.426 | 0.948 | 1.136 |
| _cons | 0.040 | 0.004 | -36.24 | 0.000 | 0.033 | 0.047 |

**Performance in validation dataset (20% of practices) - Internal validation - Index AMI cases**

| AUROC (95% CI): 0.700 (0.692; 0.709) |  |
| --- | --- |
| 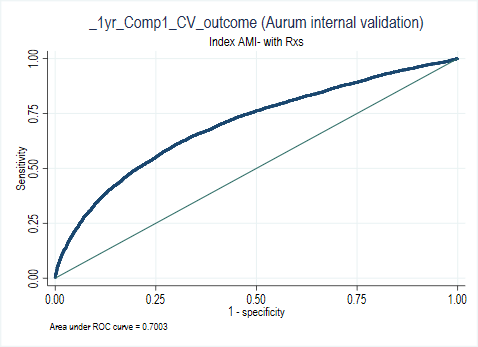 | 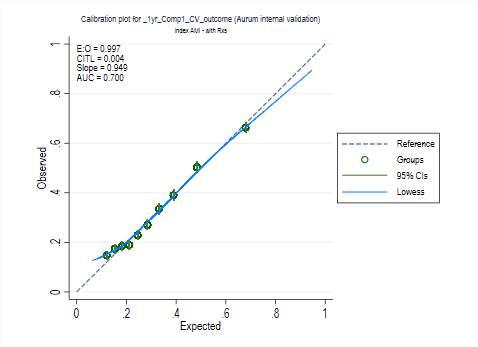 |
| 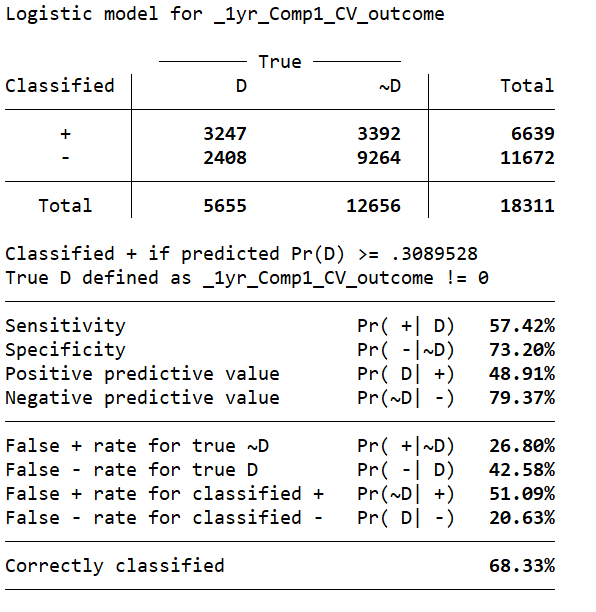 | % Correctly classified: 68.33 |

### 8) 1-year Composite 2 outcome (HF, stroke, & all-cause mortality)

|  | | | | | | |
| --- | --- | --- | --- | --- | --- | --- |
| _1yr_Comp2_outcome | Odds ratio | Std. err. | z | P>|z| | [95% conf. interval] | |
| age | 1.047 | 0.001 | 47.37 | 0.000 | 1.045 | 1.049 |
|  |  |  |  |  |  |  |
| gender |  |  |  |  |  |  |
| Female | 0.932 | 0.021 | -3.11 | 0.002 | 0.892 | 0.974 |
|  |  |  |  |  |  |  |
| IMD |  |  |  |  |  |  |
| Q2 | 1.086 | 0.032 | 2.82 | 0.005 | 1.026 | 1.150 |
| Q3 | 1.137 | 0.033 | 4.36 | 0.000 | 1.073 | 1.204 |
| Q4 | 1.264 | 0.037 | 7.93 | 0.000 | 1.193 | 1.340 |
| Q5-most deprived | 1.395 | 0.042 | 11.10 | 0.000 | 1.316 | 1.480 |
|  |  |  |  |  |  |  |
| Other than White |  |  |  |  |  |  |
| non-White | 0.922 | 0.037 | -2.00 | 0.045 | 0.852 | 0.998 |
|  |  |  |  |  |  |  |
| Drinker |  |  |  |  |  |  |
| drinker | 0.879 | 0.021 | -5.34 | 0.000 | 0.838 | 0.922 |
|  |  |  |  |  |  |  |
| Smoking_status |  |  |  |  |  |  |
| current smoker | 1.185 | 0.033 | 6.04 | 0.000 | 1.122 | 1.252 |
| ex-smoker | 0.944 | 0.024 | -2.31 | 0.021 | 0.899 | 0.991 |
|  |  |  |  |  |  |  |
| BMI | 0.989 | 0.002 | -6.25 | 0.000 | 0.985 | 0.992 |
| HT_b | 0.947 | 0.020 | -2.53 | 0.011 | 0.908 | 0.988 |
| Antihypertensives_b | 1.040 | 0.032 | 1.27 | 0.205 | 0.979 | 1.105 |
| Hyperlipidaemia_b | 1.002 | 0.025 | 0.08 | 0.934 | 0.955 | 1.051 |
| Lipid_reg_treatment_b | 0.879 | 0.022 | -5.26 | 0.000 | 0.838 | 0.922 |
|  |  |  |  |  |  |  |
| Diabetes_type_b |  |  |  |  |  |  |
| T1DM | 1.982 | 0.215 | 6.32 | 0.000 | 1.603 | 2.450 |
| T2DM | 1.071 | 0.051 | 1.44 | 0.151 | 0.975 | 1.175 |
| DM - nos | 1.542 | 0.127 | 5.24 | 0.000 | 1.311 | 1.813 |
|  |  |  |  |  |  |  |
| Antidiabetics_b | 1.463 | 0.074 | 7.53 | 0.000 | 1.325 | 1.615 |
| Diuretics_b | 1.418 | 0.036 | 13.95 | 0.000 | 1.350 | 1.490 |
| Anticoagulants_b | 1.265 | 0.047 | 6.35 | 0.000 | 1.176 | 1.360 |
| Antiplatelets_b | 1.074 | 0.026 | 3.02 | 0.003 | 1.025 | 1.126 |
| HF_b | 3.674 | 0.134 | 35.67 | 0.000 | 3.421 | 3.946 |
| AF_b | 1.310 | 0.043 | 8.31 | 0.000 | 1.230 | 1.397 |
| Heart_valve_dis_b | 1.329 | 0.104 | 3.65 | 0.000 | 1.141 | 1.549 |
| VT_VF_b | 0.912 | 0.129 | -0.65 | 0.518 | 0.691 | 1.205 |
| Cardiomyopathy_b | 1.679 | 0.200 | 4.34 | 0.000 | 1.329 | 2.121 |
| CV_procedures_b | 0.767 | 0.025 | -8.18 | 0.000 | 0.720 | 0.817 |
| TIA_stroke_b | 2.268 | 0.064 | 28.98 | 0.000 | 2.146 | 2.397 |
| PVD_b | 1.571 | 0.055 | 12.97 | 0.000 | 1.467 | 1.682 |
| CKD_b | 1.206 | 0.028 | 8.07 | 0.000 | 1.152 | 1.262 |
| Hypothyroidism_b | 1.012 | 0.033 | 0.36 | 0.720 | 0.949 | 1.079 |
| Liver_dis_b | 1.125 | 0.243 | 0.54 | 0.586 | 0.737 | 1.717 |
| Lupus_b | 1.324 | 0.205 | 1.81 | 0.071 | 0.977 | 1.794 |
| Erectile_dysfunction_b | 0.926 | 0.032 | -2.24 | 0.025 | 0.866 | 0.990 |
| Any_tumour_b | 1.264 | 0.030 | 9.78 | 0.000 | 1.206 | 1.324 |
| Menopause_b | 0.809 | 0.029 | -5.92 | 0.000 | 0.754 | 0.868 |
| Dementia_b | 1.381 | 0.075 | 5.95 | 0.000 | 1.241 | 1.536 |
| RA_b | 1.154 | 0.056 | 2.93 | 0.003 | 1.049 | 1.270 |
| _cons | 0.010 | 0.001 | -46.23 | 0.000 | 0.008 | 0.012 |

**Performance in validation dataset (20% of practices) - Internal validation - Index AMI cases**

| AUROC (95% CI): 0.763 (0.756; 0.771) |  |
| --- | --- |
| 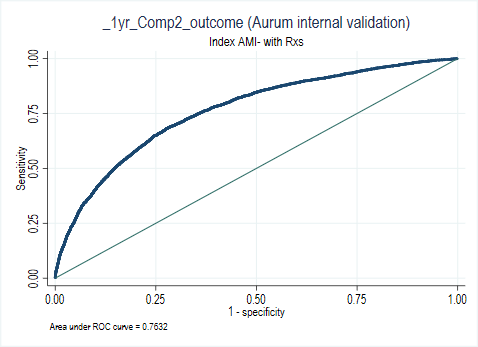 | 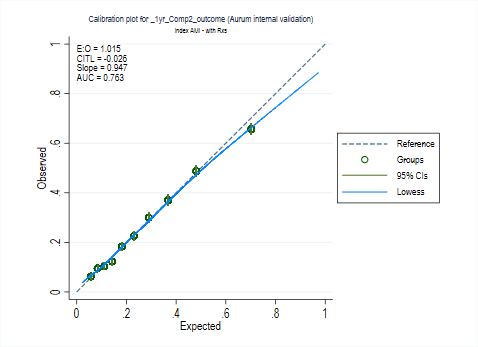 |
| 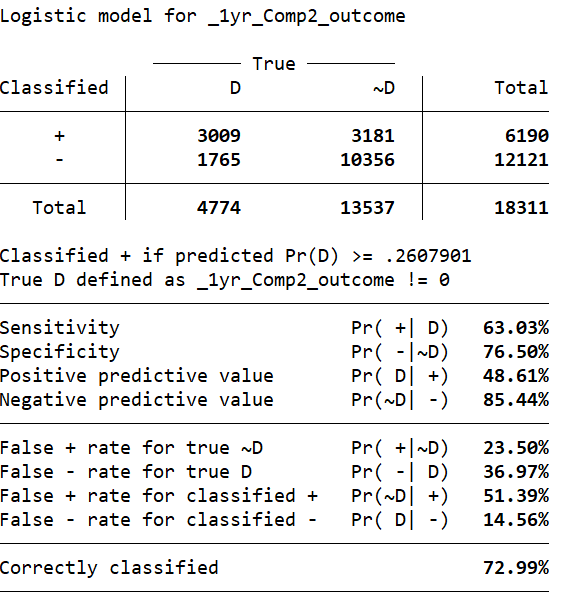 | % Correctly classified: 72.99 |

### 9) 1-year Composite 2 CV outcome (HF, stroke, & CV mortality)

|  | | | | | | |
| --- | --- | --- | --- | --- | --- | --- |
| _1yr_Comp2_CV_outcome | Odds ratio | Std. err. | z | P>|z| | [95% conf. interval] | |
| age | 1.043 | 0.001 | 42.57 | 0.000 | 1.041 | 1.046 |
|  |  |  |  |  |  |  |
| gender |  |  |  |  |  |  |
| Female | 0.945 | 0.022 | -2.44 | 0.015 | 0.904 | 0.989 |
|  |  |  |  |  |  |  |
| IMD |  |  |  |  |  |  |
| Q2 | 1.056 | 0.032 | 1.80 | 0.071 | 0.995 | 1.120 |
| Q3 | 1.092 | 0.033 | 2.92 | 0.004 | 1.029 | 1.159 |
| Q4 | 1.218 | 0.037 | 6.50 | 0.000 | 1.148 | 1.293 |
| Q5-most deprived | 1.334 | 0.041 | 9.37 | 0.000 | 1.256 | 1.417 |
|  |  |  |  |  |  |  |
| Other than White |  |  |  |  |  |  |
| non-White | 0.967 | 0.040 | -0.82 | 0.414 | 0.892 | 1.048 |
|  |  |  |  |  |  |  |
| Drinker |  |  |  |  |  |  |
| drinker | 0.925 | 0.023 | -3.13 | 0.002 | 0.882 | 0.972 |
|  |  |  |  |  |  |  |
| Smoking_status |  |  |  |  |  |  |
| current smoker | 1.127 | 0.033 | 4.13 | 0.000 | 1.065 | 1.192 |
| ex-smoker | 0.937 | 0.024 | -2.56 | 0.011 | 0.891 | 0.985 |
|  |  |  |  |  |  |  |
| BMI | 0.995 | 0.002 | -2.70 | 0.007 | 0.991 | 0.999 |
| HT_b | 0.994 | 0.022 | -0.29 | 0.771 | 0.952 | 1.037 |
| Antihypertensives_b | 1.013 | 0.033 | 0.40 | 0.688 | 0.951 | 1.080 |
| Hyperlipidaemia_b | 1.013 | 0.025 | 0.51 | 0.609 | 0.964 | 1.064 |
| Lipid_reg_treatment_b | 0.893 | 0.022 | -4.51 | 0.000 | 0.850 | 0.938 |
|  |  |  |  |  |  |  |
| Diabetes_type_b |  |  |  |  |  |  |
| T1DM | 1.878 | 0.206 | 5.73 | 0.000 | 1.514 | 2.329 |
| T2DM | 1.062 | 0.052 | 1.25 | 0.212 | 0.966 | 1.168 |
| DM - nos | 1.467 | 0.123 | 4.58 | 0.000 | 1.245 | 1.728 |
|  |  |  |  |  |  |  |
| Antidiabetics_b | 1.452 | 0.075 | 7.26 | 0.000 | 1.313 | 1.606 |
| Diuretics_b | 1.412 | 0.037 | 13.32 | 0.000 | 1.342 | 1.486 |
| Anticoagulants_b | 1.222 | 0.046 | 5.36 | 0.000 | 1.135 | 1.314 |
| Antiplatelets_b | 1.074 | 0.026 | 2.93 | 0.003 | 1.024 | 1.127 |
| HF_b | 3.761 | 0.134 | 37.24 | 0.000 | 3.508 | 4.033 |
| AF_b | 1.354 | 0.044 | 9.27 | 0.000 | 1.270 | 1.444 |
| Heart_valve_dis_b | 1.315 | 0.102 | 3.52 | 0.000 | 1.129 | 1.531 |
| VT_VF_b | 0.986 | 0.141 | -0.10 | 0.921 | 0.746 | 1.304 |
| Cardiomyopathy_b | 1.834 | 0.218 | 5.11 | 0.000 | 1.454 | 2.314 |
| CV_procedures_b | 0.804 | 0.027 | -6.60 | 0.000 | 0.753 | 0.858 |
| TIA_stroke_b | 2.415 | 0.068 | 31.23 | 0.000 | 2.285 | 2.552 |
| PVD_b | 1.572 | 0.055 | 12.89 | 0.000 | 1.467 | 1.684 |
| CKD_b | 1.173 | 0.028 | 6.75 | 0.000 | 1.120 | 1.229 |
| Hypothyroidism_b | 0.961 | 0.032 | -1.18 | 0.236 | 0.899 | 1.026 |
| Liver_dis_b | 0.881 | 0.208 | -0.54 | 0.591 | 0.554 | 1.400 |
| Lupus_b | 1.313 | 0.209 | 1.71 | 0.088 | 0.960 | 1.794 |
| Erectile_dysfunction_b | 0.932 | 0.033 | -2.01 | 0.045 | 0.870 | 0.998 |
| Any_tumour_b | 1.073 | 0.027 | 2.85 | 0.004 | 1.022 | 1.127 |
| Menopause_b | 0.756 | 0.028 | -7.48 | 0.000 | 0.702 | 0.813 |
| Dementia_b | 1.080 | 0.060 | 1.39 | 0.163 | 0.969 | 1.203 |
| RA_b | 1.133 | 0.057 | 2.49 | 0.013 | 1.027 | 1.250 |
| _cons | 0.009 | 0.001 | -45.64 | 0.000 | 0.007 | 0.011 |

**Performance in validation dataset (20% of practices) - Internal validation - Index AMI cases**

| AUROC (95% CI): 0.758 (0.750; 0.766) |  |
| --- | --- |
| 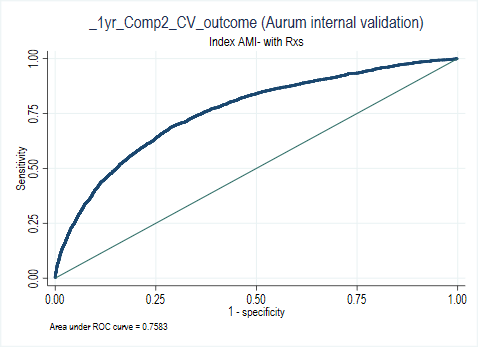 | 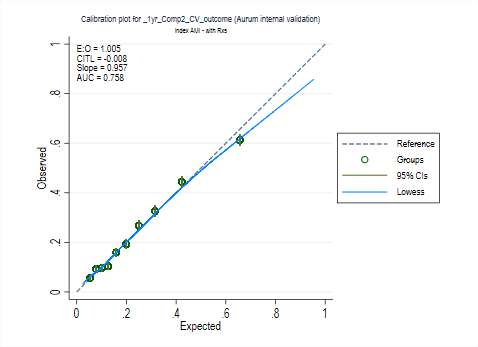 |
| 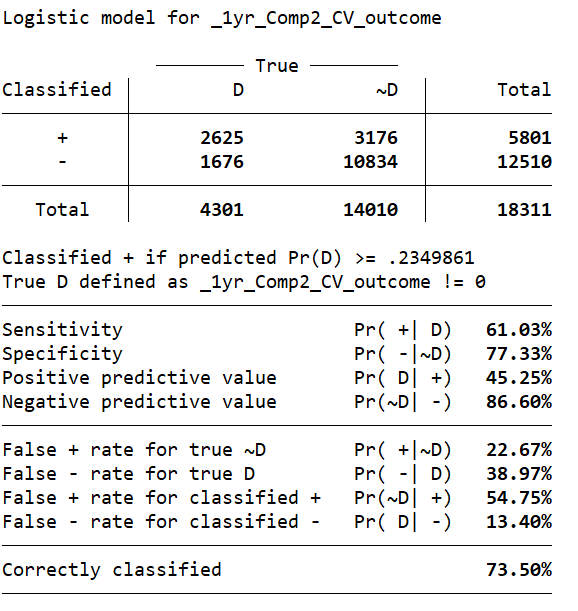 | % Correctly classified: 73.50 |

## External validation (CPRD GOLD) - with therapies

### 1) 1-year all-cause mortality

|  | | | | | | |
| --- | --- | --- | --- | --- | --- | --- |
| _1year_All_cause_death | Odds ratio | Std. err. | z | P>|z| | [95% conf. interval] | |
| age | 1.068 | 0.002 | 44.26 | 0.000 | 1.065 | 1.072 |
|  |  |  |  |  |  |  |
| gender |  |  |  |  |  |  |
| Female | 0.901 | 0.027 | -3.47 | 0.001 | 0.850 | 0.956 |
|  |  |  |  |  |  |  |
| IMD |  |  |  |  |  |  |
| Q2 | 1.069 | 0.043 | 1.67 | 0.095 | 0.988 | 1.157 |
| Q3 | 1.144 | 0.046 | 3.35 | 0.001 | 1.057 | 1.239 |
| Q4 | 1.252 | 0.051 | 5.54 | 0.000 | 1.157 | 1.356 |
| Q5-most deprived | 1.298 | 0.054 | 6.28 | 0.000 | 1.196 | 1.408 |
|  |  |  |  |  |  |  |
| Other than White |  |  |  |  |  |  |
| non-White | 0.843 | 0.052 | -2.74 | 0.006 | 0.746 | 0.952 |
|  |  |  |  |  |  |  |
| Drinker |  |  |  |  |  |  |
| drinker | 0.850 | 0.027 | -5.13 | 0.000 | 0.799 | 0.905 |
|  |  |  |  |  |  |  |
| Smoking_status |  |  |  |  |  |  |
| current smoker | 1.231 | 0.047 | 5.46 | 0.000 | 1.142 | 1.326 |
| ex-smoker | 0.867 | 0.029 | -4.30 | 0.000 | 0.813 | 0.926 |
|  |  |  |  |  |  |  |
| BMI | 0.968 | 0.003 | -12.40 | 0.000 | 0.962 | 0.973 |
| HT_b | 0.865 | 0.024 | -5.14 | 0.000 | 0.818 | 0.914 |
| Antihypertensives_b | 1.173 | 0.057 | 3.26 | 0.001 | 1.066 | 1.291 |
| Hyperlipidaemia_b | 0.909 | 0.031 | -2.80 | 0.005 | 0.851 | 0.972 |
| Lipid_reg_treatment_b | 0.840 | 0.028 | -5.33 | 0.000 | 0.787 | 0.895 |
|  |  |  |  |  |  |  |
| Diabetes_type_b |  |  |  |  |  |  |
| T1DM | 2.383 | 0.336 | 6.16 | 0.000 | 1.808 | 3.142 |
| T2DM | 1.103 | 0.069 | 1.58 | 0.114 | 0.977 | 1.246 |
| DM - nos | 1.667 | 0.173 | 4.93 | 0.000 | 1.360 | 2.042 |
|  |  |  |  |  |  |  |
| Antidiabetics_b | 1.371 | 0.090 | 4.78 | 0.000 | 1.204 | 1.560 |
| Diuretics_b | 1.505 | 0.055 | 11.26 | 0.000 | 1.402 | 1.616 |
| Anticoagulants_b | 1.204 | 0.054 | 4.11 | 0.000 | 1.102 | 1.315 |
| Antiplatelets_b | 1.158 | 0.038 | 4.50 | 0.000 | 1.086 | 1.234 |
| HF_b | 1.756 | 0.069 | 14.36 | 0.000 | 1.626 | 1.896 |
| AF_b | 1.143 | 0.045 | 3.43 | 0.001 | 1.059 | 1.234 |
| Heart_valve_dis_b | 1.368 | 0.120 | 3.58 | 0.000 | 1.153 | 1.623 |
| VT_VF_b | 0.748 | 0.144 | -1.51 | 0.131 | 0.513 | 1.090 |
| Cardiomyopathy_b | 1.147 | 0.178 | 0.88 | 0.377 | 0.846 | 1.556 |
| CV_procedures_b | 0.766 | 0.035 | -5.83 | 0.000 | 0.700 | 0.838 |
| TIA_stroke_b | 1.301 | 0.045 | 7.56 | 0.000 | 1.215 | 1.393 |
| PVD_b | 1.626 | 0.068 | 11.62 | 0.000 | 1.498 | 1.765 |
| CKD_b | 1.257 | 0.036 | 7.88 | 0.000 | 1.188 | 1.331 |
| Hypothyroidism_b | 1.015 | 0.043 | 0.36 | 0.715 | 0.935 | 1.102 |
| Liver_dis_b | 1.688 | 0.499 | 1.77 | 0.077 | 0.945 | 3.013 |
| Lupus_b | 1.412 | 0.296 | 1.65 | 0.099 | 0.937 | 2.129 |
| Erectile_dysfunction_b | 0.815 | 0.043 | -3.87 | 0.000 | 0.735 | 0.904 |
| Any_tumour_b | 1.481 | 0.044 | 13.25 | 0.000 | 1.398 | 1.570 |
| Menopause_b | 0.806 | 0.041 | -4.25 | 0.000 | 0.729 | 0.890 |
| Dementia_b | 1.768 | 0.102 | 9.90 | 0.000 | 1.579 | 1.979 |
| RA_b | 1.243 | 0.079 | 3.42 | 0.001 | 1.098 | 1.408 |
| _cons | 0.001 | 0.000 | -44.35 | 0.000 | 0.001 | 0.002 |

**Performance in validation dataset (CPRD GOLD) - External validation - Index AMI cases**

| AUROC (95% CI): 0.800 (0.790; 0.810) |  |
| --- | --- |
| 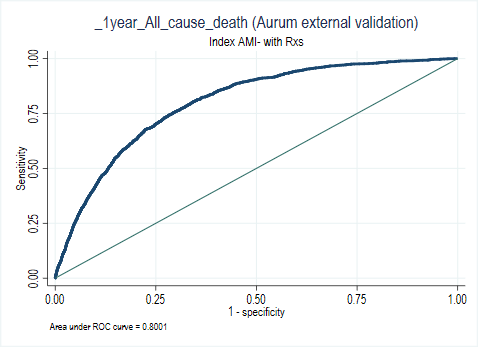 | 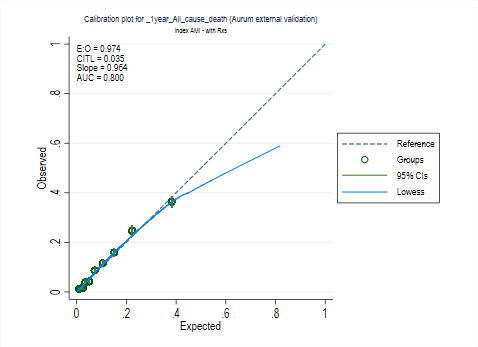 |
| 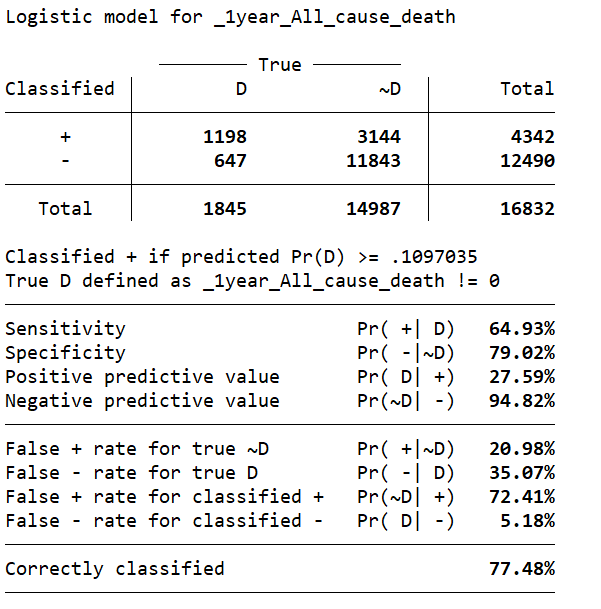 | % Correctly classified: 77.48 |

### 2) 1-year Stroke

|  | | | | | | |
| --- | --- | --- | --- | --- | --- | --- |
| _1year_Stroke | Odds ratio | Std. err. | z | P>|z| | [95% conf. interval] | |
| age | 1.036 | 0.002 | 20.11 | 0.000 | 1.032 | 1.039 |
|  |  |  |  |  |  |  |
| gender |  |  |  |  |  |  |
| Female | 1.036 | 0.039 | 0.96 | 0.339 | 0.963 | 1.115 |
|  |  |  |  |  |  |  |
| IMD |  |  |  |  |  |  |
| Q2 | 0.956 | 0.047 | -0.91 | 0.365 | 0.868 | 1.054 |
| Q3 | 1.063 | 0.052 | 1.24 | 0.216 | 0.965 | 1.171 |
| Q4 | 1.125 | 0.055 | 2.38 | 0.017 | 1.021 | 1.239 |
| Q5-most deprived | 1.089 | 0.055 | 1.69 | 0.092 | 0.986 | 1.203 |
|  |  |  |  |  |  |  |
| Other than White |  |  |  |  |  |  |
| non-White | 0.981 | 0.068 | -0.27 | 0.784 | 0.857 | 1.124 |
|  |  |  |  |  |  |  |
| Drinker |  |  |  |  |  |  |
| drinker | 0.867 | 0.033 | -3.71 | 0.000 | 0.804 | 0.935 |
|  |  |  |  |  |  |  |
| Smoking_status |  |  |  |  |  |  |
| current smoker | 1.132 | 0.053 | 2.63 | 0.009 | 1.032 | 1.242 |
| ex-smoker | 0.932 | 0.039 | -1.70 | 0.090 | 0.860 | 1.011 |
|  |  |  |  |  |  |  |
| BMI | 0.988 | 0.003 | -3.77 | 0.000 | 0.982 | 0.994 |
| HT_b | 1.064 | 0.037 | 1.79 | 0.074 | 0.994 | 1.139 |
| Antihypertensives_b | 1.343 | 0.083 | 4.78 | 0.000 | 1.190 | 1.517 |
| Hyperlipidaemia_b | 1.128 | 0.043 | 3.18 | 0.001 | 1.048 | 1.216 |
| Lipid_reg_treatment_b | 1.001 | 0.042 | 0.03 | 0.978 | 0.923 | 1.086 |
|  |  |  |  |  |  |  |
| Diabetes_type_b |  |  |  |  |  |  |
| T1DM | 1.749 | 0.286 | 3.42 | 0.001 | 1.269 | 2.410 |
| T2DM | 1.016 | 0.076 | 0.21 | 0.832 | 0.877 | 1.177 |
| DM - nos | 1.341 | 0.164 | 2.41 | 0.016 | 1.056 | 1.704 |
|  |  |  |  |  |  |  |
| Antidiabetics_b | 1.304 | 0.103 | 3.35 | 0.001 | 1.117 | 1.523 |
| Diuretics_b | 1.148 | 0.049 | 3.27 | 0.001 | 1.057 | 1.248 |
| Anticoagulants_b | 1.035 | 0.056 | 0.63 | 0.531 | 0.930 | 1.151 |
| Antiplatelets_b | 1.220 | 0.052 | 4.63 | 0.000 | 1.121 | 1.326 |
| HF_b | 1.111 | 0.056 | 2.09 | 0.036 | 1.007 | 1.227 |
| AF_b | 1.390 | 0.064 | 7.11 | 0.000 | 1.270 | 1.523 |
| Heart_valve_dis_b | 1.123 | 0.120 | 1.08 | 0.280 | 0.910 | 1.384 |
| VT_VF_b | 0.886 | 0.190 | -0.57 | 0.572 | 0.583 | 1.348 |
| Cardiomyopathy_b | 1.381 | 0.246 | 1.81 | 0.070 | 0.974 | 1.958 |
| CV_procedures_b | 0.786 | 0.041 | -4.59 | 0.000 | 0.709 | 0.871 |
| TIA_stroke_b | 6.237 | 0.219 | 52.19 | 0.000 | 5.822 | 6.680 |
| PVD_b | 1.471 | 0.073 | 7.83 | 0.000 | 1.336 | 1.620 |
| CKD_b | 1.105 | 0.040 | 2.78 | 0.005 | 1.030 | 1.185 |
| Hypothyroidism_b | 1.023 | 0.052 | 0.45 | 0.649 | 0.926 | 1.131 |
| Liver_dis_b | 0.714 | 0.315 | -0.76 | 0.445 | 0.300 | 1.696 |
| Lupus_b | 1.188 | 0.294 | 0.70 | 0.486 | 0.732 | 1.930 |
| Erectile_dysfunction_b | 1.003 | 0.059 | 0.05 | 0.962 | 0.894 | 1.125 |
| Any_tumour_b | 1.008 | 0.039 | 0.20 | 0.843 | 0.933 | 1.088 |
| Menopause_b | 0.942 | 0.055 | -1.03 | 0.305 | 0.840 | 1.056 |
| Dementia_b | 1.326 | 0.098 | 3.84 | 0.000 | 1.148 | 1.532 |
| RA_b | 1.042 | 0.084 | 0.51 | 0.610 | 0.890 | 1.221 |
| _cons | 0.003 | 0.000 | -32.91 | 0.000 | 0.002 | 0.004 |

**Performance in validation dataset (CPRD GOLD) - External validation - Index AMI cases**

| AUROC (95% CI): 0.777 (0.763; 0.791) |  |
| --- | --- |
| 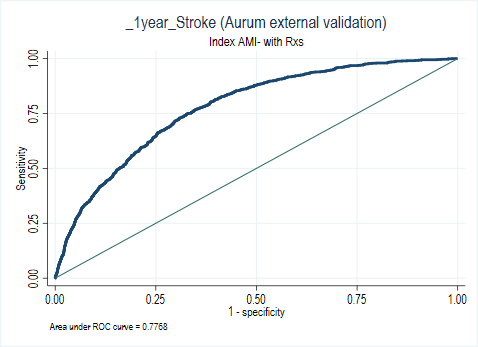 | 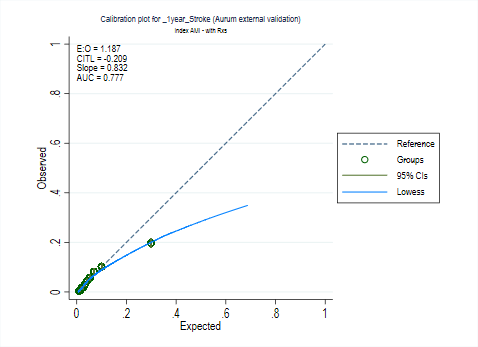 |
| 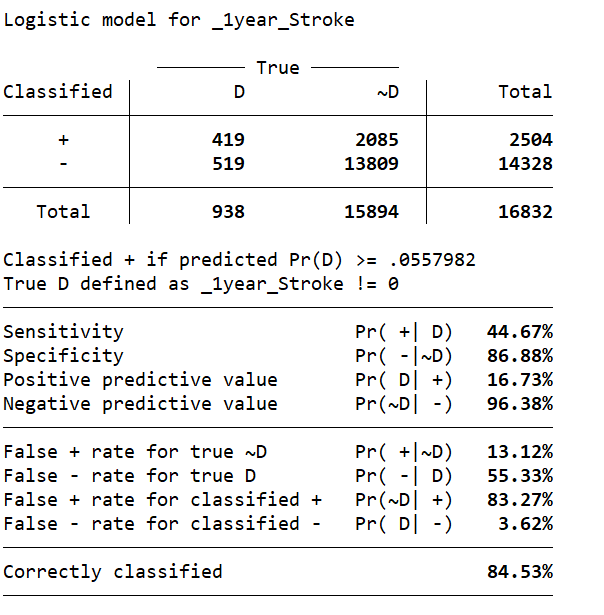 | % Correctly classified: 84.53 |

### 3) 1-year heart failure (HF)

|  | | | | | | |
| --- | --- | --- | --- | --- | --- | --- |
| _1year_HF | Odds ratio | Std. err. | z | P>|z| | [95% conf. interval] | |
| age | 1.038 | 0.001 | 33.25 | 0.000 | 1.036 | 1.040 |
|  |  |  |  |  |  |  |
| gender |  |  |  |  |  |  |
| Female | 0.897 | 0.023 | -4.21 | 0.000 | 0.853 | 0.944 |
|  |  |  |  |  |  |  |
| IMD |  |  |  |  |  |  |
| Q2 | 1.099 | 0.037 | 2.81 | 0.005 | 1.029 | 1.174 |
| Q3 | 1.041 | 0.035 | 1.19 | 0.233 | 0.974 | 1.113 |
| Q4 | 1.177 | 0.040 | 4.80 | 0.000 | 1.101 | 1.258 |
| Q5-most deprived | 1.322 | 0.045 | 8.17 | 0.000 | 1.237 | 1.414 |
|  |  |  |  |  |  |  |
| Other than White |  |  |  |  |  |  |
| non-White | 1.004 | 0.046 | 0.08 | 0.939 | 0.918 | 1.097 |
|  |  |  |  |  |  |  |
| Drinker |  |  |  |  |  |  |
| drinker | 0.870 | 0.024 | -5.11 | 0.000 | 0.825 | 0.918 |
|  |  |  |  |  |  |  |
| Smoking_status |  |  |  |  |  |  |
| current smoker | 1.115 | 0.036 | 3.39 | 0.001 | 1.047 | 1.188 |
| ex-smoker | 0.962 | 0.027 | -1.37 | 0.171 | 0.910 | 1.017 |
|  |  |  |  |  |  |  |
| BMI | 1.000 | 0.002 | 0.17 | 0.866 | 0.996 | 1.004 |
| HT_b | 0.997 | 0.024 | -0.12 | 0.902 | 0.950 | 1.046 |
| Antihypertensives_b | 0.905 | 0.034 | -2.67 | 0.007 | 0.842 | 0.974 |
| Hyperlipidaemia_b | 0.995 | 0.028 | -0.19 | 0.848 | 0.942 | 1.051 |
| Lipid_reg_treatment_b | 0.907 | 0.025 | -3.48 | 0.001 | 0.859 | 0.958 |
|  |  |  |  |  |  |  |
| Diabetes_type_b |  |  |  |  |  |  |
| T1DM | 1.839 | 0.218 | 5.13 | 0.000 | 1.457 | 2.320 |
| T2DM | 1.132 | 0.060 | 2.34 | 0.019 | 1.021 | 1.256 |
| DM - nos | 1.612 | 0.143 | 5.40 | 0.000 | 1.356 | 1.917 |
|  |  |  |  |  |  |  |
| Antidiabetics_b | 1.359 | 0.076 | 5.49 | 0.000 | 1.218 | 1.516 |
| Diuretics_b | 1.481 | 0.044 | 13.10 | 0.000 | 1.397 | 1.571 |
| Anticoagulants_b | 1.230 | 0.049 | 5.19 | 0.000 | 1.137 | 1.330 |
| Antiplatelets_b | 1.009 | 0.028 | 0.32 | 0.746 | 0.956 | 1.064 |
| HF_b | 4.688 | 0.161 | 44.95 | 0.000 | 4.383 | 5.015 |
| AF_b | 1.321 | 0.046 | 7.95 | 0.000 | 1.234 | 1.415 |
| Heart_valve_dis_b | 1.311 | 0.106 | 3.35 | 0.001 | 1.119 | 1.536 |
| VT_VF_b | 1.004 | 0.153 | 0.03 | 0.980 | 0.745 | 1.353 |
| Cardiomyopathy_b | 1.247 | 0.153 | 1.80 | 0.072 | 0.980 | 1.588 |
| CV_procedures_b | 0.836 | 0.031 | -4.87 | 0.000 | 0.778 | 0.899 |
| TIA_stroke_b | 1.070 | 0.034 | 2.10 | 0.036 | 1.004 | 1.139 |
| PVD_b | 1.445 | 0.055 | 9.71 | 0.000 | 1.341 | 1.556 |
| CKD_b | 1.194 | 0.031 | 6.86 | 0.000 | 1.135 | 1.257 |
| Hypothyroidism_b | 0.954 | 0.036 | -1.27 | 0.203 | 0.886 | 1.026 |
| Liver_dis_b | 1.018 | 0.262 | 0.07 | 0.946 | 0.614 | 1.687 |
| Lupus_b | 1.864 | 0.303 | 3.83 | 0.000 | 1.356 | 2.563 |
| Erectile_dysfunction_b | 1.019 | 0.039 | 0.50 | 0.619 | 0.945 | 1.099 |
| Any_tumour_b | 1.101 | 0.030 | 3.52 | 0.000 | 1.043 | 1.161 |
| Menopause_b | 0.802 | 0.034 | -5.22 | 0.000 | 0.738 | 0.871 |
| Dementia_b | 0.762 | 0.049 | -4.24 | 0.000 | 0.672 | 0.864 |
| RA_b | 1.208 | 0.066 | 3.46 | 0.001 | 1.086 | 1.345 |
| _cons | 0.009 | 0.001 | -41.27 | 0.000 | 0.007 | 0.011 |

**Performance in validation dataset (CPRD GOLD) - External validation - Index AMI cases**

| AUROC (95% CI): 0.715 (0.705; 0.724) |  |
| --- | --- |
| 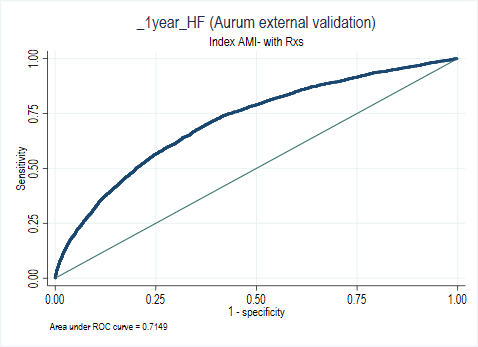 | 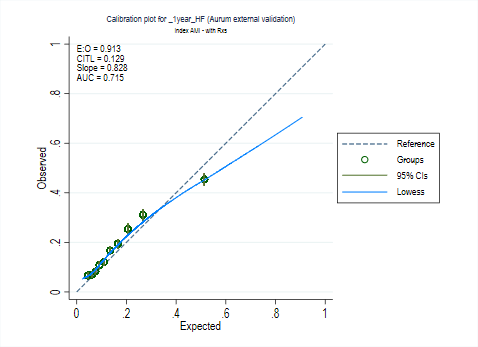 |
| 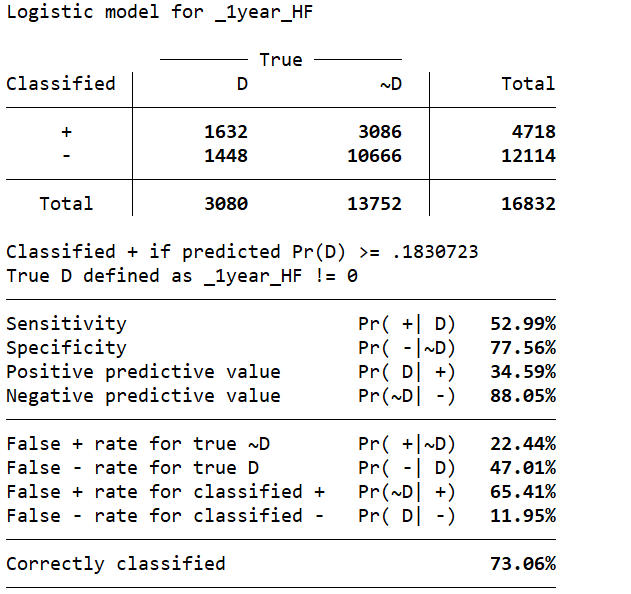 | % Correctly classified: 73.06 |

### 4) 1-year Recurrent MI

|  | | | | | | |
| --- | --- | --- | --- | --- | --- | --- |
| _1year_Recurrent_MI | Odds ratio | Std. err. | z | P>|z| | [95% conf. interval] | |
| age | 1.011 | 0.001 | 9.81 | 0.000 | 1.009 | 1.014 |
|  |  |  |  |  |  |  |
| gender |  |  |  |  |  |  |
| Female | 0.981 | 0.028 | -0.69 | 0.488 | 0.928 | 1.036 |
|  |  |  |  |  |  |  |
| IMD |  |  |  |  |  |  |
| Q2 | 1.016 | 0.036 | 0.45 | 0.652 | 0.948 | 1.090 |
| Q3 | 1.009 | 0.036 | 0.24 | 0.812 | 0.940 | 1.082 |
| Q4 | 0.986 | 0.036 | -0.37 | 0.710 | 0.918 | 1.060 |
| Q5-most deprived | 1.052 | 0.039 | 1.38 | 0.166 | 0.979 | 1.131 |
|  |  |  |  |  |  |  |
| Other than White |  |  |  |  |  |  |
| non-White | 1.047 | 0.050 | 0.97 | 0.332 | 0.954 | 1.149 |
|  |  |  |  |  |  |  |
| Drinker |  |  |  |  |  |  |
| drinker | 0.986 | 0.030 | -0.45 | 0.655 | 0.929 | 1.047 |
|  |  |  |  |  |  |  |
| Smoking_status |  |  |  |  |  |  |
| current smoker | 1.045 | 0.036 | 1.29 | 0.198 | 0.977 | 1.118 |
| ex-smoker | 0.957 | 0.030 | -1.43 | 0.153 | 0.900 | 1.017 |
|  |  |  |  |  |  |  |
| BMI | 0.995 | 0.002 | -2.40 | 0.017 | 0.990 | 0.999 |
| HT_b | 1.021 | 0.028 | 0.77 | 0.443 | 0.968 | 1.077 |
| Antihypertensives_b | 0.941 | 0.035 | -1.64 | 0.101 | 0.876 | 1.012 |
| Hyperlipidaemia_b | 0.991 | 0.031 | -0.29 | 0.776 | 0.933 | 1.053 |
| Lipid_reg_treatment_b | 0.914 | 0.028 | -2.96 | 0.003 | 0.861 | 0.970 |
|  |  |  |  |  |  |  |
| Diabetes_type_b |  |  |  |  |  |  |
| T1DM | 1.187 | 0.161 | 1.26 | 0.206 | 0.910 | 1.547 |
| T2DM | 1.022 | 0.062 | 0.35 | 0.725 | 0.907 | 1.150 |
| DM - nos | 1.321 | 0.132 | 2.78 | 0.005 | 1.085 | 1.607 |
|  |  |  |  |  |  |  |
| Antidiabetics_b | 1.170 | 0.075 | 2.46 | 0.014 | 1.032 | 1.327 |
| Diuretics_b | 1.138 | 0.036 | 4.04 | 0.000 | 1.069 | 1.211 |
| Anticoagulants_b | 0.974 | 0.048 | -0.55 | 0.585 | 0.885 | 1.071 |
| Antiplatelets_b | 1.075 | 0.032 | 2.42 | 0.015 | 1.014 | 1.141 |
| HF_b | 1.140 | 0.051 | 2.93 | 0.003 | 1.044 | 1.245 |
| AF_b | 0.981 | 0.042 | -0.45 | 0.652 | 0.902 | 1.067 |
| Heart_valve_dis_b | 1.300 | 0.121 | 2.81 | 0.005 | 1.083 | 1.562 |
| VT_VF_b | 0.598 | 0.131 | -2.35 | 0.019 | 0.389 | 0.919 |
| Cardiomyopathy_b | 0.803 | 0.138 | -1.28 | 0.201 | 0.573 | 1.124 |
| CV_procedures_b | 0.737 | 0.031 | -7.17 | 0.000 | 0.678 | 0.801 |
| TIA_stroke_b | 1.059 | 0.039 | 1.53 | 0.126 | 0.984 | 1.139 |
| PVD_b | 1.372 | 0.059 | 7.35 | 0.000 | 1.261 | 1.493 |
| CKD_b | 1.145 | 0.034 | 4.49 | 0.000 | 1.079 | 1.214 |
| Hypothyroidism_b | 1.059 | 0.044 | 1.38 | 0.167 | 0.976 | 1.148 |
| Liver_dis_b | 1.482 | 0.338 | 1.72 | 0.085 | 0.948 | 2.317 |
| Lupus_b | 1.172 | 0.224 | 0.83 | 0.406 | 0.805 | 1.706 |
| Erectile_dysfunction_b | 0.950 | 0.040 | -1.21 | 0.226 | 0.875 | 1.032 |
| Any_tumour_b | 1.064 | 0.033 | 2.00 | 0.046 | 1.001 | 1.130 |
| Menopause_b | 0.902 | 0.040 | -2.31 | 0.021 | 0.826 | 0.984 |
| Dementia_b | 1.131 | 0.078 | 1.79 | 0.074 | 0.988 | 1.295 |
| RA_b | 0.914 | 0.059 | -1.38 | 0.167 | 0.805 | 1.038 |
| _cons | 0.064 | 0.008 | -23.21 | 0.000 | 0.051 | 0.081 |

**Performance in validation dataset (CPRD GOLD) - External validation - Index AMI cases**

| AUROC (95% CI): 0.572 (0.563; 0.581) |  |
| --- | --- |
| 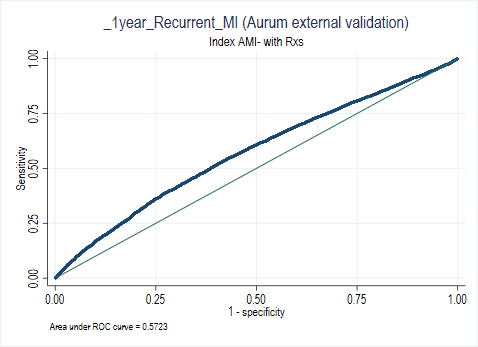 | 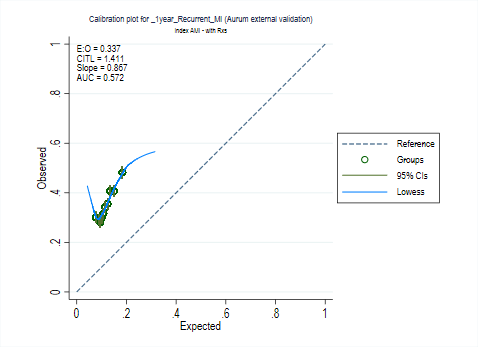 |
| 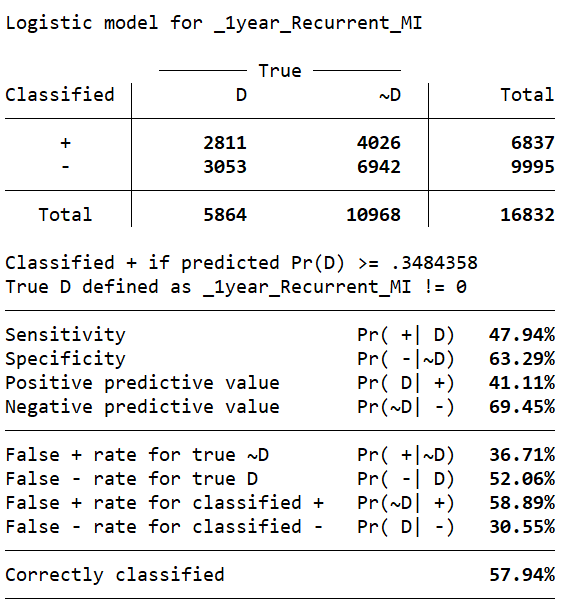 | % Correctly classified: 57.94 |

### 5) 1-year CV mortality

|  | | | | | | |
| --- | --- | --- | --- | --- | --- | --- |
| _1year_CV_death | Odds ratio | Std. err. | z | P>|z| | [95% conf. interval] | |
| age | 1.070 | 0.002 | 32.95 | 0.000 | 1.065 | 1.074 |
|  |  |  |  |  |  |  |
| gender |  |  |  |  |  |  |
| Female | 0.847 | 0.033 | -4.21 | 0.000 | 0.784 | 0.915 |
|  |  |  |  |  |  |  |
| IMD |  |  |  |  |  |  |
| Q2 | 0.973 | 0.052 | -0.51 | 0.607 | 0.876 | 1.080 |
| Q3 | 1.102 | 0.058 | 1.84 | 0.066 | 0.993 | 1.222 |
| Q4 | 1.175 | 0.062 | 3.04 | 0.002 | 1.059 | 1.304 |
| Q5-most deprived | 1.167 | 0.064 | 2.81 | 0.005 | 1.048 | 1.299 |
|  |  |  |  |  |  |  |
| Other than White |  |  |  |  |  |  |
| non-White | 0.776 | 0.066 | -2.98 | 0.003 | 0.657 | 0.917 |
|  |  |  |  |  |  |  |
| Drinker |  |  |  |  |  |  |
| drinker | 0.821 | 0.033 | -4.83 | 0.000 | 0.758 | 0.890 |
|  |  |  |  |  |  |  |
| Smoking_status |  |  |  |  |  |  |
| current smoker | 1.047 | 0.052 | 0.92 | 0.359 | 0.949 | 1.154 |
| ex-smoker | 0.785 | 0.033 | -5.67 | 0.000 | 0.722 | 0.854 |
|  |  |  |  |  |  |  |
| BMI | 0.981 | 0.003 | -5.65 | 0.000 | 0.974 | 0.987 |
| HT_b | 0.932 | 0.034 | -1.90 | 0.057 | 0.867 | 1.002 |
| Antihypertensives_b | 1.099 | 0.078 | 1.33 | 0.183 | 0.957 | 1.262 |
| Hyperlipidaemia_b | 0.920 | 0.041 | -1.87 | 0.062 | 0.844 | 1.004 |
| Lipid_reg_treatment_b | 0.871 | 0.037 | -3.22 | 0.001 | 0.800 | 0.947 |
|  |  |  |  |  |  |  |
| Diabetes_type_b |  |  |  |  |  |  |
| T1DM | 2.950 | 0.494 | 6.46 | 0.000 | 2.125 | 4.096 |
| T2DM | 1.064 | 0.086 | 0.76 | 0.445 | 0.907 | 1.247 |
| DM - nos | 1.416 | 0.190 | 2.60 | 0.009 | 1.089 | 1.841 |
|  |  |  |  |  |  |  |
| Antidiabetics_b | 1.439 | 0.123 | 4.25 | 0.000 | 1.217 | 1.701 |
| Diuretics_b | 1.591 | 0.080 | 9.19 | 0.000 | 1.441 | 1.756 |
| Anticoagulants_b | 1.099 | 0.063 | 1.65 | 0.099 | 0.982 | 1.229 |
| Antiplatelets_b | 1.237 | 0.054 | 4.87 | 0.000 | 1.135 | 1.347 |
| HF_b | 1.969 | 0.092 | 14.56 | 0.000 | 1.797 | 2.157 |
| AF_b | 1.294 | 0.062 | 5.35 | 0.000 | 1.177 | 1.422 |
| Heart_valve_dis_b | 1.554 | 0.159 | 4.31 | 0.000 | 1.272 | 1.899 |
| VT_VF_b | 1.133 | 0.244 | 0.58 | 0.561 | 0.743 | 1.728 |
| Cardiomyopathy_b | 1.066 | 0.210 | 0.32 | 0.746 | 0.724 | 1.569 |
| CV_procedures_b | 0.790 | 0.047 | -3.96 | 0.000 | 0.703 | 0.887 |
| TIA_stroke_b | 1.230 | 0.054 | 4.68 | 0.000 | 1.128 | 1.341 |
| PVD_b | 1.723 | 0.089 | 10.52 | 0.000 | 1.557 | 1.907 |
| CKD_b | 1.174 | 0.044 | 4.28 | 0.000 | 1.091 | 1.264 |
| Hypothyroidism_b | 1.020 | 0.055 | 0.37 | 0.715 | 0.917 | 1.134 |
| Liver_dis_b | 1.566 | 0.637 | 1.10 | 0.270 | 0.706 | 3.477 |
| Lupus_b | 1.567 | 0.425 | 1.65 | 0.098 | 0.920 | 2.667 |
| Erectile_dysfunction_b | 0.834 | 0.059 | -2.56 | 0.010 | 0.726 | 0.958 |
| Any_tumour_b | 1.054 | 0.043 | 1.30 | 0.193 | 0.974 | 1.141 |
| Menopause_b | 0.700 | 0.051 | -4.94 | 0.000 | 0.608 | 0.807 |
| Dementia_b | 1.528 | 0.109 | 5.94 | 0.000 | 1.328 | 1.757 |
| RA_b | 1.214 | 0.102 | 2.31 | 0.021 | 1.030 | 1.431 |
| _cons | 0.000 | 0.000 | -37.71 | 0.000 | 0.000 | 0.001 |

**Performance in validation dataset (CPRD GOLD) - External validation - Index AMI cases**

| AUROC (95% CI): 0.797 (0.784; 0.810) |  |
| --- | --- |
| 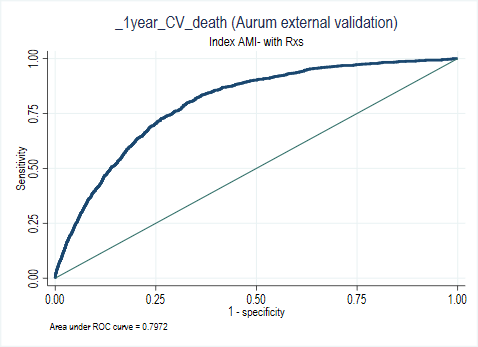 | 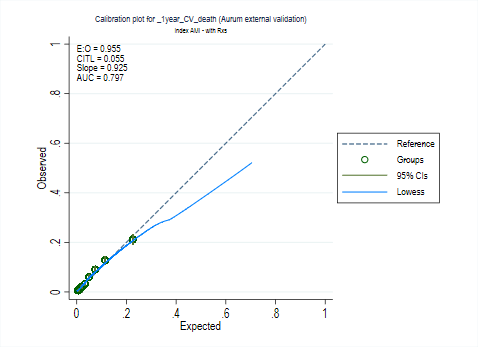 |
| 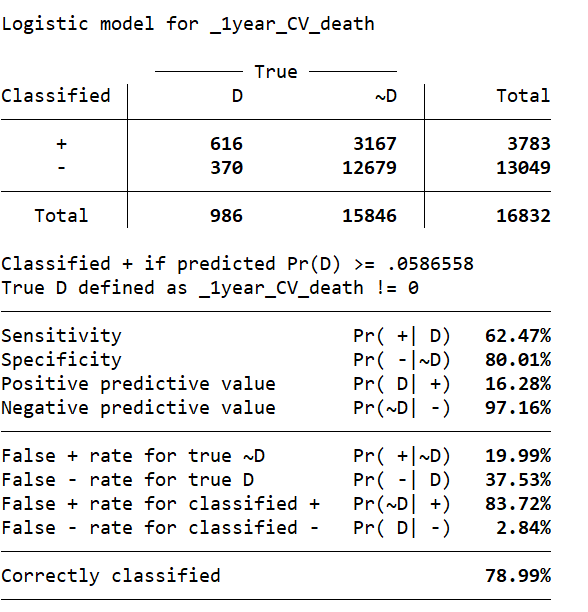 | % Correctly classified: 78.99 |

### 6) 1-year Composite 1 outcome (HF, stroke, recurrent MI, & all-cause mortality)

|  | | | | | | |
| --- | --- | --- | --- | --- | --- | --- |
| _1yr_Comp1_outcome | Odds ratio | Std. err. | z | P>|z| | [95% conf. interval] | |
| age | 1.034 | 0.001 | 39.21 | 0.000 | 1.032 | 1.036 |
|  |  |  |  |  |  |  |
| gender |  |  |  |  |  |  |
| Female | 0.977 | 0.020 | -1.11 | 0.267 | 0.939 | 1.018 |
|  |  |  |  |  |  |  |
| IMD |  |  |  |  |  |  |
| Q2 | 1.060 | 0.028 | 2.22 | 0.026 | 1.007 | 1.117 |
| Q3 | 1.070 | 0.028 | 2.55 | 0.011 | 1.016 | 1.127 |
| Q4 | 1.180 | 0.032 | 6.18 | 0.000 | 1.119 | 1.243 |
| Q5-most deprived | 1.251 | 0.034 | 8.25 | 0.000 | 1.186 | 1.320 |
|  |  |  |  |  |  |  |
| Other than White |  |  |  |  |  |  |
| non-White | 0.934 | 0.033 | -1.90 | 0.058 | 0.871 | 1.002 |
|  |  |  |  |  |  |  |
| Drinker |  |  |  |  |  |  |
| drinker | 0.924 | 0.021 | -3.53 | 0.000 | 0.884 | 0.965 |
|  |  |  |  |  |  |  |
| Smoking_status |  |  |  |  |  |  |
| current smoker | 1.112 | 0.028 | 4.18 | 0.000 | 1.058 | 1.169 |
| ex-smoker | 0.920 | 0.021 | -3.64 | 0.000 | 0.880 | 0.962 |
|  |  |  |  |  |  |  |
| BMI | 0.992 | 0.002 | -4.85 | 0.000 | 0.989 | 0.995 |
| HT_b | 0.961 | 0.019 | -1.99 | 0.046 | 0.924 | 0.999 |
| Antihypertensives_b | 0.980 | 0.026 | -0.74 | 0.459 | 0.930 | 1.033 |
| Hyperlipidaemia_b | 1.007 | 0.023 | 0.29 | 0.773 | 0.963 | 1.052 |
| Lipid_reg_treatment_b | 0.864 | 0.019 | -6.53 | 0.000 | 0.827 | 0.903 |
|  |  |  |  |  |  |  |
| Diabetes_type_b |  |  |  |  |  |  |
| T1DM | 1.689 | 0.172 | 5.16 | 0.000 | 1.384 | 2.061 |
| T2DM | 1.083 | 0.048 | 1.81 | 0.071 | 0.993 | 1.182 |
| DM - nos | 1.531 | 0.120 | 5.43 | 0.000 | 1.313 | 1.785 |
|  |  |  |  |  |  |  |
| Antidiabetics_b | 1.367 | 0.064 | 6.63 | 0.000 | 1.246 | 1.499 |
| Diuretics_b | 1.342 | 0.031 | 12.86 | 0.000 | 1.283 | 1.403 |
| Anticoagulants_b | 1.149 | 0.041 | 3.91 | 0.000 | 1.072 | 1.232 |
| Antiplatelets_b | 1.075 | 0.023 | 3.34 | 0.001 | 1.031 | 1.122 |
| HF_b | 3.108 | 0.113 | 31.30 | 0.000 | 2.895 | 3.337 |
| AF_b | 1.257 | 0.040 | 7.27 | 0.000 | 1.182 | 1.337 |
| Heart_valve_dis_b | 1.309 | 0.099 | 3.55 | 0.000 | 1.128 | 1.519 |
| VT_VF_b | 1.011 | 0.134 | 0.08 | 0.932 | 0.780 | 1.311 |
| Cardiomyopathy_b | 1.330 | 0.153 | 2.47 | 0.013 | 1.061 | 1.668 |
| CV_procedures_b | 0.711 | 0.021 | -11.38 | 0.000 | 0.670 | 0.754 |
| TIA_stroke_b | 2.045 | 0.056 | 25.94 | 0.000 | 1.937 | 2.158 |
| PVD_b | 1.544 | 0.052 | 12.91 | 0.000 | 1.445 | 1.649 |
| CKD_b | 1.199 | 0.027 | 8.18 | 0.000 | 1.148 | 1.253 |
| Hypothyroidism_b | 1.022 | 0.031 | 0.70 | 0.485 | 0.962 | 1.085 |
| Liver_dis_b | 1.382 | 0.253 | 1.77 | 0.077 | 0.966 | 1.978 |
| Lupus_b | 1.382 | 0.196 | 2.28 | 0.023 | 1.046 | 1.825 |
| Erectile_dysfunction_b | 0.962 | 0.029 | -1.27 | 0.205 | 0.906 | 1.021 |
| Any_tumour_b | 1.193 | 0.027 | 7.79 | 0.000 | 1.141 | 1.247 |
| Menopause_b | 0.793 | 0.026 | -7.13 | 0.000 | 0.744 | 0.845 |
| Dementia_b | 1.332 | 0.071 | 5.37 | 0.000 | 1.200 | 1.480 |
| RA_b | 1.077 | 0.049 | 1.62 | 0.105 | 0.985 | 1.177 |
| _cons | 0.038 | 0.003 | -37.00 | 0.000 | 0.032 | 0.046 |

**Performance in validation dataset (CPRD GOLD) - External validation - Index AMI cases**

| AUROC (95% CI): 0.655 (0.647; 0.663) |  |
| --- | --- |
| 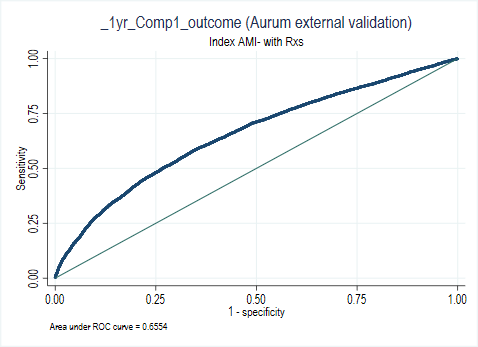 | 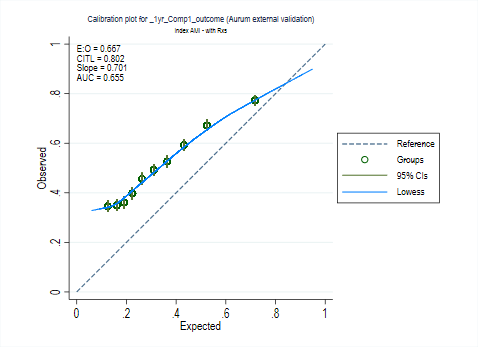 |
| 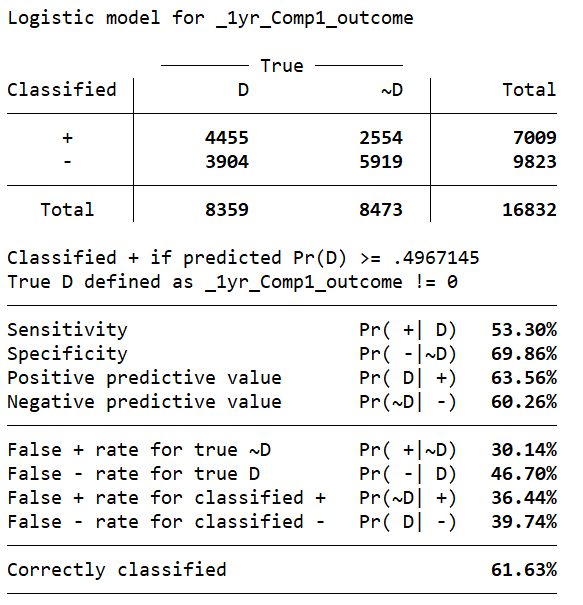 | % Correctly classified: 61.63 |

### 7) 1-year Composite 1 CV outcome (HF, stroke, recurrent MI, & CV mortality)

|  | | | | | | |
| --- | --- | --- | --- | --- | --- | --- |
| _1yr_Comp1_CV_outcome | Odds ratio | Std. err. | z | P>|z| | [95% conf. interval] | |
| age | 1.030 | 0.001 | 34.73 | 0.000 | 1.029 | 1.032 |
|  |  |  |  |  |  |  |
| gender |  |  |  |  |  |  |
| Female | 0.970 | 0.020 | -1.44 | 0.149 | 0.931 | 1.011 |
|  |  |  |  |  |  |  |
| IMD |  |  |  |  |  |  |
| Q2 | 1.020 | 0.027 | 0.76 | 0.448 | 0.968 | 1.075 |
| Q3 | 1.033 | 0.028 | 1.22 | 0.222 | 0.980 | 1.089 |
| Q4 | 1.134 | 0.031 | 4.65 | 0.000 | 1.075 | 1.195 |
| Q5-most deprived | 1.197 | 0.033 | 6.54 | 0.000 | 1.134 | 1.263 |
|  |  |  |  |  |  |  |
| Other than White |  |  |  |  |  |  |
| non-White | 0.996 | 0.036 | -0.12 | 0.905 | 0.928 | 1.068 |
|  |  |  |  |  |  |  |
| Drinker |  |  |  |  |  |  |
| drinker | 0.929 | 0.021 | -3.26 | 0.001 | 0.888 | 0.971 |
|  |  |  |  |  |  |  |
| Smoking_status |  |  |  |  |  |  |
| current smoker | 1.076 | 0.028 | 2.86 | 0.004 | 1.023 | 1.132 |
| ex-smoker | 0.937 | 0.022 | -2.81 | 0.005 | 0.896 | 0.981 |
|  |  |  |  |  |  |  |
| BMI | 0.997 | 0.002 | -1.74 | 0.083 | 0.994 | 1.000 |
| HT_b | 1.014 | 0.020 | 0.70 | 0.485 | 0.975 | 1.055 |
| Antihypertensives_b | 0.964 | 0.026 | -1.32 | 0.187 | 0.914 | 1.018 |
| Hyperlipidaemia_b | 0.997 | 0.023 | -0.15 | 0.879 | 0.953 | 1.042 |
| Lipid_reg_treatment_b | 0.896 | 0.020 | -4.85 | 0.000 | 0.857 | 0.937 |
|  |  |  |  |  |  |  |
| Diabetes_type_b |  |  |  |  |  |  |
| T1DM | 1.504 | 0.154 | 4.00 | 0.000 | 1.231 | 1.837 |
| T2DM | 1.035 | 0.046 | 0.77 | 0.439 | 0.948 | 1.130 |
| DM - nos | 1.386 | 0.109 | 4.16 | 0.000 | 1.189 | 1.617 |
|  |  |  |  |  |  |  |
| Antidiabetics_b | 1.416 | 0.067 | 7.33 | 0.000 | 1.291 | 1.554 |
| Diuretics_b | 1.317 | 0.030 | 11.89 | 0.000 | 1.259 | 1.378 |
| Anticoagulants_b | 1.102 | 0.039 | 2.73 | 0.006 | 1.028 | 1.182 |
| Antiplatelets_b | 1.056 | 0.023 | 2.47 | 0.013 | 1.011 | 1.103 |
| HF_b | 3.180 | 0.113 | 32.69 | 0.000 | 2.967 | 3.408 |
| AF_b | 1.297 | 0.041 | 8.28 | 0.000 | 1.220 | 1.379 |
| Heart_valve_dis_b | 1.223 | 0.092 | 2.67 | 0.008 | 1.055 | 1.417 |
| VT_VF_b | 0.934 | 0.125 | -0.51 | 0.610 | 0.719 | 1.214 |
| Cardiomyopathy_b | 1.573 | 0.181 | 3.94 | 0.000 | 1.256 | 1.971 |
| CV_procedures_b | 0.739 | 0.022 | -10.02 | 0.000 | 0.696 | 0.784 |
| TIA_stroke_b | 2.125 | 0.058 | 27.56 | 0.000 | 2.014 | 2.242 |
| PVD_b | 1.504 | 0.050 | 12.18 | 0.000 | 1.409 | 1.607 |
| CKD_b | 1.185 | 0.026 | 7.62 | 0.000 | 1.135 | 1.238 |
| Hypothyroidism_b | 1.000 | 0.031 | 0.02 | 0.987 | 0.941 | 1.063 |
| Liver_dis_b | 1.283 | 0.240 | 1.33 | 0.182 | 0.890 | 1.850 |
| Lupus_b | 1.364 | 0.195 | 2.16 | 0.030 | 1.030 | 1.805 |
| Erectile_dysfunction_b | 0.976 | 0.030 | -0.78 | 0.435 | 0.919 | 1.037 |
| Any_tumour_b | 1.070 | 0.025 | 2.95 | 0.003 | 1.023 | 1.120 |
| Menopause_b | 0.786 | 0.026 | -7.24 | 0.000 | 0.737 | 0.839 |
| Dementia_b | 1.100 | 0.059 | 1.79 | 0.074 | 0.991 | 1.221 |
| RA_b | 1.038 | 0.048 | 0.80 | 0.426 | 0.948 | 1.136 |
| _cons | 0.040 | 0.004 | -36.24 | 0.000 | 0.033 | 0.047 |

**Performance in validation dataset (CPRD GOLD) - External validation - Index AMI cases**

| AUROC (95% CI): 0.650 (0.641; 0.658) |  |
| --- | --- |
| 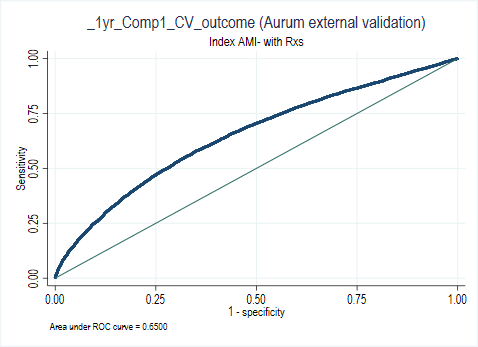 |  |
|  | % Correctly classified: 61.58 |

### 8) 1-year Composite 2 outcome (HF, stroke, & all-cause mortality)

|  | | | | | | |
| --- | --- | --- | --- | --- | --- | --- |
| _1yr_Comp2_outcome | Odds ratio | Std. err. | z | P>|z| | [95% conf. interval] | |
| age | 1.047 | 0.001 | 47.37 | 0.000 | 1.045 | 1.049 |
|  |  |  |  |  |  |  |
| gender |  |  |  |  |  |  |
| Female | 0.932 | 0.021 | -3.11 | 0.002 | 0.892 | 0.974 |
|  |  |  |  |  |  |  |
| IMD |  |  |  |  |  |  |
| Q2 | 1.086 | 0.032 | 2.82 | 0.005 | 1.026 | 1.150 |
| Q3 | 1.137 | 0.033 | 4.36 | 0.000 | 1.073 | 1.204 |
| Q4 | 1.264 | 0.037 | 7.93 | 0.000 | 1.193 | 1.340 |
| Q5-most deprived | 1.395 | 0.042 | 11.10 | 0.000 | 1.316 | 1.480 |
|  |  |  |  |  |  |  |
| Other than White |  |  |  |  |  |  |
| non-White | 0.922 | 0.037 | -2.00 | 0.045 | 0.852 | 0.998 |
|  |  |  |  |  |  |  |
| Drinker |  |  |  |  |  |  |
| drinker | 0.879 | 0.021 | -5.34 | 0.000 | 0.838 | 0.922 |
|  |  |  |  |  |  |  |
| Smoking_status |  |  |  |  |  |  |
| current smoker | 1.185 | 0.033 | 6.04 | 0.000 | 1.122 | 1.252 |
| ex-smoker | 0.944 | 0.024 | -2.31 | 0.021 | 0.899 | 0.991 |
|  |  |  |  |  |  |  |
| BMI | 0.989 | 0.002 | -6.25 | 0.000 | 0.985 | 0.992 |
| HT_b | 0.947 | 0.020 | -2.53 | 0.011 | 0.908 | 0.988 |
| Antihypertensives_b | 1.040 | 0.032 | 1.27 | 0.205 | 0.979 | 1.105 |
| Hyperlipidaemia_b | 1.002 | 0.025 | 0.08 | 0.934 | 0.955 | 1.051 |
| Lipid_reg_treatment_b | 0.879 | 0.022 | -5.26 | 0.000 | 0.838 | 0.922 |
|  |  |  |  |  |  |  |
| Diabetes_type_b |  |  |  |  |  |  |
| T1DM | 1.982 | 0.215 | 6.32 | 0.000 | 1.603 | 2.450 |
| T2DM | 1.071 | 0.051 | 1.44 | 0.151 | 0.975 | 1.175 |
| DM - nos | 1.542 | 0.127 | 5.24 | 0.000 | 1.311 | 1.813 |
|  |  |  |  |  |  |  |
| Antidiabetics_b | 1.463 | 0.074 | 7.53 | 0.000 | 1.325 | 1.615 |
| Diuretics_b | 1.418 | 0.036 | 13.95 | 0.000 | 1.350 | 1.490 |
| Anticoagulants_b | 1.265 | 0.047 | 6.35 | 0.000 | 1.176 | 1.360 |
| Antiplatelets_b | 1.074 | 0.026 | 3.02 | 0.003 | 1.025 | 1.126 |
| HF_b | 3.674 | 0.134 | 35.67 | 0.000 | 3.421 | 3.946 |
| AF_b | 1.310 | 0.043 | 8.31 | 0.000 | 1.230 | 1.397 |
| Heart_valve_dis_b | 1.329 | 0.104 | 3.65 | 0.000 | 1.141 | 1.549 |
| VT_VF_b | 0.912 | 0.129 | -0.65 | 0.518 | 0.691 | 1.205 |
| Cardiomyopathy_b | 1.679 | 0.200 | 4.34 | 0.000 | 1.329 | 2.121 |
| CV_procedures_b | 0.767 | 0.025 | -8.18 | 0.000 | 0.720 | 0.817 |
| TIA_stroke_b | 2.268 | 0.064 | 28.98 | 0.000 | 2.146 | 2.397 |
| PVD_b | 1.571 | 0.055 | 12.97 | 0.000 | 1.467 | 1.682 |
| CKD_b | 1.206 | 0.028 | 8.07 | 0.000 | 1.152 | 1.262 |
| Hypothyroidism_b | 1.012 | 0.033 | 0.36 | 0.720 | 0.949 | 1.079 |
| Liver_dis_b | 1.125 | 0.243 | 0.54 | 0.586 | 0.737 | 1.717 |
| Lupus_b | 1.324 | 0.205 | 1.81 | 0.071 | 0.977 | 1.794 |
| Erectile_dysfunction_b | 0.926 | 0.032 | -2.24 | 0.025 | 0.866 | 0.990 |
| Any_tumour_b | 1.264 | 0.030 | 9.78 | 0.000 | 1.206 | 1.324 |
| Menopause_b | 0.809 | 0.029 | -5.92 | 0.000 | 0.754 | 0.868 |
| Dementia_b | 1.381 | 0.075 | 5.95 | 0.000 | 1.241 | 1.536 |
| RA_b | 1.154 | 0.056 | 2.93 | 0.003 | 1.049 | 1.270 |
| _cons | 0.010 | 0.001 | -46.23 | 0.000 | 0.008 | 0.012 |

**Performance in validation dataset (CPRD GOLD) - External validation - Index AMI cases**

| AUROC (95% CI): 0.748 (0.739; 0.756) |  |
| --- | --- |
|  |  |
|  | % Correctly classified: 71.94 |

### 9) 1-year Composite 2 CV outcome (HF, stroke, & CV mortality)

|  | | | | | | |
| --- | --- | --- | --- | --- | --- | --- |
| _1yr_Comp2_CV_outcome | Odds ratio | Std. err. | z | P>|z| | [95% conf. interval] | |
| age | 1.043 | 0.001 | 42.57 | 0.000 | 1.041 | 1.046 |
|  |  |  |  |  |  |  |
| gender |  |  |  |  |  |  |
| Female | 0.945 | 0.022 | -2.44 | 0.015 | 0.904 | 0.989 |
|  |  |  |  |  |  |  |
| IMD |  |  |  |  |  |  |
| Q2 | 1.056 | 0.032 | 1.80 | 0.071 | 0.995 | 1.120 |
| Q3 | 1.092 | 0.033 | 2.92 | 0.004 | 1.029 | 1.159 |
| Q4 | 1.218 | 0.037 | 6.50 | 0.000 | 1.148 | 1.293 |
| Q5-most deprived | 1.334 | 0.041 | 9.37 | 0.000 | 1.256 | 1.417 |
|  |  |  |  |  |  |  |
| Other than White |  |  |  |  |  |  |
| non-White | 0.967 | 0.040 | -0.82 | 0.414 | 0.892 | 1.048 |
|  |  |  |  |  |  |  |
| Drinker |  |  |  |  |  |  |
| drinker | 0.925 | 0.023 | -3.13 | 0.002 | 0.882 | 0.972 |
|  |  |  |  |  |  |  |
| Smoking_status |  |  |  |  |  |  |
| current smoker | 1.127 | 0.033 | 4.13 | 0.000 | 1.065 | 1.192 |
| ex-smoker | 0.937 | 0.024 | -2.56 | 0.011 | 0.891 | 0.985 |
|  |  |  |  |  |  |  |
| BMI | 0.995 | 0.002 | -2.70 | 0.007 | 0.991 | 0.999 |
| HT_b | 0.994 | 0.022 | -0.29 | 0.771 | 0.952 | 1.037 |
| Antihypertensives_b | 1.013 | 0.033 | 0.40 | 0.688 | 0.951 | 1.080 |
| Hyperlipidaemia_b | 1.013 | 0.025 | 0.51 | 0.609 | 0.964 | 1.064 |
| Lipid_reg_treatment_b | 0.893 | 0.022 | -4.51 | 0.000 | 0.850 | 0.938 |
|  |  |  |  |  |  |  |
| Diabetes_type_b |  |  |  |  |  |  |
| T1DM | 1.878 | 0.206 | 5.73 | 0.000 | 1.514 | 2.329 |
| T2DM | 1.062 | 0.052 | 1.25 | 0.212 | 0.966 | 1.168 |
| DM - nos | 1.467 | 0.123 | 4.58 | 0.000 | 1.245 | 1.728 |
|  |  |  |  |  |  |  |
| Antidiabetics_b | 1.452 | 0.075 | 7.26 | 0.000 | 1.313 | 1.606 |
| Diuretics_b | 1.412 | 0.037 | 13.32 | 0.000 | 1.342 | 1.486 |
| Anticoagulants_b | 1.222 | 0.046 | 5.36 | 0.000 | 1.135 | 1.314 |
| Antiplatelets_b | 1.074 | 0.026 | 2.93 | 0.003 | 1.024 | 1.127 |
| HF_b | 3.761 | 0.134 | 37.24 | 0.000 | 3.508 | 4.033 |
| AF_b | 1.354 | 0.044 | 9.27 | 0.000 | 1.270 | 1.444 |
| Heart_valve_dis_b | 1.315 | 0.102 | 3.52 | 0.000 | 1.129 | 1.531 |
| VT_VF_b | 0.986 | 0.141 | -0.10 | 0.921 | 0.746 | 1.304 |
| Cardiomyopathy_b | 1.834 | 0.218 | 5.11 | 0.000 | 1.454 | 2.314 |
| CV_procedures_b | 0.804 | 0.027 | -6.60 | 0.000 | 0.753 | 0.858 |
| TIA_stroke_b | 2.415 | 0.068 | 31.23 | 0.000 | 2.285 | 2.552 |
| PVD_b | 1.572 | 0.055 | 12.89 | 0.000 | 1.467 | 1.684 |
| CKD_b | 1.173 | 0.028 | 6.75 | 0.000 | 1.120 | 1.229 |
| Hypothyroidism_b | 0.961 | 0.032 | -1.18 | 0.236 | 0.899 | 1.026 |
| Liver_dis_b | 0.881 | 0.208 | -0.54 | 0.591 | 0.554 | 1.400 |
| Lupus_b | 1.313 | 0.209 | 1.71 | 0.088 | 0.960 | 1.794 |
| Erectile_dysfunction_b | 0.932 | 0.033 | -2.01 | 0.045 | 0.870 | 0.998 |
| Any_tumour_b | 1.073 | 0.027 | 2.85 | 0.004 | 1.022 | 1.127 |
| Menopause_b | 0.756 | 0.028 | -7.48 | 0.000 | 0.702 | 0.813 |
| Dementia_b | 1.080 | 0.060 | 1.39 | 0.163 | 0.969 | 1.203 |
| RA_b | 1.133 | 0.057 | 2.49 | 0.013 | 1.027 | 1.250 |
| _cons | 0.009 | 0.001 | -45.64 | 0.000 | 0.007 | 0.011 |

**Performance in validation dataset (CPRD GOLD) - External validation - Index AMI cases**

| AUROC (95% CI): 0.741 (0.731; 0.750) |  |
| --- | --- |
|  |  |
|  | % Correctly classified: 72.28 |

## Internal validation (CPRD Aurum) - without therapies

### 1) 5-year all-cause mortality

|  | | | | | | |
| --- | --- | --- | --- | --- | --- | --- |
| _5year_All_cause_death | Odds ratio | Std. err. | z | P>|z| | [95% conf. interval] | |
| age | 1.102 | 0.001 | 89.21 | 0.000 | 1.099 | 1.104 |
|  |  |  |  |  |  |  |
| gender |  |  |  |  |  |  |
| Female | 1.004 | 0.023 | 0.19 | 0.846 | 0.960 | 1.051 |
|  |  |  |  |  |  |  |
| IMD |  |  |  |  |  |  |
| Q2 | 1.112 | 0.034 | 3.49 | 0.000 | 1.048 | 1.181 |
| Q3 | 1.232 | 0.038 | 6.83 | 0.000 | 1.161 | 1.308 |
| Q4 | 1.337 | 0.041 | 9.37 | 0.000 | 1.258 | 1.420 |
| Q5-most deprived | 1.509 | 0.048 | 13.05 | 0.000 | 1.418 | 1.605 |
| unknown | 0.674 | 0.250 | -1.07 | 0.287 | 0.326 | 1.393 |
|  |  |  |  |  |  |  |
| Other than White | 1.077 | 0.041 | 1.96 | 0.050 | 1.000 | 1.160 |
| Drinker | 1.028 | 0.010 | 2.75 | 0.006 | 1.008 | 1.049 |
|  |  |  |  |  |  |  |
| Smoking_status |  |  |  |  |  |  |
| current smoker | 1.692 | 0.051 | 17.58 | 0.000 | 1.596 | 1.794 |
| ex-smoker | 0.993 | 0.026 | -0.27 | 0.784 | 0.943 | 1.045 |
| unknown | 1.747 | 0.233 | 4.18 | 0.000 | 1.344 | 2.269 |
|  |  |  |  |  |  |  |
| BMI | 0.981 | 0.002 | -9.93 | 0.000 | 0.977 | 0.985 |
| HT_b | 1.014 | 0.021 | 0.66 | 0.508 | 0.974 | 1.055 |
| Hyperlipidaemia_b | 0.885 | 0.022 | -4.99 | 0.000 | 0.843 | 0.928 |
|  |  |  |  |  |  |  |
| Diabetes_type_b |  |  |  |  |  |  |
| T1DM | 5.972 | 0.628 | 16.99 | 0.000 | 4.859 | 7.340 |
| T2DM | 1.731 | 0.046 | 20.80 | 0.000 | 1.644 | 1.823 |
| DM - nos | 2.681 | 0.211 | 12.54 | 0.000 | 2.298 | 3.128 |
|  |  |  |  |  |  |  |
| HF_b | 2.379 | 0.092 | 22.41 | 0.000 | 2.205 | 2.566 |
| AF_b | 1.524 | 0.048 | 13.50 | 0.000 | 1.434 | 1.620 |
| Heart_valve_dis_b | 1.503 | 0.126 | 4.88 | 0.000 | 1.276 | 1.771 |
| VT_VF_b | 1.369 | 0.204 | 2.11 | 0.035 | 1.022 | 1.834 |
| Cardiomyopathy_b | 1.081 | 0.141 | 0.60 | 0.548 | 0.838 | 1.396 |
| CV_procedures_b | 0.856 | 0.028 | -4.73 | 0.000 | 0.803 | 0.913 |
| TIA_stroke_b | 1.599 | 0.047 | 16.00 | 0.000 | 1.510 | 1.694 |
| PVD_b | 2.018 | 0.074 | 19.11 | 0.000 | 1.878 | 2.169 |
| CKD_b | 1.544 | 0.037 | 18.21 | 0.000 | 1.473 | 1.618 |
| Hypothyroidism_b | 0.992 | 0.034 | -0.24 | 0.810 | 0.928 | 1.060 |
| Liver_dis_b | 1.873 | 0.413 | 2.85 | 0.004 | 1.216 | 2.884 |
| Lupus_b | 1.640 | 0.270 | 3.00 | 0.003 | 1.187 | 2.265 |
| Erectile_dysfunction_b | 0.799 | 0.029 | -6.13 | 0.000 | 0.744 | 0.859 |
| Any_tumour_b | 1.618 | 0.040 | 19.54 | 0.000 | 1.541 | 1.698 |
| Menopause_b | 0.783 | 0.029 | -6.62 | 0.000 | 0.728 | 0.842 |
| Dementia_b | 4.021 | 0.281 | 19.93 | 0.000 | 3.507 | 4.611 |
| RA_b | 1.436 | 0.072 | 7.21 | 0.000 | 1.301 | 1.584 |
| _cons | 0.000 | 0.000 | -75.18 | 0.000 | 0.000 | 0.000 |

**Performance in validation dataset (20% of practices) - Internal validation - Index AMI cases**

| AUROC (95% CI): 0.843 (0.837; 0.849) |  |
| --- | --- |
|  |  |
|  | % Correctly classified: 77.83 |

### 2) 5-year Stroke

|  | | | | | | |
| --- | --- | --- | --- | --- | --- | --- |
| _5year_Stroke | Odds ratio | Std. err. | z | P>|z| | [95% conf. interval] | |
| age | 1.069 | 0.001 | 60.15 | 0.000 | 1.067 | 1.071 |
|  |  |  |  |  |  |  |
| gender |  |  |  |  |  |  |
| Female | 1.135 | 0.028 | 5.12 | 0.000 | 1.081 | 1.192 |
|  |  |  |  |  |  |  |
| IMD |  |  |  |  |  |  |
| Q2 | 1.017 | 0.033 | 0.52 | 0.600 | 0.954 | 1.085 |
| Q3 | 1.101 | 0.036 | 2.93 | 0.003 | 1.032 | 1.174 |
| Q4 | 1.211 | 0.040 | 5.79 | 0.000 | 1.135 | 1.293 |
| Q5-most deprived | 1.297 | 0.044 | 7.71 | 0.000 | 1.214 | 1.386 |
|  |  |  |  |  |  |  |
| Other than White | 1.002 | 0.046 | 0.05 | 0.961 | 0.916 | 1.096 |
| Drinker | 0.870 | 0.023 | -5.19 | 0.000 | 0.826 | 0.917 |
|  |  |  |  |  |  |  |
| Smoking_status |  |  |  |  |  |  |
| current smoker | 1.206 | 0.038 | 5.94 | 0.000 | 1.134 | 1.283 |
| ex-smoker | 0.911 | 0.025 | -3.37 | 0.001 | 0.863 | 0.962 |
|  |  |  |  |  |  |  |
| BMI | 0.985 | 0.002 | -7.07 | 0.000 | 0.981 | 0.989 |
| HT_b | 1.226 | 0.026 | 9.44 | 0.000 | 1.175 | 1.279 |
| Hyperlipidaemia_b | 1.067 | 0.027 | 2.54 | 0.011 | 1.015 | 1.123 |
|  |  |  |  |  |  |  |
| Diabetes_type_b |  |  |  |  |  |  |
| T1DM | 3.639 | 0.386 | 12.18 | 0.000 | 2.956 | 4.480 |
| T2DM | 1.629 | 0.044 | 17.89 | 0.000 | 1.545 | 1.719 |
| DM - nos | 1.753 | 0.141 | 6.96 | 0.000 | 1.497 | 2.053 |
|  |  |  |  |  |  |  |
| HF_b | 1.525 | 0.056 | 11.41 | 0.000 | 1.419 | 1.640 |
| AF_b | 1.720 | 0.054 | 17.41 | 0.000 | 1.618 | 1.828 |
| Heart_valve_dis_b | 1.362 | 0.112 | 3.75 | 0.000 | 1.159 | 1.600 |
| VT_VF_b | 1.065 | 0.164 | 0.41 | 0.680 | 0.789 | 1.439 |
| Cardiomyopathy_b | 1.232 | 0.163 | 1.58 | 0.114 | 0.951 | 1.597 |
| CV_procedures_b | 0.890 | 0.031 | -3.34 | 0.001 | 0.831 | 0.953 |
| TIA_stroke_b | 7.798 | 0.233 | 68.64 | 0.000 | 7.354 | 8.269 |
| PVD_b | 1.936 | 0.071 | 18.00 | 0.000 | 1.802 | 2.080 |
| CKD_b | 1.355 | 0.033 | 12.31 | 0.000 | 1.291 | 1.422 |
| Hypothyroidism_b | 1.019 | 0.036 | 0.54 | 0.588 | 0.951 | 1.093 |
| Liver_dis_b | 1.500 | 0.365 | 1.67 | 0.096 | 0.931 | 2.418 |
| Lupus_b | 1.511 | 0.263 | 2.37 | 0.018 | 1.075 | 2.124 |
| Erectile_dysfunction_b | 1.106 | 0.042 | 2.62 | 0.009 | 1.026 | 1.193 |
| Any_tumour_b | 1.117 | 0.029 | 4.20 | 0.000 | 1.061 | 1.176 |
| Menopause_b | 0.861 | 0.034 | -3.81 | 0.000 | 0.797 | 0.930 |
| Dementia_b | 3.188 | 0.188 | 19.63 | 0.000 | 2.839 | 3.579 |
| RA_b | 0.894 | 0.050 | -1.99 | 0.047 | 0.801 | 0.998 |
| _cons | 0.001 | 0.000 | -55.92 | 0.000 | 0.001 | 0.002 |

**Performance in validation dataset (20% of practices) - Internal validation - Index AMI cases**

| AUROC (95% CI): 0.831 (0.824; 0.838) |  |
| --- | --- |
|  |  |
|  | % Correctly classified: 79.02 |

### 3) 5-year heart failure (HF)

|  | | | | | | |
| --- | --- | --- | --- | --- | --- | --- |
| _5year_HF | Odds ratio | Std. err. | z | P>|z| | [95% conf. interval] | |
| age | 1.067 | 0.001 | 70.59 | 0.000 | 1.065 | 1.069 |
|  |  |  |  |  |  |  |
| gender |  |  |  |  |  |  |
| Female | 1.007 | 0.022 | 0.33 | 0.740 | 0.965 | 1.051 |
|  |  |  |  |  |  |  |
| IMD |  |  |  |  |  |  |
| Q2 | 1.084 | 0.031 | 2.83 | 0.005 | 1.025 | 1.146 |
| Q3 | 1.139 | 0.033 | 4.56 | 0.000 | 1.077 | 1.205 |
| Q4 | 1.302 | 0.038 | 9.16 | 0.000 | 1.231 | 1.378 |
| Q5-most deprived | 1.587 | 0.046 | 15.79 | 0.000 | 1.498 | 1.680 |
|  |  |  |  |  |  |  |
| Other than White | 1.050 | 0.040 | 1.28 | 0.200 | 0.974 | 1.132 |
| Drinker | 0.838 | 0.020 | -7.42 | 0.000 | 0.799 | 0.878 |
|  |  |  |  |  |  |  |
| Smoking_status |  |  |  |  |  |  |
| current smoker | 1.322 | 0.036 | 10.13 | 0.000 | 1.253 | 1.396 |
| ex-smoker | 1.012 | 0.025 | 0.49 | 0.626 | 0.964 | 1.062 |
|  |  |  |  |  |  |  |
| BMI | 1.011 | 0.002 | 6.00 | 0.000 | 1.007 | 1.014 |
| HT_b | 1.084 | 0.021 | 4.25 | 0.000 | 1.044 | 1.125 |
| Hyperlipidaemia_b | 0.953 | 0.022 | -2.09 | 0.037 | 0.911 | 0.997 |
|  |  |  |  |  |  |  |
| Diabetes_type_b |  |  |  |  |  |  |
| T1DM | 3.804 | 0.370 | 13.74 | 0.000 | 3.144 | 4.602 |
| T2DM | 1.710 | 0.042 | 21.75 | 0.000 | 1.629 | 1.795 |
| DM - nos | 2.499 | 0.189 | 12.13 | 0.000 | 2.155 | 2.898 |
|  |  |  |  |  |  |  |
| HF_b | 9.124 | 0.457 | 44.19 | 0.000 | 8.272 | 10.064 |
| AF_b | 1.974 | 0.061 | 21.96 | 0.000 | 1.858 | 2.098 |
| Heart_valve_dis_b | 1.751 | 0.150 | 6.55 | 0.000 | 1.481 | 2.071 |
| VT_VF_b | 1.701 | 0.247 | 3.66 | 0.000 | 1.280 | 2.261 |
| Cardiomyopathy_b | 2.242 | 0.304 | 5.96 | 0.000 | 1.719 | 2.923 |
| CV_procedures_b | 1.030 | 0.031 | 0.97 | 0.331 | 0.970 | 1.093 |
| TIA_stroke_b | 1.289 | 0.037 | 8.95 | 0.000 | 1.220 | 1.363 |
| PVD_b | 1.763 | 0.063 | 15.96 | 0.000 | 1.644 | 1.890 |
| CKD_b | 1.535 | 0.035 | 18.55 | 0.000 | 1.467 | 1.606 |
| Hypothyroidism_b | 0.958 | 0.031 | -1.32 | 0.188 | 0.898 | 1.021 |
| Liver_dis_b | 1.488 | 0.301 | 1.97 | 0.049 | 1.001 | 2.212 |
| Lupus_b | 1.879 | 0.284 | 4.17 | 0.000 | 1.397 | 2.526 |
| Erectile_dysfunction_b | 0.976 | 0.032 | -0.75 | 0.451 | 0.916 | 1.040 |
| Any_tumour_b | 1.176 | 0.028 | 6.79 | 0.000 | 1.122 | 1.233 |
| Menopause_b | 0.779 | 0.027 | -7.21 | 0.000 | 0.728 | 0.834 |
| Dementia_b | 1.777 | 0.103 | 9.87 | 0.000 | 1.585 | 1.992 |
| RA_b | 1.205 | 0.058 | 3.89 | 0.000 | 1.097 | 1.324 |
| _cons | 0.002 | 0.000 | -64.00 | 0.000 | 0.002 | 0.002 |

**Performance in validation dataset (20% of practices) - Internal validation - Index AMI cases**

| AUROC (95% CI): 0.793 (0.786; 0.800) |  |
| --- | --- |
|  |  |
|  | % Correctly classified: 73.68 |

### 4) 5- year Recurrent MI outcome

|  | | | | | | |
| --- | --- | --- | --- | --- | --- | --- |
| _5year_Recurrent_MI | Odds ratio | Std. err. | z | P>|z| | [95% conf. interval] | |
| age | 1.033 | 0.001 | 36.70 | 0.000 | 1.031 | 1.035 |
|  |  |  |  |  |  |  |
| gender |  |  |  |  |  |  |
| Female | 1.025 | 0.022 | 1.11 | 0.268 | 0.981 | 1.070 |
|  |  |  |  |  |  |  |
| IMD |  |  |  |  |  |  |
| Q2 | 1.029 | 0.029 | 1.01 | 0.312 | 0.973 | 1.089 |
| Q3 | 1.067 | 0.031 | 2.26 | 0.024 | 1.009 | 1.129 |
| Q4 | 1.085 | 0.031 | 2.81 | 0.005 | 1.025 | 1.148 |
| Q5-most deprived | 1.166 | 0.034 | 5.23 | 0.000 | 1.101 | 1.235 |
|  |  |  |  |  |  |  |
| Other than White | 1.076 | 0.041 | 1.92 | 0.055 | 0.998 | 1.160 |
| Drinker | 0.913 | 0.022 | -3.84 | 0.000 | 0.871 | 0.956 |
|  |  |  |  |  |  |  |
| Smoking_status |  |  |  |  |  |  |
| current smoker | 1.177 | 0.032 | 5.93 | 0.000 | 1.115 | 1.242 |
| ex-smoker | 0.940 | 0.023 | -2.51 | 0.012 | 0.896 | 0.987 |
|  |  |  |  |  |  |  |
| BMI | 0.994 | 0.002 | -3.28 | 0.001 | 0.991 | 0.998 |
| HT_b | 1.066 | 0.020 | 3.33 | 0.001 | 1.027 | 1.107 |
| Hyperlipidaemia_b | 0.991 | 0.023 | -0.40 | 0.689 | 0.947 | 1.036 |
|  |  |  |  |  |  |  |
| Diabetes_type_b |  |  |  |  |  |  |
| T1DM | 2.659 | 0.243 | 10.71 | 0.000 | 2.223 | 3.180 |
| T2DM | 1.302 | 0.032 | 10.70 | 0.000 | 1.240 | 1.366 |
| DM - nos | 1.732 | 0.122 | 7.78 | 0.000 | 1.508 | 1.989 |
|  |  |  |  |  |  |  |
| HF_b | 1.614 | 0.054 | 14.28 | 0.000 | 1.512 | 1.724 |
| AF_b | 1.161 | 0.034 | 5.13 | 0.000 | 1.097 | 1.230 |
| Heart_valve_dis_b | 1.368 | 0.101 | 4.23 | 0.000 | 1.183 | 1.582 |
| VT_VF_b | 0.776 | 0.113 | -1.74 | 0.081 | 0.584 | 1.032 |
| Cardiomyopathy_b | 0.910 | 0.113 | -0.77 | 0.444 | 0.714 | 1.159 |
| CV_procedures_b | 0.980 | 0.030 | -0.65 | 0.519 | 0.923 | 1.041 |
| TIA_stroke_b | 1.287 | 0.035 | 9.23 | 0.000 | 1.220 | 1.358 |
| PVD_b | 1.586 | 0.053 | 13.81 | 0.000 | 1.486 | 1.694 |
| CKD_b | 1.353 | 0.031 | 13.28 | 0.000 | 1.294 | 1.415 |
| Hypothyroidism_b | 1.067 | 0.034 | 2.02 | 0.044 | 1.002 | 1.137 |
| Liver_dis_b | 1.738 | 0.331 | 2.90 | 0.004 | 1.196 | 2.525 |
| Lupus_b | 1.496 | 0.222 | 2.71 | 0.007 | 1.118 | 2.003 |
| Erectile_dysfunction_b | 0.854 | 0.029 | -4.65 | 0.000 | 0.799 | 0.913 |
| Any_tumour_b | 1.129 | 0.027 | 5.08 | 0.000 | 1.078 | 1.184 |
| Menopause_b | 0.728 | 0.026 | -8.79 | 0.000 | 0.678 | 0.781 |
| Dementia_b | 2.072 | 0.108 | 14.04 | 0.000 | 1.872 | 2.294 |
| RA_b | 0.958 | 0.048 | -0.85 | 0.393 | 0.869 | 1.057 |
| _cons | 0.025 | 0.002 | -38.92 | 0.000 | 0.021 | 0.030 |

**Performance in validation dataset (20% of practices) - Internal validation - Index AMI cases**

| AUROC (95% CI): 0.675 (0.665; 0.684) |  |
| --- | --- |
|  |  |
|  | % Correctly classified: 66.83 |

### 5) 5-year CV mortality

|  | | | | | | |
| --- | --- | --- | --- | --- | --- | --- |
| _5year_CV_death | Odds ratio | Std. err. | z | P>|z| | [95% conf. interval] | |
| age | 1.106 | 0.001 | 74.37 | 0.000 | 1.103 | 1.108 |
|  |  |  |  |  |  |  |
| gender |  |  |  |  |  |  |
| Female | 0.883 | 0.023 | -4.69 | 0.000 | 0.839 | 0.930 |
|  |  |  |  |  |  |  |
| IMD |  |  |  |  |  |  |
| Q2 | 1.159 | 0.041 | 4.14 | 0.000 | 1.081 | 1.243 |
| Q3 | 1.284 | 0.046 | 7.01 | 0.000 | 1.197 | 1.377 |
| Q4 | 1.393 | 0.050 | 9.18 | 0.000 | 1.298 | 1.495 |
| Q5-most deprived | 1.465 | 0.054 | 10.32 | 0.000 | 1.363 | 1.576 |
|  |  |  |  |  |  |  |
| Other than White | 0.813 | 0.043 | -3.87 | 0.000 | 0.732 | 0.903 |
| Drinker | 0.763 | 0.022 | -9.61 | 0.000 | 0.722 | 0.806 |
|  |  |  |  |  |  |  |
| Smoking_status |  |  |  |  |  |  |
| current smoker | 1.360 | 0.046 | 9.01 | 0.000 | 1.272 | 1.455 |
| ex-smoker | 0.868 | 0.026 | -4.76 | 0.000 | 0.819 | 0.920 |
|  |  |  |  |  |  |  |
| BMI | 0.990 | 0.002 | -4.48 | 0.000 | 0.986 | 0.994 |
| HT_b | 1.060 | 0.025 | 2.51 | 0.012 | 1.013 | 1.110 |
| Hyperlipidaemia_b | 0.949 | 0.027 | -1.87 | 0.061 | 0.898 | 1.002 |
|  |  |  |  |  |  |  |
| Diabetes_type_b |  |  |  |  |  |  |
| T1DM | 8.377 | 0.932 | 19.11 | 0.000 | 6.736 | 10.418 |
| T2DM | 1.862 | 0.054 | 21.35 | 0.000 | 1.759 | 1.972 |
| DM - nos | 2.916 | 0.241 | 12.94 | 0.000 | 2.479 | 3.429 |
|  |  |  |  |  |  |  |
| HF_b | 2.993 | 0.110 | 29.76 | 0.000 | 2.784 | 3.217 |
| AF_b | 1.611 | 0.051 | 15.05 | 0.000 | 1.514 | 1.714 |
| Heart_valve_dis_b | 1.912 | 0.158 | 7.84 | 0.000 | 1.626 | 2.248 |
| VT_VF_b | 1.215 | 0.192 | 1.24 | 0.216 | 0.892 | 1.656 |
| Cardiomyopathy_b | 1.262 | 0.176 | 1.67 | 0.095 | 0.960 | 1.659 |
| CV_procedures_b | 0.980 | 0.037 | -0.52 | 0.600 | 0.911 | 1.055 |
| TIA_stroke_b | 1.614 | 0.049 | 15.79 | 0.000 | 1.521 | 1.713 |
| PVD_b | 2.168 | 0.082 | 20.56 | 0.000 | 2.013 | 2.333 |
| CKD_b | 1.403 | 0.036 | 13.24 | 0.000 | 1.334 | 1.475 |
| Hypothyroidism_b | 0.970 | 0.037 | -0.82 | 0.414 | 0.901 | 1.044 |
| Liver_dis_b | 1.817 | 0.503 | 2.16 | 0.031 | 1.056 | 3.125 |
| Lupus_b | 2.093 | 0.381 | 4.06 | 0.000 | 1.466 | 2.989 |
| Erectile_dysfunction_b | 0.785 | 0.035 | -5.37 | 0.000 | 0.718 | 0.857 |
| Any_tumour_b | 1.045 | 0.029 | 1.59 | 0.111 | 0.990 | 1.103 |
| Menopause_b | 0.695 | 0.032 | -7.97 | 0.000 | 0.636 | 0.760 |
| Dementia_b | 3.500 | 0.202 | 21.68 | 0.000 | 3.125 | 3.920 |
| RA_b | 1.500 | 0.084 | 7.23 | 0.000 | 1.344 | 1.675 |
| _cons | 0.000 | 0.000 | -67.06 | 0.000 | 0.000 | 0.000 |

**Performance in validation dataset (20% of practices) - Internal validation - Index AMI cases**

| AUROC (95% CI): 0.854 (0.847; 0.860) |  |
| --- | --- |
|  |  |
|  | % Correctly classified: 80.95 |

### 6) 5-year Composite 1 outcome (HF, stroke, recurrent MI, & all-cause mortality)

|  | | | | | | |
| --- | --- | --- | --- | --- | --- | --- |
| _5yr_Comp1_outcome | Odds ratio | Std. err. | z | P>|z| | [95% conf. interval] | |
| age | 1.060 | 0.001 | 71.10 | 0.000 | 1.059 | 1.062 |
|  |  |  |  |  |  |  |
| gender |  |  |  |  |  |  |
| Female | 1.059 | 0.023 | 2.68 | 0.007 | 1.016 | 1.105 |
|  |  |  |  |  |  |  |
| IMD |  |  |  |  |  |  |
| Q2 | 1.076 | 0.029 | 2.75 | 0.006 | 1.021 | 1.133 |
| Q3 | 1.197 | 0.032 | 6.74 | 0.000 | 1.136 | 1.261 |
| Q4 | 1.309 | 0.035 | 9.95 | 0.000 | 1.241 | 1.380 |
| Q5-most deprived | 1.499 | 0.041 | 14.68 | 0.000 | 1.420 | 1.583 |
|  |  |  |  |  |  |  |
| Other than White | 0.975 | 0.034 | -0.73 | 0.467 | 0.911 | 1.044 |
| Drinker | 0.865 | 0.021 | -6.12 | 0.000 | 0.825 | 0.906 |
|  |  |  |  |  |  |  |
| Smoking_status |  |  |  |  |  |  |
| current smoker | 1.302 | 0.034 | 10.16 | 0.000 | 1.238 | 1.371 |
| ex-smoker | 0.952 | 0.023 | -2.09 | 0.036 | 0.908 | 0.997 |
|  |  |  |  |  |  |  |
| BMI | 0.996 | 0.002 | -2.22 | 0.026 | 0.993 | 1.000 |
| HT_b | 1.081 | 0.020 | 4.26 | 0.000 | 1.043 | 1.121 |
| Hyperlipidaemia_b | 0.952 | 0.021 | -2.20 | 0.028 | 0.911 | 0.995 |
|  |  |  |  |  |  |  |
| Diabetes_type_b |  |  |  |  |  |  |
| T1DM | 3.567 | 0.361 | 12.56 | 0.000 | 2.925 | 4.350 |
| T2DM | 1.627 | 0.041 | 19.11 | 0.000 | 1.548 | 1.710 |
| DM - nos | 2.148 | 0.177 | 9.26 | 0.000 | 1.827 | 2.525 |
|  |  |  |  |  |  |  |
| HF_b | 5.706 | 0.356 | 27.92 | 0.000 | 5.050 | 6.448 |
| AF_b | 1.835 | 0.068 | 16.46 | 0.000 | 1.707 | 1.973 |
| Heart_valve_dis_b | 1.399 | 0.137 | 3.43 | 0.001 | 1.155 | 1.695 |
| VT_VF_b | 1.390 | 0.216 | 2.12 | 0.034 | 1.025 | 1.884 |
| Cardiomyopathy_b | 1.983 | 0.300 | 4.52 | 0.000 | 1.474 | 2.669 |
| CV_procedures_b | 0.878 | 0.026 | -4.43 | 0.000 | 0.828 | 0.930 |
| TIA_stroke_b | 2.992 | 0.109 | 30.02 | 0.000 | 2.785 | 3.214 |
| PVD_b | 1.945 | 0.080 | 16.08 | 0.000 | 1.793 | 2.109 |
| CKD_b | 1.587 | 0.041 | 17.85 | 0.000 | 1.509 | 1.670 |
| Hypothyroidism_b | 1.020 | 0.035 | 0.60 | 0.550 | 0.955 | 1.090 |
| Liver_dis_b | 1.847 | 0.337 | 3.36 | 0.001 | 1.292 | 2.639 |
| Lupus_b | 1.648 | 0.252 | 3.27 | 0.001 | 1.222 | 2.223 |
| Erectile_dysfunction_b | 0.914 | 0.027 | -3.02 | 0.003 | 0.862 | 0.969 |
| Any_tumour_b | 1.330 | 0.033 | 11.38 | 0.000 | 1.266 | 1.397 |
| Menopause_b | 0.762 | 0.025 | -8.39 | 0.000 | 0.715 | 0.812 |
| Dementia_b | 3.844 | 0.376 | 13.78 | 0.000 | 3.174 | 4.656 |
| RA_b | 1.192 | 0.057 | 3.65 | 0.000 | 1.085 | 1.310 |
| _cons | 0.013 | 0.001 | -50.07 | 0.000 | 0.011 | 0.015 |

**Performance in validation dataset (20% of practices) - Internal validation - Index AMI cases**

| AUROC (95% CI): 0.780 (0.774; 0.787) |  |
| --- | --- |
|  |  |
|  | % Correctly classified: 71.41 |

### 7) 5-year Composite 1 CV outcome (HF, stroke, recurrent MI, & CV mortality)

|  | | | | | | |
| --- | --- | --- | --- | --- | --- | --- |
| _5yr_Comp1_CV_outcome | Odds ratio | Std. err. | z | P>|z| | [95% conf. interval] | |
| age | 1.054 | 0.001 | 65.50 | 0.000 | 1.053 | 1.056 |
|  |  |  |  |  |  |  |
| gender |  |  |  |  |  |  |
| Female | 1.053 | 0.022 | 2.43 | 0.015 | 1.010 | 1.097 |
|  |  |  |  |  |  |  |
| IMD |  |  |  |  |  |  |
| Q2 | 1.068 | 0.028 | 2.49 | 0.013 | 1.014 | 1.124 |
| Q3 | 1.148 | 0.030 | 5.22 | 0.000 | 1.090 | 1.209 |
| Q4 | 1.245 | 0.033 | 8.19 | 0.000 | 1.181 | 1.312 |
| Q5-most deprived | 1.412 | 0.038 | 12.66 | 0.000 | 1.338 | 1.489 |
|  |  |  |  |  |  |  |
| Other than White | 0.978 | 0.034 | -0.63 | 0.526 | 0.914 | 1.047 |
| Drinker | 0.861 | 0.020 | -6.43 | 0.000 | 0.822 | 0.901 |
|  |  |  |  |  |  |  |
| Smoking_status |  |  |  |  |  |  |
| current smoker | 1.225 | 0.031 | 7.94 | 0.000 | 1.165 | 1.288 |
| ex-smoker | 0.947 | 0.022 | -2.36 | 0.018 | 0.904 | 0.991 |
|  |  |  |  |  |  |  |
| BMI | 1.002 | 0.002 | 0.95 | 0.344 | 0.998 | 1.005 |
| HT_b | 1.102 | 0.020 | 5.41 | 0.000 | 1.064 | 1.142 |
| Hyperlipidaemia_b | 0.973 | 0.021 | -1.24 | 0.215 | 0.932 | 1.016 |
|  |  |  |  |  |  |  |
| Diabetes_type_b |  |  |  |  |  |  |
| T1DM | 3.067 | 0.300 | 11.44 | 0.000 | 2.531 | 3.716 |
| T2DM | 1.606 | 0.040 | 19.10 | 0.000 | 1.530 | 1.686 |
| DM - nos | 2.162 | 0.173 | 9.61 | 0.000 | 1.847 | 2.530 |
|  |  |  |  |  |  |  |
| HF_b | 5.779 | 0.337 | 30.10 | 0.000 | 5.155 | 6.478 |
| AF_b | 1.928 | 0.068 | 18.60 | 0.000 | 1.799 | 2.066 |
| Heart_valve_dis_b | 1.508 | 0.143 | 4.33 | 0.000 | 1.252 | 1.816 |
| VT_VF_b | 1.623 | 0.249 | 3.16 | 0.002 | 1.202 | 2.192 |
| Cardiomyopathy_b | 1.972 | 0.290 | 4.63 | 0.000 | 1.479 | 2.630 |
| CV_procedures_b | 0.908 | 0.026 | -3.30 | 0.001 | 0.858 | 0.962 |
| TIA_stroke_b | 3.279 | 0.115 | 33.83 | 0.000 | 3.061 | 3.512 |
| PVD_b | 1.962 | 0.078 | 17.02 | 0.000 | 1.816 | 2.121 |
| CKD_b | 1.558 | 0.039 | 17.89 | 0.000 | 1.484 | 1.635 |
| Hypothyroidism_b | 1.028 | 0.034 | 0.85 | 0.398 | 0.964 | 1.097 |
| Liver_dis_b | 2.128 | 0.385 | 4.17 | 0.000 | 1.493 | 3.033 |
| Lupus_b | 1.820 | 0.273 | 3.99 | 0.000 | 1.356 | 2.442 |
| Erectile_dysfunction_b | 0.963 | 0.028 | -1.27 | 0.203 | 0.909 | 1.021 |
| Any_tumour_b | 1.143 | 0.028 | 5.52 | 0.000 | 1.090 | 1.198 |
| Menopause_b | 0.764 | 0.025 | -8.38 | 0.000 | 0.717 | 0.814 |
| Dementia_b | 3.220 | 0.269 | 14.01 | 0.000 | 2.734 | 3.793 |
| RA_b | 1.108 | 0.052 | 2.19 | 0.029 | 1.011 | 1.215 |
| _cons | 0.015 | 0.001 | -49.12 | 0.000 | 0.012 | 0.017 |

**Performance in validation dataset (20% of practices) - Internal validation - Index AMI cases**

| AUROC (95% CI): 0.774 (0.767; 0.781) |  |
| --- | --- |
|  |  |
|  | % Correctly classified: 71.04 |

### 8) 5-year Composite 2 outcome (HF, stroke, & all-cause mortality)

|  | | | | | | |
| --- | --- | --- | --- | --- | --- | --- |
| _5yr_Comp2_outcome | Odds ratio | Std. err. | z | P>|z| | [95% conf. interval] | |
| age | 1.075 | 0.001 | 81.79 | 0.000 | 1.074 | 1.077 |
|  |  |  |  |  |  |  |
| gender |  |  |  |  |  |  |
| Female | 1.051 | 0.023 | 2.27 | 0.023 | 1.007 | 1.098 |
|  |  |  |  |  |  |  |
| IMD |  |  |  |  |  |  |
| Q2 | 1.111 | 0.031 | 3.79 | 0.000 | 1.052 | 1.173 |
| Q3 | 1.263 | 0.035 | 8.38 | 0.000 | 1.196 | 1.334 |
| Q4 | 1.385 | 0.039 | 11.51 | 0.000 | 1.310 | 1.464 |
| Q5-most deprived | 1.691 | 0.049 | 18.20 | 0.000 | 1.598 | 1.789 |
|  |  |  |  |  |  |  |
| Other than White | 0.946 | 0.035 | -1.51 | 0.132 | 0.880 | 1.017 |
| Drinker | 0.841 | 0.021 | -7.05 | 0.000 | 0.802 | 0.883 |
|  |  |  |  |  |  |  |
| Smoking_status |  |  |  |  |  |  |
| current smoker | 1.390 | 0.038 | 12.11 | 0.000 | 1.318 | 1.466 |
| ex-smoker | 0.956 | 0.024 | -1.82 | 0.069 | 0.911 | 1.003 |
|  |  |  |  |  |  |  |
| BMI | 0.998 | 0.002 | -1.38 | 0.168 | 0.994 | 1.001 |
| HT_b | 1.088 | 0.021 | 4.46 | 0.000 | 1.049 | 1.129 |
| Hyperlipidaemia_b | 0.936 | 0.022 | -2.89 | 0.004 | 0.894 | 0.979 |
|  |  |  |  |  |  |  |
| Diabetes_type_b |  |  |  |  |  |  |
| T1DM | 4.725 | 0.485 | 15.13 | 0.000 | 3.864 | 5.777 |
| T2DM | 1.742 | 0.045 | 21.42 | 0.000 | 1.656 | 1.833 |
| DM - nos | 2.457 | 0.204 | 10.84 | 0.000 | 2.089 | 2.891 |
|  |  |  |  |  |  |  |
| HF_b | 6.559 | 0.399 | 30.94 | 0.000 | 5.823 | 7.389 |
| AF_b | 1.912 | 0.070 | 17.80 | 0.000 | 1.780 | 2.053 |
| Heart_valve_dis_b | 1.387 | 0.134 | 3.38 | 0.001 | 1.148 | 1.677 |
| VT_VF_b | 1.618 | 0.257 | 3.03 | 0.002 | 1.185 | 2.208 |
| Cardiomyopathy_b | 2.449 | 0.372 | 5.89 | 0.000 | 1.817 | 3.299 |
| CV_procedures_b | 0.912 | 0.028 | -3.03 | 0.002 | 0.859 | 0.968 |
| TIA_stroke_b | 3.490 | 0.126 | 34.62 | 0.000 | 3.252 | 3.746 |
| PVD_b | 2.073 | 0.085 | 17.79 | 0.000 | 1.913 | 2.246 |
| CKD_b | 1.555 | 0.040 | 17.23 | 0.000 | 1.479 | 1.635 |
| Hypothyroidism_b | 1.015 | 0.035 | 0.42 | 0.672 | 0.949 | 1.085 |
| Liver_dis_b | 1.377 | 0.265 | 1.66 | 0.097 | 0.944 | 2.009 |
| Lupus_b | 1.880 | 0.296 | 4.01 | 0.000 | 1.381 | 2.560 |
| Erectile_dysfunction_b | 0.894 | 0.028 | -3.61 | 0.000 | 0.841 | 0.950 |
| Any_tumour_b | 1.442 | 0.036 | 14.52 | 0.000 | 1.373 | 1.515 |
| Menopause_b | 0.760 | 0.025 | -8.19 | 0.000 | 0.711 | 0.811 |
| Dementia_b | 4.091 | 0.387 | 14.91 | 0.000 | 3.399 | 4.923 |
| RA_b | 1.278 | 0.063 | 5.01 | 0.000 | 1.161 | 1.407 |
| _cons | 0.003 | 0.000 | -62.00 | 0.000 | 0.003 | 0.004 |

**Performance in validation dataset (20% of practices) - Internal validation - Index AMI cases**

| AUROC (95% CI): 0.821 (0.815; 0.827) |  |
| --- | --- |
|  |  |
|  | % Correctly classified: 74.70 |

### 9) 5-year Composite 2 CV outcome (HF, stroke, & CV mortality)

|  | | | | | | |
| --- | --- | --- | --- | --- | --- | --- |
| _5yr_Comp2_CV_outcome | Odds ratio | Std. err. | z | P>|z| | [95% conf. interval] | |
| age | 1.071 | 0.001 | 77.39 | 0.000 | 1.069 | 1.073 |
|  |  |  |  |  |  |  |
| gender |  |  |  |  |  |  |
| Female | 1.045 | 0.023 | 2.01 | 0.045 | 1.001 | 1.091 |
|  |  |  |  |  |  |  |
| IMD |  |  |  |  |  |  |
| Q2 | 1.097 | 0.030 | 3.33 | 0.001 | 1.039 | 1.158 |
| Q3 | 1.214 | 0.034 | 6.96 | 0.000 | 1.150 | 1.282 |
| Q4 | 1.363 | 0.039 | 10.95 | 0.000 | 1.289 | 1.440 |
| Q5-most deprived | 1.583 | 0.046 | 15.97 | 0.000 | 1.496 | 1.675 |
|  |  |  |  |  |  |  |
| Other than White | 0.973 | 0.036 | -0.74 | 0.461 | 0.905 | 1.047 |
| Drinker | 0.830 | 0.020 | -7.70 | 0.000 | 0.792 | 0.871 |
|  |  |  |  |  |  |  |
| Smoking_status |  |  |  |  |  |  |
| current smoker | 1.272 | 0.034 | 8.90 | 0.000 | 1.206 | 1.341 |
| ex-smoker | 0.940 | 0.023 | -2.55 | 0.011 | 0.896 | 0.986 |
|  |  |  |  |  |  |  |
| BMI | 1.003 | 0.002 | 1.61 | 0.108 | 0.999 | 1.006 |
| HT_b | 1.139 | 0.021 | 6.93 | 0.000 | 1.098 | 1.182 |
| Hyperlipidaemia_b | 0.979 | 0.022 | -0.95 | 0.344 | 0.936 | 1.023 |
|  |  |  |  |  |  |  |
| Diabetes_type_b |  |  |  |  |  |  |
| T1DM | 4.364 | 0.438 | 14.67 | 0.000 | 3.584 | 5.313 |
| T2DM | 1.727 | 0.044 | 21.53 | 0.000 | 1.643 | 1.815 |
| DM - nos | 2.225 | 0.178 | 9.99 | 0.000 | 1.902 | 2.603 |
|  |  |  |  |  |  |  |
| HF_b | 7.515 | 0.439 | 34.55 | 0.000 | 6.703 | 8.426 |
| AF_b | 1.936 | 0.067 | 19.07 | 0.000 | 1.809 | 2.072 |
| Heart_valve_dis_b | 1.476 | 0.138 | 4.15 | 0.000 | 1.228 | 1.774 |
| VT_VF_b | 1.795 | 0.279 | 3.76 | 0.000 | 1.323 | 2.436 |
| Cardiomyopathy_b | 2.212 | 0.323 | 5.44 | 0.000 | 1.662 | 2.944 |
| CV_procedures_b | 0.969 | 0.029 | -1.02 | 0.305 | 0.914 | 1.029 |
| TIA_stroke_b | 3.777 | 0.130 | 38.60 | 0.000 | 3.531 | 4.041 |
| PVD_b | 2.010 | 0.079 | 17.86 | 0.000 | 1.862 | 2.170 |
| CKD_b | 1.488 | 0.037 | 16.15 | 0.000 | 1.418 | 1.562 |
| Hypothyroidism_b | 0.991 | 0.033 | -0.28 | 0.779 | 0.927 | 1.058 |
| Liver_dis_b | 1.139 | 0.228 | 0.65 | 0.515 | 0.770 | 1.685 |
| Lupus_b | 2.128 | 0.331 | 4.86 | 0.000 | 1.569 | 2.886 |
| Erectile_dysfunction_b | 0.937 | 0.029 | -2.08 | 0.037 | 0.881 | 0.996 |
| Any_tumour_b | 1.146 | 0.028 | 5.56 | 0.000 | 1.092 | 1.202 |
| Menopause_b | 0.778 | 0.026 | -7.47 | 0.000 | 0.728 | 0.831 |
| Dementia_b | 3.572 | 0.292 | 15.58 | 0.000 | 3.043 | 4.192 |
| RA_b | 1.189 | 0.057 | 3.58 | 0.000 | 1.082 | 1.307 |
| _cons | 0.003 | 0.000 | -61.81 | 0.000 | 0.002 | 0.004 |

**Performance in validation dataset (20% of practices) - Internal validation - Index AMI cases**

| AUROC (95% CI): 0.812 (0.806; 0.818) |  |
| --- | --- |
|  |  |
|  | % Correctly classified: 74.40 |

## External validation (CPRD GOLD) - without therapies

### 1) 5-year all-cause mortality

|  | | | | | | |
| --- | --- | --- | --- | --- | --- | --- |
| _5year_All_cause_death | Odds ratio | Std. err. | z | P>|z| | [95% conf. interval] | |
| age | 1.102 | 0.001 | 89.21 | 0.000 | 1.099 | 1.104 |
|  |  |  |  |  |  |  |
| gender |  |  |  |  |  |  |
| Female | 1.004 | 0.023 | 0.19 | 0.846 | 0.960 | 1.051 |
|  |  |  |  |  |  |  |
| IMD |  |  |  |  |  |  |
| Q2 | 1.112 | 0.034 | 3.49 | 0.000 | 1.048 | 1.181 |
| Q3 | 1.232 | 0.038 | 6.83 | 0.000 | 1.161 | 1.308 |
| Q4 | 1.337 | 0.041 | 9.37 | 0.000 | 1.258 | 1.420 |
| Q5-most deprived | 1.509 | 0.048 | 13.05 | 0.000 | 1.418 | 1.605 |
| unknown | 0.674 | 0.250 | -1.07 | 0.287 | 0.326 | 1.393 |
|  |  |  |  |  |  |  |
| Other than White | 1.077 | 0.041 | 1.96 | 0.050 | 1.000 | 1.160 |
| Drinker | 1.028 | 0.010 | 2.75 | 0.006 | 1.008 | 1.049 |
|  |  |  |  |  |  |  |
| Smoking_status |  |  |  |  |  |  |
| current smoker | 1.692 | 0.051 | 17.58 | 0.000 | 1.596 | 1.794 |
| ex-smoker | 0.993 | 0.026 | -0.27 | 0.784 | 0.943 | 1.045 |
| unknown | 1.747 | 0.233 | 4.18 | 0.000 | 1.344 | 2.269 |
|  |  |  |  |  |  |  |
| BMI | 0.981 | 0.002 | -9.93 | 0.000 | 0.977 | 0.985 |
| HT_b | 1.014 | 0.021 | 0.66 | 0.508 | 0.974 | 1.055 |
| Hyperlipidaemia_b | 0.885 | 0.022 | -4.99 | 0.000 | 0.843 | 0.928 |
|  |  |  |  |  |  |  |
| Diabetes_type_b |  |  |  |  |  |  |
| T1DM | 5.972 | 0.628 | 16.99 | 0.000 | 4.859 | 7.340 |
| T2DM | 1.731 | 0.046 | 20.80 | 0.000 | 1.644 | 1.823 |
| DM - nos | 2.681 | 0.211 | 12.54 | 0.000 | 2.298 | 3.128 |
|  |  |  |  |  |  |  |
| HF_b | 2.379 | 0.092 | 22.41 | 0.000 | 2.205 | 2.566 |
| AF_b | 1.524 | 0.048 | 13.50 | 0.000 | 1.434 | 1.620 |
| Heart_valve_dis_b | 1.503 | 0.126 | 4.88 | 0.000 | 1.276 | 1.771 |
| VT_VF_b | 1.369 | 0.204 | 2.11 | 0.035 | 1.022 | 1.834 |
| Cardiomyopathy_b | 1.081 | 0.141 | 0.60 | 0.548 | 0.838 | 1.396 |
| CV_procedures_b | 0.856 | 0.028 | -4.73 | 0.000 | 0.803 | 0.913 |
| TIA_stroke_b | 1.599 | 0.047 | 16.00 | 0.000 | 1.510 | 1.694 |
| PVD_b | 2.018 | 0.074 | 19.11 | 0.000 | 1.878 | 2.169 |
| CKD_b | 1.544 | 0.037 | 18.21 | 0.000 | 1.473 | 1.618 |
| Hypothyroidism_b | 0.992 | 0.034 | -0.24 | 0.810 | 0.928 | 1.060 |
| Liver_dis_b | 1.873 | 0.413 | 2.85 | 0.004 | 1.216 | 2.884 |
| Lupus_b | 1.640 | 0.270 | 3.00 | 0.003 | 1.187 | 2.265 |
| Erectile_dysfunction_b | 0.799 | 0.029 | -6.13 | 0.000 | 0.744 | 0.859 |
| Any_tumour_b | 1.618 | 0.040 | 19.54 | 0.000 | 1.541 | 1.698 |
| Menopause_b | 0.783 | 0.029 | -6.62 | 0.000 | 0.728 | 0.842 |
| Dementia_b | 4.021 | 0.281 | 19.93 | 0.000 | 3.507 | 4.611 |
| RA_b | 1.436 | 0.072 | 7.21 | 0.000 | 1.301 | 1.584 |
| _cons | 0.000 | 0.000 | -75.18 | 0.000 | 0.000 | 0.000 |

**Performance in validation dataset (CPRD GOLD) - External validation - Index AMI cases**

| AUROC (95% CI): 0.837 (0.831; 0.843) |  |
| --- | --- |
|  |  |
|  | % Correctly classified: 76.91 |

### 2) 5-year Stroke

|  | | | | | | |
| --- | --- | --- | --- | --- | --- | --- |
| _5year_Stroke | Odds ratio | Std. err. | z | P>|z| | [95% conf. interval] | |
| age | 1.069 | 0.001 | 60.15 | 0.000 | 1.067 | 1.071 |
|  |  |  |  |  |  |  |
| gender |  |  |  |  |  |  |
| Female | 1.135 | 0.028 | 5.12 | 0.000 | 1.081 | 1.192 |
|  |  |  |  |  |  |  |
| IMD |  |  |  |  |  |  |
| Q2 | 1.017 | 0.033 | 0.52 | 0.600 | 0.954 | 1.085 |
| Q3 | 1.101 | 0.036 | 2.93 | 0.003 | 1.032 | 1.174 |
| Q4 | 1.211 | 0.040 | 5.79 | 0.000 | 1.135 | 1.293 |
| Q5-most deprived | 1.297 | 0.044 | 7.71 | 0.000 | 1.214 | 1.386 |
|  |  |  |  |  |  |  |
| Other than White | 1.002 | 0.046 | 0.05 | 0.961 | 0.916 | 1.096 |
| Drinker | 0.870 | 0.023 | -5.19 | 0.000 | 0.826 | 0.917 |
|  |  |  |  |  |  |  |
| Smoking_status |  |  |  |  |  |  |
| current smoker | 1.206 | 0.038 | 5.94 | 0.000 | 1.134 | 1.283 |
| ex-smoker | 0.911 | 0.025 | -3.37 | 0.001 | 0.863 | 0.962 |
|  |  |  |  |  |  |  |
| BMI | 0.985 | 0.002 | -7.07 | 0.000 | 0.981 | 0.989 |
| HT_b | 1.226 | 0.026 | 9.44 | 0.000 | 1.175 | 1.279 |
| Hyperlipidaemia_b | 1.067 | 0.027 | 2.54 | 0.011 | 1.015 | 1.123 |
|  |  |  |  |  |  |  |
| Diabetes_type_b |  |  |  |  |  |  |
| T1DM | 3.639 | 0.386 | 12.18 | 0.000 | 2.956 | 4.480 |
| T2DM | 1.629 | 0.044 | 17.89 | 0.000 | 1.545 | 1.719 |
| DM - nos | 1.753 | 0.141 | 6.96 | 0.000 | 1.497 | 2.053 |
|  |  |  |  |  |  |  |
| HF_b | 1.525 | 0.056 | 11.41 | 0.000 | 1.419 | 1.640 |
| AF_b | 1.720 | 0.054 | 17.41 | 0.000 | 1.618 | 1.828 |
| Heart_valve_dis_b | 1.362 | 0.112 | 3.75 | 0.000 | 1.159 | 1.600 |
| VT_VF_b | 1.065 | 0.164 | 0.41 | 0.680 | 0.789 | 1.439 |
| Cardiomyopathy_b | 1.232 | 0.163 | 1.58 | 0.114 | 0.951 | 1.597 |
| CV_procedures_b | 0.890 | 0.031 | -3.34 | 0.001 | 0.831 | 0.953 |
| TIA_stroke_b | 7.798 | 0.233 | 68.64 | 0.000 | 7.354 | 8.269 |
| PVD_b | 1.936 | 0.071 | 18.00 | 0.000 | 1.802 | 2.080 |
| CKD_b | 1.355 | 0.033 | 12.31 | 0.000 | 1.291 | 1.422 |
| Hypothyroidism_b | 1.019 | 0.036 | 0.54 | 0.588 | 0.951 | 1.093 |
| Liver_dis_b | 1.500 | 0.365 | 1.67 | 0.096 | 0.931 | 2.418 |
| Lupus_b | 1.511 | 0.263 | 2.37 | 0.018 | 1.075 | 2.124 |
| Erectile_dysfunction_b | 1.106 | 0.042 | 2.62 | 0.009 | 1.026 | 1.193 |
| Any_tumour_b | 1.117 | 0.029 | 4.20 | 0.000 | 1.061 | 1.176 |
| Menopause_b | 0.861 | 0.034 | -3.81 | 0.000 | 0.797 | 0.930 |
| Dementia_b | 3.188 | 0.188 | 19.63 | 0.000 | 2.839 | 3.579 |
| RA_b | 0.894 | 0.050 | -1.99 | 0.047 | 0.801 | 0.998 |
| _cons | 0.001 | 0.000 | -55.92 | 0.000 | 0.001 | 0.002 |

**Performance in validation dataset (CPRD GOLD) - External validation - Index AMI cases**

| AUROC (95% CI): 0.827 (0.820; 0.834) |  |
| --- | --- |
|  |  |
|  | % Correctly classified: 77.95 |

### 3) 5-year heart failure (HF)

|  | | | | | | |
| --- | --- | --- | --- | --- | --- | --- |
| _5year_HF | Odds ratio | Std. err. | z | P>|z| | [95% conf. interval] | |
| age | 1.067 | 0.001 | 70.59 | 0.000 | 1.065 | 1.069 |
|  |  |  |  |  |  |  |
| gender |  |  |  |  |  |  |
| Female | 1.007 | 0.022 | 0.33 | 0.740 | 0.965 | 1.051 |
|  |  |  |  |  |  |  |
| IMD |  |  |  |  |  |  |
| Q2 | 1.084 | 0.031 | 2.83 | 0.005 | 1.025 | 1.146 |
| Q3 | 1.139 | 0.033 | 4.56 | 0.000 | 1.077 | 1.205 |
| Q4 | 1.302 | 0.038 | 9.16 | 0.000 | 1.231 | 1.378 |
| Q5-most deprived | 1.587 | 0.046 | 15.79 | 0.000 | 1.498 | 1.680 |
|  |  |  |  |  |  |  |
| Other than White | 1.050 | 0.040 | 1.28 | 0.200 | 0.974 | 1.132 |
| Drinker | 0.838 | 0.020 | -7.42 | 0.000 | 0.799 | 0.878 |
|  |  |  |  |  |  |  |
| Smoking_status |  |  |  |  |  |  |
| current smoker | 1.322 | 0.036 | 10.13 | 0.000 | 1.253 | 1.396 |
| ex-smoker | 1.012 | 0.025 | 0.49 | 0.626 | 0.964 | 1.062 |
|  |  |  |  |  |  |  |
| BMI | 1.011 | 0.002 | 6.00 | 0.000 | 1.007 | 1.014 |
| HT_b | 1.084 | 0.021 | 4.25 | 0.000 | 1.044 | 1.125 |
| Hyperlipidaemia_b | 0.953 | 0.022 | -2.09 | 0.037 | 0.911 | 0.997 |
|  |  |  |  |  |  |  |
| Diabetes_type_b |  |  |  |  |  |  |
| T1DM | 3.804 | 0.370 | 13.74 | 0.000 | 3.144 | 4.602 |
| T2DM | 1.710 | 0.042 | 21.75 | 0.000 | 1.629 | 1.795 |
| DM - nos | 2.499 | 0.189 | 12.13 | 0.000 | 2.155 | 2.898 |
|  |  |  |  |  |  |  |
| HF_b | 9.124 | 0.457 | 44.19 | 0.000 | 8.272 | 10.064 |
| AF_b | 1.974 | 0.061 | 21.96 | 0.000 | 1.858 | 2.098 |
| Heart_valve_dis_b | 1.751 | 0.150 | 6.55 | 0.000 | 1.481 | 2.071 |
| VT_VF_b | 1.701 | 0.247 | 3.66 | 0.000 | 1.280 | 2.261 |
| Cardiomyopathy_b | 2.242 | 0.304 | 5.96 | 0.000 | 1.719 | 2.923 |
| CV_procedures_b | 1.030 | 0.031 | 0.97 | 0.331 | 0.970 | 1.093 |
| TIA_stroke_b | 1.289 | 0.037 | 8.95 | 0.000 | 1.220 | 1.363 |
| PVD_b | 1.763 | 0.063 | 15.96 | 0.000 | 1.644 | 1.890 |
| CKD_b | 1.535 | 0.035 | 18.55 | 0.000 | 1.467 | 1.606 |
| Hypothyroidism_b | 0.958 | 0.031 | -1.32 | 0.188 | 0.898 | 1.021 |
| Liver_dis_b | 1.488 | 0.301 | 1.97 | 0.049 | 1.001 | 2.212 |
| Lupus_b | 1.879 | 0.284 | 4.17 | 0.000 | 1.397 | 2.526 |
| Erectile_dysfunction_b | 0.976 | 0.032 | -0.75 | 0.451 | 0.916 | 1.040 |
| Any_tumour_b | 1.176 | 0.028 | 6.79 | 0.000 | 1.122 | 1.233 |
| Menopause_b | 0.779 | 0.027 | -7.21 | 0.000 | 0.728 | 0.834 |
| Dementia_b | 1.777 | 0.103 | 9.87 | 0.000 | 1.585 | 1.992 |
| RA_b | 1.205 | 0.058 | 3.89 | 0.000 | 1.097 | 1.324 |
| _cons | 0.002 | 0.000 | -64.00 | 0.000 | 0.002 | 0.002 |

**Performance in validation dataset (CPRD GOLD) - External validation - Index AMI cases**

| AUROC (95% CI): 0.776 (0.769; 0.783) |  |
| --- | --- |
|  |  |
|  | % Correctly classified: 71.50 |

### 4) 5-year Recurrent MI

|  | | | | | | |
| --- | --- | --- | --- | --- | --- | --- |
| _5year_Recurrent_MI | Odds ratio | Std. err. | z | P>|z| | [95% conf. interval] | |
| age | 1.033 | 0.001 | 36.70 | 0.000 | 1.031 | 1.035 |
|  |  |  |  |  |  |  |
| gender |  |  |  |  |  |  |
| Female | 1.025 | 0.022 | 1.11 | 0.268 | 0.981 | 1.070 |
|  |  |  |  |  |  |  |
| IMD |  |  |  |  |  |  |
| Q2 | 1.029 | 0.029 | 1.01 | 0.312 | 0.973 | 1.089 |
| Q3 | 1.067 | 0.031 | 2.26 | 0.024 | 1.009 | 1.129 |
| Q4 | 1.085 | 0.031 | 2.81 | 0.005 | 1.025 | 1.148 |
| Q5-most deprived | 1.166 | 0.034 | 5.23 | 0.000 | 1.101 | 1.235 |
|  |  |  |  |  |  |  |
| Other than White | 1.076 | 0.041 | 1.92 | 0.055 | 0.998 | 1.160 |
| Drinker | 0.913 | 0.022 | -3.84 | 0.000 | 0.871 | 0.956 |
|  |  |  |  |  |  |  |
| Smoking_status |  |  |  |  |  |  |
| current smoker | 1.177 | 0.032 | 5.93 | 0.000 | 1.115 | 1.242 |
| ex-smoker | 0.940 | 0.023 | -2.51 | 0.012 | 0.896 | 0.987 |
|  |  |  |  |  |  |  |
| BMI | 0.994 | 0.002 | -3.28 | 0.001 | 0.991 | 0.998 |
| HT_b | 1.066 | 0.020 | 3.33 | 0.001 | 1.027 | 1.107 |
| Hyperlipidaemia_b | 0.991 | 0.023 | -0.40 | 0.689 | 0.947 | 1.036 |
|  |  |  |  |  |  |  |
| Diabetes_type_b |  |  |  |  |  |  |
| T1DM | 2.659 | 0.243 | 10.71 | 0.000 | 2.223 | 3.180 |
| T2DM | 1.302 | 0.032 | 10.70 | 0.000 | 1.240 | 1.366 |
| DM - nos | 1.732 | 0.122 | 7.78 | 0.000 | 1.508 | 1.989 |
|  |  |  |  |  |  |  |
| HF_b | 1.614 | 0.054 | 14.28 | 0.000 | 1.512 | 1.724 |
| AF_b | 1.161 | 0.034 | 5.13 | 0.000 | 1.097 | 1.230 |
| Heart_valve_dis_b | 1.368 | 0.101 | 4.23 | 0.000 | 1.183 | 1.582 |
| VT_VF_b | 0.776 | 0.113 | -1.74 | 0.081 | 0.584 | 1.032 |
| Cardiomyopathy_b | 0.910 | 0.113 | -0.77 | 0.444 | 0.714 | 1.159 |
| CV_procedures_b | 0.980 | 0.030 | -0.65 | 0.519 | 0.923 | 1.041 |
| TIA_stroke_b | 1.287 | 0.035 | 9.23 | 0.000 | 1.220 | 1.358 |
| PVD_b | 1.586 | 0.053 | 13.81 | 0.000 | 1.486 | 1.694 |
| CKD_b | 1.353 | 0.031 | 13.28 | 0.000 | 1.294 | 1.415 |
| Hypothyroidism_b | 1.067 | 0.034 | 2.02 | 0.044 | 1.002 | 1.137 |
| Liver_dis_b | 1.738 | 0.331 | 2.90 | 0.004 | 1.196 | 2.525 |
| Lupus_b | 1.496 | 0.222 | 2.71 | 0.007 | 1.118 | 2.003 |
| Erectile_dysfunction_b | 0.854 | 0.029 | -4.65 | 0.000 | 0.799 | 0.913 |
| Any_tumour_b | 1.129 | 0.027 | 5.08 | 0.000 | 1.078 | 1.184 |
| Menopause_b | 0.728 | 0.026 | -8.79 | 0.000 | 0.678 | 0.781 |
| Dementia_b | 2.072 | 0.108 | 14.04 | 0.000 | 1.872 | 2.294 |
| RA_b | 0.958 | 0.048 | -0.85 | 0.393 | 0.869 | 1.057 |
| _cons | 0.025 | 0.002 | -38.92 | 0.000 | 0.021 | 0.030 |

**Performance in validation dataset (CPRD GOLD) - External validation - Index AMI cases**

| AUROC (95% CI): 0.646 (0.638; 0.654) |  |
| --- | --- |
|  |  |
|  | % Correctly classified: 58.04 |

### 5) 5-year CV mortality

|  | | | | | | |
| --- | --- | --- | --- | --- | --- | --- |
| _5year_CV_death | Odds ratio | Std. err. | z | P>|z| | [95% conf. interval] | |
| age | 1.106 | 0.001 | 74.37 | 0.000 | 1.103 | 1.108 |
|  |  |  |  |  |  |  |
| gender |  |  |  |  |  |  |
| Female | 0.883 | 0.023 | -4.69 | 0.000 | 0.839 | 0.930 |
|  |  |  |  |  |  |  |
| IMD |  |  |  |  |  |  |
| Q2 | 1.159 | 0.041 | 4.14 | 0.000 | 1.081 | 1.243 |
| Q3 | 1.284 | 0.046 | 7.01 | 0.000 | 1.197 | 1.377 |
| Q4 | 1.393 | 0.050 | 9.18 | 0.000 | 1.298 | 1.495 |
| Q5-most deprived | 1.465 | 0.054 | 10.32 | 0.000 | 1.363 | 1.576 |
|  |  |  |  |  |  |  |
| Other than White | 0.813 | 0.043 | -3.87 | 0.000 | 0.732 | 0.903 |
| Drinker | 0.763 | 0.022 | -9.61 | 0.000 | 0.722 | 0.806 |
|  |  |  |  |  |  |  |
| Smoking_status |  |  |  |  |  |  |
| current smoker | 1.360 | 0.046 | 9.01 | 0.000 | 1.272 | 1.455 |
| ex-smoker | 0.868 | 0.026 | -4.76 | 0.000 | 0.819 | 0.920 |
|  |  |  |  |  |  |  |
| BMI | 0.990 | 0.002 | -4.48 | 0.000 | 0.986 | 0.994 |
| HT_b | 1.060 | 0.025 | 2.51 | 0.012 | 1.013 | 1.110 |
| Hyperlipidaemia_b | 0.949 | 0.027 | -1.87 | 0.061 | 0.898 | 1.002 |
|  |  |  |  |  |  |  |
| Diabetes_type_b |  |  |  |  |  |  |
| T1DM | 8.377 | 0.932 | 19.11 | 0.000 | 6.736 | 10.418 |
| T2DM | 1.862 | 0.054 | 21.35 | 0.000 | 1.759 | 1.972 |
| DM - nos | 2.916 | 0.241 | 12.94 | 0.000 | 2.479 | 3.429 |
|  |  |  |  |  |  |  |
| HF_b | 2.993 | 0.110 | 29.76 | 0.000 | 2.784 | 3.217 |
| AF_b | 1.611 | 0.051 | 15.05 | 0.000 | 1.514 | 1.714 |
| Heart_valve_dis_b | 1.912 | 0.158 | 7.84 | 0.000 | 1.626 | 2.248 |
| VT_VF_b | 1.215 | 0.192 | 1.24 | 0.216 | 0.892 | 1.656 |
| Cardiomyopathy_b | 1.262 | 0.176 | 1.67 | 0.095 | 0.960 | 1.659 |
| CV_procedures_b | 0.980 | 0.037 | -0.52 | 0.600 | 0.911 | 1.055 |
| TIA_stroke_b | 1.614 | 0.049 | 15.79 | 0.000 | 1.521 | 1.713 |
| PVD_b | 2.168 | 0.082 | 20.56 | 0.000 | 2.013 | 2.333 |
| CKD_b | 1.403 | 0.036 | 13.24 | 0.000 | 1.334 | 1.475 |
| Hypothyroidism_b | 0.970 | 0.037 | -0.82 | 0.414 | 0.901 | 1.044 |
| Liver_dis_b | 1.817 | 0.503 | 2.16 | 0.031 | 1.056 | 3.125 |
| Lupus_b | 2.093 | 0.381 | 4.06 | 0.000 | 1.466 | 2.989 |
| Erectile_dysfunction_b | 0.785 | 0.035 | -5.37 | 0.000 | 0.718 | 0.857 |
| Any_tumour_b | 1.045 | 0.029 | 1.59 | 0.111 | 0.990 | 1.103 |
| Menopause_b | 0.695 | 0.032 | -7.97 | 0.000 | 0.636 | 0.760 |
| Dementia_b | 3.500 | 0.202 | 21.68 | 0.000 | 3.125 | 3.920 |
| RA_b | 1.500 | 0.084 | 7.23 | 0.000 | 1.344 | 1.675 |
| _cons | 0.000 | 0.000 | -67.06 | 0.000 | 0.000 | 0.000 |

**Performance in validation dataset (CPRD GOLD) - External validation - Index AMI cases**

| AUROC (95% CI): 0.851 (0.843; 0.857) |  |
| --- | --- |
|  |  |
|  | % Correctly classified: 80.05 |

### 6) 5-year Composite 1 outcome (HF, stroke, recurrent MI, & all-cause mortality)

|  | | | | | | |
| --- | --- | --- | --- | --- | --- | --- |
| _5yr_Comp1_outcome | Odds ratio | Std. err. | z | P>|z| | [95% conf. interval] | |
| age | 1.060 | 0.001 | 71.10 | 0.000 | 1.059 | 1.062 |
|  |  |  |  |  |  |  |
| gender |  |  |  |  |  |  |
| Female | 1.059 | 0.023 | 2.68 | 0.007 | 1.016 | 1.105 |
|  |  |  |  |  |  |  |
| IMD |  |  |  |  |  |  |
| Q2 | 1.076 | 0.029 | 2.75 | 0.006 | 1.021 | 1.133 |
| Q3 | 1.197 | 0.032 | 6.74 | 0.000 | 1.136 | 1.261 |
| Q4 | 1.309 | 0.035 | 9.95 | 0.000 | 1.241 | 1.380 |
| Q5-most deprived | 1.499 | 0.041 | 14.68 | 0.000 | 1.420 | 1.583 |
|  |  |  |  |  |  |  |
| Other than White | 0.975 | 0.034 | -0.73 | 0.467 | 0.911 | 1.044 |
| Drinker | 0.865 | 0.021 | -6.12 | 0.000 | 0.825 | 0.906 |
|  |  |  |  |  |  |  |
| Smoking_status |  |  |  |  |  |  |
| current smoker | 1.302 | 0.034 | 10.16 | 0.000 | 1.238 | 1.371 |
| ex-smoker | 0.952 | 0.023 | -2.09 | 0.036 | 0.908 | 0.997 |
|  |  |  |  |  |  |  |
| BMI | 0.996 | 0.002 | -2.22 | 0.026 | 0.993 | 1.000 |
| HT_b | 1.081 | 0.020 | 4.26 | 0.000 | 1.043 | 1.121 |
| Hyperlipidaemia_b | 0.952 | 0.021 | -2.20 | 0.028 | 0.911 | 0.995 |
|  |  |  |  |  |  |  |
| Diabetes_type_b |  |  |  |  |  |  |
| T1DM | 3.567 | 0.361 | 12.56 | 0.000 | 2.925 | 4.350 |
| T2DM | 1.627 | 0.041 | 19.11 | 0.000 | 1.548 | 1.710 |
| DM - nos | 2.148 | 0.177 | 9.26 | 0.000 | 1.827 | 2.525 |
|  |  |  |  |  |  |  |
| HF_b | 5.706 | 0.356 | 27.92 | 0.000 | 5.050 | 6.448 |
| AF_b | 1.835 | 0.068 | 16.46 | 0.000 | 1.707 | 1.973 |
| Heart_valve_dis_b | 1.399 | 0.137 | 3.43 | 0.001 | 1.155 | 1.695 |
| VT_VF_b | 1.390 | 0.216 | 2.12 | 0.034 | 1.025 | 1.884 |
| Cardiomyopathy_b | 1.983 | 0.300 | 4.52 | 0.000 | 1.474 | 2.669 |
| CV_procedures_b | 0.878 | 0.026 | -4.43 | 0.000 | 0.828 | 0.930 |
| TIA_stroke_b | 2.992 | 0.109 | 30.02 | 0.000 | 2.785 | 3.214 |
| PVD_b | 1.945 | 0.080 | 16.08 | 0.000 | 1.793 | 2.109 |
| CKD_b | 1.587 | 0.041 | 17.85 | 0.000 | 1.509 | 1.670 |
| Hypothyroidism_b | 1.020 | 0.035 | 0.60 | 0.550 | 0.955 | 1.090 |
| Liver_dis_b | 1.847 | 0.337 | 3.36 | 0.001 | 1.292 | 2.639 |
| Lupus_b | 1.648 | 0.252 | 3.27 | 0.001 | 1.222 | 2.223 |
| Erectile_dysfunction_b | 0.914 | 0.027 | -3.02 | 0.003 | 0.862 | 0.969 |
| Any_tumour_b | 1.330 | 0.033 | 11.38 | 0.000 | 1.266 | 1.397 |
| Menopause_b | 0.762 | 0.025 | -8.39 | 0.000 | 0.715 | 0.812 |
| Dementia_b | 3.844 | 0.376 | 13.78 | 0.000 | 3.174 | 4.656 |
| RA_b | 1.192 | 0.057 | 3.65 | 0.000 | 1.085 | 1.310 |
| _cons | 0.013 | 0.001 | -50.07 | 0.000 | 0.011 | 0.015 |

**Performance in validation dataset (CPRD GOLD) - External validation - Index AMI cases**

| AUROC (95% CI): 0.724 (0.715; 0.733) |  |
| --- | --- |
|  |  |
|  | % Correctly classified: 64.27 |

### 7) 5-year Composite 1 CV outcome (HF, stroke, recurrent MI, & CV mortality)

|  | | | | | | |
| --- | --- | --- | --- | --- | --- | --- |
| _5yr_Comp1_CV_outcome | Odds ratio | Std. err. | z | P>|z| | [95% conf. interval] | |
| age | 1.056 | 0.001 | 67.76 | 0.000 | 1.055 | 1.058 |
|  |  |  |  |  |  |  |
| gender |  |  |  |  |  |  |
| Female | 1.051 | 0.022 | 2.36 | 0.018 | 1.008 | 1.095 |
|  |  |  |  |  |  |  |
| IMD |  |  |  |  |  |  |
| Q2 | 1.056 | 0.028 | 2.07 | 0.039 | 1.003 | 1.112 |
| Q3 | 1.145 | 0.030 | 5.11 | 0.000 | 1.087 | 1.205 |
| Q4 | 1.256 | 0.034 | 8.53 | 0.000 | 1.192 | 1.324 |
| Q5-most deprived | 1.465 | 0.040 | 14.07 | 0.000 | 1.389 | 1.545 |
| unknown | 1.305 | 0.372 | 0.93 | 0.351 | 0.746 | 2.281 |
|  |  |  |  |  |  |  |
| Other than White | 1.000 | 0.031 | 0.01 | 0.990 | 0.941 | 1.063 |
| Drinker | 1.009 | 0.009 | 1.04 | 0.299 | 0.992 | 1.027 |
|  |  |  |  |  |  |  |
| Smoking_status |  |  |  |  |  |  |
| current smoker | 1.242 | 0.032 | 8.45 | 0.000 | 1.181 | 1.307 |
| ex-smoker | 0.935 | 0.022 | -2.88 | 0.004 | 0.893 | 0.979 |
| unknown | 1.434 | 0.145 | 3.55 | 0.000 | 1.175 | 1.749 |
|  |  |  |  |  |  |  |
| BMI | 1.001 | 0.002 | 0.51 | 0.607 | 0.998 | 1.004 |
| HT_b | 1.100 | 0.020 | 5.27 | 0.000 | 1.062 | 1.140 |
| Hyperlipidaemia_b | 0.980 | 0.022 | -0.91 | 0.365 | 0.939 | 1.023 |
|  |  |  |  |  |  |  |
| Diabetes_type_b |  |  |  |  |  |  |
| T1DM | 3.268 | 0.322 | 12.02 | 0.000 | 2.694 | 3.965 |
| T2DM | 1.636 | 0.041 | 19.82 | 0.000 | 1.558 | 1.717 |
| DM - nos | 2.078 | 0.166 | 9.16 | 0.000 | 1.777 | 2.430 |
|  |  |  |  |  |  |  |
| HF_b | 6.268 | 0.377 | 30.53 | 0.000 | 5.571 | 7.052 |
| AF_b | 1.845 | 0.065 | 17.40 | 0.000 | 1.722 | 1.977 |
| Heart_valve_dis_b | 1.476 | 0.140 | 4.11 | 0.000 | 1.226 | 1.777 |
| VT_VF_b | 1.437 | 0.218 | 2.39 | 0.017 | 1.068 | 1.935 |
| Cardiomyopathy_b | 2.247 | 0.339 | 5.37 | 0.000 | 1.672 | 3.019 |
| CV_procedures_b | 0.917 | 0.027 | -2.97 | 0.003 | 0.866 | 0.971 |
| TIA_stroke_b | 3.220 | 0.113 | 33.36 | 0.000 | 3.007 | 3.449 |
| PVD_b | 1.928 | 0.076 | 16.55 | 0.000 | 1.783 | 2.083 |
| CKD_b | 1.578 | 0.039 | 18.35 | 0.000 | 1.503 | 1.657 |
| Hypothyroidism_b | 1.011 | 0.033 | 0.33 | 0.741 | 0.948 | 1.078 |
| Liver_dis_b | 1.629 | 0.295 | 2.69 | 0.007 | 1.142 | 2.322 |
| Lupus_b | 1.606 | 0.241 | 3.16 | 0.002 | 1.198 | 2.154 |
| Erectile_dysfunction_b | 0.946 | 0.028 | -1.88 | 0.060 | 0.892 | 1.002 |
| Any_tumour_b | 1.138 | 0.028 | 5.34 | 0.000 | 1.085 | 1.193 |
| Menopause_b | 0.772 | 0.025 | -8.05 | 0.000 | 0.725 | 0.822 |
| Dementia_b | 2.786 | 0.223 | 12.78 | 0.000 | 2.381 | 3.261 |
| RA_b | 1.101 | 0.052 | 2.04 | 0.041 | 1.004 | 1.207 |
| _cons | 0.011 | 0.001 | -53.41 | 0.000 | 0.010 | 0.013 |

**Performance in validation dataset (CPRD GOLD) - External validation - Index AMI cases**

| AUROC (95% CI): 0.716 (0.707; 0.724) |  |
| --- | --- |
|  |  |
|  | % Correctly classified: 63.21 |

### 8) 5-year Composite 2 outcome (HF, stroke, & all-cause mortality)

|  | | | | | | |
| --- | --- | --- | --- | --- | --- | --- |
| _5yr_Comp2_outcome | Odds ratio | Std. err. | z | P>|z| | [95% conf. interval] | |
| age | 1.075 | 0.001 | 81.79 | 0.000 | 1.074 | 1.077 |
|  |  |  |  |  |  |  |
| gender |  |  |  |  |  |  |
| Female | 1.051 | 0.023 | 2.27 | 0.023 | 1.007 | 1.098 |
|  |  |  |  |  |  |  |
| IMD |  |  |  |  |  |  |
| Q2 | 1.111 | 0.031 | 3.79 | 0.000 | 1.052 | 1.173 |
| Q3 | 1.263 | 0.035 | 8.38 | 0.000 | 1.196 | 1.334 |
| Q4 | 1.385 | 0.039 | 11.51 | 0.000 | 1.310 | 1.464 |
| Q5-most deprived | 1.691 | 0.049 | 18.20 | 0.000 | 1.598 | 1.789 |
|  |  |  |  |  |  |  |
| Other than White | 0.946 | 0.035 | -1.51 | 0.132 | 0.880 | 1.017 |
| Drinker | 0.841 | 0.021 | -7.05 | 0.000 | 0.802 | 0.883 |
|  |  |  |  |  |  |  |
| Smoking_status |  |  |  |  |  |  |
| current smoker | 1.390 | 0.038 | 12.11 | 0.000 | 1.318 | 1.466 |
| ex-smoker | 0.956 | 0.024 | -1.82 | 0.069 | 0.911 | 1.003 |
|  |  |  |  |  |  |  |
| BMI | 0.998 | 0.002 | -1.38 | 0.168 | 0.994 | 1.001 |
| HT_b | 1.088 | 0.021 | 4.46 | 0.000 | 1.049 | 1.129 |
| Hyperlipidaemia_b | 0.936 | 0.022 | -2.89 | 0.004 | 0.894 | 0.979 |
|  |  |  |  |  |  |  |
| Diabetes_type_b |  |  |  |  |  |  |
| T1DM | 4.725 | 0.485 | 15.13 | 0.000 | 3.864 | 5.777 |
| T2DM | 1.742 | 0.045 | 21.42 | 0.000 | 1.656 | 1.833 |
| DM - nos | 2.457 | 0.204 | 10.84 | 0.000 | 2.089 | 2.891 |
|  |  |  |  |  |  |  |
| HF_b | 6.559 | 0.399 | 30.94 | 0.000 | 5.823 | 7.389 |
| AF_b | 1.912 | 0.070 | 17.80 | 0.000 | 1.780 | 2.053 |
| Heart_valve_dis_b | 1.387 | 0.134 | 3.38 | 0.001 | 1.148 | 1.677 |
| VT_VF_b | 1.618 | 0.257 | 3.03 | 0.002 | 1.185 | 2.208 |
| Cardiomyopathy_b | 2.449 | 0.372 | 5.89 | 0.000 | 1.817 | 3.299 |
| CV_procedures_b | 0.912 | 0.028 | -3.03 | 0.002 | 0.859 | 0.968 |
| TIA_stroke_b | 3.490 | 0.126 | 34.62 | 0.000 | 3.252 | 3.746 |
| PVD_b | 2.073 | 0.085 | 17.79 | 0.000 | 1.913 | 2.246 |
| CKD_b | 1.555 | 0.040 | 17.23 | 0.000 | 1.479 | 1.635 |
| Hypothyroidism_b | 1.015 | 0.035 | 0.42 | 0.672 | 0.949 | 1.085 |
| Liver_dis_b | 1.377 | 0.265 | 1.66 | 0.097 | 0.944 | 2.009 |
| Lupus_b | 1.880 | 0.296 | 4.01 | 0.000 | 1.381 | 2.560 |
| Erectile_dysfunction_b | 0.894 | 0.028 | -3.61 | 0.000 | 0.841 | 0.950 |
| Any_tumour_b | 1.442 | 0.036 | 14.52 | 0.000 | 1.373 | 1.515 |
| Menopause_b | 0.760 | 0.025 | -8.19 | 0.000 | 0.711 | 0.811 |
| Dementia_b | 4.091 | 0.387 | 14.91 | 0.000 | 3.399 | 4.923 |
| RA_b | 1.278 | 0.063 | 5.01 | 0.000 | 1.161 | 1.407 |
| _cons | 0.003 | 0.000 | -62.00 | 0.000 | 0.003 | 0.004 |

**Performance in validation dataset (CPRD GOLD) - External validation - Index AMI cases**

| AUROC (95% CI): 0.805 (0.799; 0.812) |  |
| --- | --- |
|  |  |
|  | % Correctly classified: 73.18 |

### 9) 5-year Composite 2 CV outcome (HF, stroke, & CV mortality)

|  | | | | | | |
| --- | --- | --- | --- | --- | --- | --- |
| _5yr_Comp2_CV_outcome | Odds ratio | Std. err. | z | P>|z| | [95% conf. interval] | |
| age | 1.071 | 0.001 | 77.39 | 0.000 | 1.069 | 1.073 |
|  |  |  |  |  |  |  |
| gender |  |  |  |  |  |  |
| Female | 1.045 | 0.023 | 2.01 | 0.045 | 1.001 | 1.091 |
|  |  |  |  |  |  |  |
| IMD |  |  |  |  |  |  |
| Q2 | 1.097 | 0.030 | 3.33 | 0.001 | 1.039 | 1.158 |
| Q3 | 1.214 | 0.034 | 6.96 | 0.000 | 1.150 | 1.282 |
| Q4 | 1.363 | 0.039 | 10.95 | 0.000 | 1.289 | 1.440 |
| Q5-most deprived | 1.583 | 0.046 | 15.97 | 0.000 | 1.496 | 1.675 |
|  |  |  |  |  |  |  |
| Other than White | 0.973 | 0.036 | -0.74 | 0.461 | 0.905 | 1.047 |
| Drinker | 0.830 | 0.020 | -7.70 | 0.000 | 0.792 | 0.871 |
|  |  |  |  |  |  |  |
| Smoking_status |  |  |  |  |  |  |
| current smoker | 1.272 | 0.034 | 8.90 | 0.000 | 1.206 | 1.341 |
| ex-smoker | 0.940 | 0.023 | -2.55 | 0.011 | 0.896 | 0.986 |
|  |  |  |  |  |  |  |
| BMI | 1.003 | 0.002 | 1.61 | 0.108 | 0.999 | 1.006 |
| HT_b | 1.139 | 0.021 | 6.93 | 0.000 | 1.098 | 1.182 |
| Hyperlipidaemia_b | 0.979 | 0.022 | -0.95 | 0.344 | 0.936 | 1.023 |
|  |  |  |  |  |  |  |
| Diabetes_type_b |  |  |  |  |  |  |
| T1DM | 4.364 | 0.438 | 14.67 | 0.000 | 3.584 | 5.313 |
| T2DM | 1.727 | 0.044 | 21.53 | 0.000 | 1.643 | 1.815 |
| DM - nos | 2.225 | 0.178 | 9.99 | 0.000 | 1.902 | 2.603 |
|  |  |  |  |  |  |  |
| HF_b | 7.515 | 0.439 | 34.55 | 0.000 | 6.703 | 8.426 |
| AF_b | 1.936 | 0.067 | 19.07 | 0.000 | 1.809 | 2.072 |
| Heart_valve_dis_b | 1.476 | 0.138 | 4.15 | 0.000 | 1.228 | 1.774 |
| VT_VF_b | 1.795 | 0.279 | 3.76 | 0.000 | 1.323 | 2.436 |
| Cardiomyopathy_b | 2.212 | 0.323 | 5.44 | 0.000 | 1.662 | 2.944 |
| CV_procedures_b | 0.969 | 0.029 | -1.02 | 0.305 | 0.914 | 1.029 |
| TIA_stroke_b | 3.777 | 0.130 | 38.60 | 0.000 | 3.531 | 4.041 |
| PVD_b | 2.010 | 0.079 | 17.86 | 0.000 | 1.862 | 2.170 |
| CKD_b | 1.488 | 0.037 | 16.15 | 0.000 | 1.418 | 1.562 |
| Hypothyroidism_b | 0.991 | 0.033 | -0.28 | 0.779 | 0.927 | 1.058 |
| Liver_dis_b | 1.139 | 0.228 | 0.65 | 0.515 | 0.770 | 1.685 |
| Lupus_b | 2.128 | 0.331 | 4.86 | 0.000 | 1.569 | 2.886 |
| Erectile_dysfunction_b | 0.937 | 0.029 | -2.08 | 0.037 | 0.881 | 0.996 |
| Any_tumour_b | 1.146 | 0.028 | 5.56 | 0.000 | 1.092 | 1.202 |
| Menopause_b | 0.778 | 0.026 | -7.47 | 0.000 | 0.728 | 0.831 |
| Dementia_b | 3.572 | 0.292 | 15.58 | 0.000 | 3.043 | 4.192 |
| RA_b | 1.189 | 0.057 | 3.58 | 0.000 | 1.082 | 1.307 |
| _cons | 0.003 | 0.000 | -61.81 | 0.000 | 0.002 | 0.004 |

**Performance in validation dataset (CPRD GOLD) - External validation - Index AMI cases**

| AUROC (95% CI): 0.795 (0.788; 0.802) |  |
| --- | --- |
|  |  |
|  | % Correctly classified: 72.28 |

## Internal validation (CPRD Aurum) - with therapies

### 1) 5-year all-cause mortality

|  | | | | | | |
| --- | --- | --- | --- | --- | --- | --- |
| _5year_All_cause_death | Odds ratio | Std. err. | z | P>|z| | [95% conf. interval] | |
| age | 1.095 | 0.001 | 80.21 | 0.000 | 1.092 | 1.097 |
|  |  |  |  |  |  |  |
| gender |  |  |  |  |  |  |
| Female | 0.903 | 0.021 | -4.30 | 0.000 | 0.862 | 0.946 |
|  |  |  |  |  |  |  |
| IMD |  |  |  |  |  |  |
| Q2 | 1.095 | 0.034 | 2.94 | 0.003 | 1.031 | 1.163 |
| Q3 | 1.237 | 0.038 | 6.90 | 0.000 | 1.165 | 1.315 |
| Q4 | 1.336 | 0.042 | 9.23 | 0.000 | 1.256 | 1.420 |
| Q5-most deprived | 1.477 | 0.047 | 12.20 | 0.000 | 1.387 | 1.572 |
|  |  |  |  |  |  |  |
| Other than White |  |  |  |  |  |  |
| non-White | 0.733 | 0.033 | -6.93 | 0.000 | 0.671 | 0.800 |
|  |  |  |  |  |  |  |
| Drinker |  |  |  |  |  |  |
| drinker | 0.780 | 0.020 | -9.65 | 0.000 | 0.742 | 0.821 |
|  |  |  |  |  |  |  |
| Smoking_status |  |  |  |  |  |  |
| current smoker | 1.668 | 0.050 | 16.93 | 0.000 | 1.572 | 1.769 |
| ex-smoker | 0.956 | 0.025 | -1.67 | 0.094 | 0.908 | 1.008 |
|  |  |  |  |  |  |  |
| BMI | 0.973 | 0.002 | -13.61 | 0.000 | 0.970 | 0.977 |
| HT_b | 0.823 | 0.019 | -8.56 | 0.000 | 0.787 | 0.861 |
| Antihypertensives_b | 1.104 | 0.037 | 2.97 | 0.003 | 1.034 | 1.178 |
| Hyperlipidaemia_b | 0.878 | 0.023 | -5.01 | 0.000 | 0.835 | 0.924 |
| Lipid_reg_treatment_b | 0.820 | 0.021 | -7.58 | 0.000 | 0.780 | 0.864 |
|  |  |  |  |  |  |  |
| Diabetes_type_b |  |  |  |  |  |  |
| T1DM | 3.611 | 0.423 | 10.95 | 0.000 | 2.870 | 4.544 |
| T2DM | 1.114 | 0.056 | 2.15 | 0.032 | 1.009 | 1.229 |
| DM - nos | 1.730 | 0.154 | 6.17 | 0.000 | 1.454 | 2.059 |
|  |  |  |  |  |  |  |
| Antidiabetics_b | 1.732 | 0.093 | 10.25 | 0.000 | 1.559 | 1.924 |
| Diuretics_b | 1.710 | 0.045 | 20.49 | 0.000 | 1.624 | 1.800 |
| Anticoagulants_b | 1.267 | 0.050 | 5.99 | 0.000 | 1.172 | 1.369 |
| Antiplatelets_b | 1.285 | 0.032 | 10.07 | 0.000 | 1.224 | 1.349 |
| HF_b | 2.065 | 0.081 | 18.51 | 0.000 | 1.912 | 2.230 |
| AF_b | 1.272 | 0.044 | 6.92 | 0.000 | 1.188 | 1.362 |
| Heart_valve_dis_b | 1.413 | 0.119 | 4.12 | 0.000 | 1.199 | 1.666 |
| VT_VF_b | 1.164 | 0.174 | 1.02 | 0.309 | 0.869 | 1.559 |
| Cardiomyopathy_b | 1.012 | 0.131 | 0.10 | 0.924 | 0.786 | 1.305 |
| CV_procedures_b | 0.818 | 0.027 | -6.01 | 0.000 | 0.766 | 0.873 |
| TIA_stroke_b | 1.484 | 0.045 | 12.95 | 0.000 | 1.398 | 1.575 |
| PVD_b | 1.807 | 0.067 | 15.93 | 0.000 | 1.680 | 1.944 |
| CKD_b | 1.397 | 0.034 | 13.78 | 0.000 | 1.332 | 1.465 |
| Hypothyroidism_b | 0.968 | 0.033 | -0.93 | 0.353 | 0.905 | 1.036 |
| Liver_dis_b | 1.581 | 0.361 | 2.00 | 0.045 | 1.010 | 2.474 |
| Lupus_b | 1.803 | 0.295 | 3.61 | 0.000 | 1.309 | 2.483 |
| Erectile_dysfunction_b | 0.784 | 0.029 | -6.61 | 0.000 | 0.729 | 0.842 |
| Any_tumour_b | 1.582 | 0.039 | 18.43 | 0.000 | 1.506 | 1.661 |
| Menopause_b | 0.728 | 0.027 | -8.51 | 0.000 | 0.676 | 0.783 |
| Dementia_b | 4.194 | 0.298 | 20.18 | 0.000 | 3.649 | 4.820 |
| RA_b | 1.426 | 0.072 | 7.01 | 0.000 | 1.291 | 1.575 |
| _cons | 0.001 | 0.000 | -65.79 | 0.000 | 0.000 | 0.001 |

**Performance in validation dataset (20% of practices) - Internal validation - Index AMI cases**

| AUROC (95% CI): 0.847 (0.841; 0.853) |  |
| --- | --- |
|  |  |
|  | % Correctly classified: 77.96 |

### 2) 5-year Stroke

|  | | | | | | |
| --- | --- | --- | --- | --- | --- | --- |
| _5year_Stroke | Odds ratio | Std. err. | z | P>|z| | [95% conf. interval] | |
| age | 1.064 | 0.001 | 53.22 | 0.000 | 1.062 | 1.066 |
|  |  |  |  |  |  |  |
| gender |  |  |  |  |  |  |
| Female | 1.077 | 0.027 | 2.95 | 0.003 | 1.025 | 1.131 |
|  |  |  |  |  |  |  |
| IMD |  |  |  |  |  |  |
| Q2 | 1.005 | 0.033 | 0.14 | 0.889 | 0.942 | 1.072 |
| Q3 | 1.112 | 0.037 | 3.23 | 0.001 | 1.043 | 1.187 |
| Q4 | 1.213 | 0.040 | 5.81 | 0.000 | 1.137 | 1.295 |
| Q5-most deprived | 1.276 | 0.043 | 7.17 | 0.000 | 1.193 | 1.363 |
|  |  |  |  |  |  |  |
| Other than White |  |  |  |  |  |  |
| non-White | 0.921 | 0.043 | -1.76 | 0.079 | 0.841 | 1.009 |
|  |  |  |  |  |  |  |
| Drinker |  |  |  |  |  |  |
| drinker | 0.922 | 0.025 | -3.01 | 0.003 | 0.875 | 0.972 |
|  |  |  |  |  |  |  |
| Smoking_status |  |  |  |  |  |  |
| current smoker | 1.260 | 0.040 | 7.27 | 0.000 | 1.184 | 1.341 |
| ex-smoker | 0.884 | 0.025 | -4.42 | 0.000 | 0.837 | 0.934 |
|  |  |  |  |  |  |  |
| BMI | 0.981 | 0.002 | -9.00 | 0.000 | 0.977 | 0.985 |
| HT_b | 0.995 | 0.023 | -0.21 | 0.837 | 0.950 | 1.042 |
| Antihypertensives_b | 1.341 | 0.051 | 7.72 | 0.000 | 1.245 | 1.444 |
| Hyperlipidaemia_b | 1.040 | 0.028 | 1.46 | 0.145 | 0.987 | 1.097 |
| Lipid_reg_treatment_b | 0.843 | 0.023 | -6.20 | 0.000 | 0.799 | 0.890 |
|  |  |  |  |  |  |  |
| Diabetes_type_b |  |  |  |  |  |  |
| T1DM | 1.742 | 0.208 | 4.65 | 0.000 | 1.378 | 2.201 |
| T2DM | 0.919 | 0.049 | -1.59 | 0.112 | 0.828 | 1.020 |
| DM - nos | 0.991 | 0.091 | -0.10 | 0.922 | 0.827 | 1.188 |
|  |  |  |  |  |  |  |
| Antidiabetics_b | 1.922 | 0.108 | 11.60 | 0.000 | 1.721 | 2.146 |
| Diuretics_b | 1.376 | 0.038 | 11.45 | 0.000 | 1.303 | 1.454 |
| Anticoagulants_b | 1.024 | 0.041 | 0.59 | 0.557 | 0.946 | 1.108 |
| Antiplatelets_b | 1.371 | 0.036 | 11.94 | 0.000 | 1.302 | 1.444 |
| HF_b | 1.402 | 0.052 | 9.09 | 0.000 | 1.304 | 1.508 |
| AF_b | 1.587 | 0.055 | 13.38 | 0.000 | 1.483 | 1.698 |
| Heart_valve_dis_b | 1.311 | 0.107 | 3.31 | 0.001 | 1.117 | 1.539 |
| VT_VF_b | 1.077 | 0.163 | 0.49 | 0.626 | 0.800 | 1.449 |
| Cardiomyopathy_b | 1.187 | 0.155 | 1.32 | 0.187 | 0.920 | 1.533 |
| CV_procedures_b | 0.859 | 0.030 | -4.32 | 0.000 | 0.801 | 0.920 |
| TIA_stroke_b | 6.851 | 0.212 | 62.10 | 0.000 | 6.448 | 7.280 |
| PVD_b | 1.716 | 0.063 | 14.61 | 0.000 | 1.596 | 1.844 |
| CKD_b | 1.294 | 0.032 | 10.36 | 0.000 | 1.232 | 1.358 |
| Hypothyroidism_b | 1.087 | 0.038 | 2.36 | 0.018 | 1.014 | 1.165 |
| Liver_dis_b | 1.644 | 0.393 | 2.08 | 0.038 | 1.029 | 2.625 |
| Lupus_b | 1.678 | 0.285 | 3.04 | 0.002 | 1.202 | 2.342 |
| Erectile_dysfunction_b | 1.022 | 0.040 | 0.56 | 0.572 | 0.947 | 1.103 |
| Any_tumour_b | 1.067 | 0.028 | 2.45 | 0.014 | 1.013 | 1.124 |
| Menopause_b | 0.841 | 0.033 | -4.39 | 0.000 | 0.778 | 0.908 |
| Dementia_b | 3.260 | 0.194 | 19.86 | 0.000 | 2.901 | 3.663 |
| RA_b | 0.894 | 0.050 | -2.00 | 0.045 | 0.801 | 0.998 |
| _cons | 0.002 | 0.000 | -53.44 | 0.000 | 0.001 | 0.002 |

**Performance in validation dataset (20% of practices) - Internal validation - Index AMI cases**

| AUROC (95% CI): 0.833 (0.826; 0.841) |  |
| --- | --- |
|  |  |
|  | % Correctly classified: 78.91 |

### 3) 5-year heart failure (HF)

|  | | | | | | |
| --- | --- | --- | --- | --- | --- | --- |
| _5year_HF | Odds ratio | Std. err. | z | P>|z| | [95% conf. interval] | |
| age | 1.061 | 0.001 | 61.78 | 0.000 | 1.059 | 1.063 |
|  |  |  |  |  |  |  |
| gender |  |  |  |  |  |  |
| Female | 0.901 | 0.020 | -4.69 | 0.000 | 0.863 | 0.941 |
|  |  |  |  |  |  |  |
| IMD |  |  |  |  |  |  |
| Q2 | 1.067 | 0.030 | 2.29 | 0.022 | 1.009 | 1.129 |
| Q3 | 1.114 | 0.032 | 3.78 | 0.000 | 1.054 | 1.179 |
| Q4 | 1.261 | 0.036 | 8.03 | 0.000 | 1.192 | 1.335 |
| Q5-most deprived | 1.457 | 0.043 | 12.82 | 0.000 | 1.375 | 1.543 |
|  |  |  |  |  |  |  |
| Other than White |  |  |  |  |  |  |
| non-White | 1.055 | 0.041 | 1.40 | 0.163 | 0.979 | 1.138 |
|  |  |  |  |  |  |  |
| Drinker |  |  |  |  |  |  |
| drinker | 0.820 | 0.020 | -8.27 | 0.000 | 0.782 | 0.860 |
|  |  |  |  |  |  |  |
| Smoking_status |  |  |  |  |  |  |
| current smoker | 1.284 | 0.035 | 9.03 | 0.000 | 1.216 | 1.355 |
| ex-smoker | 0.973 | 0.024 | -1.10 | 0.273 | 0.927 | 1.021 |
|  |  |  |  |  |  |  |
| BMI | 1.003 | 0.002 | 1.48 | 0.138 | 0.999 | 1.006 |
| HT_b | 0.905 | 0.019 | -4.70 | 0.000 | 0.868 | 0.943 |
| Antihypertensives_b | 0.995 | 0.029 | -0.17 | 0.866 | 0.939 | 1.054 |
| Hyperlipidaemia_b | 0.979 | 0.024 | -0.86 | 0.387 | 0.934 | 1.027 |
| Lipid_reg_treatment_b | 0.885 | 0.021 | -5.07 | 0.000 | 0.844 | 0.928 |
|  |  |  |  |  |  |  |
| Diabetes_type_b |  |  |  |  |  |  |
| T1DM | 2.279 | 0.248 | 7.57 | 0.000 | 1.841 | 2.820 |
| T2DM | 1.129 | 0.053 | 2.59 | 0.010 | 1.030 | 1.238 |
| DM - nos | 1.397 | 0.118 | 3.95 | 0.000 | 1.183 | 1.649 |
|  |  |  |  |  |  |  |
| Antidiabetics_b | 1.681 | 0.084 | 10.36 | 0.000 | 1.524 | 1.854 |
| Diuretics_b | 1.708 | 0.041 | 22.31 | 0.000 | 1.630 | 1.790 |
| Anticoagulants_b | 1.250 | 0.048 | 5.77 | 0.000 | 1.158 | 1.348 |
| Antiplatelets_b | 1.111 | 0.026 | 4.56 | 0.000 | 1.062 | 1.162 |
| HF_b | 7.684 | 0.384 | 40.78 | 0.000 | 6.967 | 8.475 |
| AF_b | 1.639 | 0.056 | 14.45 | 0.000 | 1.533 | 1.753 |
| Heart_valve_dis_b | 1.805 | 0.156 | 6.83 | 0.000 | 1.523 | 2.138 |
| VT_VF_b | 1.424 | 0.207 | 2.44 | 0.015 | 1.072 | 1.893 |
| Cardiomyopathy_b | 2.245 | 0.307 | 5.91 | 0.000 | 1.717 | 2.936 |
| CV_procedures_b | 0.935 | 0.029 | -2.16 | 0.031 | 0.880 | 0.994 |
| TIA_stroke_b | 1.296 | 0.038 | 8.80 | 0.000 | 1.223 | 1.373 |
| PVD_b | 1.721 | 0.062 | 15.06 | 0.000 | 1.603 | 1.846 |
| CKD_b | 1.424 | 0.033 | 15.11 | 0.000 | 1.360 | 1.491 |
| Hypothyroidism_b | 1.038 | 0.034 | 1.15 | 0.250 | 0.974 | 1.108 |
| Liver_dis_b | 1.195 | 0.249 | 0.86 | 0.392 | 0.795 | 1.797 |
| Lupus_b | 1.804 | 0.273 | 3.90 | 0.000 | 1.341 | 2.426 |
| Erectile_dysfunction_b | 0.932 | 0.031 | -2.14 | 0.032 | 0.874 | 0.994 |
| Any_tumour_b | 1.160 | 0.028 | 6.17 | 0.000 | 1.107 | 1.216 |
| Menopause_b | 0.767 | 0.027 | -7.63 | 0.000 | 0.716 | 0.821 |
| Dementia_b | 2.185 | 0.131 | 13.00 | 0.000 | 1.942 | 2.458 |
| RA_b | 1.254 | 0.060 | 4.72 | 0.000 | 1.142 | 1.378 |
| _cons | 0.004 | 0.000 | -56.99 | 0.000 | 0.003 | 0.004 |

**Performance in validation dataset (20% of practices) - Internal validation - Index AMI cases**

| AUROC (95% CI): 0.800 (0.793; 0.806) |  |
| --- | --- |
|  |  |
|  | % Correctly classified: 74.14 |

### 4) 5-year Recurrent MI

|  | | | | | | |
| --- | --- | --- | --- | --- | --- | --- |
| _5year_Recurrent_MI | Odds ratio | Std. err. | z | P>|z| | [95% conf. interval] | |
| age | 1.030 | 0.001 | 31.81 | 0.000 | 1.028 | 1.032 |
|  |  |  |  |  |  |  |
| gender |  |  |  |  |  |  |
| Female | 0.981 | 0.022 | -0.85 | 0.396 | 0.940 | 1.025 |
|  |  |  |  |  |  |  |
| IMD |  |  |  |  |  |  |
| Q2 | 1.088 | 0.031 | 2.96 | 0.003 | 1.029 | 1.150 |
| Q3 | 1.041 | 0.030 | 1.41 | 0.159 | 0.984 | 1.102 |
| Q4 | 1.056 | 0.031 | 1.88 | 0.061 | 0.998 | 1.118 |
| Q5-most deprived | 1.139 | 0.034 | 4.40 | 0.000 | 1.075 | 1.206 |
|  |  |  |  |  |  |  |
| Other than White |  |  |  |  |  |  |
| non-White | 1.086 | 0.042 | 2.15 | 0.031 | 1.007 | 1.171 |
|  |  |  |  |  |  |  |
| Drinker |  |  |  |  |  |  |
| drinker | 0.900 | 0.021 | -4.42 | 0.000 | 0.859 | 0.943 |
|  |  |  |  |  |  |  |
| Smoking_status |  |  |  |  |  |  |
| current smoker | 1.163 | 0.032 | 5.50 | 0.000 | 1.102 | 1.228 |
| ex-smoker | 0.927 | 0.023 | -3.08 | 0.002 | 0.883 | 0.973 |
|  |  |  |  |  |  |  |
| BMI | 0.990 | 0.002 | -5.62 | 0.000 | 0.986 | 0.993 |
| HT_b | 0.957 | 0.020 | -2.07 | 0.038 | 0.918 | 0.998 |
| Antihypertensives_b | 0.957 | 0.029 | -1.45 | 0.148 | 0.902 | 1.016 |
| Hyperlipidaemia_b | 1.028 | 0.025 | 1.15 | 0.249 | 0.981 | 1.078 |
| Lipid_reg_treatment_b | 0.873 | 0.021 | -5.61 | 0.000 | 0.832 | 0.915 |
|  |  |  |  |  |  |  |
| Diabetes_type_b |  |  |  |  |  |  |
| T1DM | 2.039 | 0.210 | 6.92 | 0.000 | 1.667 | 2.495 |
| T2DM | 0.979 | 0.047 | -0.44 | 0.661 | 0.892 | 1.075 |
| DM - nos | 1.268 | 0.103 | 2.93 | 0.003 | 1.082 | 1.486 |
|  |  |  |  |  |  |  |
| Antidiabetics_b | 1.454 | 0.073 | 7.43 | 0.000 | 1.317 | 1.605 |
| Diuretics_b | 1.347 | 0.034 | 11.83 | 0.000 | 1.282 | 1.416 |
| Anticoagulants_b | 1.041 | 0.038 | 1.08 | 0.278 | 0.968 | 1.119 |
| Antiplatelets_b | 1.203 | 0.028 | 7.83 | 0.000 | 1.149 | 1.261 |
| HF_b | 1.462 | 0.050 | 11.18 | 0.000 | 1.368 | 1.562 |
| AF_b | 1.121 | 0.036 | 3.54 | 0.000 | 1.052 | 1.194 |
| Heart_valve_dis_b | 1.331 | 0.099 | 3.85 | 0.000 | 1.151 | 1.539 |
| VT_VF_b | 1.006 | 0.138 | 0.04 | 0.966 | 0.768 | 1.317 |
| Cardiomyopathy_b | 0.817 | 0.102 | -1.62 | 0.106 | 0.639 | 1.044 |
| CV_procedures_b | 0.895 | 0.028 | -3.52 | 0.000 | 0.842 | 0.952 |
| TIA_stroke_b | 1.250 | 0.035 | 7.91 | 0.000 | 1.183 | 1.321 |
| PVD_b | 1.479 | 0.050 | 11.56 | 0.000 | 1.384 | 1.580 |
| CKD_b | 1.268 | 0.029 | 10.31 | 0.000 | 1.212 | 1.326 |
| Hypothyroidism_b | 1.035 | 0.033 | 1.07 | 0.286 | 0.972 | 1.103 |
| Liver_dis_b | 2.099 | 0.385 | 4.04 | 0.000 | 1.465 | 3.007 |
| Lupus_b | 1.520 | 0.225 | 2.83 | 0.005 | 1.137 | 2.031 |
| Erectile_dysfunction_b | 0.858 | 0.029 | -4.51 | 0.000 | 0.802 | 0.917 |
| Any_tumour_b | 1.161 | 0.028 | 6.25 | 0.000 | 1.108 | 1.217 |
| Menopause_b | 0.757 | 0.027 | -7.77 | 0.000 | 0.705 | 0.812 |
| Dementia_b | 2.278 | 0.118 | 15.83 | 0.000 | 2.057 | 2.522 |
| RA_b | 0.908 | 0.046 | -1.91 | 0.057 | 0.823 | 1.003 |
| _cons | 0.033 | 0.003 | -35.24 | 0.000 | 0.027 | 0.040 |

**Performance in validation dataset (20% of practices) - Internal validation - Index AMI cases**

| AUROC (95% CI): 0.675 (0.666; 0.685) |  |
| --- | --- |
|  |  |
|  | % Correctly classified: 67.07 |

### 5) 5-year CV mortality

|  | | | | | | |
| --- | --- | --- | --- | --- | --- | --- |
| _5year_CV_death | Odds ratio | Std. err. | z | P>|z| | [95% conf. interval] | |
| age | 1.100 | 0.002 | 68.37 | 0.000 | 1.097 | 1.103 |
|  |  |  |  |  |  |  |
| gender |  |  |  |  |  |  |
| Female | 0.776 | 0.021 | -9.51 | 0.000 | 0.736 | 0.817 |
|  |  |  |  |  |  |  |
| IMD |  |  |  |  |  |  |
| Q2 | 1.085 | 0.039 | 2.30 | 0.021 | 1.012 | 1.163 |
| Q3 | 1.139 | 0.041 | 3.65 | 0.000 | 1.062 | 1.222 |
| Q4 | 1.293 | 0.047 | 7.13 | 0.000 | 1.205 | 1.387 |
| Q5-most deprived | 1.368 | 0.050 | 8.51 | 0.000 | 1.273 | 1.471 |
|  |  |  |  |  |  |  |
| Other than White |  |  |  |  |  |  |
| non-White | 0.776 | 0.042 | -4.73 | 0.000 | 0.698 | 0.862 |
|  |  |  |  |  |  |  |
| Drinker |  |  |  |  |  |  |
| drinker | 0.797 | 0.023 | -7.98 | 0.000 | 0.754 | 0.843 |
|  |  |  |  |  |  |  |
| Smoking_status |  |  |  |  |  |  |
| current smoker | 1.329 | 0.045 | 8.31 | 0.000 | 1.242 | 1.421 |
| ex-smoker | 0.810 | 0.024 | -7.10 | 0.000 | 0.764 | 0.858 |
|  |  |  |  |  |  |  |
| BMI | 0.983 | 0.002 | -7.58 | 0.000 | 0.978 | 0.987 |
| HT_b | 0.860 | 0.022 | -5.96 | 0.000 | 0.819 | 0.904 |
| Antihypertensives_b | 1.105 | 0.048 | 2.28 | 0.023 | 1.014 | 1.204 |
| Hyperlipidaemia_b | 0.985 | 0.029 | -0.53 | 0.598 | 0.930 | 1.043 |
| Lipid_reg_treatment_b | 0.784 | 0.023 | -8.19 | 0.000 | 0.740 | 0.831 |
|  |  |  |  |  |  |  |
| Diabetes_type_b |  |  |  |  |  |  |
| T1DM | 5.453 | 0.680 | 13.60 | 0.000 | 4.271 | 6.963 |
| T2DM | 1.259 | 0.069 | 4.21 | 0.000 | 1.131 | 1.402 |
| DM - nos | 1.880 | 0.177 | 6.69 | 0.000 | 1.562 | 2.262 |
|  |  |  |  |  |  |  |
| Antidiabetics_b | 1.586 | 0.092 | 7.95 | 0.000 | 1.415 | 1.777 |
| Diuretics_b | 1.847 | 0.058 | 19.45 | 0.000 | 1.736 | 1.965 |
| Anticoagulants_b | 1.201 | 0.049 | 4.44 | 0.000 | 1.108 | 1.301 |
| Antiplatelets_b | 1.373 | 0.040 | 11.00 | 0.000 | 1.297 | 1.452 |
| HF_b | 2.475 | 0.092 | 24.29 | 0.000 | 2.301 | 2.663 |
| AF_b | 1.364 | 0.048 | 8.77 | 0.000 | 1.272 | 1.461 |
| Heart_valve_dis_b | 1.612 | 0.134 | 5.76 | 0.000 | 1.370 | 1.896 |
| VT_VF_b | 1.418 | 0.218 | 2.27 | 0.023 | 1.049 | 1.918 |
| Cardiomyopathy_b | 1.239 | 0.170 | 1.56 | 0.120 | 0.946 | 1.622 |
| CV_procedures_b | 0.866 | 0.033 | -3.78 | 0.000 | 0.803 | 0.933 |
| TIA_stroke_b | 1.537 | 0.048 | 13.74 | 0.000 | 1.446 | 1.634 |
| PVD_b | 1.960 | 0.075 | 17.70 | 0.000 | 1.819 | 2.111 |
| CKD_b | 1.351 | 0.035 | 11.70 | 0.000 | 1.285 | 1.421 |
| Hypothyroidism_b | 0.937 | 0.035 | -1.72 | 0.086 | 0.870 | 1.009 |
| Liver_dis_b | 1.390 | 0.405 | 1.13 | 0.258 | 0.786 | 2.459 |
| Lupus_b | 2.234 | 0.402 | 4.46 | 0.000 | 1.570 | 3.179 |
| Erectile_dysfunction_b | 0.741 | 0.034 | -6.59 | 0.000 | 0.678 | 0.810 |
| Any_tumour_b | 0.968 | 0.027 | -1.16 | 0.244 | 0.917 | 1.022 |
| Menopause_b | 0.707 | 0.032 | -7.65 | 0.000 | 0.647 | 0.772 |
| Dementia_b | 3.157 | 0.183 | 19.81 | 0.000 | 2.817 | 3.537 |
| RA_b | 1.489 | 0.084 | 7.09 | 0.000 | 1.334 | 1.662 |
| _cons | 0.000 | 0.000 | -63.30 | 0.000 | 0.000 | 0.000 |

**Performance in validation dataset (20% of practices) - Internal validation - Index AMI cases**

| AUROC (95% CI): 0.854 (0.847; 0.861) |  |
| --- | --- |
|  |  |
|  | % Correctly classified: 80.66 |

### 6) 5-year Composite 1 outcome (HF, stroke, recurrent MI, & all-cause mortality)

|  | | | | | | |
| --- | --- | --- | --- | --- | --- | --- |
| _5yr_Comp1_outcome | Odds ratio | Std. err. | z | P>|z| | [95% conf. interval] | |
| age | 1.055 | 0.001 | 62.92 | 0.000 | 1.054 | 1.057 |
|  |  |  |  |  |  |  |
| gender |  |  |  |  |  |  |
| Female | 1.005 | 0.022 | 0.21 | 0.834 | 0.963 | 1.048 |
|  |  |  |  |  |  |  |
| IMD |  |  |  |  |  |  |
| Q2 | 1.098 | 0.029 | 3.51 | 0.000 | 1.042 | 1.157 |
| Q3 | 1.204 | 0.032 | 6.90 | 0.000 | 1.142 | 1.269 |
| Q4 | 1.274 | 0.035 | 8.90 | 0.000 | 1.208 | 1.344 |
| Q5-most deprived | 1.492 | 0.041 | 14.42 | 0.000 | 1.413 | 1.576 |
|  |  |  |  |  |  |  |
| Other than White |  |  |  |  |  |  |
| non-White | 0.962 | 0.034 | -1.09 | 0.276 | 0.898 | 1.031 |
|  |  |  |  |  |  |  |
| Drinker |  |  |  |  |  |  |
| drinker | 0.887 | 0.021 | -4.97 | 0.000 | 0.847 | 0.930 |
|  |  |  |  |  |  |  |
| Smoking_status |  |  |  |  |  |  |
| current smoker | 1.304 | 0.034 | 10.16 | 0.000 | 1.239 | 1.373 |
| ex-smoker | 0.945 | 0.023 | -2.38 | 0.017 | 0.902 | 0.990 |
|  |  |  |  |  |  |  |
| BMI | 0.992 | 0.002 | -4.84 | 0.000 | 0.989 | 0.995 |
| HT_b | 0.893 | 0.019 | -5.34 | 0.000 | 0.856 | 0.931 |
| Antihypertensives_b | 1.033 | 0.026 | 1.30 | 0.195 | 0.983 | 1.086 |
| Hyperlipidaemia_b | 0.972 | 0.023 | -1.18 | 0.237 | 0.928 | 1.019 |
| Lipid_reg_treatment_b | 0.839 | 0.019 | -7.58 | 0.000 | 0.801 | 0.878 |
|  |  |  |  |  |  |  |
| Diabetes_type_b |  |  |  |  |  |  |
| T1DM | 2.027 | 0.229 | 6.27 | 0.000 | 1.625 | 2.528 |
| T2DM | 1.100 | 0.052 | 1.99 | 0.046 | 1.002 | 1.208 |
| DM - nos | 1.506 | 0.137 | 4.50 | 0.000 | 1.260 | 1.800 |
|  |  |  |  |  |  |  |
| Antidiabetics_b | 1.657 | 0.085 | 9.82 | 0.000 | 1.498 | 1.833 |
| Diuretics_b | 1.575 | 0.036 | 19.83 | 0.000 | 1.506 | 1.648 |
| Anticoagulants_b | 1.274 | 0.055 | 5.61 | 0.000 | 1.171 | 1.386 |
| Antiplatelets_b | 1.183 | 0.026 | 7.52 | 0.000 | 1.132 | 1.236 |
| HF_b | 5.155 | 0.327 | 25.83 | 0.000 | 4.552 | 5.838 |
| AF_b | 1.566 | 0.063 | 11.19 | 0.000 | 1.447 | 1.694 |
| Heart_valve_dis_b | 1.266 | 0.124 | 2.42 | 0.016 | 1.046 | 1.534 |
| VT_VF_b | 1.323 | 0.207 | 1.79 | 0.073 | 0.974 | 1.797 |
| Cardiomyopathy_b | 2.011 | 0.313 | 4.49 | 0.000 | 1.482 | 2.727 |
| CV_procedures_b | 0.849 | 0.026 | -5.42 | 0.000 | 0.800 | 0.901 |
| TIA_stroke_b | 2.870 | 0.108 | 27.99 | 0.000 | 2.666 | 3.090 |
| PVD_b | 1.829 | 0.077 | 14.41 | 0.000 | 1.685 | 1.986 |
| CKD_b | 1.450 | 0.038 | 14.17 | 0.000 | 1.377 | 1.526 |
| Hypothyroidism_b | 0.998 | 0.034 | -0.07 | 0.947 | 0.933 | 1.067 |
| Liver_dis_b | 2.004 | 0.366 | 3.81 | 0.000 | 1.401 | 2.866 |
| Lupus_b | 1.531 | 0.234 | 2.79 | 0.005 | 1.135 | 2.065 |
| Erectile_dysfunction_b | 0.908 | 0.027 | -3.23 | 0.001 | 0.856 | 0.963 |
| Any_tumour_b | 1.348 | 0.034 | 11.82 | 0.000 | 1.283 | 1.417 |
| Menopause_b | 0.749 | 0.024 | -8.84 | 0.000 | 0.703 | 0.799 |
| Dementia_b | 4.024 | 0.401 | 13.97 | 0.000 | 3.310 | 4.892 |
| RA_b | 1.173 | 0.057 | 3.30 | 0.001 | 1.067 | 1.290 |
| _cons | 0.018 | 0.002 | -45.34 | 0.000 | 0.015 | 0.022 |

**Performance in validation dataset (20% of practices) - Internal validation - Index AMI cases**

| AUROC (95% CI): 0.785 (0.779; 0.792) |  |
| --- | --- |
|  |  |
|  | % Correctly classified: 71.63 |

### 7) 5-year Composite 1 CV outcome (HF, stroke, recurrent MI, & CV mortality)

|  | | | | | | |
| --- | --- | --- | --- | --- | --- | --- |
| _5yr_Comp1_CV_outcome | Odds ratio | Std. err. | z | P>|z| | [95% conf. interval] | |
| age | 1.051 | 0.001 | 58.72 | 0.000 | 1.049 | 1.053 |
|  |  |  |  |  |  |  |
| gender |  |  |  |  |  |  |
| Female | 1.014 | 0.022 | 0.65 | 0.514 | 0.972 | 1.057 |
|  |  |  |  |  |  |  |
| IMD |  |  |  |  |  |  |
| Q2 | 1.085 | 0.029 | 3.07 | 0.002 | 1.030 | 1.142 |
| Q3 | 1.196 | 0.032 | 6.72 | 0.000 | 1.135 | 1.260 |
| Q4 | 1.276 | 0.034 | 9.02 | 0.000 | 1.210 | 1.345 |
| Q5-most deprived | 1.486 | 0.041 | 14.42 | 0.000 | 1.408 | 1.568 |
|  |  |  |  |  |  |  |
| Other than White |  |  |  |  |  |  |
| non-White | 1.000 | 0.035 | 0.00 | 0.997 | 0.934 | 1.071 |
|  |  |  |  |  |  |  |
| Drinker |  |  |  |  |  |  |
| drinker | 0.899 | 0.021 | -4.50 | 0.000 | 0.858 | 0.942 |
|  |  |  |  |  |  |  |
| Smoking_status |  |  |  |  |  |  |
| current smoker | 1.217 | 0.031 | 7.60 | 0.000 | 1.157 | 1.280 |
| ex-smoker | 0.918 | 0.022 | -3.62 | 0.000 | 0.877 | 0.962 |
|  |  |  |  |  |  |  |
| BMI | 0.998 | 0.002 | -1.24 | 0.214 | 0.995 | 1.001 |
| HT_b | 0.933 | 0.019 | -3.31 | 0.001 | 0.896 | 0.972 |
| Antihypertensives_b | 1.016 | 0.026 | 0.63 | 0.527 | 0.967 | 1.068 |
| Hyperlipidaemia_b | 0.980 | 0.023 | -0.87 | 0.383 | 0.936 | 1.026 |
| Lipid_reg_treatment_b | 0.839 | 0.019 | -7.65 | 0.000 | 0.802 | 0.878 |
|  |  |  |  |  |  |  |
| Diabetes_type_b |  |  |  |  |  |  |
| T1DM | 1.982 | 0.218 | 6.22 | 0.000 | 1.597 | 2.459 |
| T2DM | 1.153 | 0.054 | 3.04 | 0.002 | 1.052 | 1.264 |
| DM - nos | 1.606 | 0.143 | 5.32 | 0.000 | 1.349 | 1.912 |
|  |  |  |  |  |  |  |
| Antidiabetics_b | 1.553 | 0.078 | 8.73 | 0.000 | 1.407 | 1.714 |
| Diuretics_b | 1.545 | 0.035 | 19.25 | 0.000 | 1.478 | 1.615 |
| Anticoagulants_b | 1.285 | 0.054 | 6.02 | 0.000 | 1.184 | 1.395 |
| Antiplatelets_b | 1.214 | 0.027 | 8.83 | 0.000 | 1.163 | 1.267 |
| HF_b | 5.283 | 0.316 | 27.86 | 0.000 | 4.700 | 5.940 |
| AF_b | 1.556 | 0.060 | 11.55 | 0.000 | 1.444 | 1.677 |
| Heart_valve_dis_b | 1.343 | 0.127 | 3.12 | 0.002 | 1.116 | 1.617 |
| VT_VF_b | 1.555 | 0.240 | 2.86 | 0.004 | 1.149 | 2.105 |
| Cardiomyopathy_b | 1.831 | 0.271 | 4.09 | 0.000 | 1.370 | 2.448 |
| CV_procedures_b | 0.843 | 0.025 | -5.69 | 0.000 | 0.795 | 0.894 |
| TIA_stroke_b | 2.954 | 0.106 | 30.09 | 0.000 | 2.753 | 3.170 |
| PVD_b | 1.892 | 0.076 | 15.77 | 0.000 | 1.748 | 2.048 |
| CKD_b | 1.455 | 0.037 | 14.85 | 0.000 | 1.384 | 1.528 |
| Hypothyroidism_b | 0.990 | 0.033 | -0.30 | 0.764 | 0.928 | 1.057 |
| Liver_dis_b | 1.656 | 0.300 | 2.79 | 0.005 | 1.162 | 2.361 |
| Lupus_b | 1.375 | 0.205 | 2.14 | 0.033 | 1.027 | 1.843 |
| Erectile_dysfunction_b | 0.937 | 0.028 | -2.18 | 0.029 | 0.884 | 0.993 |
| Any_tumour_b | 1.160 | 0.028 | 6.06 | 0.000 | 1.105 | 1.216 |
| Menopause_b | 0.746 | 0.024 | -9.08 | 0.000 | 0.700 | 0.794 |
| Dementia_b | 3.372 | 0.290 | 14.15 | 0.000 | 2.850 | 3.991 |
| RA_b | 1.163 | 0.055 | 3.19 | 0.001 | 1.060 | 1.277 |
| _cons | 0.018 | 0.002 | -45.81 | 0.000 | 0.015 | 0.021 |

**Performance in validation dataset (20% of practices) - Internal validation - Index AMI cases**

| AUROC (95% CI): 0.773 (0.766; 0.779) |  |
| --- | --- |
|  |  |
|  | % Correctly classified: 70.76 |

### 8) 5-year Composite 2 outcome (HF, stroke, & all-cause mortality)

|  | | | | | | |
| --- | --- | --- | --- | --- | --- | --- |
| _5yr_Comp2_outcome | Odds ratio | Std. err. | z | P>|z| | [95% conf. interval] | |
| age | 1.069 | 0.001 | 72.69 | 0.000 | 1.067 | 1.071 |
|  |  |  |  |  |  |  |
| gender |  |  |  |  |  |  |
| Female | 0.981 | 0.022 | -0.88 | 0.381 | 0.938 | 1.025 |
|  |  |  |  |  |  |  |
| IMD |  |  |  |  |  |  |
| Q2 | 1.100 | 0.031 | 3.41 | 0.001 | 1.041 | 1.162 |
| Q3 | 1.235 | 0.035 | 7.50 | 0.000 | 1.169 | 1.305 |
| Q4 | 1.372 | 0.039 | 11.10 | 0.000 | 1.298 | 1.451 |
| Q5-most deprived | 1.622 | 0.047 | 16.64 | 0.000 | 1.532 | 1.717 |
|  |  |  |  |  |  |  |
| Other than White |  |  |  |  |  |  |
| non-White | 0.875 | 0.033 | -3.55 | 0.000 | 0.813 | 0.942 |
|  |  |  |  |  |  |  |
| Drinker |  |  |  |  |  |  |
| drinker | 0.846 | 0.021 | -6.79 | 0.000 | 0.806 | 0.888 |
|  |  |  |  |  |  |  |
| Smoking_status |  |  |  |  |  |  |
| current smoker | 1.374 | 0.038 | 11.62 | 0.000 | 1.302 | 1.450 |
| ex-smoker | 0.944 | 0.023 | -2.33 | 0.020 | 0.899 | 0.991 |
|  |  |  |  |  |  |  |
| BMI | 0.991 | 0.002 | -4.88 | 0.000 | 0.988 | 0.995 |
| HT_b | 0.864 | 0.019 | -6.71 | 0.000 | 0.828 | 0.902 |
| Antihypertensives_b | 1.098 | 0.030 | 3.48 | 0.001 | 1.042 | 1.158 |
| Hyperlipidaemia_b | 0.965 | 0.024 | -1.46 | 0.144 | 0.920 | 1.012 |
| Lipid_reg_treatment_b | 0.850 | 0.020 | -6.75 | 0.000 | 0.811 | 0.891 |
|  |  |  |  |  |  |  |
| Diabetes_type_b |  |  |  |  |  |  |
| T1DM | 2.400 | 0.274 | 7.66 | 0.000 | 1.918 | 3.002 |
| T2DM | 1.102 | 0.054 | 1.99 | 0.047 | 1.001 | 1.212 |
| DM - nos | 1.556 | 0.142 | 4.84 | 0.000 | 1.301 | 1.862 |
|  |  |  |  |  |  |  |
| Antidiabetics_b | 1.784 | 0.093 | 11.05 | 0.000 | 1.610 | 1.976 |
| Diuretics_b | 1.640 | 0.039 | 21.01 | 0.000 | 1.566 | 1.717 |
| Anticoagulants_b | 1.329 | 0.057 | 6.59 | 0.000 | 1.221 | 1.446 |
| Antiplatelets_b | 1.231 | 0.028 | 9.07 | 0.000 | 1.177 | 1.287 |
| HF_b | 5.937 | 0.369 | 28.66 | 0.000 | 5.257 | 6.707 |
| AF_b | 1.556 | 0.062 | 11.18 | 0.000 | 1.440 | 1.682 |
| Heart_valve_dis_b | 1.338 | 0.131 | 2.98 | 0.003 | 1.105 | 1.621 |
| VT_VF_b | 1.602 | 0.256 | 2.95 | 0.003 | 1.171 | 2.193 |
| Cardiomyopathy_b | 1.789 | 0.265 | 3.93 | 0.000 | 1.338 | 2.391 |
| CV_procedures_b | 0.855 | 0.027 | -5.01 | 0.000 | 0.804 | 0.909 |
| TIA_stroke_b | 3.048 | 0.112 | 30.28 | 0.000 | 2.835 | 3.276 |
| PVD_b | 1.875 | 0.078 | 15.20 | 0.000 | 1.729 | 2.033 |
| CKD_b | 1.435 | 0.037 | 13.90 | 0.000 | 1.364 | 1.510 |
| Hypothyroidism_b | 0.995 | 0.034 | -0.16 | 0.874 | 0.930 | 1.064 |
| Liver_dis_b | 1.333 | 0.256 | 1.50 | 0.135 | 0.915 | 1.944 |
| Lupus_b | 1.702 | 0.267 | 3.39 | 0.001 | 1.251 | 2.315 |
| Erectile_dysfunction_b | 0.865 | 0.027 | -4.61 | 0.000 | 0.813 | 0.920 |
| Any_tumour_b | 1.401 | 0.036 | 13.27 | 0.000 | 1.333 | 1.472 |
| Menopause_b | 0.752 | 0.025 | -8.44 | 0.000 | 0.704 | 0.804 |
| Dementia_b | 4.170 | 0.398 | 14.98 | 0.000 | 3.460 | 5.027 |
| RA_b | 1.281 | 0.063 | 5.01 | 0.000 | 1.163 | 1.411 |
| _cons | 0.005 | 0.000 | -56.11 | 0.000 | 0.004 | 0.006 |

**Performance in validation dataset (20% of practices) - Internal validation - Index AMI cases**

| AUROC (95% CI): 0.824 (0.818; 0.830) |  |
| --- | --- |
|  |  |
|  | % Correctly classified: 74.95 |

### 9) 5-year Composite 2 CV outcome (HF, stroke, & CV mortality)

|  | | | | | | |
| --- | --- | --- | --- | --- | --- | --- |
| _5yr_Comp2_CV_outcome | Odds ratio | Std. err. | z | P>|z| | [95% conf. interval] | |
| age | 1.066 | 0.001 | 68.83 | 0.000 | 1.064 | 1.068 |
|  |  |  |  |  |  |  |
| gender |  |  |  |  |  |  |
| Female | 0.959 | 0.021 | -1.88 | 0.060 | 0.918 | 1.002 |
|  |  |  |  |  |  |  |
| IMD |  |  |  |  |  |  |
| Q2 | 1.083 | 0.030 | 2.83 | 0.005 | 1.025 | 1.144 |
| Q3 | 1.208 | 0.034 | 6.71 | 0.000 | 1.143 | 1.276 |
| Q4 | 1.345 | 0.038 | 10.41 | 0.000 | 1.272 | 1.423 |
| Q5-most deprived | 1.571 | 0.046 | 15.57 | 0.000 | 1.484 | 1.663 |
|  |  |  |  |  |  |  |
| Other than White |  |  |  |  |  |  |
| non-White | 0.921 | 0.035 | -2.20 | 0.028 | 0.855 | 0.991 |
|  |  |  |  |  |  |  |
| Drinker |  |  |  |  |  |  |
| drinker | 0.823 | 0.020 | -8.01 | 0.000 | 0.785 | 0.863 |
|  |  |  |  |  |  |  |
| Smoking_status |  |  |  |  |  |  |
| current smoker | 1.275 | 0.035 | 8.90 | 0.000 | 1.209 | 1.345 |
| ex-smoker | 0.923 | 0.023 | -3.26 | 0.001 | 0.880 | 0.969 |
|  |  |  |  |  |  |  |
| BMI | 0.997 | 0.002 | -1.73 | 0.084 | 0.994 | 1.000 |
| HT_b | 0.928 | 0.020 | -3.50 | 0.000 | 0.889 | 0.967 |
| Antihypertensives_b | 1.067 | 0.029 | 2.37 | 0.018 | 1.011 | 1.127 |
| Hyperlipidaemia_b | 0.968 | 0.024 | -1.33 | 0.183 | 0.923 | 1.015 |
| Lipid_reg_treatment_b | 0.877 | 0.021 | -5.51 | 0.000 | 0.837 | 0.919 |
|  |  |  |  |  |  |  |
| Diabetes_type_b |  |  |  |  |  |  |
| T1DM | 2.324 | 0.261 | 7.50 | 0.000 | 1.864 | 2.896 |
| T2DM | 1.088 | 0.052 | 1.77 | 0.077 | 0.991 | 1.195 |
| DM - nos | 1.418 | 0.126 | 3.93 | 0.000 | 1.191 | 1.689 |
|  |  |  |  |  |  |  |
| Antidiabetics_b | 1.809 | 0.093 | 11.52 | 0.000 | 1.635 | 2.001 |
| Diuretics_b | 1.617 | 0.038 | 20.52 | 0.000 | 1.544 | 1.693 |
| Anticoagulants_b | 1.277 | 0.053 | 5.87 | 0.000 | 1.177 | 1.386 |
| Antiplatelets_b | 1.233 | 0.028 | 9.24 | 0.000 | 1.179 | 1.289 |
| HF_b | 6.406 | 0.376 | 31.66 | 0.000 | 5.710 | 7.186 |
| AF_b | 1.683 | 0.064 | 13.71 | 0.000 | 1.563 | 1.813 |
| Heart_valve_dis_b | 1.441 | 0.136 | 3.86 | 0.000 | 1.197 | 1.735 |
| VT_VF_b | 1.588 | 0.247 | 2.98 | 0.003 | 1.171 | 2.154 |
| Cardiomyopathy_b | 1.671 | 0.239 | 3.59 | 0.000 | 1.263 | 2.211 |
| CV_procedures_b | 0.878 | 0.027 | -4.17 | 0.000 | 0.826 | 0.933 |
| TIA_stroke_b | 3.410 | 0.121 | 34.63 | 0.000 | 3.181 | 3.655 |
| PVD_b | 1.890 | 0.075 | 16.03 | 0.000 | 1.748 | 2.043 |
| CKD_b | 1.378 | 0.034 | 12.83 | 0.000 | 1.312 | 1.447 |
| Hypothyroidism_b | 1.011 | 0.034 | 0.32 | 0.751 | 0.946 | 1.080 |
| Liver_dis_b | 0.925 | 0.190 | -0.38 | 0.703 | 0.619 | 1.382 |
| Lupus_b | 1.750 | 0.272 | 3.61 | 0.000 | 1.291 | 2.373 |
| Erectile_dysfunction_b | 0.881 | 0.028 | -3.98 | 0.000 | 0.828 | 0.938 |
| Any_tumour_b | 1.135 | 0.028 | 5.12 | 0.000 | 1.081 | 1.191 |
| Menopause_b | 0.763 | 0.026 | -8.01 | 0.000 | 0.714 | 0.815 |
| Dementia_b | 2.668 | 0.202 | 12.98 | 0.000 | 2.301 | 3.095 |
| RA_b | 1.230 | 0.060 | 4.26 | 0.000 | 1.119 | 1.354 |
| _cons | 0.005 | 0.000 | -56.43 | 0.000 | 0.004 | 0.005 |

**Performance in validation dataset (20% of practices) - Internal validation - Index AMI cases**

| AUROC (95% CI): 0.818 (0.812; 0.825) |  |
| --- | --- |
|  |  |
|  | % Correctly classified: 75.03 |

## External validation (CPRD GOLD) - with therapies

### 1) 5-year all-cause mortality

|  | | | | | | |
| --- | --- | --- | --- | --- | --- | --- |
| _5year_All_cause_death | Odds ratio | Std. err. | z | P>|z| | [95% conf. interval] | |
| age | 1.095 | 0.001 | 80.21 | 0.000 | 1.092 | 1.097 |
|  |  |  |  |  |  |  |
| gender |  |  |  |  |  |  |
| Female | 0.903 | 0.021 | -4.30 | 0.000 | 0.862 | 0.946 |
|  |  |  |  |  |  |  |
| IMD |  |  |  |  |  |  |
| Q2 | 1.095 | 0.034 | 2.94 | 0.003 | 1.031 | 1.163 |
| Q3 | 1.237 | 0.038 | 6.90 | 0.000 | 1.165 | 1.315 |
| Q4 | 1.336 | 0.042 | 9.23 | 0.000 | 1.256 | 1.420 |
| Q5-most deprived | 1.477 | 0.047 | 12.20 | 0.000 | 1.387 | 1.572 |
|  |  |  |  |  |  |  |
| Other than White |  |  |  |  |  |  |
| non-White | 0.733 | 0.033 | -6.93 | 0.000 | 0.671 | 0.800 |
|  |  |  |  |  |  |  |
| Drinker |  |  |  |  |  |  |
| drinker | 0.780 | 0.020 | -9.65 | 0.000 | 0.742 | 0.821 |
|  |  |  |  |  |  |  |
| Smoking_status |  |  |  |  |  |  |
| current smoker | 1.668 | 0.050 | 16.93 | 0.000 | 1.572 | 1.769 |
| ex-smoker | 0.956 | 0.025 | -1.67 | 0.094 | 0.908 | 1.008 |
|  |  |  |  |  |  |  |
| BMI | 0.973 | 0.002 | -13.61 | 0.000 | 0.970 | 0.977 |
| HT_b | 0.823 | 0.019 | -8.56 | 0.000 | 0.787 | 0.861 |
| Antihypertensives_b | 1.104 | 0.037 | 2.97 | 0.003 | 1.034 | 1.178 |
| Hyperlipidaemia_b | 0.878 | 0.023 | -5.01 | 0.000 | 0.835 | 0.924 |
| Lipid_reg_treatment_b | 0.820 | 0.021 | -7.58 | 0.000 | 0.780 | 0.864 |
|  |  |  |  |  |  |  |
| Diabetes_type_b |  |  |  |  |  |  |
| T1DM | 3.611 | 0.423 | 10.95 | 0.000 | 2.870 | 4.544 |
| T2DM | 1.114 | 0.056 | 2.15 | 0.032 | 1.009 | 1.229 |
| DM - nos | 1.730 | 0.154 | 6.17 | 0.000 | 1.454 | 2.059 |
|  |  |  |  |  |  |  |
| Antidiabetics_b | 1.732 | 0.093 | 10.25 | 0.000 | 1.559 | 1.924 |
| Diuretics_b | 1.710 | 0.045 | 20.49 | 0.000 | 1.624 | 1.800 |
| Anticoagulants_b | 1.267 | 0.050 | 5.99 | 0.000 | 1.172 | 1.369 |
| Antiplatelets_b | 1.285 | 0.032 | 10.07 | 0.000 | 1.224 | 1.349 |
| HF_b | 2.065 | 0.081 | 18.51 | 0.000 | 1.912 | 2.230 |
| AF_b | 1.272 | 0.044 | 6.92 | 0.000 | 1.188 | 1.362 |
| Heart_valve_dis_b | 1.413 | 0.119 | 4.12 | 0.000 | 1.199 | 1.666 |
| VT_VF_b | 1.164 | 0.174 | 1.02 | 0.309 | 0.869 | 1.559 |
| Cardiomyopathy_b | 1.012 | 0.131 | 0.10 | 0.924 | 0.786 | 1.305 |
| CV_procedures_b | 0.818 | 0.027 | -6.01 | 0.000 | 0.766 | 0.873 |
| TIA_stroke_b | 1.484 | 0.045 | 12.95 | 0.000 | 1.398 | 1.575 |
| PVD_b | 1.807 | 0.067 | 15.93 | 0.000 | 1.680 | 1.944 |
| CKD_b | 1.397 | 0.034 | 13.78 | 0.000 | 1.332 | 1.465 |
| Hypothyroidism_b | 0.968 | 0.033 | -0.93 | 0.353 | 0.905 | 1.036 |
| Liver_dis_b | 1.581 | 0.361 | 2.00 | 0.045 | 1.010 | 2.474 |
| Lupus_b | 1.803 | 0.295 | 3.61 | 0.000 | 1.309 | 2.483 |
| Erectile_dysfunction_b | 0.784 | 0.029 | -6.61 | 0.000 | 0.729 | 0.842 |
| Any_tumour_b | 1.582 | 0.039 | 18.43 | 0.000 | 1.506 | 1.661 |
| Menopause_b | 0.728 | 0.027 | -8.51 | 0.000 | 0.676 | 0.783 |
| Dementia_b | 4.194 | 0.298 | 20.18 | 0.000 | 3.649 | 4.820 |
| RA_b | 1.426 | 0.072 | 7.01 | 0.000 | 1.291 | 1.575 |
| _cons | 0.001 | 0.000 | -65.79 | 0.000 | 0.000 | 0.001 |

**Performance in validation dataset (CPRD GOLD) - External validation - Index AMI cases**

| AUROC (95% CI): 0.841 (0.835; 0.847) |  |
| --- | --- |
|  |  |
|  | % Correctly classified: 77.01 |

### 2) 5-year Stroke

|  | | | | | | |
| --- | --- | --- | --- | --- | --- | --- |
| _5year_Stroke | Odds ratio | Std. err. | z | P>|z| | [95% conf. interval] | |
| age | 1.064 | 0.001 | 53.22 | 0.000 | 1.062 | 1.066 |
|  |  |  |  |  |  |  |
| gender |  |  |  |  |  |  |
| Female | 1.077 | 0.027 | 2.95 | 0.003 | 1.025 | 1.131 |
|  |  |  |  |  |  |  |
| IMD |  |  |  |  |  |  |
| Q2 | 1.005 | 0.033 | 0.14 | 0.889 | 0.942 | 1.072 |
| Q3 | 1.112 | 0.037 | 3.23 | 0.001 | 1.043 | 1.187 |
| Q4 | 1.213 | 0.040 | 5.81 | 0.000 | 1.137 | 1.295 |
| Q5-most deprived | 1.276 | 0.043 | 7.17 | 0.000 | 1.193 | 1.363 |
|  |  |  |  |  |  |  |
| Other than White |  |  |  |  |  |  |
| non-White | 0.921 | 0.043 | -1.76 | 0.079 | 0.841 | 1.009 |
|  |  |  |  |  |  |  |
| Drinker |  |  |  |  |  |  |
| drinker | 0.922 | 0.025 | -3.01 | 0.003 | 0.875 | 0.972 |
|  |  |  |  |  |  |  |
| Smoking_status |  |  |  |  |  |  |
| current smoker | 1.260 | 0.040 | 7.27 | 0.000 | 1.184 | 1.341 |
| ex-smoker | 0.884 | 0.025 | -4.42 | 0.000 | 0.837 | 0.934 |
|  |  |  |  |  |  |  |
| BMI | 0.981 | 0.002 | -9.00 | 0.000 | 0.977 | 0.985 |
| HT_b | 0.995 | 0.023 | -0.21 | 0.837 | 0.950 | 1.042 |
| Antihypertensives_b | 1.341 | 0.051 | 7.72 | 0.000 | 1.245 | 1.444 |
| Hyperlipidaemia_b | 1.040 | 0.028 | 1.46 | 0.145 | 0.987 | 1.097 |
| Lipid_reg_treatment_b | 0.843 | 0.023 | -6.20 | 0.000 | 0.799 | 0.890 |
|  |  |  |  |  |  |  |
| Diabetes_type_b |  |  |  |  |  |  |
| T1DM | 1.742 | 0.208 | 4.65 | 0.000 | 1.378 | 2.201 |
| T2DM | 0.919 | 0.049 | -1.59 | 0.112 | 0.828 | 1.020 |
| DM - nos | 0.991 | 0.091 | -0.10 | 0.922 | 0.827 | 1.188 |
|  |  |  |  |  |  |  |
| Antidiabetics_b | 1.922 | 0.108 | 11.60 | 0.000 | 1.721 | 2.146 |
| Diuretics_b | 1.376 | 0.038 | 11.45 | 0.000 | 1.303 | 1.454 |
| Anticoagulants_b | 1.024 | 0.041 | 0.59 | 0.557 | 0.946 | 1.108 |
| Antiplatelets_b | 1.371 | 0.036 | 11.94 | 0.000 | 1.302 | 1.444 |
| HF_b | 1.402 | 0.052 | 9.09 | 0.000 | 1.304 | 1.508 |
| AF_b | 1.587 | 0.055 | 13.38 | 0.000 | 1.483 | 1.698 |
| Heart_valve_dis_b | 1.311 | 0.107 | 3.31 | 0.001 | 1.117 | 1.539 |
| VT_VF_b | 1.077 | 0.163 | 0.49 | 0.626 | 0.800 | 1.449 |
| Cardiomyopathy_b | 1.187 | 0.155 | 1.32 | 0.187 | 0.920 | 1.533 |
| CV_procedures_b | 0.859 | 0.030 | -4.32 | 0.000 | 0.801 | 0.920 |
| TIA_stroke_b | 6.851 | 0.212 | 62.10 | 0.000 | 6.448 | 7.280 |
| PVD_b | 1.716 | 0.063 | 14.61 | 0.000 | 1.596 | 1.844 |
| CKD_b | 1.294 | 0.032 | 10.36 | 0.000 | 1.232 | 1.358 |
| Hypothyroidism_b | 1.087 | 0.038 | 2.36 | 0.018 | 1.014 | 1.165 |
| Liver_dis_b | 1.644 | 0.393 | 2.08 | 0.038 | 1.029 | 2.625 |
| Lupus_b | 1.678 | 0.285 | 3.04 | 0.002 | 1.202 | 2.342 |
| Erectile_dysfunction_b | 1.022 | 0.040 | 0.56 | 0.572 | 0.947 | 1.103 |
| Any_tumour_b | 1.067 | 0.028 | 2.45 | 0.014 | 1.013 | 1.124 |
| Menopause_b | 0.841 | 0.033 | -4.39 | 0.000 | 0.778 | 0.908 |
| Dementia_b | 3.260 | 0.194 | 19.86 | 0.000 | 2.901 | 3.663 |
| RA_b | 0.894 | 0.050 | -2.00 | 0.045 | 0.801 | 0.998 |
| _cons | 0.002 | 0.000 | -53.44 | 0.000 | 0.001 | 0.002 |

**Performance in validation dataset (CPRD GOLD) - External validation - Index AMI cases**

| AUROC (95% CI): 0.830 (0.823; 0.837) |  |
| --- | --- |
|  |  |
|  | % Correctly classified: 77.88 |

### 3) 5-year heart failure (HF)

|  | | | | | | |
| --- | --- | --- | --- | --- | --- | --- |
| _5year_HF | Odds ratio | Std. err. | z | P>|z| | [95% conf. interval] | |
| age | 1.061 | 0.001 | 61.78 | 0.000 | 1.059 | 1.063 |
|  |  |  |  |  |  |  |
| gender |  |  |  |  |  |  |
| Female | 0.901 | 0.020 | -4.69 | 0.000 | 0.863 | 0.941 |
|  |  |  |  |  |  |  |
| IMD |  |  |  |  |  |  |
| Q2 | 1.067 | 0.030 | 2.29 | 0.022 | 1.009 | 1.129 |
| Q3 | 1.114 | 0.032 | 3.78 | 0.000 | 1.054 | 1.179 |
| Q4 | 1.261 | 0.036 | 8.03 | 0.000 | 1.192 | 1.335 |
| Q5-most deprived | 1.457 | 0.043 | 12.82 | 0.000 | 1.375 | 1.543 |
|  |  |  |  |  |  |  |
| Other than White |  |  |  |  |  |  |
| non-White | 1.055 | 0.041 | 1.40 | 0.163 | 0.979 | 1.138 |
|  |  |  |  |  |  |  |
| Drinker |  |  |  |  |  |  |
| drinker | 0.820 | 0.020 | -8.27 | 0.000 | 0.782 | 0.860 |
|  |  |  |  |  |  |  |
| Smoking_status |  |  |  |  |  |  |
| current smoker | 1.284 | 0.035 | 9.03 | 0.000 | 1.216 | 1.355 |
| ex-smoker | 0.973 | 0.024 | -1.10 | 0.273 | 0.927 | 1.021 |
|  |  |  |  |  |  |  |
| BMI | 1.003 | 0.002 | 1.48 | 0.138 | 0.999 | 1.006 |
| HT_b | 0.905 | 0.019 | -4.70 | 0.000 | 0.868 | 0.943 |
| Antihypertensives_b | 0.995 | 0.029 | -0.17 | 0.866 | 0.939 | 1.054 |
| Hyperlipidaemia_b | 0.979 | 0.024 | -0.86 | 0.387 | 0.934 | 1.027 |
| Lipid_reg_treatment_b | 0.885 | 0.021 | -5.07 | 0.000 | 0.844 | 0.928 |
|  |  |  |  |  |  |  |
| Diabetes_type_b |  |  |  |  |  |  |
| T1DM | 2.279 | 0.248 | 7.57 | 0.000 | 1.841 | 2.820 |
| T2DM | 1.129 | 0.053 | 2.59 | 0.010 | 1.030 | 1.238 |
| DM - nos | 1.397 | 0.118 | 3.95 | 0.000 | 1.183 | 1.649 |
|  |  |  |  |  |  |  |
| Antidiabetics_b | 1.681 | 0.084 | 10.36 | 0.000 | 1.524 | 1.854 |
| Diuretics_b | 1.708 | 0.041 | 22.31 | 0.000 | 1.630 | 1.790 |
| Anticoagulants_b | 1.250 | 0.048 | 5.77 | 0.000 | 1.158 | 1.348 |
| Antiplatelets_b | 1.111 | 0.026 | 4.56 | 0.000 | 1.062 | 1.162 |
| HF_b | 7.684 | 0.384 | 40.78 | 0.000 | 6.967 | 8.475 |
| AF_b | 1.639 | 0.056 | 14.45 | 0.000 | 1.533 | 1.753 |
| Heart_valve_dis_b | 1.805 | 0.156 | 6.83 | 0.000 | 1.523 | 2.138 |
| VT_VF_b | 1.424 | 0.207 | 2.44 | 0.015 | 1.072 | 1.893 |
| Cardiomyopathy_b | 2.245 | 0.307 | 5.91 | 0.000 | 1.717 | 2.936 |
| CV_procedures_b | 0.935 | 0.029 | -2.16 | 0.031 | 0.880 | 0.994 |
| TIA_stroke_b | 1.296 | 0.038 | 8.80 | 0.000 | 1.223 | 1.373 |
| PVD_b | 1.721 | 0.062 | 15.06 | 0.000 | 1.603 | 1.846 |
| CKD_b | 1.424 | 0.033 | 15.11 | 0.000 | 1.360 | 1.491 |
| Hypothyroidism_b | 1.038 | 0.034 | 1.15 | 0.250 | 0.974 | 1.108 |
| Liver_dis_b | 1.195 | 0.249 | 0.86 | 0.392 | 0.795 | 1.797 |
| Lupus_b | 1.804 | 0.273 | 3.90 | 0.000 | 1.341 | 2.426 |
| Erectile_dysfunction_b | 0.932 | 0.031 | -2.14 | 0.032 | 0.874 | 0.994 |
| Any_tumour_b | 1.160 | 0.028 | 6.17 | 0.000 | 1.107 | 1.216 |
| Menopause_b | 0.767 | 0.027 | -7.63 | 0.000 | 0.716 | 0.821 |
| Dementia_b | 2.185 | 0.131 | 13.00 | 0.000 | 1.942 | 2.458 |
| RA_b | 1.254 | 0.060 | 4.72 | 0.000 | 1.142 | 1.378 |
| _cons | 0.004 | 0.000 | -56.99 | 0.000 | 0.003 | 0.004 |

**Performance in validation dataset (CPRD GOLD) - External validation - Index AMI cases**

| AUROC (95% CI): 0.780 (0.773; 0.787) |  |
| --- | --- |
|  |  |
|  | % Correctly classified: 72.29 |

### 4) 5-year Recurrent MI

|  | | | | | | |
| --- | --- | --- | --- | --- | --- | --- |
| _5year_Recurrent_MI | Odds ratio | Std. err. | z | P>|z| | [95% conf. interval] | |
| age | 1.030 | 0.001 | 31.81 | 0.000 | 1.028 | 1.032 |
|  |  |  |  |  |  |  |
| gender |  |  |  |  |  |  |
| Female | 0.981 | 0.022 | -0.85 | 0.396 | 0.940 | 1.025 |
|  |  |  |  |  |  |  |
| IMD |  |  |  |  |  |  |
| Q2 | 1.088 | 0.031 | 2.96 | 0.003 | 1.029 | 1.150 |
| Q3 | 1.041 | 0.030 | 1.41 | 0.159 | 0.984 | 1.102 |
| Q4 | 1.056 | 0.031 | 1.88 | 0.061 | 0.998 | 1.118 |
| Q5-most deprived | 1.139 | 0.034 | 4.40 | 0.000 | 1.075 | 1.206 |
|  |  |  |  |  |  |  |
| Other than White |  |  |  |  |  |  |
| non-White | 1.086 | 0.042 | 2.15 | 0.031 | 1.007 | 1.171 |
|  |  |  |  |  |  |  |
| Drinker |  |  |  |  |  |  |
| drinker | 0.900 | 0.021 | -4.42 | 0.000 | 0.859 | 0.943 |
|  |  |  |  |  |  |  |
| Smoking_status |  |  |  |  |  |  |
| current smoker | 1.163 | 0.032 | 5.50 | 0.000 | 1.102 | 1.228 |
| ex-smoker | 0.927 | 0.023 | -3.08 | 0.002 | 0.883 | 0.973 |
|  |  |  |  |  |  |  |
| BMI | 0.990 | 0.002 | -5.62 | 0.000 | 0.986 | 0.993 |
| HT_b | 0.957 | 0.020 | -2.07 | 0.038 | 0.918 | 0.998 |
| Antihypertensives_b | 0.957 | 0.029 | -1.45 | 0.148 | 0.902 | 1.016 |
| Hyperlipidaemia_b | 1.028 | 0.025 | 1.15 | 0.249 | 0.981 | 1.078 |
| Lipid_reg_treatment_b | 0.873 | 0.021 | -5.61 | 0.000 | 0.832 | 0.915 |
|  |  |  |  |  |  |  |
| Diabetes_type_b |  |  |  |  |  |  |
| T1DM | 2.039 | 0.210 | 6.92 | 0.000 | 1.667 | 2.495 |
| T2DM | 0.979 | 0.047 | -0.44 | 0.661 | 0.892 | 1.075 |
| DM - nos | 1.268 | 0.103 | 2.93 | 0.003 | 1.082 | 1.486 |
|  |  |  |  |  |  |  |
| Antidiabetics_b | 1.454 | 0.073 | 7.43 | 0.000 | 1.317 | 1.605 |
| Diuretics_b | 1.347 | 0.034 | 11.83 | 0.000 | 1.282 | 1.416 |
| Anticoagulants_b | 1.041 | 0.038 | 1.08 | 0.278 | 0.968 | 1.119 |
| Antiplatelets_b | 1.203 | 0.028 | 7.83 | 0.000 | 1.149 | 1.261 |
| HF_b | 1.462 | 0.050 | 11.18 | 0.000 | 1.368 | 1.562 |
| AF_b | 1.121 | 0.036 | 3.54 | 0.000 | 1.052 | 1.194 |
| Heart_valve_dis_b | 1.331 | 0.099 | 3.85 | 0.000 | 1.151 | 1.539 |
| VT_VF_b | 1.006 | 0.138 | 0.04 | 0.966 | 0.768 | 1.317 |
| Cardiomyopathy_b | 0.817 | 0.102 | -1.62 | 0.106 | 0.639 | 1.044 |
| CV_procedures_b | 0.895 | 0.028 | -3.52 | 0.000 | 0.842 | 0.952 |
| TIA_stroke_b | 1.250 | 0.035 | 7.91 | 0.000 | 1.183 | 1.321 |
| PVD_b | 1.479 | 0.050 | 11.56 | 0.000 | 1.384 | 1.580 |
| CKD_b | 1.268 | 0.029 | 10.31 | 0.000 | 1.212 | 1.326 |
| Hypothyroidism_b | 1.035 | 0.033 | 1.07 | 0.286 | 0.972 | 1.103 |
| Liver_dis_b | 2.099 | 0.385 | 4.04 | 0.000 | 1.465 | 3.007 |
| Lupus_b | 1.520 | 0.225 | 2.83 | 0.005 | 1.137 | 2.031 |
| Erectile_dysfunction_b | 0.858 | 0.029 | -4.51 | 0.000 | 0.802 | 0.917 |
| Any_tumour_b | 1.161 | 0.028 | 6.25 | 0.000 | 1.108 | 1.217 |
| Menopause_b | 0.757 | 0.027 | -7.77 | 0.000 | 0.705 | 0.812 |
| Dementia_b | 2.278 | 0.118 | 15.83 | 0.000 | 2.057 | 2.522 |
| RA_b | 0.908 | 0.046 | -1.91 | 0.057 | 0.823 | 1.003 |
| _cons | 0.033 | 0.003 | -35.24 | 0.000 | 0.027 | 0.040 |

**Performance in validation dataset (CPRD GOLD) - External validation - Index AMI cases**

| AUROC (95% CI): 0.641 (0.633; 0.649) |  |
| --- | --- |
|  |  |
|  | % Correctly classified: 57.85 |

### 5) 5-year CV mortality

|  | | | | | | |
| --- | --- | --- | --- | --- | --- | --- |
| _5year_CV_death | Odds ratio | Std. err. | z | P>|z| | [95% conf. interval] | |
| age | 1.100 | 0.002 | 68.37 | 0.000 | 1.097 | 1.103 |
|  |  |  |  |  |  |  |
| gender |  |  |  |  |  |  |
| Female | 0.776 | 0.021 | -9.51 | 0.000 | 0.736 | 0.817 |
|  |  |  |  |  |  |  |
| IMD |  |  |  |  |  |  |
| Q2 | 1.085 | 0.039 | 2.30 | 0.021 | 1.012 | 1.163 |
| Q3 | 1.139 | 0.041 | 3.65 | 0.000 | 1.062 | 1.222 |
| Q4 | 1.293 | 0.047 | 7.13 | 0.000 | 1.205 | 1.387 |
| Q5-most deprived | 1.368 | 0.050 | 8.51 | 0.000 | 1.273 | 1.471 |
|  |  |  |  |  |  |  |
| Other than White |  |  |  |  |  |  |
| non-White | 0.776 | 0.042 | -4.73 | 0.000 | 0.698 | 0.862 |
|  |  |  |  |  |  |  |
| Drinker |  |  |  |  |  |  |
| drinker | 0.797 | 0.023 | -7.98 | 0.000 | 0.754 | 0.843 |
|  |  |  |  |  |  |  |
| Smoking_status |  |  |  |  |  |  |
| current smoker | 1.329 | 0.045 | 8.31 | 0.000 | 1.242 | 1.421 |
| ex-smoker | 0.810 | 0.024 | -7.10 | 0.000 | 0.764 | 0.858 |
|  |  |  |  |  |  |  |
| BMI | 0.983 | 0.002 | -7.58 | 0.000 | 0.978 | 0.987 |
| HT_b | 0.860 | 0.022 | -5.96 | 0.000 | 0.819 | 0.904 |
| Antihypertensives_b | 1.105 | 0.048 | 2.28 | 0.023 | 1.014 | 1.204 |
| Hyperlipidaemia_b | 0.985 | 0.029 | -0.53 | 0.598 | 0.930 | 1.043 |
| Lipid_reg_treatment_b | 0.784 | 0.023 | -8.19 | 0.000 | 0.740 | 0.831 |
|  |  |  |  |  |  |  |
| Diabetes_type_b |  |  |  |  |  |  |
| T1DM | 5.453 | 0.680 | 13.60 | 0.000 | 4.271 | 6.963 |
| T2DM | 1.259 | 0.069 | 4.21 | 0.000 | 1.131 | 1.402 |
| DM - nos | 1.880 | 0.177 | 6.69 | 0.000 | 1.562 | 2.262 |
|  |  |  |  |  |  |  |
| Antidiabetics_b | 1.586 | 0.092 | 7.95 | 0.000 | 1.415 | 1.777 |
| Diuretics_b | 1.847 | 0.058 | 19.45 | 0.000 | 1.736 | 1.965 |
| Anticoagulants_b | 1.201 | 0.049 | 4.44 | 0.000 | 1.108 | 1.301 |
| Antiplatelets_b | 1.373 | 0.040 | 11.00 | 0.000 | 1.297 | 1.452 |
| HF_b | 2.475 | 0.092 | 24.29 | 0.000 | 2.301 | 2.663 |
| AF_b | 1.364 | 0.048 | 8.77 | 0.000 | 1.272 | 1.461 |
| Heart_valve_dis_b | 1.612 | 0.134 | 5.76 | 0.000 | 1.370 | 1.896 |
| VT_VF_b | 1.418 | 0.218 | 2.27 | 0.023 | 1.049 | 1.918 |
| Cardiomyopathy_b | 1.239 | 0.170 | 1.56 | 0.120 | 0.946 | 1.622 |
| CV_procedures_b | 0.866 | 0.033 | -3.78 | 0.000 | 0.803 | 0.933 |
| TIA_stroke_b | 1.537 | 0.048 | 13.74 | 0.000 | 1.446 | 1.634 |
| PVD_b | 1.960 | 0.075 | 17.70 | 0.000 | 1.819 | 2.111 |
| CKD_b | 1.351 | 0.035 | 11.70 | 0.000 | 1.285 | 1.421 |
| Hypothyroidism_b | 0.937 | 0.035 | -1.72 | 0.086 | 0.870 | 1.009 |
| Liver_dis_b | 1.390 | 0.405 | 1.13 | 0.258 | 0.786 | 2.459 |
| Lupus_b | 2.234 | 0.402 | 4.46 | 0.000 | 1.570 | 3.179 |
| Erectile_dysfunction_b | 0.741 | 0.034 | -6.59 | 0.000 | 0.678 | 0.810 |
| Any_tumour_b | 0.968 | 0.027 | -1.16 | 0.244 | 0.917 | 1.022 |
| Menopause_b | 0.707 | 0.032 | -7.65 | 0.000 | 0.647 | 0.772 |
| Dementia_b | 3.157 | 0.183 | 19.81 | 0.000 | 2.817 | 3.537 |
| RA_b | 1.489 | 0.084 | 7.09 | 0.000 | 1.334 | 1.662 |
| _cons | 0.000 | 0.000 | -63.30 | 0.000 | 0.000 | 0.000 |

**Performance in validation dataset (CPRD GOLD) - External validation - Index AMI cases**

| AUROC (95% CI): 0.854 (0.847; 0.860) |  |
| --- | --- |
|  |  |
|  | % Correctly classified: 80.44 |

### 6) 5-year Composite 1 outcome (HF, stroke, recurrent MI, & all-cause mortality)

|  | | | | | | |
| --- | --- | --- | --- | --- | --- | --- |
| _5yr_Comp1_outcome | Odds ratio | Std. err. | z | P>|z| | [95% conf. interval] | |
| age | 1.055 | 0.001 | 62.92 | 0.000 | 1.054 | 1.057 |
|  |  |  |  |  |  |  |
| gender |  |  |  |  |  |  |
| Female | 1.005 | 0.022 | 0.21 | 0.834 | 0.963 | 1.048 |
|  |  |  |  |  |  |  |
| IMD |  |  |  |  |  |  |
| Q2 | 1.098 | 0.029 | 3.51 | 0.000 | 1.042 | 1.157 |
| Q3 | 1.204 | 0.032 | 6.90 | 0.000 | 1.142 | 1.269 |
| Q4 | 1.274 | 0.035 | 8.90 | 0.000 | 1.208 | 1.344 |
| Q5-most deprived | 1.492 | 0.041 | 14.42 | 0.000 | 1.413 | 1.576 |
|  |  |  |  |  |  |  |
| Other than White |  |  |  |  |  |  |
| non-White | 0.962 | 0.034 | -1.09 | 0.276 | 0.898 | 1.031 |
|  |  |  |  |  |  |  |
| Drinker |  |  |  |  |  |  |
| drinker | 0.887 | 0.021 | -4.97 | 0.000 | 0.847 | 0.930 |
|  |  |  |  |  |  |  |
| Smoking_status |  |  |  |  |  |  |
| current smoker | 1.304 | 0.034 | 10.16 | 0.000 | 1.239 | 1.373 |
| ex-smoker | 0.945 | 0.023 | -2.38 | 0.017 | 0.902 | 0.990 |
|  |  |  |  |  |  |  |
| BMI | 0.992 | 0.002 | -4.84 | 0.000 | 0.989 | 0.995 |
| HT_b | 0.893 | 0.019 | -5.34 | 0.000 | 0.856 | 0.931 |
| Antihypertensives_b | 1.033 | 0.026 | 1.30 | 0.195 | 0.983 | 1.086 |
| Hyperlipidaemia_b | 0.972 | 0.023 | -1.18 | 0.237 | 0.928 | 1.019 |
| Lipid_reg_treatment_b | 0.839 | 0.019 | -7.58 | 0.000 | 0.801 | 0.878 |
|  |  |  |  |  |  |  |
| Diabetes_type_b |  |  |  |  |  |  |
| T1DM | 2.027 | 0.229 | 6.27 | 0.000 | 1.625 | 2.528 |
| T2DM | 1.100 | 0.052 | 1.99 | 0.046 | 1.002 | 1.208 |
| DM - nos | 1.506 | 0.137 | 4.50 | 0.000 | 1.260 | 1.800 |
|  |  |  |  |  |  |  |
| Antidiabetics_b | 1.657 | 0.085 | 9.82 | 0.000 | 1.498 | 1.833 |
| Diuretics_b | 1.575 | 0.036 | 19.83 | 0.000 | 1.506 | 1.648 |
| Anticoagulants_b | 1.274 | 0.055 | 5.61 | 0.000 | 1.171 | 1.386 |
| Antiplatelets_b | 1.183 | 0.026 | 7.52 | 0.000 | 1.132 | 1.236 |
| HF_b | 5.155 | 0.327 | 25.83 | 0.000 | 4.552 | 5.838 |
| AF_b | 1.566 | 0.063 | 11.19 | 0.000 | 1.447 | 1.694 |
| Heart_valve_dis_b | 1.266 | 0.124 | 2.42 | 0.016 | 1.046 | 1.534 |
| VT_VF_b | 1.323 | 0.207 | 1.79 | 0.073 | 0.974 | 1.797 |
| Cardiomyopathy_b | 2.011 | 0.313 | 4.49 | 0.000 | 1.482 | 2.727 |
| CV_procedures_b | 0.849 | 0.026 | -5.42 | 0.000 | 0.800 | 0.901 |
| TIA_stroke_b | 2.870 | 0.108 | 27.99 | 0.000 | 2.666 | 3.090 |
| PVD_b | 1.829 | 0.077 | 14.41 | 0.000 | 1.685 | 1.986 |
| CKD_b | 1.450 | 0.038 | 14.17 | 0.000 | 1.377 | 1.526 |
| Hypothyroidism_b | 0.998 | 0.034 | -0.07 | 0.947 | 0.933 | 1.067 |
| Liver_dis_b | 2.004 | 0.366 | 3.81 | 0.000 | 1.401 | 2.866 |
| Lupus_b | 1.531 | 0.234 | 2.79 | 0.005 | 1.135 | 2.065 |
| Erectile_dysfunction_b | 0.908 | 0.027 | -3.23 | 0.001 | 0.856 | 0.963 |
| Any_tumour_b | 1.348 | 0.034 | 11.82 | 0.000 | 1.283 | 1.417 |
| Menopause_b | 0.749 | 0.024 | -8.84 | 0.000 | 0.703 | 0.799 |
| Dementia_b | 4.024 | 0.401 | 13.97 | 0.000 | 3.310 | 4.892 |
| RA_b | 1.173 | 0.057 | 3.30 | 0.001 | 1.067 | 1.290 |
| _cons | 0.018 | 0.002 | -45.34 | 0.000 | 0.015 | 0.022 |

**Performance in validation dataset (CPRD GOLD) - External validation - Index AMI cases**

| AUROC (95% CI): 0.731 (0.723; 0.739) |  |
| --- | --- |
|  |  |
|  | % Correctly classified: 64.50 |

### 7) 5-year Composite 1 CV outcome (HF, stroke, recurrent MI, & CV mortality)

|  | | | | | | |
| --- | --- | --- | --- | --- | --- | --- |
| _5yr_Comp1_CV_outcome | Odds ratio | Std. err. | z | P>|z| | [95% conf. interval] | |
| age | 1.051 | 0.001 | 58.72 | 0.000 | 1.049 | 1.053 |
|  |  |  |  |  |  |  |
| gender |  |  |  |  |  |  |
| Female | 1.014 | 0.022 | 0.65 | 0.514 | 0.972 | 1.057 |
|  |  |  |  |  |  |  |
| IMD |  |  |  |  |  |  |
| Q2 | 1.085 | 0.029 | 3.07 | 0.002 | 1.030 | 1.142 |
| Q3 | 1.196 | 0.032 | 6.72 | 0.000 | 1.135 | 1.260 |
| Q4 | 1.276 | 0.034 | 9.02 | 0.000 | 1.210 | 1.345 |
| Q5-most deprived | 1.486 | 0.041 | 14.42 | 0.000 | 1.408 | 1.568 |
|  |  |  |  |  |  |  |
| Other than White |  |  |  |  |  |  |
| non-White | 1.000 | 0.035 | 0.00 | 0.997 | 0.934 | 1.071 |
|  |  |  |  |  |  |  |
| Drinker |  |  |  |  |  |  |
| drinker | 0.899 | 0.021 | -4.50 | 0.000 | 0.858 | 0.942 |
|  |  |  |  |  |  |  |
| Smoking_status |  |  |  |  |  |  |
| current smoker | 1.217 | 0.031 | 7.60 | 0.000 | 1.157 | 1.280 |
| ex-smoker | 0.918 | 0.022 | -3.62 | 0.000 | 0.877 | 0.962 |
|  |  |  |  |  |  |  |
| BMI | 0.998 | 0.002 | -1.24 | 0.214 | 0.995 | 1.001 |
| HT_b | 0.933 | 0.019 | -3.31 | 0.001 | 0.896 | 0.972 |
| Antihypertensives_b | 1.016 | 0.026 | 0.63 | 0.527 | 0.967 | 1.068 |
| Hyperlipidaemia_b | 0.980 | 0.023 | -0.87 | 0.383 | 0.936 | 1.026 |
| Lipid_reg_treatment_b | 0.839 | 0.019 | -7.65 | 0.000 | 0.802 | 0.878 |
|  |  |  |  |  |  |  |
| Diabetes_type_b |  |  |  |  |  |  |
| T1DM | 1.982 | 0.218 | 6.22 | 0.000 | 1.597 | 2.459 |
| T2DM | 1.153 | 0.054 | 3.04 | 0.002 | 1.052 | 1.264 |
| DM - nos | 1.606 | 0.143 | 5.32 | 0.000 | 1.349 | 1.912 |
|  |  |  |  |  |  |  |
| Antidiabetics_b | 1.553 | 0.078 | 8.73 | 0.000 | 1.407 | 1.714 |
| Diuretics_b | 1.545 | 0.035 | 19.25 | 0.000 | 1.478 | 1.615 |
| Anticoagulants_b | 1.285 | 0.054 | 6.02 | 0.000 | 1.184 | 1.395 |
| Antiplatelets_b | 1.214 | 0.027 | 8.83 | 0.000 | 1.163 | 1.267 |
| HF_b | 5.283 | 0.316 | 27.86 | 0.000 | 4.700 | 5.940 |
| AF_b | 1.556 | 0.060 | 11.55 | 0.000 | 1.444 | 1.677 |
| Heart_valve_dis_b | 1.343 | 0.127 | 3.12 | 0.002 | 1.116 | 1.617 |
| VT_VF_b | 1.555 | 0.240 | 2.86 | 0.004 | 1.149 | 2.105 |
| Cardiomyopathy_b | 1.831 | 0.271 | 4.09 | 0.000 | 1.370 | 2.448 |
| CV_procedures_b | 0.843 | 0.025 | -5.69 | 0.000 | 0.795 | 0.894 |
| TIA_stroke_b | 2.954 | 0.106 | 30.09 | 0.000 | 2.753 | 3.170 |
| PVD_b | 1.892 | 0.076 | 15.77 | 0.000 | 1.748 | 2.048 |
| CKD_b | 1.455 | 0.037 | 14.85 | 0.000 | 1.384 | 1.528 |
| Hypothyroidism_b | 0.990 | 0.033 | -0.30 | 0.764 | 0.928 | 1.057 |
| Liver_dis_b | 1.656 | 0.300 | 2.79 | 0.005 | 1.162 | 2.361 |
| Lupus_b | 1.375 | 0.205 | 2.14 | 0.033 | 1.027 | 1.843 |
| Erectile_dysfunction_b | 0.937 | 0.028 | -2.18 | 0.029 | 0.884 | 0.993 |
| Any_tumour_b | 1.160 | 0.028 | 6.06 | 0.000 | 1.105 | 1.216 |
| Menopause_b | 0.746 | 0.024 | -9.08 | 0.000 | 0.700 | 0.794 |
| Dementia_b | 3.372 | 0.290 | 14.15 | 0.000 | 2.850 | 3.991 |
| RA_b | 1.163 | 0.055 | 3.19 | 0.001 | 1.060 | 1.277 |
| _cons | 0.018 | 0.002 | -45.81 | 0.000 | 0.015 | 0.021 |

**Performance in validation dataset (CPRD GOLD) - External validation - Index AMI cases**

| AUROC (95% CI): 0.723 (0.714; 0.731) |  |
| --- | --- |
|  |  |
|  | % Correctly classified: 63.51 |

### 8) 5-year Composite 2 outcome (HF, stroke, & all-cause mortality)

|  | | | | | | |
| --- | --- | --- | --- | --- | --- | --- |
| _5yr_Comp2_outcome | Odds ratio | Std. err. | z | P>|z| | [95% conf. interval] | |
| age | 1.069 | 0.001 | 72.69 | 0.000 | 1.067 | 1.071 |
|  |  |  |  |  |  |  |
| gender |  |  |  |  |  |  |
| Female | 0.981 | 0.022 | -0.88 | 0.381 | 0.938 | 1.025 |
|  |  |  |  |  |  |  |
| IMD |  |  |  |  |  |  |
| Q2 | 1.100 | 0.031 | 3.41 | 0.001 | 1.041 | 1.162 |
| Q3 | 1.235 | 0.035 | 7.50 | 0.000 | 1.169 | 1.305 |
| Q4 | 1.372 | 0.039 | 11.10 | 0.000 | 1.298 | 1.451 |
| Q5-most deprived | 1.622 | 0.047 | 16.64 | 0.000 | 1.532 | 1.717 |
|  |  |  |  |  |  |  |
| Other than White |  |  |  |  |  |  |
| non-White | 0.875 | 0.033 | -3.55 | 0.000 | 0.813 | 0.942 |
|  |  |  |  |  |  |  |
| Drinker |  |  |  |  |  |  |
| drinker | 0.846 | 0.021 | -6.79 | 0.000 | 0.806 | 0.888 |
|  |  |  |  |  |  |  |
| Smoking_status |  |  |  |  |  |  |
| current smoker | 1.374 | 0.038 | 11.62 | 0.000 | 1.302 | 1.450 |
| ex-smoker | 0.944 | 0.023 | -2.33 | 0.020 | 0.899 | 0.991 |
|  |  |  |  |  |  |  |
| BMI | 0.991 | 0.002 | -4.88 | 0.000 | 0.988 | 0.995 |
| HT_b | 0.864 | 0.019 | -6.71 | 0.000 | 0.828 | 0.902 |
| Antihypertensives_b | 1.098 | 0.030 | 3.48 | 0.001 | 1.042 | 1.158 |
| Hyperlipidaemia_b | 0.965 | 0.024 | -1.46 | 0.144 | 0.920 | 1.012 |
| Lipid_reg_treatment_b | 0.850 | 0.020 | -6.75 | 0.000 | 0.811 | 0.891 |
|  |  |  |  |  |  |  |
| Diabetes_type_b |  |  |  |  |  |  |
| T1DM | 2.400 | 0.274 | 7.66 | 0.000 | 1.918 | 3.002 |
| T2DM | 1.102 | 0.054 | 1.99 | 0.047 | 1.001 | 1.212 |
| DM - nos | 1.556 | 0.142 | 4.84 | 0.000 | 1.301 | 1.862 |
|  |  |  |  |  |  |  |
| Antidiabetics_b | 1.784 | 0.093 | 11.05 | 0.000 | 1.610 | 1.976 |
| Diuretics_b | 1.640 | 0.039 | 21.01 | 0.000 | 1.566 | 1.717 |
| Anticoagulants_b | 1.329 | 0.057 | 6.59 | 0.000 | 1.221 | 1.446 |
| Antiplatelets_b | 1.231 | 0.028 | 9.07 | 0.000 | 1.177 | 1.287 |
| HF_b | 5.937 | 0.369 | 28.66 | 0.000 | 5.257 | 6.707 |
| AF_b | 1.556 | 0.062 | 11.18 | 0.000 | 1.440 | 1.682 |
| Heart_valve_dis_b | 1.338 | 0.131 | 2.98 | 0.003 | 1.105 | 1.621 |
| VT_VF_b | 1.602 | 0.256 | 2.95 | 0.003 | 1.171 | 2.193 |
| Cardiomyopathy_b | 1.789 | 0.265 | 3.93 | 0.000 | 1.338 | 2.391 |
| CV_procedures_b | 0.855 | 0.027 | -5.01 | 0.000 | 0.804 | 0.909 |
| TIA_stroke_b | 3.048 | 0.112 | 30.28 | 0.000 | 2.835 | 3.276 |
| PVD_b | 1.875 | 0.078 | 15.20 | 0.000 | 1.729 | 2.033 |
| CKD_b | 1.435 | 0.037 | 13.90 | 0.000 | 1.364 | 1.510 |
| Hypothyroidism_b | 0.995 | 0.034 | -0.16 | 0.874 | 0.930 | 1.064 |
| Liver_dis_b | 1.333 | 0.256 | 1.50 | 0.135 | 0.915 | 1.944 |
| Lupus_b | 1.702 | 0.267 | 3.39 | 0.001 | 1.251 | 2.315 |
| Erectile_dysfunction_b | 0.865 | 0.027 | -4.61 | 0.000 | 0.813 | 0.920 |
| Any_tumour_b | 1.401 | 0.036 | 13.27 | 0.000 | 1.333 | 1.472 |
| Menopause_b | 0.752 | 0.025 | -8.44 | 0.000 | 0.704 | 0.804 |
| Dementia_b | 4.170 | 0.398 | 14.98 | 0.000 | 3.460 | 5.027 |
| RA_b | 1.281 | 0.063 | 5.01 | 0.000 | 1.163 | 1.411 |
| _cons | 0.005 | 0.000 | -56.11 | 0.000 | 0.004 | 0.006 |

**Performance in validation dataset (CPRD GOLD) - External validation - Index AMI cases**

| AUROC (95% CI): 0.808 (0.801; 0.815) |  |
| --- | --- |
|  |  |
|  | % Correctly classified: 73.25 |

### 9) 5-year Composite 2 CV outcome (HF, stroke, & CV mortality)

|  | | | | | | |
| --- | --- | --- | --- | --- | --- | --- |
| _5yr_Comp2_CV_outcome | Odds ratio | Std. err. | z | P>|z| | [95% conf. interval] | |
| age | 1.066 | 0.001 | 68.83 | 0.000 | 1.064 | 1.068 |
|  |  |  |  |  |  |  |
| gender |  |  |  |  |  |  |
| Female | 0.959 | 0.021 | -1.88 | 0.060 | 0.918 | 1.002 |
|  |  |  |  |  |  |  |
| IMD |  |  |  |  |  |  |
| Q2 | 1.083 | 0.030 | 2.83 | 0.005 | 1.025 | 1.144 |
| Q3 | 1.208 | 0.034 | 6.71 | 0.000 | 1.143 | 1.276 |
| Q4 | 1.345 | 0.038 | 10.41 | 0.000 | 1.272 | 1.423 |
| Q5-most deprived | 1.571 | 0.046 | 15.57 | 0.000 | 1.484 | 1.663 |
|  |  |  |  |  |  |  |
| Other than White |  |  |  |  |  |  |
| non-White | 0.921 | 0.035 | -2.20 | 0.028 | 0.855 | 0.991 |
|  |  |  |  |  |  |  |
| Drinker |  |  |  |  |  |  |
| drinker | 0.823 | 0.020 | -8.01 | 0.000 | 0.785 | 0.863 |
|  |  |  |  |  |  |  |
| Smoking_status |  |  |  |  |  |  |
| current smoker | 1.275 | 0.035 | 8.90 | 0.000 | 1.209 | 1.345 |
| ex-smoker | 0.923 | 0.023 | -3.26 | 0.001 | 0.880 | 0.969 |
|  |  |  |  |  |  |  |
| BMI | 0.997 | 0.002 | -1.73 | 0.084 | 0.994 | 1.000 |
| HT_b | 0.928 | 0.020 | -3.50 | 0.000 | 0.889 | 0.967 |
| Antihypertensives_b | 1.067 | 0.029 | 2.37 | 0.018 | 1.011 | 1.127 |
| Hyperlipidaemia_b | 0.968 | 0.024 | -1.33 | 0.183 | 0.923 | 1.015 |
| Lipid_reg_treatment_b | 0.877 | 0.021 | -5.51 | 0.000 | 0.837 | 0.919 |
|  |  |  |  |  |  |  |
| Diabetes_type_b |  |  |  |  |  |  |
| T1DM | 2.324 | 0.261 | 7.50 | 0.000 | 1.864 | 2.896 |
| T2DM | 1.088 | 0.052 | 1.77 | 0.077 | 0.991 | 1.195 |
| DM - nos | 1.418 | 0.126 | 3.93 | 0.000 | 1.191 | 1.689 |
|  |  |  |  |  |  |  |
| Antidiabetics_b | 1.809 | 0.093 | 11.52 | 0.000 | 1.635 | 2.001 |
| Diuretics_b | 1.617 | 0.038 | 20.52 | 0.000 | 1.544 | 1.693 |
| Anticoagulants_b | 1.277 | 0.053 | 5.87 | 0.000 | 1.177 | 1.386 |
| Antiplatelets_b | 1.233 | 0.028 | 9.24 | 0.000 | 1.179 | 1.289 |
| HF_b | 6.406 | 0.376 | 31.66 | 0.000 | 5.710 | 7.186 |
| AF_b | 1.683 | 0.064 | 13.71 | 0.000 | 1.563 | 1.813 |
| Heart_valve_dis_b | 1.441 | 0.136 | 3.86 | 0.000 | 1.197 | 1.735 |
| VT_VF_b | 1.588 | 0.247 | 2.98 | 0.003 | 1.171 | 2.154 |
| Cardiomyopathy_b | 1.671 | 0.239 | 3.59 | 0.000 | 1.263 | 2.211 |
| CV_procedures_b | 0.878 | 0.027 | -4.17 | 0.000 | 0.826 | 0.933 |
| TIA_stroke_b | 3.410 | 0.121 | 34.63 | 0.000 | 3.181 | 3.655 |
| PVD_b | 1.890 | 0.075 | 16.03 | 0.000 | 1.748 | 2.043 |
| CKD_b | 1.378 | 0.034 | 12.83 | 0.000 | 1.312 | 1.447 |
| Hypothyroidism_b | 1.011 | 0.034 | 0.32 | 0.751 | 0.946 | 1.080 |
| Liver_dis_b | 0.925 | 0.190 | -0.38 | 0.703 | 0.619 | 1.382 |
| Lupus_b | 1.750 | 0.272 | 3.61 | 0.000 | 1.291 | 2.373 |
| Erectile_dysfunction_b | 0.881 | 0.028 | -3.98 | 0.000 | 0.828 | 0.938 |
| Any_tumour_b | 1.135 | 0.028 | 5.12 | 0.000 | 1.081 | 1.191 |
| Menopause_b | 0.763 | 0.026 | -8.01 | 0.000 | 0.714 | 0.815 |
| Dementia_b | 2.668 | 0.202 | 12.98 | 0.000 | 2.301 | 3.095 |
| RA_b | 1.230 | 0.060 | 4.26 | 0.000 | 1.119 | 1.354 |
| _cons | 0.005 | 0.000 | -56.43 | 0.000 | 0.004 | 0.005 |

**Performance in validation dataset (CPRD GOLD) - External validation - Index AMI cases**

| AUROC (95% CI): 0.794 (0.787; 0.801) |  |
| --- | --- |
|  |  |
|  | % Correctly classified: 72.46 |

# Internal and external validation - Audit models

## Internal validation (CPRD Aurum) - without therapies

### 1) 1-year all-cause mortality

|  | | | | | | |
| --- | --- | --- | --- | --- | --- | --- |
| _1year_All_cause_death | Odds ratio | Std. err. | z | P>|z| | [95% conf. interval] | |
| age | 1.114 | 0.003 | 46.92 | 0.000 | 1.109 | 1.119 |
|  |  |  |  |  |  |  |
| gender |  |  |  |  |  |  |
| Female | 0.929 | 0.039 | -1.77 | 0.077 | 0.857 | 1.008 |
|  |  |  |  |  |  |  |
| IMD |  |  |  |  |  |  |
| Q2 | 1.109 | 0.061 | 1.89 | 0.058 | 0.996 | 1.234 |
| Q3 | 1.470 | 0.079 | 7.14 | 0.000 | 1.322 | 1.634 |
| Q4 | 1.404 | 0.077 | 6.17 | 0.000 | 1.260 | 1.564 |
| Q5-most deprived | 1.546 | 0.087 | 7.74 | 0.000 | 1.385 | 1.727 |
|  |  |  |  |  |  |  |
| ethnicity2 | 0.599 | 0.049 | -6.28 | 0.000 | 0.511 | 0.703 |
| Alcohol_status3 | 0.786 | 0.035 | -5.36 | 0.000 | 0.720 | 0.859 |
|  |  |  |  |  |  |  |
| Smoking_status |  |  |  |  |  |  |
| current smoker | 2.011 | 0.109 | 12.92 | 0.000 | 1.809 | 2.236 |
| ex-smoker | 1.171 | 0.056 | 3.31 | 0.001 | 1.067 | 1.285 |
|  |  |  |  |  |  |  |
| BMI_2014_f | 0.933 | 0.004 | -17.23 | 0.000 | 0.925 | 0.940 |
| HT_b | 1.027 | 0.036 | 0.74 | 0.460 | 0.958 | 1.100 |
| Hyperlipidaemia_b | 0.889 | 0.034 | -3.11 | 0.002 | 0.826 | 0.958 |
|  |  |  |  |  |  |  |
| Diabetes_type_au_b |  |  |  |  |  |  |
| T1DM | 4.562 | 0.959 | 7.22 | 0.000 | 3.022 | 6.886 |
| T2DM | 2.244 | 0.093 | 19.55 | 0.000 | 2.069 | 2.434 |
| DM - nos | 3.509 | 0.439 | 10.03 | 0.000 | 2.746 | 4.485 |
|  |  |  |  |  |  |  |
| HF_b | 2.233 | 0.089 | 20.18 | 0.000 | 2.065 | 2.414 |
| AF_b | 1.517 | 0.060 | 10.49 | 0.000 | 1.403 | 1.640 |
| Heart_valve_dis_b | 2.152 | 0.235 | 7.03 | 0.000 | 1.738 | 2.664 |
| VT_VF_b | 2.016 | 0.254 | 5.56 | 0.000 | 1.574 | 2.582 |
| Cardiomyopathy_b | 1.602 | 0.214 | 3.52 | 0.000 | 1.233 | 2.082 |
| CV_procedures_b | 0.679 | 0.025 | -10.52 | 0.000 | 0.632 | 0.730 |
| TIA_stroke_b | 1.485 | 0.063 | 9.35 | 0.000 | 1.367 | 1.613 |
| PVD_b | 1.737 | 0.093 | 10.37 | 0.000 | 1.565 | 1.929 |
| CKD_b | 1.522 | 0.055 | 11.63 | 0.000 | 1.418 | 1.634 |
| Hypothyroidism_b | 1.046 | 0.054 | 0.88 | 0.381 | 0.946 | 1.158 |
| Liver_dis_b | 4.311 | 1.281 | 4.92 | 0.000 | 2.407 | 7.719 |
| Lupus_b | 1.080 | 0.340 | 0.25 | 0.806 | 0.583 | 2.003 |
| Erectile_dysfunction_b | 0.813 | 0.045 | -3.71 | 0.000 | 0.729 | 0.907 |
| Any_tumour_b | 1.664 | 0.063 | 13.52 | 0.000 | 1.546 | 1.792 |
| Menopause_b | 0.666 | 0.043 | -6.23 | 0.000 | 0.586 | 0.757 |
| Dementia_b | 4.601 | 0.274 | 25.64 | 0.000 | 4.094 | 5.170 |
| RA_b | 1.849 | 0.143 | 7.96 | 0.000 | 1.589 | 2.151 |
| num_ami_before_31_dec_14 | 0.990 | 0.002 | -4.52 | 0.000 | 0.986 | 0.995 |
| _cons | 0.000 | 0.000 | -39.48 | 0.000 | 0.000 | 0.000 |

**Performance in validation dataset (20% of practices) - Internal validation - audit AMI cases**

| AUROC (95% CI): 0.886 (0.878; 0.895) |  |
| --- | --- |
|  |  |
|  | % Correctly classified: 85.91 |

### 2) 1-year Stroke

|  | | | | | | |
| --- | --- | --- | --- | --- | --- | --- |
| _1year_Stroke | Odds ratio | Std. err. | z | P>|z| | [95% conf. interval] | |
| age | 1.036 | 0.002 | 15.34 | 0.000 | 1.031 | 1.041 |
|  |  |  |  |  |  |  |
| gender |  |  |  |  |  |  |
| Female | 1.065 | 0.054 | 1.24 | 0.214 | 0.964 | 1.177 |
|  |  |  |  |  |  |  |
| IMD |  |  |  |  |  |  |
| Q2 | 1.075 | 0.068 | 1.14 | 0.255 | 0.949 | 1.217 |
| Q3 | 1.055 | 0.068 | 0.84 | 0.404 | 0.930 | 1.198 |
| Q4 | 1.150 | 0.074 | 2.17 | 0.030 | 1.014 | 1.305 |
| Q5-most deprived | 1.189 | 0.078 | 2.66 | 0.008 | 1.046 | 1.351 |
|  |  |  |  |  |  |  |
| ethnicity2 | 0.889 | 0.074 | -1.42 | 0.154 | 0.756 | 1.045 |
| Alcohol_status3 | 0.760 | 0.040 | -5.28 | 0.000 | 0.686 | 0.842 |
|  |  |  |  |  |  |  |
| Smoking_status |  |  |  |  |  |  |
| current smoker | 1.093 | 0.069 | 1.41 | 0.158 | 0.966 | 1.237 |
| ex-smoker | 1.058 | 0.059 | 1.02 | 0.308 | 0.949 | 1.180 |
|  |  |  |  |  |  |  |
| BMI_2014_f | 0.983 | 0.004 | -3.91 | 0.000 | 0.975 | 0.992 |
| HT_b | 1.109 | 0.047 | 2.46 | 0.014 | 1.021 | 1.204 |
| Hyperlipidaemia_b | 1.068 | 0.046 | 1.53 | 0.126 | 0.982 | 1.162 |
|  |  |  |  |  |  |  |
| Diabetes_type_au_b |  |  |  |  |  |  |
| T1DM | 2.562 | 0.558 | 4.32 | 0.000 | 1.672 | 3.926 |
| T2DM | 1.322 | 0.065 | 5.71 | 0.000 | 1.201 | 1.456 |
| DM - nos | 2.691 | 0.356 | 7.48 | 0.000 | 2.076 | 3.488 |
|  |  |  |  |  |  |  |
| HF_b | 1.122 | 0.057 | 2.25 | 0.024 | 1.015 | 1.241 |
| AF_b | 1.377 | 0.066 | 6.69 | 0.000 | 1.254 | 1.513 |
| Heart_valve_dis_b | 1.177 | 0.160 | 1.20 | 0.229 | 0.902 | 1.536 |
| VT_VF_b | 1.092 | 0.181 | 0.53 | 0.597 | 0.788 | 1.512 |
| Cardiomyopathy_b | 0.841 | 0.153 | -0.95 | 0.341 | 0.588 | 1.201 |
| CV_procedures_b | 0.876 | 0.037 | -3.13 | 0.002 | 0.806 | 0.952 |
| TIA_stroke_b | 11.579 | 0.474 | 59.86 | 0.000 | 10.687 | 12.546 |
| PVD_b | 1.202 | 0.077 | 2.86 | 0.004 | 1.059 | 1.363 |
| CKD_b | 1.143 | 0.051 | 3.00 | 0.003 | 1.047 | 1.247 |
| Hypothyroidism_b | 1.076 | 0.067 | 1.19 | 0.235 | 0.953 | 1.215 |
| Liver_dis_b | 1.209 | 0.497 | 0.46 | 0.644 | 0.540 | 2.707 |
| Lupus_b | 1.398 | 0.416 | 1.12 | 0.261 | 0.780 | 2.506 |
| Erectile_dysfunction_b | 1.066 | 0.065 | 1.06 | 0.289 | 0.947 | 1.201 |
| Any_tumour_b | 1.117 | 0.054 | 2.29 | 0.022 | 1.016 | 1.227 |
| Menopause_b | 1.009 | 0.071 | 0.13 | 0.895 | 0.879 | 1.159 |
| Dementia_b | 1.915 | 0.141 | 8.84 | 0.000 | 1.658 | 2.212 |
| RA_b | 0.970 | 0.098 | -0.31 | 0.759 | 0.795 | 1.182 |
| num_ami_before_31_dec_14 | 1.001 | 0.002 | 0.44 | 0.663 | 0.997 | 1.005 |
| _cons | 0.003 | 0.001 | -23.23 | 0.000 | 0.002 | 0.005 |

**Performance in validation dataset (20% of practices) - Internal validation - audit AMI cases**

| AUROC (95% CI): 0.855 (0.841; 0.869) |  |
| --- | --- |
|  |  |
|  | % Correctly classified: 89.33 |

### 3) 1-year heart failure (HF)

|  | | | | | | |
| --- | --- | --- | --- | --- | --- | --- |
| _1year_HF | Odds ratio | Std. err. | z | P>|z| | [95% conf. interval] | |
| age | 1.054 | 0.002 | 28.92 | 0.000 | 1.050 | 1.057 |
|  |  |  |  |  |  |  |
| gender |  |  |  |  |  |  |
| Female | 1.023 | 0.041 | 0.57 | 0.571 | 0.945 | 1.107 |
|  |  |  |  |  |  |  |
| IMD |  |  |  |  |  |  |
| Q2 | 1.162 | 0.060 | 2.92 | 0.003 | 1.051 | 1.284 |
| Q3 | 1.374 | 0.070 | 6.23 | 0.000 | 1.243 | 1.518 |
| Q4 | 1.260 | 0.065 | 4.49 | 0.000 | 1.139 | 1.394 |
| Q5-most deprived | 1.425 | 0.074 | 6.81 | 0.000 | 1.287 | 1.579 |
|  |  |  |  |  |  |  |
| ethnicity2 | 1.115 | 0.070 | 1.72 | 0.085 | 0.985 | 1.261 |
| Alcohol_status3 | 0.784 | 0.033 | -5.79 | 0.000 | 0.722 | 0.851 |
|  |  |  |  |  |  |  |
| Smoking_status |  |  |  |  |  |  |
| current smoker | 1.294 | 0.064 | 5.21 | 0.000 | 1.174 | 1.425 |
| ex-smoker | 1.062 | 0.047 | 1.38 | 0.169 | 0.975 | 1.158 |
|  |  |  |  |  |  |  |
| BMI_2014_f | 1.014 | 0.003 | 4.24 | 0.000 | 1.007 | 1.020 |
| HT_b | 1.125 | 0.037 | 3.58 | 0.000 | 1.055 | 1.200 |
| Hyperlipidaemia_b | 1.064 | 0.036 | 1.80 | 0.071 | 0.995 | 1.137 |
|  |  |  |  |  |  |  |
| Diabetes_type_au_b |  |  |  |  |  |  |
| T1DM | 3.524 | 0.627 | 7.08 | 0.000 | 2.487 | 4.995 |
| T2DM | 1.679 | 0.063 | 13.73 | 0.000 | 1.559 | 1.808 |
| DM - nos | 2.155 | 0.254 | 6.52 | 0.000 | 1.711 | 2.715 |
|  |  |  |  |  |  |  |
| HF_b | 14.724 | 0.494 | 80.20 | 0.000 | 13.787 | 15.724 |
| AF_b | 2.165 | 0.081 | 20.65 | 0.000 | 2.012 | 2.330 |
| Heart_valve_dis_b | 2.148 | 0.232 | 7.09 | 0.000 | 1.738 | 2.653 |
| VT_VF_b | 2.052 | 0.226 | 6.54 | 0.000 | 1.654 | 2.545 |
| Cardiomyopathy_b | 2.272 | 0.245 | 7.60 | 0.000 | 1.839 | 2.808 |
| CV_procedures_b | 0.886 | 0.029 | -3.68 | 0.000 | 0.831 | 0.945 |
| TIA_stroke_b | 1.374 | 0.058 | 7.57 | 0.000 | 1.265 | 1.491 |
| PVD_b | 1.646 | 0.085 | 9.64 | 0.000 | 1.487 | 1.821 |
| CKD_b | 1.518 | 0.052 | 12.06 | 0.000 | 1.418 | 1.624 |
| Hypothyroidism_b | 1.017 | 0.051 | 0.33 | 0.743 | 0.921 | 1.122 |
| Liver_dis_b | 1.913 | 0.531 | 2.34 | 0.019 | 1.110 | 3.295 |
| Lupus_b | 1.228 | 0.318 | 0.80 | 0.426 | 0.740 | 2.039 |
| Erectile_dysfunction_b | 0.966 | 0.045 | -0.75 | 0.456 | 0.881 | 1.059 |
| Any_tumour_b | 1.214 | 0.047 | 5.04 | 0.000 | 1.126 | 1.309 |
| Menopause_b | 0.816 | 0.048 | -3.46 | 0.001 | 0.727 | 0.915 |
| Dementia_b | 1.144 | 0.080 | 1.91 | 0.056 | 0.997 | 1.312 |
| RA_b | 1.348 | 0.102 | 3.94 | 0.000 | 1.162 | 1.564 |
| num_ami_before_31_dec_14 | 1.001 | 0.002 | 0.44 | 0.660 | 0.998 | 1.004 |
| _cons | 0.001 | 0.000 | -38.51 | 0.000 | 0.000 | 0.001 |

**Performance in validation dataset (20% of practices) - Internal validation - audit AMI cases**

| AUROC (95% CI): 0.871 (0.861; 0.880) |  |
| --- | --- |
|  |  |
|  | % Correctly classified: 86.23 |

### 4) 1-year Recurrent MI

|  | | | | | | |
| --- | --- | --- | --- | --- | --- | --- |
| _1year_Recurrent_MI | Odds ratio | Std. err. | z | P>|z| | [95% conf. interval] | |
| age | 1.026 | 0.003 | 8.82 | 0.000 | 1.020 | 1.032 |
|  |  |  |  |  |  |  |
| gender |  |  |  |  |  |  |
| Female | 1.026 | 0.068 | 0.38 | 0.702 | 0.900 | 1.169 |
|  |  |  |  |  |  |  |
| IMD |  |  |  |  |  |  |
| Q2 | 1.009 | 0.088 | 0.10 | 0.921 | 0.851 | 1.196 |
| Q3 | 1.180 | 0.100 | 1.95 | 0.051 | 0.999 | 1.393 |
| Q4 | 1.086 | 0.094 | 0.95 | 0.340 | 0.917 | 1.286 |
| Q5-most deprived | 1.234 | 0.105 | 2.46 | 0.014 | 1.044 | 1.460 |
|  |  |  |  |  |  |  |
| ethnicity2 | 1.153 | 0.111 | 1.49 | 0.137 | 0.956 | 1.391 |
| Alcohol_status3 | 0.836 | 0.057 | -2.62 | 0.009 | 0.732 | 0.956 |
|  |  |  |  |  |  |  |
| Smoking_status |  |  |  |  |  |  |
| current smoker | 1.109 | 0.088 | 1.31 | 0.191 | 0.949 | 1.296 |
| ex-smoker | 0.847 | 0.062 | -2.28 | 0.022 | 0.734 | 0.977 |
|  |  |  |  |  |  |  |
| BMI_2014_f | 0.999 | 0.005 | -0.12 | 0.902 | 0.989 | 1.010 |
| HT_b | 1.151 | 0.064 | 2.55 | 0.011 | 1.033 | 1.283 |
| Hyperlipidaemia_b | 1.122 | 0.063 | 2.05 | 0.040 | 1.005 | 1.253 |
|  |  |  |  |  |  |  |
| Diabetes_type_au_b |  |  |  |  |  |  |
| T1DM | 4.484 | 0.948 | 7.09 | 0.000 | 2.962 | 6.787 |
| T2DM | 1.749 | 0.108 | 9.07 | 0.000 | 1.550 | 1.973 |
| DM - nos | 3.104 | 0.478 | 7.36 | 0.000 | 2.296 | 4.197 |
|  |  |  |  |  |  |  |
| HF_b | 1.278 | 0.086 | 3.66 | 0.000 | 1.121 | 1.458 |
| AF_b | 0.888 | 0.065 | -1.62 | 0.105 | 0.770 | 1.025 |
| Heart_valve_dis_b | 0.953 | 0.204 | -0.22 | 0.822 | 0.627 | 1.449 |
| VT_VF_b | 1.096 | 0.233 | 0.43 | 0.668 | 0.722 | 1.663 |
| Cardiomyopathy_b | 1.420 | 0.289 | 1.72 | 0.086 | 0.952 | 2.117 |
| CV_procedures_b | 0.971 | 0.053 | -0.54 | 0.586 | 0.872 | 1.080 |
| TIA_stroke_b | 1.088 | 0.079 | 1.16 | 0.244 | 0.944 | 1.254 |
| PVD_b | 1.760 | 0.137 | 7.24 | 0.000 | 1.510 | 2.051 |
| CKD_b | 1.238 | 0.075 | 3.54 | 0.000 | 1.100 | 1.393 |
| Hypothyroidism_b | 1.018 | 0.086 | 0.21 | 0.834 | 0.862 | 1.201 |
| Liver_dis_b | 2.411 | 0.860 | 2.47 | 0.014 | 1.198 | 4.849 |
| Lupus_b | 0.357 | 0.256 | -1.44 | 0.151 | 0.087 | 1.456 |
| Erectile_dysfunction_b | 0.827 | 0.065 | -2.41 | 0.016 | 0.709 | 0.965 |
| Any_tumour_b | 1.155 | 0.075 | 2.21 | 0.027 | 1.017 | 1.312 |
| Menopause_b | 0.751 | 0.076 | -2.82 | 0.005 | 0.616 | 0.916 |
| Dementia_b | 1.781 | 0.181 | 5.69 | 0.000 | 1.460 | 2.173 |
| RA_b | 1.535 | 0.178 | 3.70 | 0.000 | 1.223 | 1.927 |
| num_ami_before_31_dec_14 | 0.999 | 0.003 | -0.48 | 0.631 | 0.993 | 1.004 |
| _cons | 0.003 | 0.001 | -18.32 | 0.000 | 0.002 | 0.006 |

**Performance in validation dataset (20% of practices) - Internal validation - audit AMI cases**

| AUROC (95% CI): 0.688 (0.661; 0.716) |  |
| --- | --- |
|  |  |
|  | % Correctly classified: 75.29 |

### 5) 1-year CV mortality

|  | | | | | | |
| --- | --- | --- | --- | --- | --- | --- |
| _1year_CV_death | Odds ratio | Std. err. | z | P>|z| | [95% conf. interval] | |
| age | 1.070 | 0.004 | 17.69 | 0.000 | 1.062 | 1.078 |
|  |  |  |  |  |  |  |
| gender |  |  |  |  |  |  |
| Female | 0.919 | 0.069 | -1.12 | 0.261 | 0.792 | 1.065 |
|  |  |  |  |  |  |  |
| IMD |  |  |  |  |  |  |
| Q2 | 1.098 | 0.109 | 0.94 | 0.345 | 0.904 | 1.334 |
| Q3 | 1.367 | 0.132 | 3.25 | 0.001 | 1.132 | 1.652 |
| Q4 | 1.079 | 0.110 | 0.75 | 0.454 | 0.884 | 1.318 |
| Q5-most deprived | 1.332 | 0.134 | 2.85 | 0.004 | 1.094 | 1.621 |
|  |  |  |  |  |  |  |
| ethnicity2 | 0.925 | 0.120 | -0.60 | 0.549 | 0.718 | 1.193 |
| Alcohol_status3 | 0.914 | 0.073 | -1.12 | 0.261 | 0.782 | 1.069 |
|  |  |  |  |  |  |  |
| Smoking_status |  |  |  |  |  |  |
| current smoker | 1.339 | 0.130 | 3.00 | 0.003 | 1.107 | 1.621 |
| ex-smoker | 1.121 | 0.095 | 1.35 | 0.177 | 0.950 | 1.324 |
|  |  |  |  |  |  |  |
| BMI_2014_f | 0.975 | 0.007 | -3.68 | 0.000 | 0.962 | 0.988 |
| HT_b | 1.042 | 0.066 | 0.65 | 0.515 | 0.920 | 1.181 |
| Hyperlipidaemia_b | 1.149 | 0.075 | 2.13 | 0.033 | 1.011 | 1.305 |
|  |  |  |  |  |  |  |
| Diabetes_type_au_b |  |  |  |  |  |  |
| T1DM | 3.763 | 1.185 | 4.21 | 0.000 | 2.030 | 6.975 |
| T2DM | 1.998 | 0.140 | 9.88 | 0.000 | 1.742 | 2.292 |
| DM - nos | 3.408 | 0.610 | 6.85 | 0.000 | 2.399 | 4.841 |
|  |  |  |  |  |  |  |
| HF_b | 2.669 | 0.174 | 15.05 | 0.000 | 2.348 | 3.033 |
| AF_b | 1.534 | 0.104 | 6.30 | 0.000 | 1.343 | 1.752 |
| Heart_valve_dis_b | 1.546 | 0.262 | 2.57 | 0.010 | 1.109 | 2.156 |
| VT_VF_b | 2.363 | 0.406 | 5.00 | 0.000 | 1.686 | 3.310 |
| Cardiomyopathy_b | 2.147 | 0.386 | 4.25 | 0.000 | 1.510 | 3.054 |
| CV_procedures_b | 0.748 | 0.050 | -4.36 | 0.000 | 0.657 | 0.852 |
| TIA_stroke_b | 1.311 | 0.096 | 3.69 | 0.000 | 1.136 | 1.514 |
| PVD_b | 1.336 | 0.124 | 3.13 | 0.002 | 1.115 | 1.602 |
| CKD_b | 1.348 | 0.089 | 4.52 | 0.000 | 1.184 | 1.534 |
| Hypothyroidism_b | 0.905 | 0.085 | -1.06 | 0.290 | 0.753 | 1.088 |
| Liver_dis_b | 2.120 | 1.140 | 1.40 | 0.162 | 0.739 | 6.082 |
| Lupus_b | 0.272 | 0.275 | -1.29 | 0.199 | 0.037 | 1.981 |
| Erectile_dysfunction_b | 0.927 | 0.089 | -0.79 | 0.427 | 0.768 | 1.118 |
| Any_tumour_b | 1.030 | 0.073 | 0.41 | 0.679 | 0.896 | 1.183 |
| Menopause_b | 0.923 | 0.107 | -0.69 | 0.488 | 0.736 | 1.158 |
| Dementia_b | 1.494 | 0.155 | 3.87 | 0.000 | 1.219 | 1.832 |
| RA_b | 1.201 | 0.176 | 1.25 | 0.213 | 0.901 | 1.600 |
| num_ami_before_31_dec_14 | 0.994 | 0.004 | -1.66 | 0.098 | 0.986 | 1.001 |
| _cons | 0.000 | 0.000 | -22.06 | 0.000 | 0.000 | 0.000 |

**Performance in validation dataset (20% of practices) - Internal validation - audit AMI cases**

| AUROC (95% CI): 0.825 (0.802; 0.849) |  |
| --- | --- |
|  |  |
|  | % Correctly classified: 85.38 |

### 6) 1-year Composite 1 outcome (HF, stroke, recurrent MI, & all-cause mortality)

|  | | | | | | |
| --- | --- | --- | --- | --- | --- | --- |
| _1yr_Comp1_outcome | Odds ratio | Std. err. | z | P>|z| | [95% conf. interval] | |
| age | 1.070 | 0.002 | 47.67 | 0.000 | 1.067 | 1.073 |
|  |  |  |  |  |  |  |
| gender |  |  |  |  |  |  |
| Female | 1.034 | 0.033 | 1.04 | 0.300 | 0.971 | 1.102 |
|  |  |  |  |  |  |  |
| IMD |  |  |  |  |  |  |
| Q2 | 1.149 | 0.046 | 3.50 | 0.000 | 1.063 | 1.242 |
| Q3 | 1.282 | 0.051 | 6.21 | 0.000 | 1.186 | 1.387 |
| Q4 | 1.317 | 0.053 | 6.83 | 0.000 | 1.217 | 1.425 |
| Q5-most deprived | 1.469 | 0.060 | 9.40 | 0.000 | 1.355 | 1.591 |
|  |  |  |  |  |  |  |
| ethnicity2 | 0.982 | 0.050 | -0.36 | 0.719 | 0.889 | 1.085 |
| Alcohol_status3 | 0.784 | 0.027 | -7.03 | 0.000 | 0.732 | 0.839 |
|  |  |  |  |  |  |  |
| Smoking_status |  |  |  |  |  |  |
| current smoker | 1.598 | 0.063 | 11.92 | 0.000 | 1.480 | 1.726 |
| ex-smoker | 1.094 | 0.039 | 2.55 | 0.011 | 1.021 | 1.173 |
|  |  |  |  |  |  |  |
| BMI_2014_f | 0.984 | 0.003 | -6.08 | 0.000 | 0.979 | 0.989 |
| HT_b | 1.176 | 0.031 | 6.23 | 0.000 | 1.117 | 1.237 |
| Hyperlipidaemia_b | 1.026 | 0.028 | 0.93 | 0.354 | 0.972 | 1.083 |
|  |  |  |  |  |  |  |
| Diabetes_type_au_b |  |  |  |  |  |  |
| T1DM | 5.243 | 0.764 | 11.37 | 0.000 | 3.941 | 6.975 |
| T2DM | 1.861 | 0.058 | 19.95 | 0.000 | 1.751 | 1.978 |
| DM - nos | 3.353 | 0.346 | 11.73 | 0.000 | 2.740 | 4.105 |
|  |  |  |  |  |  |  |
| HF_b | 7.729 | 0.265 | 59.66 | 0.000 | 7.227 | 8.266 |
| AF_b | 2.055 | 0.069 | 21.56 | 0.000 | 1.925 | 2.195 |
| Heart_valve_dis_b | 1.824 | 0.189 | 5.80 | 0.000 | 1.489 | 2.235 |
| VT_VF_b | 1.931 | 0.195 | 6.52 | 0.000 | 1.584 | 2.354 |
| Cardiomyopathy_b | 1.858 | 0.199 | 5.80 | 0.000 | 1.507 | 2.292 |
| CV_procedures_b | 0.776 | 0.020 | -9.80 | 0.000 | 0.738 | 0.817 |
| TIA_stroke_b | 4.596 | 0.163 | 42.92 | 0.000 | 4.287 | 4.928 |
| PVD_b | 1.916 | 0.085 | 14.58 | 0.000 | 1.756 | 2.091 |
| CKD_b | 1.596 | 0.045 | 16.50 | 0.000 | 1.510 | 1.687 |
| Hypothyroidism_b | 1.061 | 0.044 | 1.42 | 0.154 | 0.978 | 1.150 |
| Liver_dis_b | 2.118 | 0.488 | 3.26 | 0.001 | 1.348 | 3.329 |
| Lupus_b | 1.730 | 0.357 | 2.66 | 0.008 | 1.155 | 2.592 |
| Erectile_dysfunction_b | 0.905 | 0.033 | -2.71 | 0.007 | 0.842 | 0.973 |
| Any_tumour_b | 1.472 | 0.045 | 12.52 | 0.000 | 1.385 | 1.563 |
| Menopause_b | 0.763 | 0.035 | -5.89 | 0.000 | 0.697 | 0.835 |
| Dementia_b | 3.642 | 0.238 | 19.75 | 0.000 | 3.204 | 4.141 |
| RA_b | 1.312 | 0.082 | 4.38 | 0.000 | 1.162 | 1.482 |
| num_ami_before_31_dec_14 | 1.001 | 0.001 | 0.62 | 0.534 | 0.998 | 1.003 |
| _cons | 0.001 | 0.000 | -43.87 | 0.000 | 0.001 | 0.002 |

**Performance in validation dataset (20% of practices) - Internal validation - audit AMI cases**

| AUROC (95% CI): 0.865 (0.858; 0.872) |  |
| --- | --- |
|  |  |
|  | % Correctly classified: 80.84 |

### 7) 1-year Composite 1 CV outcome (HF, stroke, recurrent MI, & CV mortality)

|  | | | | | | |
| --- | --- | --- | --- | --- | --- | --- |
| _1yr_Comp1_CV_outcome | Odds ratio | Std. err. | z | P>|z| | [95% conf. interval] | |
| age | 1.053 | 0.002 | 35.91 | 0.000 | 1.050 | 1.056 |
|  |  |  |  |  |  |  |
| gender |  |  |  |  |  |  |
| Female | 1.048 | 0.035 | 1.41 | 0.160 | 0.982 | 1.118 |
|  |  |  |  |  |  |  |
| IMD |  |  |  |  |  |  |
| Q2 | 1.186 | 0.049 | 4.13 | 0.000 | 1.094 | 1.285 |
| Q3 | 1.300 | 0.054 | 6.32 | 0.000 | 1.198 | 1.410 |
| Q4 | 1.309 | 0.055 | 6.45 | 0.000 | 1.206 | 1.420 |
| Q5-most deprived | 1.473 | 0.062 | 9.20 | 0.000 | 1.356 | 1.600 |
|  |  |  |  |  |  |  |
| ethnicity2 | 1.121 | 0.057 | 2.24 | 0.025 | 1.015 | 1.240 |
| Alcohol_status3 | 0.819 | 0.029 | -5.68 | 0.000 | 0.765 | 0.878 |
|  |  |  |  |  |  |  |
| Smoking_status |  |  |  |  |  |  |
| current smoker | 1.328 | 0.053 | 7.08 | 0.000 | 1.228 | 1.437 |
| ex-smoker | 1.015 | 0.037 | 0.40 | 0.686 | 0.945 | 1.089 |
|  |  |  |  |  |  |  |
| BMI_2014_f | 1.007 | 0.003 | 2.47 | 0.013 | 1.001 | 1.012 |
| HT_b | 1.208 | 0.032 | 7.08 | 0.000 | 1.147 | 1.273 |
| Hyperlipidaemia_b | 1.040 | 0.029 | 1.41 | 0.159 | 0.985 | 1.099 |
|  |  |  |  |  |  |  |
| Diabetes_type_au_b |  |  |  |  |  |  |
| T1DM | 3.954 | 0.578 | 9.40 | 0.000 | 2.969 | 5.266 |
| T2DM | 1.681 | 0.053 | 16.54 | 0.000 | 1.581 | 1.788 |
| DM - nos | 3.238 | 0.323 | 11.76 | 0.000 | 2.662 | 3.938 |
|  |  |  |  |  |  |  |
| HF_b | 8.394 | 0.270 | 66.04 | 0.000 | 7.881 | 8.942 |
| AF_b | 1.852 | 0.061 | 18.65 | 0.000 | 1.736 | 1.976 |
| Heart_valve_dis_b | 1.804 | 0.181 | 5.90 | 0.000 | 1.483 | 2.195 |
| VT_VF_b | 1.695 | 0.170 | 5.27 | 0.000 | 1.393 | 2.063 |
| Cardiomyopathy_b | 2.226 | 0.231 | 7.70 | 0.000 | 1.816 | 2.728 |
| CV_procedures_b | 0.853 | 0.023 | -5.95 | 0.000 | 0.810 | 0.899 |
| TIA_stroke_b | 4.351 | 0.147 | 43.38 | 0.000 | 4.071 | 4.650 |
| PVD_b | 1.736 | 0.077 | 12.43 | 0.000 | 1.591 | 1.893 |
| CKD_b | 1.397 | 0.041 | 11.52 | 0.000 | 1.320 | 1.479 |
| Hypothyroidism_b | 1.049 | 0.044 | 1.13 | 0.260 | 0.966 | 1.139 |
| Liver_dis_b | 1.918 | 0.449 | 2.78 | 0.005 | 1.213 | 3.034 |
| Lupus_b | 1.606 | 0.334 | 2.28 | 0.023 | 1.068 | 2.415 |
| Erectile_dysfunction_b | 0.962 | 0.036 | -1.03 | 0.303 | 0.893 | 1.036 |
| Any_tumour_b | 1.170 | 0.038 | 4.90 | 0.000 | 1.099 | 1.246 |
| Menopause_b | 0.837 | 0.040 | -3.77 | 0.000 | 0.763 | 0.918 |
| Dementia_b | 1.463 | 0.088 | 6.33 | 0.000 | 1.301 | 1.646 |
| RA_b | 1.333 | 0.084 | 4.57 | 0.000 | 1.178 | 1.509 |
| num_ami_before_31_dec_14 | 1.002 | 0.001 | 1.53 | 0.126 | 0.999 | 1.004 |
| _cons | 0.002 | 0.000 | -41.41 | 0.000 | 0.001 | 0.002 |

**Performance in validation dataset (20% of practices) - Internal validation - audit AMI cases**

| AUROC (95% CI): 0.843 (0.835; 0.851) |  |
| --- | --- |
|  |  |
|  | % Correctly classified: 80.85 |

### 8) 1-year Composite 2 outcome (HF, stroke, & all-cause mortality)

|  | | | | | | |
| --- | --- | --- | --- | --- | --- | --- |
| _1yr_Comp2_outcome | Odds ratio | Std. err. | z | P>|z| | [95% conf. interval] | |
| age | 1.078 | 0.002 | 49.50 | 0.000 | 1.075 | 1.081 |
|  |  |  |  |  |  |  |
| gender |  |  |  |  |  |  |
| Female | 1.015 | 0.034 | 0.46 | 0.647 | 0.951 | 1.085 |
|  |  |  |  |  |  |  |
| IMD |  |  |  |  |  |  |
| Q2 | 1.154 | 0.048 | 3.45 | 0.001 | 1.064 | 1.251 |
| Q3 | 1.314 | 0.055 | 6.54 | 0.000 | 1.211 | 1.426 |
| Q4 | 1.406 | 0.059 | 8.13 | 0.000 | 1.295 | 1.527 |
| Q5-most deprived | 1.578 | 0.067 | 10.71 | 0.000 | 1.452 | 1.716 |
|  |  |  |  |  |  |  |
| ethnicity2 | 0.894 | 0.048 | -2.09 | 0.037 | 0.805 | 0.993 |
| Alcohol_status3 | 0.712 | 0.025 | -9.54 | 0.000 | 0.664 | 0.764 |
|  |  |  |  |  |  |  |
| Smoking_status |  |  |  |  |  |  |
| current smoker | 1.495 | 0.061 | 9.83 | 0.000 | 1.380 | 1.620 |
| ex-smoker | 1.094 | 0.040 | 2.44 | 0.015 | 1.018 | 1.175 |
|  |  |  |  |  |  |  |
| BMI_2014_f | 0.986 | 0.003 | -4.90 | 0.000 | 0.981 | 0.992 |
| HT_b | 1.142 | 0.031 | 4.92 | 0.000 | 1.083 | 1.205 |
| Hyperlipidaemia_b | 0.989 | 0.028 | -0.40 | 0.686 | 0.935 | 1.046 |
|  |  |  |  |  |  |  |
| Diabetes_type_au_b |  |  |  |  |  |  |
| T1DM | 5.117 | 0.779 | 10.73 | 0.000 | 3.798 | 6.896 |
| T2DM | 1.847 | 0.060 | 19.01 | 0.000 | 1.734 | 1.968 |
| DM - nos | 3.429 | 0.362 | 11.67 | 0.000 | 2.788 | 4.217 |
|  |  |  |  |  |  |  |
| HF_b | 9.190 | 0.323 | 63.20 | 0.000 | 8.579 | 9.844 |
| AF_b | 2.102 | 0.072 | 21.80 | 0.000 | 1.967 | 2.248 |
| Heart_valve_dis_b | 1.727 | 0.182 | 5.19 | 0.000 | 1.405 | 2.122 |
| VT_VF_b | 1.715 | 0.179 | 5.16 | 0.000 | 1.398 | 2.106 |
| Cardiomyopathy_b | 1.964 | 0.213 | 6.22 | 0.000 | 1.588 | 2.430 |
| CV_procedures_b | 0.752 | 0.020 | -10.56 | 0.000 | 0.714 | 0.793 |
| TIA_stroke_b | 4.891 | 0.176 | 44.04 | 0.000 | 4.557 | 5.249 |
| PVD_b | 1.994 | 0.091 | 15.13 | 0.000 | 1.824 | 2.181 |
| CKD_b | 1.577 | 0.046 | 15.64 | 0.000 | 1.490 | 1.670 |
| Hypothyroidism_b | 1.031 | 0.044 | 0.71 | 0.480 | 0.948 | 1.120 |
| Liver_dis_b | 2.117 | 0.511 | 3.10 | 0.002 | 1.318 | 3.399 |
| Lupus_b | 1.659 | 0.358 | 2.35 | 0.019 | 1.087 | 2.531 |
| Erectile_dysfunction_b | 0.939 | 0.036 | -1.63 | 0.104 | 0.871 | 1.013 |
| Any_tumour_b | 1.451 | 0.046 | 11.72 | 0.000 | 1.364 | 1.545 |
| Menopause_b | 0.830 | 0.039 | -3.93 | 0.000 | 0.756 | 0.911 |
| Dementia_b | 3.713 | 0.243 | 20.08 | 0.000 | 3.267 | 4.220 |
| RA_b | 1.392 | 0.089 | 5.18 | 0.000 | 1.228 | 1.578 |
| num_ami_before_31_dec_14 | 0.998 | 0.001 | -1.73 | 0.084 | 0.995 | 1.000 |
| _cons | 0.001 | 0.000 | -45.51 | 0.000 | 0.000 | 0.001 |

**Performance in validation dataset (20% of practices) - Internal validation - audit AMI cases**

| AUROC (95% CI): 0.883 (0.876; 0.890) |  |
| --- | --- |
|  |  |
|  | % Correctly classified: 82.46 |

### 9) 1-year Composite 2 CV outcome (HF, stroke, & CV mortality)

|  | | | | | | |
| --- | --- | --- | --- | --- | --- | --- |
| _1yr_Comp2_CV_outcome | Odds ratio | Std. err. | z | P>|z| | [95% conf. interval] | |
| age | 1.058 | 0.002 | 36.52 | 0.000 | 1.054 | 1.061 |
|  |  |  |  |  |  |  |
| gender |  |  |  |  |  |  |
| Female | 1.056 | 0.037 | 1.56 | 0.119 | 0.986 | 1.130 |
|  |  |  |  |  |  |  |
| IMD |  |  |  |  |  |  |
| Q2 | 1.140 | 0.049 | 3.02 | 0.002 | 1.047 | 1.241 |
| Q3 | 1.243 | 0.054 | 5.00 | 0.000 | 1.142 | 1.354 |
| Q4 | 1.276 | 0.056 | 5.58 | 0.000 | 1.171 | 1.391 |
| Q5-most deprived | 1.385 | 0.061 | 7.36 | 0.000 | 1.270 | 1.510 |
|  |  |  |  |  |  |  |
| ethnicity2 | 1.079 | 0.059 | 1.39 | 0.164 | 0.970 | 1.200 |
| Alcohol_status3 | 0.795 | 0.029 | -6.28 | 0.000 | 0.740 | 0.854 |
|  |  |  |  |  |  |  |
| Smoking_status |  |  |  |  |  |  |
| current smoker | 1.388 | 0.059 | 7.73 | 0.000 | 1.277 | 1.509 |
| ex-smoker | 1.090 | 0.041 | 2.27 | 0.023 | 1.012 | 1.175 |
|  |  |  |  |  |  |  |
| BMI_2014_f | 1.005 | 0.003 | 1.65 | 0.099 | 0.999 | 1.010 |
| HT_b | 1.198 | 0.034 | 6.41 | 0.000 | 1.133 | 1.266 |
| Hyperlipidaemia_b | 1.063 | 0.031 | 2.09 | 0.036 | 1.004 | 1.127 |
|  |  |  |  |  |  |  |
| Diabetes_type_au_b |  |  |  |  |  |  |
| T1DM | 4.704 | 0.709 | 10.28 | 0.000 | 3.502 | 6.320 |
| T2DM | 1.685 | 0.056 | 15.84 | 0.000 | 1.580 | 1.798 |
| DM - nos | 2.803 | 0.292 | 9.89 | 0.000 | 2.285 | 3.439 |
|  |  |  |  |  |  |  |
| HF_b | 10.107 | 0.335 | 69.88 | 0.000 | 9.472 | 10.785 |
| AF_b | 1.966 | 0.067 | 19.87 | 0.000 | 1.839 | 2.101 |
| Heart_valve_dis_b | 1.675 | 0.172 | 5.02 | 0.000 | 1.369 | 2.048 |
| VT_VF_b | 1.840 | 0.190 | 5.91 | 0.000 | 1.503 | 2.253 |
| Cardiomyopathy_b | 2.261 | 0.239 | 7.70 | 0.000 | 1.837 | 2.782 |
| CV_procedures_b | 0.824 | 0.023 | -6.90 | 0.000 | 0.780 | 0.870 |
| TIA_stroke_b | 4.859 | 0.169 | 45.52 | 0.000 | 4.539 | 5.201 |
| PVD_b | 1.683 | 0.078 | 11.30 | 0.000 | 1.537 | 1.842 |
| CKD_b | 1.461 | 0.044 | 12.58 | 0.000 | 1.377 | 1.550 |
| Hypothyroidism_b | 1.041 | 0.046 | 0.91 | 0.364 | 0.955 | 1.134 |
| Liver_dis_b | 2.243 | 0.541 | 3.35 | 0.001 | 1.398 | 3.598 |
| Lupus_b | 1.256 | 0.286 | 1.00 | 0.316 | 0.804 | 1.961 |
| Erectile_dysfunction_b | 0.964 | 0.039 | -0.91 | 0.363 | 0.891 | 1.043 |
| Any_tumour_b | 1.171 | 0.039 | 4.73 | 0.000 | 1.097 | 1.250 |
| Menopause_b | 0.874 | 0.043 | -2.73 | 0.006 | 0.794 | 0.963 |
| Dementia_b | 1.287 | 0.080 | 4.07 | 0.000 | 1.140 | 1.454 |
| RA_b | 1.360 | 0.089 | 4.68 | 0.000 | 1.196 | 1.547 |
| num_ami_before_31_dec_14 | 1.000 | 0.001 | 0.30 | 0.767 | 0.998 | 1.003 |
| _cons | 0.001 | 0.000 | -41.75 | 0.000 | 0.001 | 0.001 |

**Performance in validation dataset (20% of practices) - Internal validation - audit AMI cases**

| AUROC (95% CI): 0.862 (0.854; 0.870) |  |
| --- | --- |
|  |  |
|  | % Correctly classified: 82.54 |

## External validation (CPRD GOLD) - without therapies

## 1) 1-year all-cause mortality

|  | | | | | | |
| --- | --- | --- | --- | --- | --- | --- |
| _1year_All_cause_death | Odds ratio | Std. err. | z | P>|z| | [95% conf. interval] | |
| age | 1.114 | 0.003 | 46.92 | 0.000 | 1.109 | 1.119 |
|  |  |  |  |  |  |  |
| gender |  |  |  |  |  |  |
| Female | 0.929 | 0.039 | -1.77 | 0.077 | 0.857 | 1.008 |
|  |  |  |  |  |  |  |
| IMD |  |  |  |  |  |  |
| Q2 | 1.109 | 0.061 | 1.89 | 0.058 | 0.996 | 1.234 |
| Q3 | 1.470 | 0.079 | 7.14 | 0.000 | 1.322 | 1.634 |
| Q4 | 1.404 | 0.077 | 6.17 | 0.000 | 1.260 | 1.564 |
| Q5-most deprived | 1.546 | 0.087 | 7.74 | 0.000 | 1.385 | 1.727 |
|  |  |  |  |  |  |  |
| ethnicity2 | 0.599 | 0.049 | -6.28 | 0.000 | 0.511 | 0.703 |
| Alcohol_status3 | 0.786 | 0.035 | -5.36 | 0.000 | 0.720 | 0.859 |
|  |  |  |  |  |  |  |
| Smoking_status |  |  |  |  |  |  |
| current smoker | 2.011 | 0.109 | 12.92 | 0.000 | 1.809 | 2.236 |
| ex-smoker | 1.171 | 0.056 | 3.31 | 0.001 | 1.067 | 1.285 |
|  |  |  |  |  |  |  |
| BMI_2014_f | 0.933 | 0.004 | -17.23 | 0.000 | 0.925 | 0.940 |
| HT_b | 1.027 | 0.036 | 0.74 | 0.460 | 0.958 | 1.100 |
| Hyperlipidaemia_b | 0.889 | 0.034 | -3.11 | 0.002 | 0.826 | 0.958 |
|  |  |  |  |  |  |  |
| Diabetes_type_au_b |  |  |  |  |  |  |
| T1DM | 4.562 | 0.959 | 7.22 | 0.000 | 3.022 | 6.886 |
| T2DM | 2.244 | 0.093 | 19.55 | 0.000 | 2.069 | 2.434 |
| DM - nos | 3.509 | 0.439 | 10.03 | 0.000 | 2.746 | 4.485 |
|  |  |  |  |  |  |  |
| HF_b | 2.233 | 0.089 | 20.18 | 0.000 | 2.065 | 2.414 |
| AF_b | 1.517 | 0.060 | 10.49 | 0.000 | 1.403 | 1.640 |
| Heart_valve_dis_b | 2.152 | 0.235 | 7.03 | 0.000 | 1.738 | 2.664 |
| VT_VF_b | 2.016 | 0.254 | 5.56 | 0.000 | 1.574 | 2.582 |
| Cardiomyopathy_b | 1.602 | 0.214 | 3.52 | 0.000 | 1.233 | 2.082 |
| CV_procedures_b | 0.679 | 0.025 | -10.52 | 0.000 | 0.632 | 0.730 |
| TIA_stroke_b | 1.485 | 0.063 | 9.35 | 0.000 | 1.367 | 1.613 |
| PVD_b | 1.737 | 0.093 | 10.37 | 0.000 | 1.565 | 1.929 |
| CKD_b | 1.522 | 0.055 | 11.63 | 0.000 | 1.418 | 1.634 |
| Hypothyroidism_b | 1.046 | 0.054 | 0.88 | 0.381 | 0.946 | 1.158 |
| Liver_dis_b | 4.311 | 1.281 | 4.92 | 0.000 | 2.407 | 7.719 |
| Lupus_b | 1.080 | 0.340 | 0.25 | 0.806 | 0.583 | 2.003 |
| Erectile_dysfunction_b | 0.813 | 0.045 | -3.71 | 0.000 | 0.729 | 0.907 |
| Any_tumour_b | 1.664 | 0.063 | 13.52 | 0.000 | 1.546 | 1.792 |
| Menopause_b | 0.666 | 0.043 | -6.23 | 0.000 | 0.586 | 0.757 |
| Dementia_b | 4.601 | 0.274 | 25.64 | 0.000 | 4.094 | 5.170 |
| RA_b | 1.849 | 0.143 | 7.96 | 0.000 | 1.589 | 2.151 |
| num_ami_before_31_dec_14 | 0.990 | 0.002 | -4.52 | 0.000 | 0.986 | 0.995 |
| _cons | 0.000 | 0.000 | -39.48 | 0.000 | 0.000 | 0.000 |

**Performance in validation dataset (CPRD GOLD) - External validation - audit AMI cases**

| AUROC (95% CI): 0.870 (0.858; 0.882) |  |
| --- | --- |
|  |  |
|  | % Correctly classified: 85.15 |

### 2) 1-year Stroke

|  | | | | | | |
| --- | --- | --- | --- | --- | --- | --- |
| _1year_Stroke | Odds ratio | Std. err. | z | P>|z| | [95% conf. interval] | |
| age | 1.036 | 0.002 | 15.34 | 0.000 | 1.031 | 1.041 |
|  |  |  |  |  |  |  |
| gender |  |  |  |  |  |  |
| Female | 1.065 | 0.054 | 1.24 | 0.214 | 0.964 | 1.177 |
|  |  |  |  |  |  |  |
| IMD |  |  |  |  |  |  |
| Q2 | 1.075 | 0.068 | 1.14 | 0.255 | 0.949 | 1.217 |
| Q3 | 1.055 | 0.068 | 0.84 | 0.404 | 0.930 | 1.198 |
| Q4 | 1.150 | 0.074 | 2.17 | 0.030 | 1.014 | 1.305 |
| Q5-most deprived | 1.189 | 0.078 | 2.66 | 0.008 | 1.046 | 1.351 |
|  |  |  |  |  |  |  |
| ethnicity2 | 0.889 | 0.074 | -1.42 | 0.154 | 0.756 | 1.045 |
| Alcohol_status3 | 0.760 | 0.040 | -5.28 | 0.000 | 0.686 | 0.842 |
|  |  |  |  |  |  |  |
| Smoking_status |  |  |  |  |  |  |
| current smoker | 1.093 | 0.069 | 1.41 | 0.158 | 0.966 | 1.237 |
| ex-smoker | 1.058 | 0.059 | 1.02 | 0.308 | 0.949 | 1.180 |
|  |  |  |  |  |  |  |
| BMI_2014_f | 0.983 | 0.004 | -3.91 | 0.000 | 0.975 | 0.992 |
| HT_b | 1.109 | 0.047 | 2.46 | 0.014 | 1.021 | 1.204 |
| Hyperlipidaemia_b | 1.068 | 0.046 | 1.53 | 0.126 | 0.982 | 1.162 |
|  |  |  |  |  |  |  |
| Diabetes_type_au_b |  |  |  |  |  |  |
| T1DM | 2.562 | 0.558 | 4.32 | 0.000 | 1.672 | 3.926 |
| T2DM | 1.322 | 0.065 | 5.71 | 0.000 | 1.201 | 1.456 |
| DM - nos | 2.691 | 0.356 | 7.48 | 0.000 | 2.076 | 3.488 |
|  |  |  |  |  |  |  |
| HF_b | 1.122 | 0.057 | 2.25 | 0.024 | 1.015 | 1.241 |
| AF_b | 1.377 | 0.066 | 6.69 | 0.000 | 1.254 | 1.513 |
| Heart_valve_dis_b | 1.177 | 0.160 | 1.20 | 0.229 | 0.902 | 1.536 |
| VT_VF_b | 1.092 | 0.181 | 0.53 | 0.597 | 0.788 | 1.512 |
| Cardiomyopathy_b | 0.841 | 0.153 | -0.95 | 0.341 | 0.588 | 1.201 |
| CV_procedures_b | 0.876 | 0.037 | -3.13 | 0.002 | 0.806 | 0.952 |
| TIA_stroke_b | 11.579 | 0.474 | 59.86 | 0.000 | 10.687 | 12.546 |
| PVD_b | 1.202 | 0.077 | 2.86 | 0.004 | 1.059 | 1.363 |
| CKD_b | 1.143 | 0.051 | 3.00 | 0.003 | 1.047 | 1.247 |
| Hypothyroidism_b | 1.076 | 0.067 | 1.19 | 0.235 | 0.953 | 1.215 |
| Liver_dis_b | 1.209 | 0.497 | 0.46 | 0.644 | 0.540 | 2.707 |
| Lupus_b | 1.398 | 0.416 | 1.12 | 0.261 | 0.780 | 2.506 |
| Erectile_dysfunction_b | 1.066 | 0.065 | 1.06 | 0.289 | 0.947 | 1.201 |
| Any_tumour_b | 1.117 | 0.054 | 2.29 | 0.022 | 1.016 | 1.227 |
| Menopause_b | 1.009 | 0.071 | 0.13 | 0.895 | 0.879 | 1.159 |
| Dementia_b | 1.915 | 0.141 | 8.84 | 0.000 | 1.658 | 2.212 |
| RA_b | 0.970 | 0.098 | -0.31 | 0.759 | 0.795 | 1.182 |
| num_ami_before_31_dec_14 | 1.001 | 0.002 | 0.44 | 0.663 | 0.997 | 1.005 |
| _cons | 0.003 | 0.001 | -23.23 | 0.000 | 0.002 | 0.005 |

**Performance in validation dataset (CPRD GOLD) - External validation - audit AMI cases**

| AUROC (95% CI): 0.804 (0.782; 0.826) |  |
| --- | --- |
|  |  |
|  | % Correctly classified: 88.29 |

### 3) 1-year heart failure (HF)

|  | | | | | | |
| --- | --- | --- | --- | --- | --- | --- |
| _1year_HF | Odds ratio | Std. err. | z | P>|z| | [95% conf. interval] | |
| age | 1.054 | 0.002 | 28.92 | 0.000 | 1.050 | 1.057 |
|  |  |  |  |  |  |  |
| gender |  |  |  |  |  |  |
| Female | 1.023 | 0.041 | 0.57 | 0.571 | 0.945 | 1.107 |
|  |  |  |  |  |  |  |
| IMD |  |  |  |  |  |  |
| Q2 | 1.162 | 0.060 | 2.92 | 0.003 | 1.051 | 1.284 |
| Q3 | 1.374 | 0.070 | 6.23 | 0.000 | 1.243 | 1.518 |
| Q4 | 1.260 | 0.065 | 4.49 | 0.000 | 1.139 | 1.394 |
| Q5-most deprived | 1.425 | 0.074 | 6.81 | 0.000 | 1.287 | 1.579 |
|  |  |  |  |  |  |  |
| ethnicity2 | 1.115 | 0.070 | 1.72 | 0.085 | 0.985 | 1.261 |
| Alcohol_status3 | 0.784 | 0.033 | -5.79 | 0.000 | 0.722 | 0.851 |
|  |  |  |  |  |  |  |
| Smoking_status |  |  |  |  |  |  |
| current smoker | 1.294 | 0.064 | 5.21 | 0.000 | 1.174 | 1.425 |
| ex-smoker | 1.062 | 0.047 | 1.38 | 0.169 | 0.975 | 1.158 |
|  |  |  |  |  |  |  |
| BMI_2014_f | 1.014 | 0.003 | 4.24 | 0.000 | 1.007 | 1.020 |
| HT_b | 1.125 | 0.037 | 3.58 | 0.000 | 1.055 | 1.200 |
| Hyperlipidaemia_b | 1.064 | 0.036 | 1.80 | 0.071 | 0.995 | 1.137 |
|  |  |  |  |  |  |  |
| Diabetes_type_au_b |  |  |  |  |  |  |
| T1DM | 3.524 | 0.627 | 7.08 | 0.000 | 2.487 | 4.995 |
| T2DM | 1.679 | 0.063 | 13.73 | 0.000 | 1.559 | 1.808 |
| DM - nos | 2.155 | 0.254 | 6.52 | 0.000 | 1.711 | 2.715 |
|  |  |  |  |  |  |  |
| HF_b | 14.724 | 0.494 | 80.20 | 0.000 | 13.787 | 15.724 |
| AF_b | 2.165 | 0.081 | 20.65 | 0.000 | 2.012 | 2.330 |
| Heart_valve_dis_b | 2.148 | 0.232 | 7.09 | 0.000 | 1.738 | 2.653 |
| VT_VF_b | 2.052 | 0.226 | 6.54 | 0.000 | 1.654 | 2.545 |
| Cardiomyopathy_b | 2.272 | 0.245 | 7.60 | 0.000 | 1.839 | 2.808 |
| CV_procedures_b | 0.886 | 0.029 | -3.68 | 0.000 | 0.831 | 0.945 |
| TIA_stroke_b | 1.374 | 0.058 | 7.57 | 0.000 | 1.265 | 1.491 |
| PVD_b | 1.646 | 0.085 | 9.64 | 0.000 | 1.487 | 1.821 |
| CKD_b | 1.518 | 0.052 | 12.06 | 0.000 | 1.418 | 1.624 |
| Hypothyroidism_b | 1.017 | 0.051 | 0.33 | 0.743 | 0.921 | 1.122 |
| Liver_dis_b | 1.913 | 0.531 | 2.34 | 0.019 | 1.110 | 3.295 |
| Lupus_b | 1.228 | 0.318 | 0.80 | 0.426 | 0.740 | 2.039 |
| Erectile_dysfunction_b | 0.966 | 0.045 | -0.75 | 0.456 | 0.881 | 1.059 |
| Any_tumour_b | 1.214 | 0.047 | 5.04 | 0.000 | 1.126 | 1.309 |
| Menopause_b | 0.816 | 0.048 | -3.46 | 0.001 | 0.727 | 0.915 |
| Dementia_b | 1.144 | 0.080 | 1.91 | 0.056 | 0.997 | 1.312 |
| RA_b | 1.348 | 0.102 | 3.94 | 0.000 | 1.162 | 1.564 |
| num_ami_before_31_dec_14 | 1.001 | 0.002 | 0.44 | 0.660 | 0.998 | 1.004 |
| _cons | 0.001 | 0.000 | -38.51 | 0.000 | 0.000 | 0.001 |

**Performance in validation dataset (CPRD GOLD) - External validation - audit AMI cases**

| AUROC (95% CI): 0.718 (0.699; 0.741) |  |
| --- | --- |
|  |  |
|  | % Correctly classified: 78.91 |

### 4) 1-year Recurrent MI

|  | | | | | | |
| --- | --- | --- | --- | --- | --- | --- |
| _1year_Recurrent_MI | Odds ratio | Std. err. | z | P>|z| | [95% conf. interval] | |
| age | 1.026 | 0.003 | 8.82 | 0.000 | 1.020 | 1.032 |
|  |  |  |  |  |  |  |
| gender |  |  |  |  |  |  |
| Female | 1.026 | 0.068 | 0.38 | 0.702 | 0.900 | 1.169 |
|  |  |  |  |  |  |  |
| IMD |  |  |  |  |  |  |
| Q2 | 1.009 | 0.088 | 0.10 | 0.921 | 0.851 | 1.196 |
| Q3 | 1.180 | 0.100 | 1.95 | 0.051 | 0.999 | 1.393 |
| Q4 | 1.086 | 0.094 | 0.95 | 0.340 | 0.917 | 1.286 |
| Q5-most deprived | 1.234 | 0.105 | 2.46 | 0.014 | 1.044 | 1.460 |
|  |  |  |  |  |  |  |
| ethnicity2 | 1.153 | 0.111 | 1.49 | 0.137 | 0.956 | 1.391 |
| Alcohol_status3 | 0.836 | 0.057 | -2.62 | 0.009 | 0.732 | 0.956 |
|  |  |  |  |  |  |  |
| Smoking_status |  |  |  |  |  |  |
| current smoker | 1.109 | 0.088 | 1.31 | 0.191 | 0.949 | 1.296 |
| ex-smoker | 0.847 | 0.062 | -2.28 | 0.022 | 0.734 | 0.977 |
|  |  |  |  |  |  |  |
| BMI_2014_f | 0.999 | 0.005 | -0.12 | 0.902 | 0.989 | 1.010 |
| HT_b | 1.151 | 0.064 | 2.55 | 0.011 | 1.033 | 1.283 |
| Hyperlipidaemia_b | 1.122 | 0.063 | 2.05 | 0.040 | 1.005 | 1.253 |
|  |  |  |  |  |  |  |
| Diabetes_type_au_b |  |  |  |  |  |  |
| T1DM | 4.484 | 0.948 | 7.09 | 0.000 | 2.962 | 6.787 |
| T2DM | 1.749 | 0.108 | 9.07 | 0.000 | 1.550 | 1.973 |
| DM - nos | 3.104 | 0.478 | 7.36 | 0.000 | 2.296 | 4.197 |
|  |  |  |  |  |  |  |
| HF_b | 1.278 | 0.086 | 3.66 | 0.000 | 1.121 | 1.458 |
| AF_b | 0.888 | 0.065 | -1.62 | 0.105 | 0.770 | 1.025 |
| Heart_valve_dis_b | 0.953 | 0.204 | -0.22 | 0.822 | 0.627 | 1.449 |
| VT_VF_b | 1.096 | 0.233 | 0.43 | 0.668 | 0.722 | 1.663 |
| Cardiomyopathy_b | 1.420 | 0.289 | 1.72 | 0.086 | 0.952 | 2.117 |
| CV_procedures_b | 0.971 | 0.053 | -0.54 | 0.586 | 0.872 | 1.080 |
| TIA_stroke_b | 1.088 | 0.079 | 1.16 | 0.244 | 0.944 | 1.254 |
| PVD_b | 1.760 | 0.137 | 7.24 | 0.000 | 1.510 | 2.051 |
| CKD_b | 1.238 | 0.075 | 3.54 | 0.000 | 1.100 | 1.393 |
| Hypothyroidism_b | 1.018 | 0.086 | 0.21 | 0.834 | 0.862 | 1.201 |
| Liver_dis_b | 2.411 | 0.860 | 2.47 | 0.014 | 1.198 | 4.849 |
| Lupus_b | 0.357 | 0.256 | -1.44 | 0.151 | 0.087 | 1.456 |
| Erectile_dysfunction_b | 0.827 | 0.065 | -2.41 | 0.016 | 0.709 | 0.965 |
| Any_tumour_b | 1.155 | 0.075 | 2.21 | 0.027 | 1.017 | 1.312 |
| Menopause_b | 0.751 | 0.076 | -2.82 | 0.005 | 0.616 | 0.916 |
| Dementia_b | 1.781 | 0.181 | 5.69 | 0.000 | 1.460 | 2.173 |
| RA_b | 1.535 | 0.178 | 3.70 | 0.000 | 1.223 | 1.927 |
| num_ami_before_31_dec_14 | 0.999 | 0.003 | -0.48 | 0.631 | 0.993 | 1.004 |
| _cons | 0.003 | 0.001 | -18.32 | 0.000 | 0.002 | 0.006 |

**Performance in validation dataset (CPRD GOLD) - External validation - audit AMI cases**

| AUROC (95% CI): 0.671 (0.637; 0.705) |  |
| --- | --- |
|  |  |
|  | % Correctly classified: 73.85 |

### 5) 1-year CV mortality

|  | | | | | | |
| --- | --- | --- | --- | --- | --- | --- |
| _1year_CV_death | Odds ratio | Std. err. | z | P>|z| | [95% conf. interval] | |
| age | 1.070 | 0.004 | 17.69 | 0.000 | 1.062 | 1.078 |
|  |  |  |  |  |  |  |
| gender |  |  |  |  |  |  |
| Female | 0.919 | 0.069 | -1.12 | 0.261 | 0.792 | 1.065 |
|  |  |  |  |  |  |  |
| IMD |  |  |  |  |  |  |
| Q2 | 1.098 | 0.109 | 0.94 | 0.345 | 0.904 | 1.334 |
| Q3 | 1.367 | 0.132 | 3.25 | 0.001 | 1.132 | 1.652 |
| Q4 | 1.079 | 0.110 | 0.75 | 0.454 | 0.884 | 1.318 |
| Q5-most deprived | 1.332 | 0.134 | 2.85 | 0.004 | 1.094 | 1.621 |
|  |  |  |  |  |  |  |
| ethnicity2 | 0.925 | 0.120 | -0.60 | 0.549 | 0.718 | 1.193 |
| Alcohol_status3 | 0.914 | 0.073 | -1.12 | 0.261 | 0.782 | 1.069 |
|  |  |  |  |  |  |  |
| Smoking_status |  |  |  |  |  |  |
| current smoker | 1.339 | 0.130 | 3.00 | 0.003 | 1.107 | 1.621 |
| ex-smoker | 1.121 | 0.095 | 1.35 | 0.177 | 0.950 | 1.324 |
|  |  |  |  |  |  |  |
| BMI_2014_f | 0.975 | 0.007 | -3.68 | 0.000 | 0.962 | 0.988 |
| HT_b | 1.042 | 0.066 | 0.65 | 0.515 | 0.920 | 1.181 |
| Hyperlipidaemia_b | 1.149 | 0.075 | 2.13 | 0.033 | 1.011 | 1.305 |
|  |  |  |  |  |  |  |
| Diabetes_type_au_b |  |  |  |  |  |  |
| T1DM | 3.763 | 1.185 | 4.21 | 0.000 | 2.030 | 6.975 |
| T2DM | 1.998 | 0.140 | 9.88 | 0.000 | 1.742 | 2.292 |
| DM - nos | 3.408 | 0.610 | 6.85 | 0.000 | 2.399 | 4.841 |
|  |  |  |  |  |  |  |
| HF_b | 2.669 | 0.174 | 15.05 | 0.000 | 2.348 | 3.033 |
| AF_b | 1.534 | 0.104 | 6.30 | 0.000 | 1.343 | 1.752 |
| Heart_valve_dis_b | 1.546 | 0.262 | 2.57 | 0.010 | 1.109 | 2.156 |
| VT_VF_b | 2.363 | 0.406 | 5.00 | 0.000 | 1.686 | 3.310 |
| Cardiomyopathy_b | 2.147 | 0.386 | 4.25 | 0.000 | 1.510 | 3.054 |
| CV_procedures_b | 0.748 | 0.050 | -4.36 | 0.000 | 0.657 | 0.852 |
| TIA_stroke_b | 1.311 | 0.096 | 3.69 | 0.000 | 1.136 | 1.514 |
| PVD_b | 1.336 | 0.124 | 3.13 | 0.002 | 1.115 | 1.602 |
| CKD_b | 1.348 | 0.089 | 4.52 | 0.000 | 1.184 | 1.534 |
| Hypothyroidism_b | 0.905 | 0.085 | -1.06 | 0.290 | 0.753 | 1.088 |
| Liver_dis_b | 2.120 | 1.140 | 1.40 | 0.162 | 0.739 | 6.082 |
| Lupus_b | 0.272 | 0.275 | -1.29 | 0.199 | 0.037 | 1.981 |
| Erectile_dysfunction_b | 0.927 | 0.089 | -0.79 | 0.427 | 0.768 | 1.118 |
| Any_tumour_b | 1.030 | 0.073 | 0.41 | 0.679 | 0.896 | 1.183 |
| Menopause_b | 0.923 | 0.107 | -0.69 | 0.488 | 0.736 | 1.158 |
| Dementia_b | 1.494 | 0.155 | 3.87 | 0.000 | 1.219 | 1.832 |
| RA_b | 1.201 | 0.176 | 1.25 | 0.213 | 0.901 | 1.600 |
| num_ami_before_31_dec_14 | 0.994 | 0.004 | -1.66 | 0.098 | 0.986 | 1.001 |
| _cons | 0.000 | 0.000 | -22.06 | 0.000 | 0.000 | 0.000 |

**Performance in validation dataset (CPRD GOLD) - External validation - audit AMI cases**

| AUROC (95% CI): 0.817 (0.787; 0.847) |  |
| --- | --- |
|  |  |
|  | % Correctly classified: 84.28 |

### 6) 1-year Composite 1 outcome (HF, stroke, recurrent MI, & all-cause mortality)

|  | | | | | | |
| --- | --- | --- | --- | --- | --- | --- |
| _1yr_Comp1_outcome | Odds ratio | Std. err. | z | P>|z| | [95% conf. interval] | |
| age | 1.070 | 0.002 | 47.67 | 0.000 | 1.067 | 1.073 |
|  |  |  |  |  |  |  |
| gender |  |  |  |  |  |  |
| Female | 1.034 | 0.033 | 1.04 | 0.300 | 0.971 | 1.102 |
|  |  |  |  |  |  |  |
| IMD |  |  |  |  |  |  |
| Q2 | 1.149 | 0.046 | 3.50 | 0.000 | 1.063 | 1.242 |
| Q3 | 1.282 | 0.051 | 6.21 | 0.000 | 1.186 | 1.387 |
| Q4 | 1.317 | 0.053 | 6.83 | 0.000 | 1.217 | 1.425 |
| Q5-most deprived | 1.469 | 0.060 | 9.40 | 0.000 | 1.355 | 1.591 |
|  |  |  |  |  |  |  |
| ethnicity2 | 0.982 | 0.050 | -0.36 | 0.719 | 0.889 | 1.085 |
| Alcohol_status3 | 0.784 | 0.027 | -7.03 | 0.000 | 0.732 | 0.839 |
|  |  |  |  |  |  |  |
| Smoking_status |  |  |  |  |  |  |
| current smoker | 1.598 | 0.063 | 11.92 | 0.000 | 1.480 | 1.726 |
| ex-smoker | 1.094 | 0.039 | 2.55 | 0.011 | 1.021 | 1.173 |
|  |  |  |  |  |  |  |
| BMI_2014_f | 0.984 | 0.003 | -6.08 | 0.000 | 0.979 | 0.989 |
| HT_b | 1.176 | 0.031 | 6.23 | 0.000 | 1.117 | 1.237 |
| Hyperlipidaemia_b | 1.026 | 0.028 | 0.93 | 0.354 | 0.972 | 1.083 |
|  |  |  |  |  |  |  |
| Diabetes_type_au_b |  |  |  |  |  |  |
| T1DM | 5.243 | 0.764 | 11.37 | 0.000 | 3.941 | 6.975 |
| T2DM | 1.861 | 0.058 | 19.95 | 0.000 | 1.751 | 1.978 |
| DM - nos | 3.353 | 0.346 | 11.73 | 0.000 | 2.740 | 4.105 |
|  |  |  |  |  |  |  |
| HF_b | 7.729 | 0.265 | 59.66 | 0.000 | 7.227 | 8.266 |
| AF_b | 2.055 | 0.069 | 21.56 | 0.000 | 1.925 | 2.195 |
| Heart_valve_dis_b | 1.824 | 0.189 | 5.80 | 0.000 | 1.489 | 2.235 |
| VT_VF_b | 1.931 | 0.195 | 6.52 | 0.000 | 1.584 | 2.354 |
| Cardiomyopathy_b | 1.858 | 0.199 | 5.80 | 0.000 | 1.507 | 2.292 |
| CV_procedures_b | 0.776 | 0.020 | -9.80 | 0.000 | 0.738 | 0.817 |
| TIA_stroke_b | 4.596 | 0.163 | 42.92 | 0.000 | 4.287 | 4.928 |
| PVD_b | 1.916 | 0.085 | 14.58 | 0.000 | 1.756 | 2.091 |
| CKD_b | 1.596 | 0.045 | 16.50 | 0.000 | 1.510 | 1.687 |
| Hypothyroidism_b | 1.061 | 0.044 | 1.42 | 0.154 | 0.978 | 1.150 |
| Liver_dis_b | 2.118 | 0.488 | 3.26 | 0.001 | 1.348 | 3.329 |
| Lupus_b | 1.730 | 0.357 | 2.66 | 0.008 | 1.155 | 2.592 |
| Erectile_dysfunction_b | 0.905 | 0.033 | -2.71 | 0.007 | 0.842 | 0.973 |
| Any_tumour_b | 1.472 | 0.045 | 12.52 | 0.000 | 1.385 | 1.563 |
| Menopause_b | 0.763 | 0.035 | -5.89 | 0.000 | 0.697 | 0.835 |
| Dementia_b | 3.642 | 0.238 | 19.75 | 0.000 | 3.204 | 4.141 |
| RA_b | 1.312 | 0.082 | 4.38 | 0.000 | 1.162 | 1.482 |
| num_ami_before_31_dec_14 | 1.001 | 0.001 | 0.62 | 0.534 | 0.998 | 1.003 |
| _cons | 0.001 | 0.000 | -43.87 | 0.000 | 0.001 | 0.002 |

**Performance in validation dataset (CPRD GOLD) - External validation - audit AMI cases**

| AUROC (95% CI): 0.813 (0.801; 0.825) |  |
| --- | --- |
|  |  |
|  | % Correctly classified: 76.67 |

### 7) 1-year Composite 1 CV outcome (HF, stroke, recurrent MI, & CV mortality)

|  | | | | | | |
| --- | --- | --- | --- | --- | --- | --- |
| _1yr_Comp1_CV_outcome | Odds ratio | Std. err. | z | P>|z| | [95% conf. interval] | |
| age | 1.053 | 0.002 | 35.91 | 0.000 | 1.050 | 1.056 |
|  |  |  |  |  |  |  |
| gender |  |  |  |  |  |  |
| Female | 1.048 | 0.035 | 1.41 | 0.160 | 0.982 | 1.118 |
|  |  |  |  |  |  |  |
| IMD |  |  |  |  |  |  |
| Q2 | 1.186 | 0.049 | 4.13 | 0.000 | 1.094 | 1.285 |
| Q3 | 1.300 | 0.054 | 6.32 | 0.000 | 1.198 | 1.410 |
| Q4 | 1.309 | 0.055 | 6.45 | 0.000 | 1.206 | 1.420 |
| Q5-most deprived | 1.473 | 0.062 | 9.20 | 0.000 | 1.356 | 1.600 |
|  |  |  |  |  |  |  |
| ethnicity2 | 1.121 | 0.057 | 2.24 | 0.025 | 1.015 | 1.240 |
| Alcohol_status3 | 0.819 | 0.029 | -5.68 | 0.000 | 0.765 | 0.878 |
|  |  |  |  |  |  |  |
| Smoking_status |  |  |  |  |  |  |
| current smoker | 1.328 | 0.053 | 7.08 | 0.000 | 1.228 | 1.437 |
| ex-smoker | 1.015 | 0.037 | 0.40 | 0.686 | 0.945 | 1.089 |
|  |  |  |  |  |  |  |
| BMI_2014_f | 1.007 | 0.003 | 2.47 | 0.013 | 1.001 | 1.012 |
| HT_b | 1.208 | 0.032 | 7.08 | 0.000 | 1.147 | 1.273 |
| Hyperlipidaemia_b | 1.040 | 0.029 | 1.41 | 0.159 | 0.985 | 1.099 |
|  |  |  |  |  |  |  |
| Diabetes_type_au_b |  |  |  |  |  |  |
| T1DM | 3.954 | 0.578 | 9.40 | 0.000 | 2.969 | 5.266 |
| T2DM | 1.681 | 0.053 | 16.54 | 0.000 | 1.581 | 1.788 |
| DM - nos | 3.238 | 0.323 | 11.76 | 0.000 | 2.662 | 3.938 |
|  |  |  |  |  |  |  |
| HF_b | 8.394 | 0.270 | 66.04 | 0.000 | 7.881 | 8.942 |
| AF_b | 1.852 | 0.061 | 18.65 | 0.000 | 1.736 | 1.976 |
| Heart_valve_dis_b | 1.804 | 0.181 | 5.90 | 0.000 | 1.483 | 2.195 |
| VT_VF_b | 1.695 | 0.170 | 5.27 | 0.000 | 1.393 | 2.063 |
| Cardiomyopathy_b | 2.226 | 0.231 | 7.70 | 0.000 | 1.816 | 2.728 |
| CV_procedures_b | 0.853 | 0.023 | -5.95 | 0.000 | 0.810 | 0.899 |
| TIA_stroke_b | 4.351 | 0.147 | 43.38 | 0.000 | 4.071 | 4.650 |
| PVD_b | 1.736 | 0.077 | 12.43 | 0.000 | 1.591 | 1.893 |
| CKD_b | 1.397 | 0.041 | 11.52 | 0.000 | 1.320 | 1.479 |
| Hypothyroidism_b | 1.049 | 0.044 | 1.13 | 0.260 | 0.966 | 1.139 |
| Liver_dis_b | 1.918 | 0.449 | 2.78 | 0.005 | 1.213 | 3.034 |
| Lupus_b | 1.606 | 0.334 | 2.28 | 0.023 | 1.068 | 2.415 |
| Erectile_dysfunction_b | 0.962 | 0.036 | -1.03 | 0.303 | 0.893 | 1.036 |
| Any_tumour_b | 1.170 | 0.038 | 4.90 | 0.000 | 1.099 | 1.246 |
| Menopause_b | 0.837 | 0.040 | -3.77 | 0.000 | 0.763 | 0.918 |
| Dementia_b | 1.463 | 0.088 | 6.33 | 0.000 | 1.301 | 1.646 |
| RA_b | 1.333 | 0.084 | 4.57 | 0.000 | 1.178 | 1.509 |
| num_ami_before_31_dec_14 | 1.002 | 0.001 | 1.53 | 0.126 | 0.999 | 1.004 |
| _cons | 0.002 | 0.000 | -41.41 | 0.000 | 0.001 | 0.002 |

**Performance in validation dataset (CPRD GOLD) - External validation - audit AMI cases**

| AUROC (95% CI): 0.757 (0.743; 0.772) |  |
| --- | --- |
|  |  |
|  | % Correctly classified: 75.30 |

### 8) 1-year Composite 2 outcome (HF, stroke, & all-cause mortality)

|  | | | | | | |
| --- | --- | --- | --- | --- | --- | --- |
| _1yr_Comp2_outcome | Odds ratio | Std. err. | z | P>|z| | [95% conf. interval] | |
| age | 1.078 | 0.002 | 49.50 | 0.000 | 1.075 | 1.081 |
|  |  |  |  |  |  |  |
| gender |  |  |  |  |  |  |
| Female | 1.015 | 0.034 | 0.46 | 0.647 | 0.951 | 1.085 |
|  |  |  |  |  |  |  |
| IMD |  |  |  |  |  |  |
| Q2 | 1.154 | 0.048 | 3.45 | 0.001 | 1.064 | 1.251 |
| Q3 | 1.314 | 0.055 | 6.54 | 0.000 | 1.211 | 1.426 |
| Q4 | 1.406 | 0.059 | 8.13 | 0.000 | 1.295 | 1.527 |
| Q5-most deprived | 1.578 | 0.067 | 10.71 | 0.000 | 1.452 | 1.716 |
|  |  |  |  |  |  |  |
| ethnicity2 | 0.894 | 0.048 | -2.09 | 0.037 | 0.805 | 0.993 |
| Alcohol_status3 | 0.712 | 0.025 | -9.54 | 0.000 | 0.664 | 0.764 |
|  |  |  |  |  |  |  |
| Smoking_status |  |  |  |  |  |  |
| current smoker | 1.495 | 0.061 | 9.83 | 0.000 | 1.380 | 1.620 |
| ex-smoker | 1.094 | 0.040 | 2.44 | 0.015 | 1.018 | 1.175 |
|  |  |  |  |  |  |  |
| BMI_2014_f | 0.986 | 0.003 | -4.90 | 0.000 | 0.981 | 0.992 |
| HT_b | 1.142 | 0.031 | 4.92 | 0.000 | 1.083 | 1.205 |
| Hyperlipidaemia_b | 0.989 | 0.028 | -0.40 | 0.686 | 0.935 | 1.046 |
|  |  |  |  |  |  |  |
| Diabetes_type_au_b |  |  |  |  |  |  |
| T1DM | 5.117 | 0.779 | 10.73 | 0.000 | 3.798 | 6.896 |
| T2DM | 1.847 | 0.060 | 19.01 | 0.000 | 1.734 | 1.968 |
| DM - nos | 3.429 | 0.362 | 11.67 | 0.000 | 2.788 | 4.217 |
|  |  |  |  |  |  |  |
| HF_b | 9.190 | 0.323 | 63.20 | 0.000 | 8.579 | 9.844 |
| AF_b | 2.102 | 0.072 | 21.80 | 0.000 | 1.967 | 2.248 |
| Heart_valve_dis_b | 1.727 | 0.182 | 5.19 | 0.000 | 1.405 | 2.122 |
| VT_VF_b | 1.715 | 0.179 | 5.16 | 0.000 | 1.398 | 2.106 |
| Cardiomyopathy_b | 1.964 | 0.213 | 6.22 | 0.000 | 1.588 | 2.430 |
| CV_procedures_b | 0.752 | 0.020 | -10.56 | 0.000 | 0.714 | 0.793 |
| TIA_stroke_b | 4.891 | 0.176 | 44.04 | 0.000 | 4.557 | 5.249 |
| PVD_b | 1.994 | 0.091 | 15.13 | 0.000 | 1.824 | 2.181 |
| CKD_b | 1.577 | 0.046 | 15.64 | 0.000 | 1.490 | 1.670 |
| Hypothyroidism_b | 1.031 | 0.044 | 0.71 | 0.480 | 0.948 | 1.120 |
| Liver_dis_b | 2.117 | 0.511 | 3.10 | 0.002 | 1.318 | 3.399 |
| Lupus_b | 1.659 | 0.358 | 2.35 | 0.019 | 1.087 | 2.531 |
| Erectile_dysfunction_b | 0.939 | 0.036 | -1.63 | 0.104 | 0.871 | 1.013 |
| Any_tumour_b | 1.451 | 0.046 | 11.72 | 0.000 | 1.364 | 1.545 |
| Menopause_b | 0.830 | 0.039 | -3.93 | 0.000 | 0.756 | 0.911 |
| Dementia_b | 3.713 | 0.243 | 20.08 | 0.000 | 3.267 | 4.220 |
| RA_b | 1.392 | 0.089 | 5.18 | 0.000 | 1.228 | 1.578 |
| num_ami_before_31_dec_14 | 0.998 | 0.001 | -1.73 | 0.084 | 0.995 | 1.000 |
| _cons | 0.001 | 0.000 | -45.51 | 0.000 | 0.000 | 0.001 |

**Performance in validation dataset (CPRD GOLD) - External validation - audit AMI cases**

| AUROC (95% CI): 0.828 (0.816; 0.840) |  |
| --- | --- |
|  |  |
|  | % Correctly classified: 78.09 |

### 9) 1-year Composite 2 CV outcome (HF, stroke, & CV mortality)

|  | | | | | | |
| --- | --- | --- | --- | --- | --- | --- |
| _1yr_Comp2_CV_outcome | Odds ratio | Std. err. | z | P>|z| | [95% conf. interval] | |
| age | 1.058 | 0.002 | 36.52 | 0.000 | 1.054 | 1.061 |
|  |  |  |  |  |  |  |
| gender |  |  |  |  |  |  |
| Female | 1.056 | 0.037 | 1.56 | 0.119 | 0.986 | 1.130 |
|  |  |  |  |  |  |  |
| IMD |  |  |  |  |  |  |
| Q2 | 1.140 | 0.049 | 3.02 | 0.002 | 1.047 | 1.241 |
| Q3 | 1.243 | 0.054 | 5.00 | 0.000 | 1.142 | 1.354 |
| Q4 | 1.276 | 0.056 | 5.58 | 0.000 | 1.171 | 1.391 |
| Q5-most deprived | 1.385 | 0.061 | 7.36 | 0.000 | 1.270 | 1.510 |
|  |  |  |  |  |  |  |
| ethnicity2 | 1.079 | 0.059 | 1.39 | 0.164 | 0.970 | 1.200 |
| Alcohol_status3 | 0.795 | 0.029 | -6.28 | 0.000 | 0.740 | 0.854 |
|  |  |  |  |  |  |  |
| Smoking_status |  |  |  |  |  |  |
| current smoker | 1.388 | 0.059 | 7.73 | 0.000 | 1.277 | 1.509 |
| ex-smoker | 1.090 | 0.041 | 2.27 | 0.023 | 1.012 | 1.175 |
|  |  |  |  |  |  |  |
| BMI_2014_f | 1.005 | 0.003 | 1.65 | 0.099 | 0.999 | 1.010 |
| HT_b | 1.198 | 0.034 | 6.41 | 0.000 | 1.133 | 1.266 |
| Hyperlipidaemia_b | 1.063 | 0.031 | 2.09 | 0.036 | 1.004 | 1.127 |
|  |  |  |  |  |  |  |
| Diabetes_type_au_b |  |  |  |  |  |  |
| T1DM | 4.704 | 0.709 | 10.28 | 0.000 | 3.502 | 6.320 |
| T2DM | 1.685 | 0.056 | 15.84 | 0.000 | 1.580 | 1.798 |
| DM - nos | 2.803 | 0.292 | 9.89 | 0.000 | 2.285 | 3.439 |
|  |  |  |  |  |  |  |
| HF_b | 10.107 | 0.335 | 69.88 | 0.000 | 9.472 | 10.785 |
| AF_b | 1.966 | 0.067 | 19.87 | 0.000 | 1.839 | 2.101 |
| Heart_valve_dis_b | 1.675 | 0.172 | 5.02 | 0.000 | 1.369 | 2.048 |
| VT_VF_b | 1.840 | 0.190 | 5.91 | 0.000 | 1.503 | 2.253 |
| Cardiomyopathy_b | 2.261 | 0.239 | 7.70 | 0.000 | 1.837 | 2.782 |
| CV_procedures_b | 0.824 | 0.023 | -6.90 | 0.000 | 0.780 | 0.870 |
| TIA_stroke_b | 4.859 | 0.169 | 45.52 | 0.000 | 4.539 | 5.201 |
| PVD_b | 1.683 | 0.078 | 11.30 | 0.000 | 1.537 | 1.842 |
| CKD_b | 1.461 | 0.044 | 12.58 | 0.000 | 1.377 | 1.550 |
| Hypothyroidism_b | 1.041 | 0.046 | 0.91 | 0.364 | 0.955 | 1.134 |
| Liver_dis_b | 2.243 | 0.541 | 3.35 | 0.001 | 1.398 | 3.598 |
| Lupus_b | 1.256 | 0.286 | 1.00 | 0.316 | 0.804 | 1.961 |
| Erectile_dysfunction_b | 0.964 | 0.039 | -0.91 | 0.363 | 0.891 | 1.043 |
| Any_tumour_b | 1.171 | 0.039 | 4.73 | 0.000 | 1.097 | 1.250 |
| Menopause_b | 0.874 | 0.043 | -2.73 | 0.006 | 0.794 | 0.963 |
| Dementia_b | 1.287 | 0.080 | 4.07 | 0.000 | 1.140 | 1.454 |
| RA_b | 1.360 | 0.089 | 4.68 | 0.000 | 1.196 | 1.547 |
| num_ami_before_31_dec_14 | 1.000 | 0.001 | 0.30 | 0.767 | 0.998 | 1.003 |
| _cons | 0.001 | 0.000 | -41.75 | 0.000 | 0.001 | 0.001 |

**Performance in validation dataset (CPRD GOLD) - External validation - audit AMI cases**

| AUROC (95% CI): 0.764 (0.748; 0.779) |  |
| --- | --- |
|  |  |
|  | % Correctly classified: 76.40 |

## Internal validation (CPRD Aurum) - with therapies

### 1) 1-year all-cause mortality

|  | | | | | | |
| --- | --- | --- | --- | --- | --- | --- |
| _1year_All_cause_death | Odds ratio | Std. err. | z | P>|z| | [95% conf. interval] | |
| age | 1.102 | 0.003 | 42.08 | 0.000 | 1.097 | 1.107 |
|  |  |  |  |  |  |  |
| gender |  |  |  |  |  |  |
| Female | 0.875 | 0.037 | -3.21 | 0.001 | 0.806 | 0.949 |
|  |  |  |  |  |  |  |
| IMD |  |  |  |  |  |  |
| Q2 | 1.021 | 0.056 | 0.37 | 0.710 | 0.916 | 1.137 |
| Q3 | 1.345 | 0.073 | 5.47 | 0.000 | 1.210 | 1.496 |
| Q4 | 1.451 | 0.079 | 6.82 | 0.000 | 1.304 | 1.615 |
| Q5-most deprived | 1.528 | 0.086 | 7.57 | 0.000 | 1.369 | 1.706 |
|  |  |  |  |  |  |  |
| ethnicity2 |  |  |  |  |  |  |
| non-White | 0.719 | 0.055 | -4.28 | 0.000 | 0.618 | 0.836 |
|  |  |  |  |  |  |  |
| Alcohol_status3 |  |  |  |  |  |  |
| drinker | 0.753 | 0.033 | -6.38 | 0.000 | 0.690 | 0.822 |
|  |  |  |  |  |  |  |
| Smoking_status |  |  |  |  |  |  |
| current smoker | 1.822 | 0.099 | 11.02 | 0.000 | 1.638 | 2.028 |
| ex-smoker | 1.240 | 0.059 | 4.53 | 0.000 | 1.130 | 1.361 |
|  |  |  |  |  |  |  |
| BMI_2014_f | 0.931 | 0.004 | -17.79 | 0.000 | 0.923 | 0.938 |
| HT_b | 0.839 | 0.031 | -4.83 | 0.000 | 0.781 | 0.901 |
| Antihypertensives_b | 0.930 | 0.185 | -0.37 | 0.714 | 0.630 | 1.373 |
| Hyperlipidaemia_b | 0.963 | 0.036 | -1.01 | 0.313 | 0.894 | 1.036 |
| Lipid_reg_treatment_b | 0.381 | 0.034 | -10.69 | 0.000 | 0.320 | 0.455 |
|  |  |  |  |  |  |  |
| Diabetes_type_au_b |  |  |  |  |  |  |
| T1DM | 2.181 | 0.487 | 3.49 | 0.000 | 1.408 | 3.379 |
| T2DM | 1.338 | 0.098 | 3.97 | 0.000 | 1.159 | 1.545 |
| DM - nos | 2.010 | 0.272 | 5.15 | 0.000 | 1.541 | 2.621 |
|  |  |  |  |  |  |  |
| Antidiabetics_b | 1.835 | 0.142 | 7.85 | 0.000 | 1.577 | 2.135 |
| Diuretics_b | 2.123 | 0.101 | 15.80 | 0.000 | 1.934 | 2.331 |
| Anticoagulants_b | 1.053 | 0.052 | 1.03 | 0.304 | 0.955 | 1.161 |
| Antiplatelets_b | 0.760 | 0.082 | -2.56 | 0.011 | 0.616 | 0.938 |
| HF_b | 1.926 | 0.078 | 16.25 | 0.000 | 1.780 | 2.085 |
| AF_b | 1.388 | 0.064 | 7.09 | 0.000 | 1.268 | 1.520 |
| Heart_valve_dis_b | 1.869 | 0.204 | 5.72 | 0.000 | 1.508 | 2.315 |
| VT_VF_b | 1.882 | 0.239 | 4.98 | 0.000 | 1.467 | 2.415 |
| Cardiomyopathy_b | 1.132 | 0.156 | 0.90 | 0.371 | 0.863 | 1.484 |
| CV_procedures_b | 0.659 | 0.024 | -11.25 | 0.000 | 0.613 | 0.709 |
| TIA_stroke_b | 1.434 | 0.061 | 8.49 | 0.000 | 1.319 | 1.558 |
| PVD_b | 1.492 | 0.081 | 7.40 | 0.000 | 1.342 | 1.659 |
| CKD_b | 1.537 | 0.056 | 11.88 | 0.000 | 1.432 | 1.651 |
| Hypothyroidism_b | 1.028 | 0.053 | 0.54 | 0.590 | 0.929 | 1.138 |
| Liver_dis_b | 2.498 | 0.800 | 2.86 | 0.004 | 1.333 | 4.681 |
| Lupus_b | 1.441 | 0.429 | 1.23 | 0.220 | 0.804 | 2.584 |
| Erectile_dysfunction_b | 0.813 | 0.045 | -3.72 | 0.000 | 0.729 | 0.907 |
| Any_tumour_b | 1.786 | 0.067 | 15.44 | 0.000 | 1.659 | 1.923 |
| Menopause_b | 0.613 | 0.040 | -7.43 | 0.000 | 0.539 | 0.698 |
| Dementia_b | 4.350 | 0.261 | 24.48 | 0.000 | 3.867 | 4.893 |
| RA_b | 1.847 | 0.142 | 8.00 | 0.000 | 1.589 | 2.147 |
| num_ami_before_31_dec_14 | 0.992 | 0.002 | -3.97 | 0.000 | 0.988 | 0.996 |
| _cons | 0.000 | 0.000 | -25.22 | 0.000 | 0.000 | 0.001 |

**Performance in validation dataset (20% of practices) - Internal validation - audit AMI cases**

| AUROC (95% CI): 0.896 (0.887; 0.904) |  |
| --- | --- |
|  |  |
|  | % Correctly classified: 86.28 |

### 2) 1-year Stroke

|  | | | | | | |
| --- | --- | --- | --- | --- | --- | --- |
| _1year_Stroke | Odds ratio | Std. err. | z | P>|z| | [95% conf. interval] | |
| age | 1.031 | 0.002 | 12.82 | 0.000 | 1.026 | 1.035 |
|  |  |  |  |  |  |  |
| gender |  |  |  |  |  |  |
| Female | 1.076 | 0.055 | 1.43 | 0.153 | 0.973 | 1.189 |
|  |  |  |  |  |  |  |
| IMD |  |  |  |  |  |  |
| Q2 | 1.020 | 0.065 | 0.31 | 0.755 | 0.901 | 1.155 |
| Q3 | 1.017 | 0.065 | 0.26 | 0.792 | 0.897 | 1.154 |
| Q4 | 1.045 | 0.068 | 0.68 | 0.496 | 0.920 | 1.187 |
| Q5-most deprived | 1.162 | 0.075 | 2.33 | 0.020 | 1.024 | 1.319 |
|  |  |  |  |  |  |  |
| ethnicity2 |  |  |  |  |  |  |
| non-White | 0.947 | 0.077 | -0.67 | 0.504 | 0.807 | 1.111 |
|  |  |  |  |  |  |  |
| Alcohol_status3 |  |  |  |  |  |  |
| drinker | 0.820 | 0.043 | -3.78 | 0.000 | 0.740 | 0.909 |
|  |  |  |  |  |  |  |
| Smoking_status |  |  |  |  |  |  |
| current smoker | 1.141 | 0.071 | 2.11 | 0.035 | 1.010 | 1.289 |
| ex-smoker | 0.981 | 0.054 | -0.35 | 0.724 | 0.880 | 1.093 |
|  |  |  |  |  |  |  |
| BMI_2014_f | 0.976 | 0.004 | -5.51 | 0.000 | 0.968 | 0.985 |
| HT_b | 1.052 | 0.045 | 1.17 | 0.242 | 0.966 | 1.145 |
| Antihypertensives_b | 0.625 | 0.137 | -2.14 | 0.032 | 0.406 | 0.961 |
| Hyperlipidaemia_b | 1.078 | 0.046 | 1.75 | 0.080 | 0.991 | 1.173 |
| Lipid_reg_treatment_b | 0.843 | 0.106 | -1.36 | 0.174 | 0.659 | 1.078 |
|  |  |  |  |  |  |  |
| Diabetes_type_au_b |  |  |  |  |  |  |
| T1DM | 0.966 | 0.246 | -0.14 | 0.891 | 0.586 | 1.591 |
| T2DM | 0.772 | 0.074 | -2.69 | 0.007 | 0.639 | 0.932 |
| DM - nos | 1.455 | 0.227 | 2.40 | 0.016 | 1.071 | 1.977 |
|  |  |  |  |  |  |  |
| Antidiabetics_b | 1.921 | 0.191 | 6.56 | 0.000 | 1.581 | 2.335 |
| Diuretics_b | 1.592 | 0.084 | 8.81 | 0.000 | 1.435 | 1.765 |
| Anticoagulants_b | 1.120 | 0.065 | 1.96 | 0.051 | 1.000 | 1.256 |
| Antiplatelets_b | 0.913 | 0.125 | -0.66 | 0.508 | 0.698 | 1.195 |
| HF_b | 0.994 | 0.052 | -0.11 | 0.911 | 0.898 | 1.101 |
| AF_b | 1.392 | 0.077 | 5.96 | 0.000 | 1.248 | 1.551 |
| Heart_valve_dis_b | 1.048 | 0.145 | 0.34 | 0.732 | 0.800 | 1.374 |
| VT_VF_b | 1.167 | 0.190 | 0.95 | 0.342 | 0.848 | 1.606 |
| Cardiomyopathy_b | 0.704 | 0.131 | -1.88 | 0.060 | 0.489 | 1.015 |
| CV_procedures_b | 0.869 | 0.037 | -3.30 | 0.001 | 0.800 | 0.945 |
| TIA_stroke_b | 11.152 | 0.457 | 58.79 | 0.000 | 10.290 | 12.085 |
| PVD_b | 1.188 | 0.076 | 2.69 | 0.007 | 1.048 | 1.347 |
| CKD_b | 1.136 | 0.051 | 2.86 | 0.004 | 1.041 | 1.240 |
| Hypothyroidism_b | 0.978 | 0.062 | -0.36 | 0.720 | 0.864 | 1.106 |
| Liver_dis_b | 1.820 | 0.648 | 1.68 | 0.093 | 0.905 | 3.659 |
| Lupus_b | 0.816 | 0.275 | -0.60 | 0.546 | 0.421 | 1.580 |
| Erectile_dysfunction_b | 1.043 | 0.064 | 0.69 | 0.490 | 0.926 | 1.175 |
| Any_tumour_b | 1.126 | 0.054 | 2.47 | 0.013 | 1.025 | 1.237 |
| Menopause_b | 0.954 | 0.068 | -0.66 | 0.509 | 0.830 | 1.097 |
| Dementia_b | 1.781 | 0.132 | 7.78 | 0.000 | 1.540 | 2.060 |
| RA_b | 0.885 | 0.091 | -1.19 | 0.235 | 0.723 | 1.083 |
| num_ami_before_31_dec_14 | 1.003 | 0.002 | 1.71 | 0.088 | 1.000 | 1.007 |
| _cons | 0.009 | 0.003 | -14.34 | 0.000 | 0.005 | 0.017 |

**Performance in validation dataset (20% of practices) - Internal validation - audit AMI cases**

| AUROC (95% CI): 0.847 (0.834; 0.861) |  |
| --- | --- |
|  |  |
|  | % Correctly classified: 89.00 |

### 3) 1-year heart failure (HF)

|  | | | | | | |
| --- | --- | --- | --- | --- | --- | --- |
| _1year_HF | Odds ratio | Std. err. | z | P>|z| | [95% conf. interval] | |
| age | 1.039 | 0.002 | 20.82 | 0.000 | 1.035 | 1.042 |
|  |  |  |  |  |  |  |
| gender |  |  |  |  |  |  |
| Female | 0.957 | 0.039 | -1.09 | 0.278 | 0.884 | 1.036 |
|  |  |  |  |  |  |  |
| IMD |  |  |  |  |  |  |
| Q2 | 1.200 | 0.062 | 3.54 | 0.000 | 1.085 | 1.327 |
| Q3 | 1.249 | 0.065 | 4.30 | 0.000 | 1.129 | 1.382 |
| Q4 | 1.328 | 0.068 | 5.50 | 0.000 | 1.200 | 1.469 |
| Q5-most deprived | 1.451 | 0.076 | 7.14 | 0.000 | 1.310 | 1.607 |
|  |  |  |  |  |  |  |
| ethnicity2 |  |  |  |  |  |  |
| non-White | 1.133 | 0.071 | 1.99 | 0.047 | 1.002 | 1.283 |
|  |  |  |  |  |  |  |
| Alcohol_status3 |  |  |  |  |  |  |
| drinker | 0.804 | 0.034 | -5.17 | 0.000 | 0.740 | 0.873 |
|  |  |  |  |  |  |  |
| Smoking_status |  |  |  |  |  |  |
| current smoker | 1.276 | 0.063 | 4.91 | 0.000 | 1.158 | 1.407 |
| ex-smoker | 1.048 | 0.046 | 1.06 | 0.288 | 0.961 | 1.143 |
|  |  |  |  |  |  |  |
| BMI_2014_f | 1.001 | 0.003 | 0.36 | 0.717 | 0.995 | 1.008 |
| HT_b | 0.945 | 0.032 | -1.69 | 0.091 | 0.884 | 1.009 |
| Antihypertensives_b | 0.505 | 0.096 | -3.58 | 0.000 | 0.348 | 0.734 |
| Hyperlipidaemia_b | 1.038 | 0.036 | 1.08 | 0.281 | 0.970 | 1.110 |
| Lipid_reg_treatment_b | 0.647 | 0.061 | -4.60 | 0.000 | 0.537 | 0.779 |
|  |  |  |  |  |  |  |
| Diabetes_type_au_b |  |  |  |  |  |  |
| T1DM | 2.959 | 0.555 | 5.78 | 0.000 | 2.049 | 4.274 |
| T2DM | 1.409 | 0.094 | 5.11 | 0.000 | 1.235 | 1.606 |
| DM - nos | 1.526 | 0.197 | 3.27 | 0.001 | 1.184 | 1.966 |
|  |  |  |  |  |  |  |
| Antidiabetics_b | 1.115 | 0.079 | 1.54 | 0.124 | 0.971 | 1.280 |
| Diuretics_b | 3.381 | 0.156 | 26.48 | 0.000 | 3.090 | 3.700 |
| Anticoagulants_b | 1.412 | 0.064 | 7.61 | 0.000 | 1.292 | 1.544 |
| Antiplatelets_b | 0.592 | 0.060 | -5.14 | 0.000 | 0.485 | 0.723 |
| HF_b | 12.226 | 0.417 | 73.48 | 0.000 | 11.436 | 13.070 |
| AF_b | 1.602 | 0.070 | 10.77 | 0.000 | 1.471 | 1.746 |
| Heart_valve_dis_b | 1.873 | 0.203 | 5.80 | 0.000 | 1.515 | 2.315 |
| VT_VF_b | 1.791 | 0.200 | 5.21 | 0.000 | 1.438 | 2.229 |
| Cardiomyopathy_b | 1.886 | 0.205 | 5.85 | 0.000 | 1.524 | 2.333 |
| CV_procedures_b | 0.886 | 0.030 | -3.62 | 0.000 | 0.830 | 0.946 |
| TIA_stroke_b | 1.359 | 0.057 | 7.32 | 0.000 | 1.252 | 1.475 |
| PVD_b | 1.642 | 0.085 | 9.60 | 0.000 | 1.484 | 1.817 |
| CKD_b | 1.409 | 0.049 | 9.88 | 0.000 | 1.317 | 1.509 |
| Hypothyroidism_b | 1.073 | 0.053 | 1.41 | 0.157 | 0.973 | 1.183 |
| Liver_dis_b | 1.398 | 0.412 | 1.14 | 0.255 | 0.785 | 2.492 |
| Lupus_b | 0.951 | 0.255 | -0.19 | 0.851 | 0.563 | 1.607 |
| Erectile_dysfunction_b | 0.980 | 0.047 | -0.43 | 0.666 | 0.893 | 1.075 |
| Any_tumour_b | 1.191 | 0.046 | 4.51 | 0.000 | 1.104 | 1.284 |
| Menopause_b | 0.839 | 0.049 | -3.02 | 0.002 | 0.749 | 0.940 |
| Dementia_b | 1.222 | 0.085 | 2.87 | 0.004 | 1.066 | 1.401 |
| RA_b | 1.155 | 0.089 | 1.87 | 0.062 | 0.993 | 1.344 |
| num_ami_before_31_dec_14 | 1.003 | 0.002 | 2.27 | 0.023 | 1.000 | 1.006 |
| _cons | 0.006 | 0.001 | -19.87 | 0.000 | 0.003 | 0.009 |

**Performance in validation dataset (20% of practices) - Internal validation - audit AMI cases**

| AUROC (95% CI): 0.883 (0.876; 0.891) |  |
| --- | --- |
|  |  |
|  | % Correctly classified: 86.62 |

### 4) 1-year Recurrent MI

|  | | | | | | |
| --- | --- | --- | --- | --- | --- | --- |
| _1year_Recurrent_MI | Odds ratio | Std. err. | z | P>|z| | [95% conf. interval] | |
| age | 1.024 | 0.003 | 8.10 | 0.000 | 1.018 | 1.030 |
|  |  |  |  |  |  |  |
| gender |  |  |  |  |  |  |
| Female | 0.959 | 0.064 | -0.62 | 0.533 | 0.841 | 1.094 |
|  |  |  |  |  |  |  |
| IMD |  |  |  |  |  |  |
| Q2 | 1.052 | 0.091 | 0.59 | 0.556 | 0.889 | 1.245 |
| Q3 | 1.220 | 0.103 | 2.36 | 0.018 | 1.034 | 1.438 |
| Q4 | 1.061 | 0.092 | 0.68 | 0.496 | 0.895 | 1.257 |
| Q5-most deprived | 1.238 | 0.106 | 2.50 | 0.012 | 1.047 | 1.464 |
|  |  |  |  |  |  |  |
| ethnicity2 |  |  |  |  |  |  |
| non-White | 1.064 | 0.103 | 0.64 | 0.520 | 0.880 | 1.287 |
|  |  |  |  |  |  |  |
| Alcohol_status3 |  |  |  |  |  |  |
| drinker | 0.762 | 0.051 | -4.09 | 0.000 | 0.669 | 0.868 |
|  |  |  |  |  |  |  |
| Smoking_status |  |  |  |  |  |  |
| current smoker | 1.190 | 0.096 | 2.17 | 0.030 | 1.017 | 1.393 |
| ex-smoker | 0.926 | 0.068 | -1.05 | 0.293 | 0.801 | 1.069 |
|  |  |  |  |  |  |  |
| BMI_2014_f | 0.996 | 0.006 | -0.76 | 0.447 | 0.985 | 1.007 |
| HT_b | 1.029 | 0.058 | 0.51 | 0.611 | 0.921 | 1.150 |
| Antihypertensives_b | 0.415 | 0.090 | -4.08 | 0.000 | 0.271 | 0.633 |
| Hyperlipidaemia_b | 1.166 | 0.065 | 2.74 | 0.006 | 1.045 | 1.302 |
| Lipid_reg_treatment_b | 0.651 | 0.095 | -2.94 | 0.003 | 0.489 | 0.867 |
|  |  |  |  |  |  |  |
| Diabetes_type_au_b |  |  |  |  |  |  |
| T1DM | 2.344 | 0.576 | 3.47 | 0.001 | 1.449 | 3.793 |
| T2DM | 1.062 | 0.127 | 0.50 | 0.615 | 0.840 | 1.343 |
| DM - nos | 1.988 | 0.358 | 3.82 | 0.000 | 1.397 | 2.829 |
|  |  |  |  |  |  |  |
| Antidiabetics_b | 1.674 | 0.205 | 4.20 | 0.000 | 1.317 | 2.129 |
| Diuretics_b | 1.511 | 0.104 | 6.01 | 0.000 | 1.321 | 1.729 |
| Anticoagulants_b | 0.874 | 0.075 | -1.56 | 0.119 | 0.738 | 1.035 |
| Antiplatelets_b | 0.605 | 0.095 | -3.20 | 0.001 | 0.445 | 0.823 |
| HF_b | 1.297 | 0.086 | 3.93 | 0.000 | 1.139 | 1.478 |
| AF_b | 0.886 | 0.073 | -1.47 | 0.141 | 0.754 | 1.041 |
| Heart_valve_dis_b | 1.245 | 0.234 | 1.17 | 0.242 | 0.862 | 1.799 |
| VT_VF_b | 1.111 | 0.237 | 0.49 | 0.621 | 0.732 | 1.686 |
| Cardiomyopathy_b | 0.835 | 0.205 | -0.74 | 0.461 | 0.516 | 1.350 |
| CV_procedures_b | 0.949 | 0.052 | -0.95 | 0.342 | 0.853 | 1.057 |
| TIA_stroke_b | 1.125 | 0.080 | 1.65 | 0.098 | 0.978 | 1.293 |
| PVD_b | 1.678 | 0.131 | 6.63 | 0.000 | 1.440 | 1.955 |
| CKD_b | 1.299 | 0.078 | 4.39 | 0.000 | 1.156 | 1.461 |
| Hypothyroidism_b | 1.039 | 0.086 | 0.46 | 0.643 | 0.883 | 1.223 |
| Liver_dis_b | 1.746 | 0.701 | 1.39 | 0.165 | 0.795 | 3.834 |
| Lupus_b | 0.371 | 0.266 | -1.38 | 0.166 | 0.091 | 1.510 |
| Erectile_dysfunction_b | 0.918 | 0.071 | -1.10 | 0.270 | 0.790 | 1.068 |
| Any_tumour_b | 1.141 | 0.073 | 2.05 | 0.041 | 1.006 | 1.294 |
| Menopause_b | 0.777 | 0.078 | -2.51 | 0.012 | 0.639 | 0.946 |
| Dementia_b | 1.268 | 0.140 | 2.15 | 0.031 | 1.022 | 1.575 |
| RA_b | 1.339 | 0.162 | 2.42 | 0.015 | 1.058 | 1.697 |
| num_ami_before_31_dec_14 | 0.999 | 0.003 | -0.49 | 0.624 | 0.993 | 1.004 |
| _cons | 0.022 | 0.008 | -10.69 | 0.000 | 0.011 | 0.044 |

**Performance in validation dataset (20% of practices) - Internal validation - audit AMI cases**

| AUROC (95% CI): 0.704 (0.679; 0.729) |  |
| --- | --- |
|  |  |
|  | % Correctly classified: 74.87 |

### 5) 1-year CV mortality

|  | | | | | | |
| --- | --- | --- | --- | --- | --- | --- |
| _1year_CV_death | Odds ratio | Std. err. | z | P>|z| | [95% conf. interval] | |
| age | 1.069 | 0.004 | 16.97 | 0.000 | 1.061 | 1.078 |
|  |  |  |  |  |  |  |
| gender |  |  |  |  |  |  |
| Female | 0.857 | 0.064 | -2.07 | 0.038 | 0.741 | 0.992 |
|  |  |  |  |  |  |  |
| IMD |  |  |  |  |  |  |
| Q2 | 1.155 | 0.115 | 1.44 | 0.149 | 0.949 | 1.405 |
| Q3 | 1.434 | 0.139 | 3.72 | 0.000 | 1.186 | 1.734 |
| Q4 | 1.111 | 0.114 | 1.03 | 0.305 | 0.909 | 1.359 |
| Q5-most deprived | 1.416 | 0.143 | 3.45 | 0.001 | 1.162 | 1.725 |
|  |  |  |  |  |  |  |
| ethnicity2 |  |  |  |  |  |  |
| non-White | 0.783 | 0.105 | -1.82 | 0.068 | 0.602 | 1.019 |
|  |  |  |  |  |  |  |
| Alcohol_status3 |  |  |  |  |  |  |
| drinker | 0.966 | 0.077 | -0.44 | 0.663 | 0.826 | 1.129 |
|  |  |  |  |  |  |  |
| Smoking_status |  |  |  |  |  |  |
| current smoker | 1.514 | 0.150 | 4.19 | 0.000 | 1.247 | 1.838 |
| ex-smoker | 1.227 | 0.107 | 2.35 | 0.019 | 1.034 | 1.455 |
|  |  |  |  |  |  |  |
| BMI_2014_f | 0.966 | 0.007 | -5.02 | 0.000 | 0.953 | 0.979 |
| HT_b | 0.921 | 0.059 | -1.28 | 0.200 | 0.812 | 1.045 |
| Antihypertensives_b | 0.300 | 0.090 | -4.01 | 0.000 | 0.167 | 0.541 |
| Hyperlipidaemia_b | 1.114 | 0.073 | 1.65 | 0.098 | 0.980 | 1.266 |
| Lipid_reg_treatment_b | 1.029 | 0.168 | 0.18 | 0.860 | 0.748 | 1.417 |
|  |  |  |  |  |  |  |
| Diabetes_type_au_b |  |  |  |  |  |  |
| T1DM | 2.431 | 0.779 | 2.77 | 0.006 | 1.297 | 4.558 |
| T2DM | 1.289 | 0.166 | 1.97 | 0.049 | 1.001 | 1.660 |
| DM - nos | 1.978 | 0.418 | 3.23 | 0.001 | 1.308 | 2.993 |
|  |  |  |  |  |  |  |
| Antidiabetics_b | 1.885 | 0.250 | 4.78 | 0.000 | 1.454 | 2.444 |
| Diuretics_b | 2.594 | 0.263 | 9.42 | 0.000 | 2.127 | 3.164 |
| Anticoagulants_b | 1.006 | 0.086 | 0.07 | 0.945 | 0.851 | 1.189 |
| Antiplatelets_b | 0.616 | 0.104 | -2.88 | 0.004 | 0.443 | 0.856 |
| HF_b | 2.045 | 0.136 | 10.77 | 0.000 | 1.795 | 2.329 |
| AF_b | 1.340 | 0.106 | 3.71 | 0.000 | 1.148 | 1.565 |
| Heart_valve_dis_b | 1.263 | 0.228 | 1.29 | 0.196 | 0.887 | 1.799 |
| VT_VF_b | 2.503 | 0.427 | 5.38 | 0.000 | 1.792 | 3.497 |
| Cardiomyopathy_b | 2.072 | 0.377 | 4.01 | 0.000 | 1.451 | 2.959 |
| CV_procedures_b | 0.790 | 0.053 | -3.54 | 0.000 | 0.693 | 0.900 |
| TIA_stroke_b | 1.335 | 0.097 | 3.99 | 0.000 | 1.159 | 1.539 |
| PVD_b | 1.249 | 0.115 | 2.42 | 0.016 | 1.043 | 1.496 |
| CKD_b | 1.263 | 0.083 | 3.57 | 0.000 | 1.111 | 1.436 |
| Hypothyroidism_b | 0.893 | 0.084 | -1.20 | 0.229 | 0.743 | 1.074 |
| Liver_dis_b | 2.808 | 1.357 | 2.14 | 0.033 | 1.089 | 7.243 |
| Lupus_b | 0.259 | 0.262 | -1.33 | 0.183 | 0.035 | 1.889 |
| Erectile_dysfunction_b | 0.847 | 0.082 | -1.73 | 0.084 | 0.701 | 1.023 |
| Any_tumour_b | 0.954 | 0.068 | -0.66 | 0.510 | 0.830 | 1.097 |
| Menopause_b | 0.739 | 0.091 | -2.45 | 0.014 | 0.581 | 0.941 |
| Dementia_b | 1.760 | 0.172 | 5.78 | 0.000 | 1.453 | 2.132 |
| RA_b | 1.253 | 0.179 | 1.58 | 0.113 | 0.948 | 1.658 |
| num_ami_before_31_dec_14 | 0.994 | 0.004 | -1.42 | 0.154 | 0.987 | 1.002 |
| _cons | 0.000 | 0.000 | -15.41 | 0.000 | 0.000 | 0.001 |

**Performance in validation dataset (20% of practices) - Internal validation - audit AMI cases**

| AUROC (95% CI): 0.839 (0.817; 0.860) |  |
| --- | --- |
|  |  |
|  | % Correctly classified: 84.56 |

### 6) 1-year Composite 1 outcome (HF, stroke, recurrent MI, & all-cause mortality)

|  | | | | | | |
| --- | --- | --- | --- | --- | --- | --- |
| _1yr_Comp1_outcome | Odds ratio | Std. err. | z | P>|z| | [95% conf. interval] | |
| age | 1.063 | 0.002 | 42.01 | 0.000 | 1.060 | 1.066 |
|  |  |  |  |  |  |  |
| gender |  |  |  |  |  |  |
| Female | 0.911 | 0.030 | -2.84 | 0.005 | 0.853 | 0.971 |
|  |  |  |  |  |  |  |
| IMD |  |  |  |  |  |  |
| Q2 | 1.136 | 0.046 | 3.16 | 0.002 | 1.050 | 1.230 |
| Q3 | 1.343 | 0.055 | 7.27 | 0.000 | 1.241 | 1.455 |
| Q4 | 1.400 | 0.057 | 8.26 | 0.000 | 1.293 | 1.516 |
| Q5-most deprived | 1.459 | 0.061 | 9.10 | 0.000 | 1.345 | 1.583 |
|  |  |  |  |  |  |  |
| ethnicity2 |  |  |  |  |  |  |
| non-White | 0.917 | 0.048 | -1.66 | 0.096 | 0.829 | 1.015 |
|  |  |  |  |  |  |  |
| Alcohol_status3 |  |  |  |  |  |  |
| drinker | 0.821 | 0.029 | -5.63 | 0.000 | 0.767 | 0.879 |
|  |  |  |  |  |  |  |
| Smoking_status |  |  |  |  |  |  |
| current smoker | 1.584 | 0.063 | 11.52 | 0.000 | 1.465 | 1.713 |
| ex-smoker | 1.110 | 0.040 | 2.90 | 0.004 | 1.034 | 1.191 |
|  |  |  |  |  |  |  |
| BMI_2014_f | 0.975 | 0.003 | -9.14 | 0.000 | 0.970 | 0.981 |
| HT_b | 0.970 | 0.026 | -1.11 | 0.266 | 0.920 | 1.023 |
| Antihypertensives_b | 0.619 | 0.083 | -3.56 | 0.000 | 0.476 | 0.806 |
| Hyperlipidaemia_b | 1.000 | 0.028 | 0.00 | 0.997 | 0.947 | 1.056 |
| Lipid_reg_treatment_b | 0.334 | 0.029 | -12.67 | 0.000 | 0.282 | 0.396 |
|  |  |  |  |  |  |  |
| Diabetes_type_au_b |  |  |  |  |  |  |
| T1DM | 3.233 | 0.507 | 7.48 | 0.000 | 2.378 | 4.397 |
| T2DM | 1.164 | 0.067 | 2.66 | 0.008 | 1.041 | 1.302 |
| DM - nos | 2.303 | 0.259 | 7.42 | 0.000 | 1.847 | 2.871 |
|  |  |  |  |  |  |  |
| Antidiabetics_b | 1.800 | 0.109 | 9.70 | 0.000 | 1.599 | 2.028 |
| Diuretics_b | 2.366 | 0.072 | 28.27 | 0.000 | 2.229 | 2.511 |
| Anticoagulants_b | 1.357 | 0.055 | 7.56 | 0.000 | 1.254 | 1.468 |
| Antiplatelets_b | 0.818 | 0.074 | -2.22 | 0.026 | 0.685 | 0.977 |
| HF_b | 6.518 | 0.229 | 53.37 | 0.000 | 6.084 | 6.982 |
| AF_b | 1.691 | 0.066 | 13.47 | 0.000 | 1.566 | 1.825 |
| Heart_valve_dis_b | 1.223 | 0.127 | 1.94 | 0.052 | 0.998 | 1.499 |
| VT_VF_b | 2.128 | 0.217 | 7.42 | 0.000 | 1.744 | 2.598 |
| Cardiomyopathy_b | 1.403 | 0.151 | 3.15 | 0.002 | 1.137 | 1.732 |
| CV_procedures_b | 0.832 | 0.022 | -6.99 | 0.000 | 0.790 | 0.876 |
| TIA_stroke_b | 4.650 | 0.168 | 42.49 | 0.000 | 4.332 | 4.991 |
| PVD_b | 1.769 | 0.080 | 12.59 | 0.000 | 1.619 | 1.933 |
| CKD_b | 1.506 | 0.043 | 14.24 | 0.000 | 1.423 | 1.593 |
| Hypothyroidism_b | 0.983 | 0.041 | -0.40 | 0.687 | 0.905 | 1.068 |
| Liver_dis_b | 2.044 | 0.482 | 3.04 | 0.002 | 1.288 | 3.244 |
| Lupus_b | 1.457 | 0.310 | 1.77 | 0.077 | 0.960 | 2.211 |
| Erectile_dysfunction_b | 0.887 | 0.033 | -3.19 | 0.001 | 0.824 | 0.955 |
| Any_tumour_b | 1.417 | 0.044 | 11.10 | 0.000 | 1.332 | 1.507 |
| Menopause_b | 0.834 | 0.038 | -3.95 | 0.000 | 0.762 | 0.912 |
| Dementia_b | 3.584 | 0.239 | 19.13 | 0.000 | 3.144 | 4.084 |
| RA_b | 1.320 | 0.083 | 4.42 | 0.000 | 1.167 | 1.493 |
| num_ami_before_31_dec_14 | 0.999 | 0.001 | -0.52 | 0.600 | 0.997 | 1.002 |
| _cons | 0.009 | 0.002 | -24.01 | 0.000 | 0.006 | 0.013 |

**Performance in validation dataset (20% of practices) - Internal validation - audit AMI cases**

| AUROC (95% CI): 0.876 (0.869; 0.882) |  |
| --- | --- |
|  |  |
|  | % Correctly classified: 81.81 |

### 7) 1-year Composite 1 CV outcome (HF, stroke, recurrent MI, & CV mortality)

|  | | | | | | |
| --- | --- | --- | --- | --- | --- | --- |
| _1yr_Comp1_CV_outcome | Odds ratio | Std. err. | z | P>|z| | [95% conf. interval] | |
| age | 1.044 | 0.002 | 29.34 | 0.000 | 1.041 | 1.047 |
|  |  |  |  |  |  |  |
| gender |  |  |  |  |  |  |
| Female | 0.904 | 0.030 | -3.02 | 0.003 | 0.846 | 0.965 |
|  |  |  |  |  |  |  |
| IMD |  |  |  |  |  |  |
| Q2 | 1.100 | 0.045 | 2.31 | 0.021 | 1.015 | 1.192 |
| Q3 | 1.228 | 0.051 | 4.96 | 0.000 | 1.132 | 1.331 |
| Q4 | 1.210 | 0.050 | 4.58 | 0.000 | 1.115 | 1.313 |
| Q5-most deprived | 1.282 | 0.054 | 5.89 | 0.000 | 1.180 | 1.392 |
|  |  |  |  |  |  |  |
| ethnicity2 |  |  |  |  |  |  |
| non-White | 1.081 | 0.055 | 1.53 | 0.127 | 0.978 | 1.196 |
|  |  |  |  |  |  |  |
| Alcohol_status3 |  |  |  |  |  |  |
| drinker | 0.763 | 0.027 | -7.77 | 0.000 | 0.712 | 0.817 |
|  |  |  |  |  |  |  |
| Smoking_status |  |  |  |  |  |  |
| current smoker | 1.290 | 0.052 | 6.34 | 0.000 | 1.192 | 1.396 |
| ex-smoker | 0.982 | 0.035 | -0.51 | 0.613 | 0.915 | 1.054 |
|  |  |  |  |  |  |  |
| BMI_2014_f | 0.996 | 0.003 | -1.56 | 0.120 | 0.990 | 1.001 |
| HT_b | 1.000 | 0.028 | -0.02 | 0.987 | 0.947 | 1.055 |
| Antihypertensives_b | 0.518 | 0.072 | -4.76 | 0.000 | 0.395 | 0.679 |
| Hyperlipidaemia_b | 1.089 | 0.031 | 3.01 | 0.003 | 1.030 | 1.150 |
| Lipid_reg_treatment_b | 0.579 | 0.048 | -6.65 | 0.000 | 0.493 | 0.680 |
|  |  |  |  |  |  |  |
| Diabetes_type_au_b |  |  |  |  |  |  |
| T1DM | 2.458 | 0.386 | 5.73 | 0.000 | 1.807 | 3.344 |
| T2DM | 1.176 | 0.068 | 2.83 | 0.005 | 1.051 | 1.316 |
| DM - nos | 2.100 | 0.229 | 6.80 | 0.000 | 1.696 | 2.601 |
|  |  |  |  |  |  |  |
| Antidiabetics_b | 1.544 | 0.093 | 7.19 | 0.000 | 1.371 | 1.738 |
| Diuretics_b | 2.380 | 0.077 | 26.80 | 0.000 | 2.234 | 2.536 |
| Anticoagulants_b | 1.339 | 0.053 | 7.36 | 0.000 | 1.239 | 1.448 |
| Antiplatelets_b | 0.738 | 0.066 | -3.42 | 0.001 | 0.620 | 0.879 |
| HF_b | 6.645 | 0.217 | 57.94 | 0.000 | 6.233 | 7.085 |
| AF_b | 1.512 | 0.058 | 10.80 | 0.000 | 1.403 | 1.630 |
| Heart_valve_dis_b | 1.460 | 0.146 | 3.79 | 0.000 | 1.201 | 1.775 |
| VT_VF_b | 1.766 | 0.177 | 5.68 | 0.000 | 1.451 | 2.149 |
| Cardiomyopathy_b | 1.716 | 0.178 | 5.20 | 0.000 | 1.400 | 2.103 |
| CV_procedures_b | 0.915 | 0.025 | -3.30 | 0.001 | 0.868 | 0.965 |
| TIA_stroke_b | 4.151 | 0.142 | 41.61 | 0.000 | 3.882 | 4.439 |
| PVD_b | 1.628 | 0.073 | 10.92 | 0.000 | 1.492 | 1.777 |
| CKD_b | 1.373 | 0.040 | 10.90 | 0.000 | 1.297 | 1.454 |
| Hypothyroidism_b | 1.058 | 0.045 | 1.33 | 0.183 | 0.974 | 1.149 |
| Liver_dis_b | 2.730 | 0.616 | 4.45 | 0.000 | 1.755 | 4.247 |
| Lupus_b | 1.326 | 0.282 | 1.33 | 0.184 | 0.874 | 2.012 |
| Erectile_dysfunction_b | 0.936 | 0.036 | -1.74 | 0.083 | 0.868 | 1.009 |
| Any_tumour_b | 1.126 | 0.036 | 3.69 | 0.000 | 1.057 | 1.200 |
| Menopause_b | 0.869 | 0.041 | -2.99 | 0.003 | 0.792 | 0.953 |
| Dementia_b | 1.284 | 0.078 | 4.13 | 0.000 | 1.141 | 1.447 |
| RA_b | 1.253 | 0.079 | 3.56 | 0.000 | 1.107 | 1.419 |
| num_ami_before_31_dec_14 | 1.002 | 0.001 | 1.88 | 0.061 | 1.000 | 1.005 |
| _cons | 0.014 | 0.003 | -21.38 | 0.000 | 0.009 | 0.020 |

**Performance in validation dataset (20% of practices) - Internal validation - audit AMI cases**

| AUROC (95% CI): 0.852 (0.844; 0.859) |  |
| --- | --- |
|  |  |
|  | % Correctly classified: 81.12 |

### 8) 1-year Composite 2 outcome (HF, stroke, & all-cause mortality)

|  | | | | | | |
| --- | --- | --- | --- | --- | --- | --- |
| _1yr_Comp2_outcome | Odds ratio | Std. err. | z | P>|z| | [95% conf. interval] | |
| age | 1.067 | 0.002 | 42.39 | 0.000 | 1.064 | 1.071 |
|  |  |  |  |  |  |  |
| gender |  |  |  |  |  |  |
| Female | 1.003 | 0.034 | 0.07 | 0.940 | 0.938 | 1.072 |
|  |  |  |  |  |  |  |
| IMD |  |  |  |  |  |  |
| Q2 | 1.143 | 0.048 | 3.18 | 0.001 | 1.053 | 1.241 |
| Q3 | 1.325 | 0.056 | 6.65 | 0.000 | 1.220 | 1.440 |
| Q4 | 1.447 | 0.061 | 8.72 | 0.000 | 1.332 | 1.572 |
| Q5-most deprived | 1.495 | 0.065 | 9.30 | 0.000 | 1.374 | 1.627 |
|  |  |  |  |  |  |  |
| ethnicity2 |  |  |  |  |  |  |
| non-White | 0.904 | 0.049 | -1.86 | 0.063 | 0.813 | 1.006 |
|  |  |  |  |  |  |  |
| Alcohol_status3 |  |  |  |  |  |  |
| drinker | 0.815 | 0.030 | -5.64 | 0.000 | 0.759 | 0.875 |
|  |  |  |  |  |  |  |
| Smoking_status |  |  |  |  |  |  |
| current smoker | 1.600 | 0.066 | 11.32 | 0.000 | 1.475 | 1.735 |
| ex-smoker | 1.092 | 0.041 | 2.37 | 0.018 | 1.015 | 1.175 |
|  |  |  |  |  |  |  |
| BMI_2014_f | 0.975 | 0.003 | -8.85 | 0.000 | 0.970 | 0.981 |
| HT_b | 0.919 | 0.026 | -2.99 | 0.003 | 0.869 | 0.971 |
| Antihypertensives_b | 0.624 | 0.088 | -3.35 | 0.001 | 0.473 | 0.823 |
| Hyperlipidaemia_b | 1.007 | 0.029 | 0.25 | 0.806 | 0.951 | 1.066 |
| Lipid_reg_treatment_b | 0.359 | 0.031 | -11.70 | 0.000 | 0.302 | 0.426 |
|  |  |  |  |  |  |  |
| Diabetes_type_au_b |  |  |  |  |  |  |
| T1DM | 2.951 | 0.483 | 6.62 | 0.000 | 2.142 | 4.066 |
| T2DM | 1.167 | 0.069 | 2.61 | 0.009 | 1.040 | 1.311 |
| DM - nos | 2.199 | 0.253 | 6.86 | 0.000 | 1.756 | 2.755 |
|  |  |  |  |  |  |  |
| Antidiabetics_b | 1.721 | 0.108 | 8.66 | 0.000 | 1.522 | 1.945 |
| Diuretics_b | 2.619 | 0.084 | 29.88 | 0.000 | 2.459 | 2.790 |
| Anticoagulants_b | 1.335 | 0.055 | 6.98 | 0.000 | 1.231 | 1.447 |
| Antiplatelets_b | 0.677 | 0.063 | -4.22 | 0.000 | 0.565 | 0.812 |
| HF_b | 7.855 | 0.281 | 57.53 | 0.000 | 7.322 | 8.426 |
| AF_b | 1.598 | 0.064 | 11.75 | 0.000 | 1.478 | 1.728 |
| Heart_valve_dis_b | 1.558 | 0.165 | 4.20 | 0.000 | 1.267 | 1.917 |
| VT_VF_b | 2.001 | 0.210 | 6.62 | 0.000 | 1.629 | 2.457 |
| Cardiomyopathy_b | 1.725 | 0.189 | 4.97 | 0.000 | 1.391 | 2.138 |
| CV_procedures_b | 0.804 | 0.022 | -7.96 | 0.000 | 0.762 | 0.848 |
| TIA_stroke_b | 4.788 | 0.175 | 42.85 | 0.000 | 4.457 | 5.143 |
| PVD_b | 1.707 | 0.079 | 11.55 | 0.000 | 1.559 | 1.870 |
| CKD_b | 1.475 | 0.044 | 13.14 | 0.000 | 1.392 | 1.563 |
| Hypothyroidism_b | 0.993 | 0.043 | -0.17 | 0.863 | 0.912 | 1.080 |
| Liver_dis_b | 1.874 | 0.461 | 2.56 | 0.011 | 1.158 | 3.034 |
| Lupus_b | 1.320 | 0.294 | 1.24 | 0.214 | 0.852 | 2.043 |
| Erectile_dysfunction_b | 0.894 | 0.035 | -2.84 | 0.005 | 0.828 | 0.966 |
| Any_tumour_b | 1.498 | 0.048 | 12.53 | 0.000 | 1.406 | 1.596 |
| Menopause_b | 0.753 | 0.036 | -5.96 | 0.000 | 0.685 | 0.826 |
| Dementia_b | 3.864 | 0.258 | 20.22 | 0.000 | 3.390 | 4.405 |
| RA_b | 1.172 | 0.077 | 2.42 | 0.015 | 1.031 | 1.333 |
| num_ami_before_31_dec_14 | 0.999 | 0.001 | -0.49 | 0.627 | 0.997 | 1.002 |
| _cons | 0.006 | 0.001 | -24.92 | 0.000 | 0.004 | 0.009 |

**Performance in validation dataset (20% of practices) - Internal validation - audit AMI cases**

| AUROC (95% CI): 0.887 (0.880; 0.893) |  |
| --- | --- |
|  |  |
|  | % Correctly classified: 82.83 |

### 9) 1-year Composite 2 CV outcome (HF, stroke, & CV mortality)

|  | | | | | | |
| --- | --- | --- | --- | --- | --- | --- |
| _1yr_Comp2_CV_outcome | Odds ratio | Std. err. | z | P>|z| | [95% conf. interval] | |
| age | 1.047 | 0.002 | 29.38 | 0.000 | 1.044 | 1.050 |
|  |  |  |  |  |  |  |
| gender |  |  |  |  |  |  |
| Female | 1.007 | 0.035 | 0.20 | 0.843 | 0.940 | 1.078 |
|  |  |  |  |  |  |  |
| IMD |  |  |  |  |  |  |
| Q2 | 1.184 | 0.051 | 3.89 | 0.000 | 1.087 | 1.289 |
| Q3 | 1.209 | 0.053 | 4.34 | 0.000 | 1.110 | 1.318 |
| Q4 | 1.277 | 0.056 | 5.57 | 0.000 | 1.172 | 1.392 |
| Q5-most deprived | 1.350 | 0.060 | 6.74 | 0.000 | 1.237 | 1.473 |
|  |  |  |  |  |  |  |
| ethnicity2 |  |  |  |  |  |  |
| non-White | 1.028 | 0.056 | 0.51 | 0.612 | 0.924 | 1.144 |
|  |  |  |  |  |  |  |
| Alcohol_status3 |  |  |  |  |  |  |
| drinker | 0.788 | 0.029 | -6.49 | 0.000 | 0.734 | 0.847 |
|  |  |  |  |  |  |  |
| Smoking_status |  |  |  |  |  |  |
| current smoker | 1.367 | 0.058 | 7.34 | 0.000 | 1.257 | 1.486 |
| ex-smoker | 1.064 | 0.041 | 1.63 | 0.104 | 0.987 | 1.147 |
|  |  |  |  |  |  |  |
| BMI_2014_f | 0.995 | 0.003 | -1.66 | 0.097 | 0.990 | 1.001 |
| HT_b | 0.977 | 0.028 | -0.81 | 0.417 | 0.923 | 1.034 |
| Antihypertensives_b | 0.480 | 0.071 | -4.94 | 0.000 | 0.358 | 0.642 |
| Hyperlipidaemia_b | 1.073 | 0.032 | 2.39 | 0.017 | 1.013 | 1.137 |
| Lipid_reg_treatment_b | 0.755 | 0.065 | -3.27 | 0.001 | 0.637 | 0.893 |
|  |  |  |  |  |  |  |
| Diabetes_type_au_b |  |  |  |  |  |  |
| T1DM | 2.042 | 0.341 | 4.27 | 0.000 | 1.472 | 2.833 |
| T2DM | 1.107 | 0.067 | 1.67 | 0.094 | 0.983 | 1.246 |
| DM - nos | 1.559 | 0.178 | 3.88 | 0.000 | 1.245 | 1.951 |
|  |  |  |  |  |  |  |
| Antidiabetics_b | 1.600 | 0.102 | 7.39 | 0.000 | 1.413 | 1.813 |
| Diuretics_b | 2.561 | 0.089 | 26.99 | 0.000 | 2.392 | 2.742 |
| Anticoagulants_b | 1.316 | 0.054 | 6.70 | 0.000 | 1.215 | 1.426 |
| Antiplatelets_b | 0.673 | 0.062 | -4.29 | 0.000 | 0.562 | 0.807 |
| HF_b | 7.923 | 0.265 | 61.85 | 0.000 | 7.420 | 8.460 |
| AF_b | 1.646 | 0.065 | 12.65 | 0.000 | 1.524 | 1.778 |
| Heart_valve_dis_b | 1.575 | 0.161 | 4.45 | 0.000 | 1.289 | 1.924 |
| VT_VF_b | 1.706 | 0.177 | 5.14 | 0.000 | 1.392 | 2.091 |
| Cardiomyopathy_b | 1.839 | 0.195 | 5.75 | 0.000 | 1.494 | 2.263 |
| CV_procedures_b | 0.871 | 0.025 | -4.86 | 0.000 | 0.824 | 0.921 |
| TIA_stroke_b | 4.441 | 0.156 | 42.56 | 0.000 | 4.146 | 4.757 |
| PVD_b | 1.609 | 0.074 | 10.28 | 0.000 | 1.469 | 1.761 |
| CKD_b | 1.375 | 0.042 | 10.52 | 0.000 | 1.296 | 1.459 |
| Hypothyroidism_b | 1.099 | 0.048 | 2.16 | 0.031 | 1.009 | 1.197 |
| Liver_dis_b | 2.049 | 0.497 | 2.96 | 0.003 | 1.273 | 3.296 |
| Lupus_b | 1.817 | 0.389 | 2.79 | 0.005 | 1.194 | 2.764 |
| Erectile_dysfunction_b | 0.998 | 0.040 | -0.04 | 0.964 | 0.922 | 1.081 |
| Any_tumour_b | 1.124 | 0.038 | 3.49 | 0.000 | 1.053 | 1.201 |
| Menopause_b | 0.784 | 0.039 | -4.92 | 0.000 | 0.712 | 0.864 |
| Dementia_b | 1.366 | 0.085 | 5.04 | 0.000 | 1.210 | 1.542 |
| RA_b | 1.193 | 0.079 | 2.65 | 0.008 | 1.047 | 1.359 |
| num_ami_before_31_dec_14 | 1.002 | 0.001 | 1.42 | 0.155 | 0.999 | 1.005 |
| _cons | 0.007 | 0.001 | -22.98 | 0.000 | 0.004 | 0.010 |

**Performance in validation dataset (20% of practices) - Internal validation - audit AMI cases**

| AUROC (95% CI): 0.867 (0.860; 0.875) |  |
| --- | --- |
|  |  |
|  | % Correctly classified: 82.44 |

## External validation (CPRD GOLD) - with therapies

### 1) 1-year all-cause mortality

|  | | | | | | |
| --- | --- | --- | --- | --- | --- | --- |
| _1year_All_cause_death | Odds ratio | Std. err. | z | P>|z| | [95% conf. interval] | |
| age | 1.102 | 0.003 | 42.08 | 0.000 | 1.097 | 1.107 |
|  |  |  |  |  |  |  |
| gender |  |  |  |  |  |  |
| Female | 0.875 | 0.037 | -3.21 | 0.001 | 0.806 | 0.949 |
|  |  |  |  |  |  |  |
| IMD |  |  |  |  |  |  |
| Q2 | 1.021 | 0.056 | 0.37 | 0.710 | 0.916 | 1.137 |
| Q3 | 1.345 | 0.073 | 5.47 | 0.000 | 1.210 | 1.496 |
| Q4 | 1.451 | 0.079 | 6.82 | 0.000 | 1.304 | 1.615 |
| Q5-most deprived | 1.528 | 0.086 | 7.57 | 0.000 | 1.369 | 1.706 |
|  |  |  |  |  |  |  |
| ethnicity2 |  |  |  |  |  |  |
| non-White | 0.719 | 0.055 | -4.28 | 0.000 | 0.618 | 0.836 |
|  |  |  |  |  |  |  |
| Alcohol_status3 |  |  |  |  |  |  |
| drinker | 0.753 | 0.033 | -6.38 | 0.000 | 0.690 | 0.822 |
|  |  |  |  |  |  |  |
| Smoking_status |  |  |  |  |  |  |
| current smoker | 1.822 | 0.099 | 11.02 | 0.000 | 1.638 | 2.028 |
| ex-smoker | 1.240 | 0.059 | 4.53 | 0.000 | 1.130 | 1.361 |
|  |  |  |  |  |  |  |
| BMI_2014_f | 0.931 | 0.004 | -17.79 | 0.000 | 0.923 | 0.938 |
| HT_b | 0.839 | 0.031 | -4.83 | 0.000 | 0.781 | 0.901 |
| Antihypertensives_b | 0.930 | 0.185 | -0.37 | 0.714 | 0.630 | 1.373 |
| Hyperlipidaemia_b | 0.963 | 0.036 | -1.01 | 0.313 | 0.894 | 1.036 |
| Lipid_reg_treatment_b | 0.381 | 0.034 | -10.69 | 0.000 | 0.320 | 0.455 |
|  |  |  |  |  |  |  |
| Diabetes_type_au_b |  |  |  |  |  |  |
| T1DM | 2.181 | 0.487 | 3.49 | 0.000 | 1.408 | 3.379 |
| T2DM | 1.338 | 0.098 | 3.97 | 0.000 | 1.159 | 1.545 |
| DM - nos | 2.010 | 0.272 | 5.15 | 0.000 | 1.541 | 2.621 |
|  |  |  |  |  |  |  |
| Antidiabetics_b | 1.835 | 0.142 | 7.85 | 0.000 | 1.577 | 2.135 |
| Diuretics_b | 2.123 | 0.101 | 15.80 | 0.000 | 1.934 | 2.331 |
| Anticoagulants_b | 1.053 | 0.052 | 1.03 | 0.304 | 0.955 | 1.161 |
| Antiplatelets_b | 0.760 | 0.082 | -2.56 | 0.011 | 0.616 | 0.938 |
| HF_b | 1.926 | 0.078 | 16.25 | 0.000 | 1.780 | 2.085 |
| AF_b | 1.388 | 0.064 | 7.09 | 0.000 | 1.268 | 1.520 |
| Heart_valve_dis_b | 1.869 | 0.204 | 5.72 | 0.000 | 1.508 | 2.315 |
| VT_VF_b | 1.882 | 0.239 | 4.98 | 0.000 | 1.467 | 2.415 |
| Cardiomyopathy_b | 1.132 | 0.156 | 0.90 | 0.371 | 0.863 | 1.484 |
| CV_procedures_b | 0.659 | 0.024 | -11.25 | 0.000 | 0.613 | 0.709 |
| TIA_stroke_b | 1.434 | 0.061 | 8.49 | 0.000 | 1.319 | 1.558 |
| PVD_b | 1.492 | 0.081 | 7.40 | 0.000 | 1.342 | 1.659 |
| CKD_b | 1.537 | 0.056 | 11.88 | 0.000 | 1.432 | 1.651 |
| Hypothyroidism_b | 1.028 | 0.053 | 0.54 | 0.590 | 0.929 | 1.138 |
| Liver_dis_b | 2.498 | 0.800 | 2.86 | 0.004 | 1.333 | 4.681 |
| Lupus_b | 1.441 | 0.429 | 1.23 | 0.220 | 0.804 | 2.584 |
| Erectile_dysfunction_b | 0.813 | 0.045 | -3.72 | 0.000 | 0.729 | 0.907 |
| Any_tumour_b | 1.786 | 0.067 | 15.44 | 0.000 | 1.659 | 1.923 |
| Menopause_b | 0.613 | 0.040 | -7.43 | 0.000 | 0.539 | 0.698 |
| Dementia_b | 4.350 | 0.261 | 24.48 | 0.000 | 3.867 | 4.893 |
| RA_b | 1.847 | 0.142 | 8.00 | 0.000 | 1.589 | 2.147 |
| num_ami_before_31_dec_14 | 0.992 | 0.002 | -3.97 | 0.000 | 0.988 | 0.996 |
| _cons | 0.000 | 0.000 | -25.22 | 0.000 | 0.000 | 0.001 |

**Performance in validation dataset (CPRD GOLD) - External validation - audit AMI cases**

| AUROC (95% CI): 0.876 (0.864; 0.887) |  |
| --- | --- |
|  |  |
|  | % Correctly classified: 85.16 |

### 2) 1-year Stroke

|  | | | | | | |
| --- | --- | --- | --- | --- | --- | --- |
| _1year_Stroke | Odds ratio | Std. err. | z | P>|z| | [95% conf. interval] | |
| age | 1.031 | 0.002 | 12.82 | 0.000 | 1.026 | 1.035 |
|  |  |  |  |  |  |  |
| gender |  |  |  |  |  |  |
| Female | 1.076 | 0.055 | 1.43 | 0.153 | 0.973 | 1.189 |
|  |  |  |  |  |  |  |
| IMD |  |  |  |  |  |  |
| Q2 | 1.020 | 0.065 | 0.31 | 0.755 | 0.901 | 1.155 |
| Q3 | 1.017 | 0.065 | 0.26 | 0.792 | 0.897 | 1.154 |
| Q4 | 1.045 | 0.068 | 0.68 | 0.496 | 0.920 | 1.187 |
| Q5-most deprived | 1.162 | 0.075 | 2.33 | 0.020 | 1.024 | 1.319 |
|  |  |  |  |  |  |  |
| ethnicity2 |  |  |  |  |  |  |
| non-White | 0.947 | 0.077 | -0.67 | 0.504 | 0.807 | 1.111 |
|  |  |  |  |  |  |  |
| Alcohol_status3 |  |  |  |  |  |  |
| drinker | 0.820 | 0.043 | -3.78 | 0.000 | 0.740 | 0.909 |
|  |  |  |  |  |  |  |
| Smoking_status |  |  |  |  |  |  |
| current smoker | 1.141 | 0.071 | 2.11 | 0.035 | 1.010 | 1.289 |
| ex-smoker | 0.981 | 0.054 | -0.35 | 0.724 | 0.880 | 1.093 |
|  |  |  |  |  |  |  |
| BMI_2014_f | 0.976 | 0.004 | -5.51 | 0.000 | 0.968 | 0.985 |
| HT_b | 1.052 | 0.045 | 1.17 | 0.242 | 0.966 | 1.145 |
| Antihypertensives_b | 0.625 | 0.137 | -2.14 | 0.032 | 0.406 | 0.961 |
| Hyperlipidaemia_b | 1.078 | 0.046 | 1.75 | 0.080 | 0.991 | 1.173 |
| Lipid_reg_treatment_b | 0.843 | 0.106 | -1.36 | 0.174 | 0.659 | 1.078 |
|  |  |  |  |  |  |  |
| Diabetes_type_au_b |  |  |  |  |  |  |
| T1DM | 0.966 | 0.246 | -0.14 | 0.891 | 0.586 | 1.591 |
| T2DM | 0.772 | 0.074 | -2.69 | 0.007 | 0.639 | 0.932 |
| DM - nos | 1.455 | 0.227 | 2.40 | 0.016 | 1.071 | 1.977 |
|  |  |  |  |  |  |  |
| Antidiabetics_b | 1.921 | 0.191 | 6.56 | 0.000 | 1.581 | 2.335 |
| Diuretics_b | 1.592 | 0.084 | 8.81 | 0.000 | 1.435 | 1.765 |
| Anticoagulants_b | 1.120 | 0.065 | 1.96 | 0.051 | 1.000 | 1.256 |
| Antiplatelets_b | 0.913 | 0.125 | -0.66 | 0.508 | 0.698 | 1.195 |
| HF_b | 0.994 | 0.052 | -0.11 | 0.911 | 0.898 | 1.101 |
| AF_b | 1.392 | 0.077 | 5.96 | 0.000 | 1.248 | 1.551 |
| Heart_valve_dis_b | 1.048 | 0.145 | 0.34 | 0.732 | 0.800 | 1.374 |
| VT_VF_b | 1.167 | 0.190 | 0.95 | 0.342 | 0.848 | 1.606 |
| Cardiomyopathy_b | 0.704 | 0.131 | -1.88 | 0.060 | 0.489 | 1.015 |
| CV_procedures_b | 0.869 | 0.037 | -3.30 | 0.001 | 0.800 | 0.945 |
| TIA_stroke_b | 11.152 | 0.457 | 58.79 | 0.000 | 10.290 | 12.085 |
| PVD_b | 1.188 | 0.076 | 2.69 | 0.007 | 1.048 | 1.347 |
| CKD_b | 1.136 | 0.051 | 2.86 | 0.004 | 1.041 | 1.240 |
| Hypothyroidism_b | 0.978 | 0.062 | -0.36 | 0.720 | 0.864 | 1.106 |
| Liver_dis_b | 1.820 | 0.648 | 1.68 | 0.093 | 0.905 | 3.659 |
| Lupus_b | 0.816 | 0.275 | -0.60 | 0.546 | 0.421 | 1.580 |
| Erectile_dysfunction_b | 1.043 | 0.064 | 0.69 | 0.490 | 0.926 | 1.175 |
| Any_tumour_b | 1.126 | 0.054 | 2.47 | 0.013 | 1.025 | 1.237 |
| Menopause_b | 0.954 | 0.068 | -0.66 | 0.509 | 0.830 | 1.097 |
| Dementia_b | 1.781 | 0.132 | 7.78 | 0.000 | 1.540 | 2.060 |
| RA_b | 0.885 | 0.091 | -1.19 | 0.235 | 0.723 | 1.083 |
| num_ami_before_31_dec_14 | 1.003 | 0.002 | 1.71 | 0.088 | 1.000 | 1.007 |
| _cons | 0.009 | 0.003 | -14.34 | 0.000 | 0.005 | 0.017 |

**Performance in validation dataset (CPRD GOLD) - External validation - audit AMI cases**

| AUROC (95% CI): 0.810 (0.791; 0.830) |  |
| --- | --- |
|  |  |
|  | % Correctly classified: 87.67 |

### 3) 1-year heart failure (HF)

|  | | | | | | |
| --- | --- | --- | --- | --- | --- | --- |
| _1year_HF | Odds ratio | Std. err. | z | P>|z| | [95% conf. interval] | |
| age | 1.039 | 0.002 | 20.82 | 0.000 | 1.035 | 1.042 |
|  |  |  |  |  |  |  |
| gender |  |  |  |  |  |  |
| Female | 0.957 | 0.039 | -1.09 | 0.278 | 0.884 | 1.036 |
|  |  |  |  |  |  |  |
| IMD |  |  |  |  |  |  |
| Q2 | 1.200 | 0.062 | 3.54 | 0.000 | 1.085 | 1.327 |
| Q3 | 1.249 | 0.065 | 4.30 | 0.000 | 1.129 | 1.382 |
| Q4 | 1.328 | 0.068 | 5.50 | 0.000 | 1.200 | 1.469 |
| Q5-most deprived | 1.451 | 0.076 | 7.14 | 0.000 | 1.310 | 1.607 |
|  |  |  |  |  |  |  |
| ethnicity2 |  |  |  |  |  |  |
| non-White | 1.133 | 0.071 | 1.99 | 0.047 | 1.002 | 1.283 |
|  |  |  |  |  |  |  |
| Alcohol_status3 |  |  |  |  |  |  |
| drinker | 0.804 | 0.034 | -5.17 | 0.000 | 0.740 | 0.873 |
|  |  |  |  |  |  |  |
| Smoking_status |  |  |  |  |  |  |
| current smoker | 1.276 | 0.063 | 4.91 | 0.000 | 1.158 | 1.407 |
| ex-smoker | 1.048 | 0.046 | 1.06 | 0.288 | 0.961 | 1.143 |
|  |  |  |  |  |  |  |
| BMI_2014_f | 1.001 | 0.003 | 0.36 | 0.717 | 0.995 | 1.008 |
| HT_b | 0.945 | 0.032 | -1.69 | 0.091 | 0.884 | 1.009 |
| Antihypertensives_b | 0.505 | 0.096 | -3.58 | 0.000 | 0.348 | 0.734 |
| Hyperlipidaemia_b | 1.038 | 0.036 | 1.08 | 0.281 | 0.970 | 1.110 |
| Lipid_reg_treatment_b | 0.647 | 0.061 | -4.60 | 0.000 | 0.537 | 0.779 |
|  |  |  |  |  |  |  |
| Diabetes_type_au_b |  |  |  |  |  |  |
| T1DM | 2.959 | 0.555 | 5.78 | 0.000 | 2.049 | 4.274 |
| T2DM | 1.409 | 0.094 | 5.11 | 0.000 | 1.235 | 1.606 |
| DM - nos | 1.526 | 0.197 | 3.27 | 0.001 | 1.184 | 1.966 |
|  |  |  |  |  |  |  |
| Antidiabetics_b | 1.115 | 0.079 | 1.54 | 0.124 | 0.971 | 1.280 |
| Diuretics_b | 3.381 | 0.156 | 26.48 | 0.000 | 3.090 | 3.700 |
| Anticoagulants_b | 1.412 | 0.064 | 7.61 | 0.000 | 1.292 | 1.544 |
| Antiplatelets_b | 0.592 | 0.060 | -5.14 | 0.000 | 0.485 | 0.723 |
| HF_b | 12.226 | 0.417 | 73.48 | 0.000 | 11.436 | 13.070 |
| AF_b | 1.602 | 0.070 | 10.77 | 0.000 | 1.471 | 1.746 |
| Heart_valve_dis_b | 1.873 | 0.203 | 5.80 | 0.000 | 1.515 | 2.315 |
| VT_VF_b | 1.791 | 0.200 | 5.21 | 0.000 | 1.438 | 2.229 |
| Cardiomyopathy_b | 1.886 | 0.205 | 5.85 | 0.000 | 1.524 | 2.333 |
| CV_procedures_b | 0.886 | 0.030 | -3.62 | 0.000 | 0.830 | 0.946 |
| TIA_stroke_b | 1.359 | 0.057 | 7.32 | 0.000 | 1.252 | 1.475 |
| PVD_b | 1.642 | 0.085 | 9.60 | 0.000 | 1.484 | 1.817 |
| CKD_b | 1.409 | 0.049 | 9.88 | 0.000 | 1.317 | 1.509 |
| Hypothyroidism_b | 1.073 | 0.053 | 1.41 | 0.157 | 0.973 | 1.183 |
| Liver_dis_b | 1.398 | 0.412 | 1.14 | 0.255 | 0.785 | 2.492 |
| Lupus_b | 0.951 | 0.255 | -0.19 | 0.851 | 0.563 | 1.607 |
| Erectile_dysfunction_b | 0.980 | 0.047 | -0.43 | 0.666 | 0.893 | 1.075 |
| Any_tumour_b | 1.191 | 0.046 | 4.51 | 0.000 | 1.104 | 1.284 |
| Menopause_b | 0.839 | 0.049 | -3.02 | 0.002 | 0.749 | 0.940 |
| Dementia_b | 1.222 | 0.085 | 2.87 | 0.004 | 1.066 | 1.401 |
| RA_b | 1.155 | 0.089 | 1.87 | 0.062 | 0.993 | 1.344 |
| num_ami_before_31_dec_14 | 1.003 | 0.002 | 2.27 | 0.023 | 1.000 | 1.006 |
| _cons | 0.006 | 0.001 | -19.87 | 0.000 | 0.003 | 0.009 |

**Performance in validation dataset (CPRD GOLD) - External validation - audit AMI cases**

| AUROC (95% CI): 0.747 (0.726; 0.768) |  |
| --- | --- |
|  |  |
|  | % Correctly classified: 79.66 |

### 4) 1-year Recurrent MI

|  | | | | | | |
| --- | --- | --- | --- | --- | --- | --- |
| _1year_Recurrent_MI | Odds ratio | Std. err. | z | P>|z| | [95% conf. interval] | |
| age | 1.024 | 0.003 | 8.10 | 0.000 | 1.018 | 1.030 |
|  |  |  |  |  |  |  |
| gender |  |  |  |  |  |  |
| Female | 0.959 | 0.064 | -0.62 | 0.533 | 0.841 | 1.094 |
|  |  |  |  |  |  |  |
| IMD |  |  |  |  |  |  |
| Q2 | 1.052 | 0.091 | 0.59 | 0.556 | 0.889 | 1.245 |
| Q3 | 1.220 | 0.103 | 2.36 | 0.018 | 1.034 | 1.438 |
| Q4 | 1.061 | 0.092 | 0.68 | 0.496 | 0.895 | 1.257 |
| Q5-most deprived | 1.238 | 0.106 | 2.50 | 0.012 | 1.047 | 1.464 |
|  |  |  |  |  |  |  |
| ethnicity2 |  |  |  |  |  |  |
| non-White | 1.064 | 0.103 | 0.64 | 0.520 | 0.880 | 1.287 |
|  |  |  |  |  |  |  |
| Alcohol_status3 |  |  |  |  |  |  |
| drinker | 0.762 | 0.051 | -4.09 | 0.000 | 0.669 | 0.868 |
|  |  |  |  |  |  |  |
| Smoking_status |  |  |  |  |  |  |
| current smoker | 1.190 | 0.096 | 2.17 | 0.030 | 1.017 | 1.393 |
| ex-smoker | 0.926 | 0.068 | -1.05 | 0.293 | 0.801 | 1.069 |
|  |  |  |  |  |  |  |
| BMI_2014_f | 0.996 | 0.006 | -0.76 | 0.447 | 0.985 | 1.007 |
| HT_b | 1.029 | 0.058 | 0.51 | 0.611 | 0.921 | 1.150 |
| Antihypertensives_b | 0.415 | 0.090 | -4.08 | 0.000 | 0.271 | 0.633 |
| Hyperlipidaemia_b | 1.166 | 0.065 | 2.74 | 0.006 | 1.045 | 1.302 |
| Lipid_reg_treatment_b | 0.651 | 0.095 | -2.94 | 0.003 | 0.489 | 0.867 |
|  |  |  |  |  |  |  |
| Diabetes_type_au_b |  |  |  |  |  |  |
| T1DM | 2.344 | 0.576 | 3.47 | 0.001 | 1.449 | 3.793 |
| T2DM | 1.062 | 0.127 | 0.50 | 0.615 | 0.840 | 1.343 |
| DM - nos | 1.988 | 0.358 | 3.82 | 0.000 | 1.397 | 2.829 |
|  |  |  |  |  |  |  |
| Antidiabetics_b | 1.674 | 0.205 | 4.20 | 0.000 | 1.317 | 2.129 |
| Diuretics_b | 1.511 | 0.104 | 6.01 | 0.000 | 1.321 | 1.729 |
| Anticoagulants_b | 0.874 | 0.075 | -1.56 | 0.119 | 0.738 | 1.035 |
| Antiplatelets_b | 0.605 | 0.095 | -3.20 | 0.001 | 0.445 | 0.823 |
| HF_b | 1.297 | 0.086 | 3.93 | 0.000 | 1.139 | 1.478 |
| AF_b | 0.886 | 0.073 | -1.47 | 0.141 | 0.754 | 1.041 |
| Heart_valve_dis_b | 1.245 | 0.234 | 1.17 | 0.242 | 0.862 | 1.799 |
| VT_VF_b | 1.111 | 0.237 | 0.49 | 0.621 | 0.732 | 1.686 |
| Cardiomyopathy_b | 0.835 | 0.205 | -0.74 | 0.461 | 0.516 | 1.350 |
| CV_procedures_b | 0.949 | 0.052 | -0.95 | 0.342 | 0.853 | 1.057 |
| TIA_stroke_b | 1.125 | 0.080 | 1.65 | 0.098 | 0.978 | 1.293 |
| PVD_b | 1.678 | 0.131 | 6.63 | 0.000 | 1.440 | 1.955 |
| CKD_b | 1.299 | 0.078 | 4.39 | 0.000 | 1.156 | 1.461 |
| Hypothyroidism_b | 1.039 | 0.086 | 0.46 | 0.643 | 0.883 | 1.223 |
| Liver_dis_b | 1.746 | 0.701 | 1.39 | 0.165 | 0.795 | 3.834 |
| Lupus_b | 0.371 | 0.266 | -1.38 | 0.166 | 0.091 | 1.510 |
| Erectile_dysfunction_b | 0.918 | 0.071 | -1.10 | 0.270 | 0.790 | 1.068 |
| Any_tumour_b | 1.141 | 0.073 | 2.05 | 0.041 | 1.006 | 1.294 |
| Menopause_b | 0.777 | 0.078 | -2.51 | 0.012 | 0.639 | 0.946 |
| Dementia_b | 1.268 | 0.140 | 2.15 | 0.031 | 1.022 | 1.575 |
| RA_b | 1.339 | 0.162 | 2.42 | 0.015 | 1.058 | 1.697 |
| num_ami_before_31_dec_14 | 0.999 | 0.003 | -0.49 | 0.624 | 0.993 | 1.004 |
| _cons | 0.022 | 0.008 | -10.69 | 0.000 | 0.011 | 0.044 |

**Performance in validation dataset (CPRD GOLD) - External validation - audit AMI cases**

| AUROC (95% CI): 0.718 (0.688; 0.748) |  |
| --- | --- |
|  |  |
|  | % Correctly classified: 75.34 |

### 5) 1-year CV mortality

|  | | | | | | |
| --- | --- | --- | --- | --- | --- | --- |
| _1year_CV_death | Odds ratio | Std. err. | z | P>|z| | [95% conf. interval] | |
| age | 1.069 | 0.004 | 16.97 | 0.000 | 1.061 | 1.078 |
|  |  |  |  |  |  |  |
| gender |  |  |  |  |  |  |
| Female | 0.857 | 0.064 | -2.07 | 0.038 | 0.741 | 0.992 |
|  |  |  |  |  |  |  |
| IMD |  |  |  |  |  |  |
| Q2 | 1.155 | 0.115 | 1.44 | 0.149 | 0.949 | 1.405 |
| Q3 | 1.434 | 0.139 | 3.72 | 0.000 | 1.186 | 1.734 |
| Q4 | 1.111 | 0.114 | 1.03 | 0.305 | 0.909 | 1.359 |
| Q5-most deprived | 1.416 | 0.143 | 3.45 | 0.001 | 1.162 | 1.725 |
|  |  |  |  |  |  |  |
| ethnicity2 |  |  |  |  |  |  |
| non-White | 0.783 | 0.105 | -1.82 | 0.068 | 0.602 | 1.019 |
|  |  |  |  |  |  |  |
| Alcohol_status3 |  |  |  |  |  |  |
| drinker | 0.966 | 0.077 | -0.44 | 0.663 | 0.826 | 1.129 |
|  |  |  |  |  |  |  |
| Smoking_status |  |  |  |  |  |  |
| current smoker | 1.514 | 0.150 | 4.19 | 0.000 | 1.247 | 1.838 |
| ex-smoker | 1.227 | 0.107 | 2.35 | 0.019 | 1.034 | 1.455 |
|  |  |  |  |  |  |  |
| BMI_2014_f | 0.966 | 0.007 | -5.02 | 0.000 | 0.953 | 0.979 |
| HT_b | 0.921 | 0.059 | -1.28 | 0.200 | 0.812 | 1.045 |
| Antihypertensives_b | 0.300 | 0.090 | -4.01 | 0.000 | 0.167 | 0.541 |
| Hyperlipidaemia_b | 1.114 | 0.073 | 1.65 | 0.098 | 0.980 | 1.266 |
| Lipid_reg_treatment_b | 1.029 | 0.168 | 0.18 | 0.860 | 0.748 | 1.417 |
|  |  |  |  |  |  |  |
| Diabetes_type_au_b |  |  |  |  |  |  |
| T1DM | 2.431 | 0.779 | 2.77 | 0.006 | 1.297 | 4.558 |
| T2DM | 1.289 | 0.166 | 1.97 | 0.049 | 1.001 | 1.660 |
| DM - nos | 1.978 | 0.418 | 3.23 | 0.001 | 1.308 | 2.993 |
|  |  |  |  |  |  |  |
| Antidiabetics_b | 1.885 | 0.250 | 4.78 | 0.000 | 1.454 | 2.444 |
| Diuretics_b | 2.594 | 0.263 | 9.42 | 0.000 | 2.127 | 3.164 |
| Anticoagulants_b | 1.006 | 0.086 | 0.07 | 0.945 | 0.851 | 1.189 |
| Antiplatelets_b | 0.616 | 0.104 | -2.88 | 0.004 | 0.443 | 0.856 |
| HF_b | 2.045 | 0.136 | 10.77 | 0.000 | 1.795 | 2.329 |
| AF_b | 1.340 | 0.106 | 3.71 | 0.000 | 1.148 | 1.565 |
| Heart_valve_dis_b | 1.263 | 0.228 | 1.29 | 0.196 | 0.887 | 1.799 |
| VT_VF_b | 2.503 | 0.427 | 5.38 | 0.000 | 1.792 | 3.497 |
| Cardiomyopathy_b | 2.072 | 0.377 | 4.01 | 0.000 | 1.451 | 2.959 |
| CV_procedures_b | 0.790 | 0.053 | -3.54 | 0.000 | 0.693 | 0.900 |
| TIA_stroke_b | 1.335 | 0.097 | 3.99 | 0.000 | 1.159 | 1.539 |
| PVD_b | 1.249 | 0.115 | 2.42 | 0.016 | 1.043 | 1.496 |
| CKD_b | 1.263 | 0.083 | 3.57 | 0.000 | 1.111 | 1.436 |
| Hypothyroidism_b | 0.893 | 0.084 | -1.20 | 0.229 | 0.743 | 1.074 |
| Liver_dis_b | 2.808 | 1.357 | 2.14 | 0.033 | 1.089 | 7.243 |
| Lupus_b | 0.259 | 0.262 | -1.33 | 0.183 | 0.035 | 1.889 |
| Erectile_dysfunction_b | 0.847 | 0.082 | -1.73 | 0.084 | 0.701 | 1.023 |
| Any_tumour_b | 0.954 | 0.068 | -0.66 | 0.510 | 0.830 | 1.097 |
| Menopause_b | 0.739 | 0.091 | -2.45 | 0.014 | 0.581 | 0.941 |
| Dementia_b | 1.760 | 0.172 | 5.78 | 0.000 | 1.453 | 2.132 |
| RA_b | 1.253 | 0.179 | 1.58 | 0.113 | 0.948 | 1.658 |
| num_ami_before_31_dec_14 | 0.994 | 0.004 | -1.42 | 0.154 | 0.987 | 1.002 |
| _cons | 0.000 | 0.000 | -15.41 | 0.000 | 0.000 | 0.001 |

**Performance in validation dataset (CPRD GOLD) - External validation - audit AMI cases**

| AUROC (95% CI): 0.815 (0.784; 0.845) |  |
| --- | --- |
|  |  |
|  | % Correctly classified: 83.66 |

### 6) 1-year Composite 1 outcome (HF, stroke, recurrent MI, & all-cause mortality)

|  | | | | | | |
| --- | --- | --- | --- | --- | --- | --- |
| _1yr_Comp1_outcome | Odds ratio | Std. err. | z | P>|z| | [95% conf. interval] | |
| age | 1.063 | 0.002 | 42.01 | 0.000 | 1.060 | 1.066 |
|  |  |  |  |  |  |  |
| gender |  |  |  |  |  |  |
| Female | 0.911 | 0.030 | -2.84 | 0.005 | 0.853 | 0.971 |
|  |  |  |  |  |  |  |
| IMD |  |  |  |  |  |  |
| Q2 | 1.136 | 0.046 | 3.16 | 0.002 | 1.050 | 1.230 |
| Q3 | 1.343 | 0.055 | 7.27 | 0.000 | 1.241 | 1.455 |
| Q4 | 1.400 | 0.057 | 8.26 | 0.000 | 1.293 | 1.516 |
| Q5-most deprived | 1.459 | 0.061 | 9.10 | 0.000 | 1.345 | 1.583 |
|  |  |  |  |  |  |  |
| ethnicity2 |  |  |  |  |  |  |
| non-White | 0.917 | 0.048 | -1.66 | 0.096 | 0.829 | 1.015 |
|  |  |  |  |  |  |  |
| Alcohol_status3 |  |  |  |  |  |  |
| drinker | 0.821 | 0.029 | -5.63 | 0.000 | 0.767 | 0.879 |
|  |  |  |  |  |  |  |
| Smoking_status |  |  |  |  |  |  |
| current smoker | 1.584 | 0.063 | 11.52 | 0.000 | 1.465 | 1.713 |
| ex-smoker | 1.110 | 0.040 | 2.90 | 0.004 | 1.034 | 1.191 |
|  |  |  |  |  |  |  |
| BMI_2014_f | 0.975 | 0.003 | -9.14 | 0.000 | 0.970 | 0.981 |
| HT_b | 0.970 | 0.026 | -1.11 | 0.266 | 0.920 | 1.023 |
| Antihypertensives_b | 0.619 | 0.083 | -3.56 | 0.000 | 0.476 | 0.806 |
| Hyperlipidaemia_b | 1.000 | 0.028 | 0.00 | 0.997 | 0.947 | 1.056 |
| Lipid_reg_treatment_b | 0.334 | 0.029 | -12.67 | 0.000 | 0.282 | 0.396 |
|  |  |  |  |  |  |  |
| Diabetes_type_au_b |  |  |  |  |  |  |
| T1DM | 3.233 | 0.507 | 7.48 | 0.000 | 2.378 | 4.397 |
| T2DM | 1.164 | 0.067 | 2.66 | 0.008 | 1.041 | 1.302 |
| DM - nos | 2.303 | 0.259 | 7.42 | 0.000 | 1.847 | 2.871 |
|  |  |  |  |  |  |  |
| Antidiabetics_b | 1.800 | 0.109 | 9.70 | 0.000 | 1.599 | 2.028 |
| Diuretics_b | 2.366 | 0.072 | 28.27 | 0.000 | 2.229 | 2.511 |
| Anticoagulants_b | 1.357 | 0.055 | 7.56 | 0.000 | 1.254 | 1.468 |
| Antiplatelets_b | 0.818 | 0.074 | -2.22 | 0.026 | 0.685 | 0.977 |
| HF_b | 6.518 | 0.229 | 53.37 | 0.000 | 6.084 | 6.982 |
| AF_b | 1.691 | 0.066 | 13.47 | 0.000 | 1.566 | 1.825 |
| Heart_valve_dis_b | 1.223 | 0.127 | 1.94 | 0.052 | 0.998 | 1.499 |
| VT_VF_b | 2.128 | 0.217 | 7.42 | 0.000 | 1.744 | 2.598 |
| Cardiomyopathy_b | 1.403 | 0.151 | 3.15 | 0.002 | 1.137 | 1.732 |
| CV_procedures_b | 0.832 | 0.022 | -6.99 | 0.000 | 0.790 | 0.876 |
| TIA_stroke_b | 4.650 | 0.168 | 42.49 | 0.000 | 4.332 | 4.991 |
| PVD_b | 1.769 | 0.080 | 12.59 | 0.000 | 1.619 | 1.933 |
| CKD_b | 1.506 | 0.043 | 14.24 | 0.000 | 1.423 | 1.593 |
| Hypothyroidism_b | 0.983 | 0.041 | -0.40 | 0.687 | 0.905 | 1.068 |
| Liver_dis_b | 2.044 | 0.482 | 3.04 | 0.002 | 1.288 | 3.244 |
| Lupus_b | 1.457 | 0.310 | 1.77 | 0.077 | 0.960 | 2.211 |
| Erectile_dysfunction_b | 0.887 | 0.033 | -3.19 | 0.001 | 0.824 | 0.955 |
| Any_tumour_b | 1.417 | 0.044 | 11.10 | 0.000 | 1.332 | 1.507 |
| Menopause_b | 0.834 | 0.038 | -3.95 | 0.000 | 0.762 | 0.912 |
| Dementia_b | 3.584 | 0.239 | 19.13 | 0.000 | 3.144 | 4.084 |
| RA_b | 1.320 | 0.083 | 4.42 | 0.000 | 1.167 | 1.493 |
| num_ami_before_31_dec_14 | 0.999 | 0.001 | -0.52 | 0.600 | 0.997 | 1.002 |
| _cons | 0.009 | 0.002 | -24.01 | 0.000 | 0.006 | 0.013 |

**Performance in validation dataset (CPRD GOLD) - External validation - audit AMI cases**

| AUROC (95% CI): 0.818 (0.808; 0.828) |  |
| --- | --- |
|  |  |
|  | % Correctly classified: 76.76 |

### 7) 1-year Composite 1 CV outcome (HF, stroke, recurrent MI, & CV mortality)

|  | | | | | | |
| --- | --- | --- | --- | --- | --- | --- |
| _1yr_Comp1_CV_outcome | Odds ratio | Std. err. | z | P>|z| | [95% conf. interval] | |
| age | 1.044 | 0.002 | 29.34 | 0.000 | 1.041 | 1.047 |
|  |  |  |  |  |  |  |
| gender |  |  |  |  |  |  |
| Female | 0.904 | 0.030 | -3.02 | 0.003 | 0.846 | 0.965 |
|  |  |  |  |  |  |  |
| IMD |  |  |  |  |  |  |
| Q2 | 1.100 | 0.045 | 2.31 | 0.021 | 1.015 | 1.192 |
| Q3 | 1.228 | 0.051 | 4.96 | 0.000 | 1.132 | 1.331 |
| Q4 | 1.210 | 0.050 | 4.58 | 0.000 | 1.115 | 1.313 |
| Q5-most deprived | 1.282 | 0.054 | 5.89 | 0.000 | 1.180 | 1.392 |
|  |  |  |  |  |  |  |
| ethnicity2 |  |  |  |  |  |  |
| non-White | 1.081 | 0.055 | 1.53 | 0.127 | 0.978 | 1.196 |
|  |  |  |  |  |  |  |
| Alcohol_status3 |  |  |  |  |  |  |
| drinker | 0.763 | 0.027 | -7.77 | 0.000 | 0.712 | 0.817 |
|  |  |  |  |  |  |  |
| Smoking_status |  |  |  |  |  |  |
| current smoker | 1.290 | 0.052 | 6.34 | 0.000 | 1.192 | 1.396 |
| ex-smoker | 0.982 | 0.035 | -0.51 | 0.613 | 0.915 | 1.054 |
|  |  |  |  |  |  |  |
| BMI_2014_f | 0.996 | 0.003 | -1.56 | 0.120 | 0.990 | 1.001 |
| HT_b | 1.000 | 0.028 | -0.02 | 0.987 | 0.947 | 1.055 |
| Antihypertensives_b | 0.518 | 0.072 | -4.76 | 0.000 | 0.395 | 0.679 |
| Hyperlipidaemia_b | 1.089 | 0.031 | 3.01 | 0.003 | 1.030 | 1.150 |
| Lipid_reg_treatment_b | 0.579 | 0.048 | -6.65 | 0.000 | 0.493 | 0.680 |
|  |  |  |  |  |  |  |
| Diabetes_type_au_b |  |  |  |  |  |  |
| T1DM | 2.458 | 0.386 | 5.73 | 0.000 | 1.807 | 3.344 |
| T2DM | 1.176 | 0.068 | 2.83 | 0.005 | 1.051 | 1.316 |
| DM - nos | 2.100 | 0.229 | 6.80 | 0.000 | 1.696 | 2.601 |
|  |  |  |  |  |  |  |
| Antidiabetics_b | 1.544 | 0.093 | 7.19 | 0.000 | 1.371 | 1.738 |
| Diuretics_b | 2.380 | 0.077 | 26.80 | 0.000 | 2.234 | 2.536 |
| Anticoagulants_b | 1.339 | 0.053 | 7.36 | 0.000 | 1.239 | 1.448 |
| Antiplatelets_b | 0.738 | 0.066 | -3.42 | 0.001 | 0.620 | 0.879 |
| HF_b | 6.645 | 0.217 | 57.94 | 0.000 | 6.233 | 7.085 |
| AF_b | 1.512 | 0.058 | 10.80 | 0.000 | 1.403 | 1.630 |
| Heart_valve_dis_b | 1.460 | 0.146 | 3.79 | 0.000 | 1.201 | 1.775 |
| VT_VF_b | 1.766 | 0.177 | 5.68 | 0.000 | 1.451 | 2.149 |
| Cardiomyopathy_b | 1.716 | 0.178 | 5.20 | 0.000 | 1.400 | 2.103 |
| CV_procedures_b | 0.915 | 0.025 | -3.30 | 0.001 | 0.868 | 0.965 |
| TIA_stroke_b | 4.151 | 0.142 | 41.61 | 0.000 | 3.882 | 4.439 |
| PVD_b | 1.628 | 0.073 | 10.92 | 0.000 | 1.492 | 1.777 |
| CKD_b | 1.373 | 0.040 | 10.90 | 0.000 | 1.297 | 1.454 |
| Hypothyroidism_b | 1.058 | 0.045 | 1.33 | 0.183 | 0.974 | 1.149 |
| Liver_dis_b | 2.730 | 0.616 | 4.45 | 0.000 | 1.755 | 4.247 |
| Lupus_b | 1.326 | 0.282 | 1.33 | 0.184 | 0.874 | 2.012 |
| Erectile_dysfunction_b | 0.936 | 0.036 | -1.74 | 0.083 | 0.868 | 1.009 |
| Any_tumour_b | 1.126 | 0.036 | 3.69 | 0.000 | 1.057 | 1.200 |
| Menopause_b | 0.869 | 0.041 | -2.99 | 0.003 | 0.792 | 0.953 |
| Dementia_b | 1.284 | 0.078 | 4.13 | 0.000 | 1.141 | 1.447 |
| RA_b | 1.253 | 0.079 | 3.56 | 0.000 | 1.107 | 1.419 |
| num_ami_before_31_dec_14 | 1.002 | 0.001 | 1.88 | 0.061 | 1.000 | 1.005 |
| _cons | 0.014 | 0.003 | -21.38 | 0.000 | 0.009 | 0.020 |

**Performance in validation dataset (CPRD GOLD) - External validation - audit AMI cases**

| AUROC (95% CI): 0.763 (0.749; 0.777) |  |
| --- | --- |
|  |  |
|  | % Correctly classified: 75.30 |

### 8) 1-year Composite 2 outcome (HF, stroke, & all-cause mortality)

|  | | | | | | |
| --- | --- | --- | --- | --- | --- | --- |
| _1yr_Comp2_outcome | Odds ratio | Std. err. | z | P>|z| | [95% conf. interval] | |
| age | 1.067 | 0.002 | 42.39 | 0.000 | 1.064 | 1.071 |
|  |  |  |  |  |  |  |
| gender |  |  |  |  |  |  |
| Female | 1.003 | 0.034 | 0.07 | 0.940 | 0.938 | 1.072 |
|  |  |  |  |  |  |  |
| IMD |  |  |  |  |  |  |
| Q2 | 1.143 | 0.048 | 3.18 | 0.001 | 1.053 | 1.241 |
| Q3 | 1.325 | 0.056 | 6.65 | 0.000 | 1.220 | 1.440 |
| Q4 | 1.447 | 0.061 | 8.72 | 0.000 | 1.332 | 1.572 |
| Q5-most deprived | 1.495 | 0.065 | 9.30 | 0.000 | 1.374 | 1.627 |
|  |  |  |  |  |  |  |
| ethnicity2 |  |  |  |  |  |  |
| non-White | 0.904 | 0.049 | -1.86 | 0.063 | 0.813 | 1.006 |
|  |  |  |  |  |  |  |
| Alcohol_status3 |  |  |  |  |  |  |
| drinker | 0.815 | 0.030 | -5.64 | 0.000 | 0.759 | 0.875 |
|  |  |  |  |  |  |  |
| Smoking_status |  |  |  |  |  |  |
| current smoker | 1.600 | 0.066 | 11.32 | 0.000 | 1.475 | 1.735 |
| ex-smoker | 1.092 | 0.041 | 2.37 | 0.018 | 1.015 | 1.175 |
|  |  |  |  |  |  |  |
| BMI_2014_f | 0.975 | 0.003 | -8.85 | 0.000 | 0.970 | 0.981 |
| HT_b | 0.919 | 0.026 | -2.99 | 0.003 | 0.869 | 0.971 |
| Antihypertensives_b | 0.624 | 0.088 | -3.35 | 0.001 | 0.473 | 0.823 |
| Hyperlipidaemia_b | 1.007 | 0.029 | 0.25 | 0.806 | 0.951 | 1.066 |
| Lipid_reg_treatment_b | 0.359 | 0.031 | -11.70 | 0.000 | 0.302 | 0.426 |
|  |  |  |  |  |  |  |
| Diabetes_type_au_b |  |  |  |  |  |  |
| T1DM | 2.951 | 0.483 | 6.62 | 0.000 | 2.142 | 4.066 |
| T2DM | 1.167 | 0.069 | 2.61 | 0.009 | 1.040 | 1.311 |
| DM - nos | 2.199 | 0.253 | 6.86 | 0.000 | 1.756 | 2.755 |
|  |  |  |  |  |  |  |
| Antidiabetics_b | 1.721 | 0.108 | 8.66 | 0.000 | 1.522 | 1.945 |
| Diuretics_b | 2.619 | 0.084 | 29.88 | 0.000 | 2.459 | 2.790 |
| Anticoagulants_b | 1.335 | 0.055 | 6.98 | 0.000 | 1.231 | 1.447 |
| Antiplatelets_b | 0.677 | 0.063 | -4.22 | 0.000 | 0.565 | 0.812 |
| HF_b | 7.855 | 0.281 | 57.53 | 0.000 | 7.322 | 8.426 |
| AF_b | 1.598 | 0.064 | 11.75 | 0.000 | 1.478 | 1.728 |
| Heart_valve_dis_b | 1.558 | 0.165 | 4.20 | 0.000 | 1.267 | 1.917 |
| VT_VF_b | 2.001 | 0.210 | 6.62 | 0.000 | 1.629 | 2.457 |
| Cardiomyopathy_b | 1.725 | 0.189 | 4.97 | 0.000 | 1.391 | 2.138 |
| CV_procedures_b | 0.804 | 0.022 | -7.96 | 0.000 | 0.762 | 0.848 |
| TIA_stroke_b | 4.788 | 0.175 | 42.85 | 0.000 | 4.457 | 5.143 |
| PVD_b | 1.707 | 0.079 | 11.55 | 0.000 | 1.559 | 1.870 |
| CKD_b | 1.475 | 0.044 | 13.14 | 0.000 | 1.392 | 1.563 |
| Hypothyroidism_b | 0.993 | 0.043 | -0.17 | 0.863 | 0.912 | 1.080 |
| Liver_dis_b | 1.874 | 0.461 | 2.56 | 0.011 | 1.158 | 3.034 |
| Lupus_b | 1.320 | 0.294 | 1.24 | 0.214 | 0.852 | 2.043 |
| Erectile_dysfunction_b | 0.894 | 0.035 | -2.84 | 0.005 | 0.828 | 0.966 |
| Any_tumour_b | 1.498 | 0.048 | 12.53 | 0.000 | 1.406 | 1.596 |
| Menopause_b | 0.753 | 0.036 | -5.96 | 0.000 | 0.685 | 0.826 |
| Dementia_b | 3.864 | 0.258 | 20.22 | 0.000 | 3.390 | 4.405 |
| RA_b | 1.172 | 0.077 | 2.42 | 0.015 | 1.031 | 1.333 |
| num_ami_before_31_dec_14 | 0.999 | 0.001 | -0.49 | 0.627 | 0.997 | 1.002 |
| _cons | 0.006 | 0.001 | -24.92 | 0.000 | 0.004 | 0.009 |

**Performance in validation dataset (CPRD GOLD) - External validation - audit AMI cases**

| AUROC (95% CI): 0.826 (0.816; 0.837) |  |
| --- | --- |
|  |  |
|  | % Correctly classified: 77.39 |

### 9) 1-year Composite 2 CV outcome (HF, stroke, & CV mortality)

|  | | | | | | |
| --- | --- | --- | --- | --- | --- | --- |
| _1yr_Comp2_CV_outcome | Odds ratio | Std. err. | z | P>|z| | [95% conf. interval] | |
| age | 1.047 | 0.002 | 29.38 | 0.000 | 1.044 | 1.050 |
|  |  |  |  |  |  |  |
| gender |  |  |  |  |  |  |
| Female | 1.007 | 0.035 | 0.20 | 0.843 | 0.940 | 1.078 |
|  |  |  |  |  |  |  |
| IMD |  |  |  |  |  |  |
| Q2 | 1.184 | 0.051 | 3.89 | 0.000 | 1.087 | 1.289 |
| Q3 | 1.209 | 0.053 | 4.34 | 0.000 | 1.110 | 1.318 |
| Q4 | 1.277 | 0.056 | 5.57 | 0.000 | 1.172 | 1.392 |
| Q5-most deprived | 1.350 | 0.060 | 6.74 | 0.000 | 1.237 | 1.473 |
|  |  |  |  |  |  |  |
| ethnicity2 |  |  |  |  |  |  |
| non-White | 1.028 | 0.056 | 0.51 | 0.612 | 0.924 | 1.144 |
|  |  |  |  |  |  |  |
| Alcohol_status3 |  |  |  |  |  |  |
| drinker | 0.788 | 0.029 | -6.49 | 0.000 | 0.734 | 0.847 |
|  |  |  |  |  |  |  |
| Smoking_status |  |  |  |  |  |  |
| current smoker | 1.367 | 0.058 | 7.34 | 0.000 | 1.257 | 1.486 |
| ex-smoker | 1.064 | 0.041 | 1.63 | 0.104 | 0.987 | 1.147 |
|  |  |  |  |  |  |  |
| BMI_2014_f | 0.995 | 0.003 | -1.66 | 0.097 | 0.990 | 1.001 |
| HT_b | 0.977 | 0.028 | -0.81 | 0.417 | 0.923 | 1.034 |
| Antihypertensives_b | 0.480 | 0.071 | -4.94 | 0.000 | 0.358 | 0.642 |
| Hyperlipidaemia_b | 1.073 | 0.032 | 2.39 | 0.017 | 1.013 | 1.137 |
| Lipid_reg_treatment_b | 0.755 | 0.065 | -3.27 | 0.001 | 0.637 | 0.893 |
|  |  |  |  |  |  |  |
| Diabetes_type_au_b |  |  |  |  |  |  |
| T1DM | 2.042 | 0.341 | 4.27 | 0.000 | 1.472 | 2.833 |
| T2DM | 1.107 | 0.067 | 1.67 | 0.094 | 0.983 | 1.246 |
| DM - nos | 1.559 | 0.178 | 3.88 | 0.000 | 1.245 | 1.951 |
|  |  |  |  |  |  |  |
| Antidiabetics_b | 1.600 | 0.102 | 7.39 | 0.000 | 1.413 | 1.813 |
| Diuretics_b | 2.561 | 0.089 | 26.99 | 0.000 | 2.392 | 2.742 |
| Anticoagulants_b | 1.316 | 0.054 | 6.70 | 0.000 | 1.215 | 1.426 |
| Antiplatelets_b | 0.673 | 0.062 | -4.29 | 0.000 | 0.562 | 0.807 |
| HF_b | 7.923 | 0.265 | 61.85 | 0.000 | 7.420 | 8.460 |
| AF_b | 1.646 | 0.065 | 12.65 | 0.000 | 1.524 | 1.778 |
| Heart_valve_dis_b | 1.575 | 0.161 | 4.45 | 0.000 | 1.289 | 1.924 |
| VT_VF_b | 1.706 | 0.177 | 5.14 | 0.000 | 1.392 | 2.091 |
| Cardiomyopathy_b | 1.839 | 0.195 | 5.75 | 0.000 | 1.494 | 2.263 |
| CV_procedures_b | 0.871 | 0.025 | -4.86 | 0.000 | 0.824 | 0.921 |
| TIA_stroke_b | 4.441 | 0.156 | 42.56 | 0.000 | 4.146 | 4.757 |
| PVD_b | 1.609 | 0.074 | 10.28 | 0.000 | 1.469 | 1.761 |
| CKD_b | 1.375 | 0.042 | 10.52 | 0.000 | 1.296 | 1.459 |
| Hypothyroidism_b | 1.099 | 0.048 | 2.16 | 0.031 | 1.009 | 1.197 |
| Liver_dis_b | 2.049 | 0.497 | 2.96 | 0.003 | 1.273 | 3.296 |
| Lupus_b | 1.817 | 0.389 | 2.79 | 0.005 | 1.194 | 2.764 |
| Erectile_dysfunction_b | 0.998 | 0.040 | -0.04 | 0.964 | 0.922 | 1.081 |
| Any_tumour_b | 1.124 | 0.038 | 3.49 | 0.000 | 1.053 | 1.201 |
| Menopause_b | 0.784 | 0.039 | -4.92 | 0.000 | 0.712 | 0.864 |
| Dementia_b | 1.366 | 0.085 | 5.04 | 0.000 | 1.210 | 1.542 |
| RA_b | 1.193 | 0.079 | 2.65 | 0.008 | 1.047 | 1.359 |
| num_ami_before_31_dec_14 | 1.002 | 0.001 | 1.42 | 0.155 | 0.999 | 1.005 |
| _cons | 0.007 | 0.001 | -22.98 | 0.000 | 0.004 | 0.010 |

**Performance in validation dataset (CPRD GOLD) - External validation - audit AMI cases**

| AUROC (95% CI): 0.776 (0.761; 0.790) |  |
| --- | --- |
|  |  |
|  | % Correctly classified: 76.67 |

## Internal validation (CPRD Aurum) - without therapies

### 1) 5-year all-cause mortality

|  | | | | | | |
| --- | --- | --- | --- | --- | --- | --- |
| _5year_All_cause_death | Odds ratio | Std. err. | z | P>|z| | [95% conf. interval] | |
| age | 1.100 | 0.002 | 63.67 | 0.000 | 1.097 | 1.103 |
|  |  |  |  |  |  |  |
| gender |  |  |  |  |  |  |
| Female | 0.970 | 0.030 | -0.96 | 0.338 | 0.913 | 1.032 |
|  |  |  |  |  |  |  |
| IMD |  |  |  |  |  |  |
| Q2 | 1.284 | 0.049 | 6.50 | 0.000 | 1.191 | 1.385 |
| Q3 | 1.389 | 0.054 | 8.41 | 0.000 | 1.287 | 1.500 |
| Q4 | 1.496 | 0.059 | 10.23 | 0.000 | 1.385 | 1.616 |
| Q5-most deprived | 1.649 | 0.066 | 12.43 | 0.000 | 1.524 | 1.784 |
|  |  |  |  |  |  |  |
| ethnicity2 | 0.721 | 0.038 | -6.21 | 0.000 | 0.650 | 0.799 |
| Alcohol_status3 | 0.810 | 0.027 | -6.22 | 0.000 | 0.758 | 0.866 |
|  |  |  |  |  |  |  |
| Smoking_status |  |  |  |  |  |  |
| current smoker | 1.930 | 0.075 | 16.86 | 0.000 | 1.788 | 2.083 |
| ex-smoker | 1.208 | 0.042 | 5.44 | 0.000 | 1.129 | 1.293 |
|  |  |  |  |  |  |  |
| BMI_2014_f | 0.974 | 0.003 | -9.71 | 0.000 | 0.969 | 0.979 |
| HT_b | 1.049 | 0.027 | 1.89 | 0.058 | 0.998 | 1.103 |
| Hyperlipidaemia_b | 0.968 | 0.026 | -1.22 | 0.221 | 0.918 | 1.020 |
|  |  |  |  |  |  |  |
| Diabetes_type_au_b |  |  |  |  |  |  |
| T1DM | 4.458 | 0.666 | 10.00 | 0.000 | 3.325 | 5.975 |
| T2DM | 1.761 | 0.054 | 18.51 | 0.000 | 1.659 | 1.870 |
| DM - nos | 2.659 | 0.268 | 9.71 | 0.000 | 2.183 | 3.240 |
|  |  |  |  |  |  |  |
| HF_b | 1.921 | 0.062 | 20.13 | 0.000 | 1.803 | 2.047 |
| AF_b | 1.445 | 0.046 | 11.47 | 0.000 | 1.357 | 1.538 |
| Heart_valve_dis_b | 1.656 | 0.161 | 5.17 | 0.000 | 1.368 | 2.005 |
| VT_VF_b | 1.354 | 0.137 | 3.00 | 0.003 | 1.111 | 1.651 |
| Cardiomyopathy_b | 1.706 | 0.177 | 5.15 | 0.000 | 1.392 | 2.091 |
| CV_procedures_b | 0.793 | 0.020 | -9.22 | 0.000 | 0.754 | 0.833 |
| TIA_stroke_b | 1.546 | 0.053 | 12.73 | 0.000 | 1.446 | 1.653 |
| PVD_b | 1.757 | 0.075 | 13.19 | 0.000 | 1.616 | 1.910 |
| CKD_b | 1.542 | 0.042 | 15.90 | 0.000 | 1.462 | 1.627 |
| Hypothyroidism_b | 0.962 | 0.039 | -0.97 | 0.331 | 0.889 | 1.040 |
| Liver_dis_b | 1.505 | 0.373 | 1.65 | 0.099 | 0.926 | 2.445 |
| Lupus_b | 1.647 | 0.342 | 2.40 | 0.016 | 1.097 | 2.473 |
| Erectile_dysfunction_b | 0.851 | 0.031 | -4.39 | 0.000 | 0.792 | 0.915 |
| Any_tumour_b | 1.482 | 0.043 | 13.43 | 0.000 | 1.400 | 1.570 |
| Menopause_b | 0.766 | 0.034 | -5.96 | 0.000 | 0.701 | 0.836 |
| Dementia_b | 4.265 | 0.280 | 22.11 | 0.000 | 3.750 | 4.850 |
| RA_b | 1.247 | 0.076 | 3.65 | 0.000 | 1.108 | 1.404 |
| num_ami_before_31_dec_14 | 0.996 | 0.001 | -3.40 | 0.001 | 0.993 | 0.998 |
| _cons | 0.000 | 0.000 | -52.34 | 0.000 | 0.000 | 0.000 |

**Performance in validation dataset (20% of practices) - Internal validation - audit AMI cases**

| AUROC (95% CI): 0.851 (0.844; 0.858) |  |
| --- | --- |
|  |  |
|  | % Correctly classified: 79.14 |

### 2) 5-year Stroke

|  | | | | | | |
| --- | --- | --- | --- | --- | --- | --- |
| _5year_Stroke | Odds ratio | Std. err. | z | P>|z| | [95% conf. interval] | |
| age | 1.068 | 0.002 | 44.30 | 0.000 | 1.064 | 1.071 |
|  |  |  |  |  |  |  |
| gender |  |  |  |  |  |  |
| Female | 1.161 | 0.038 | 4.58 | 0.000 | 1.089 | 1.238 |
|  |  |  |  |  |  |  |
| IMD |  |  |  |  |  |  |
| Q2 | 1.175 | 0.047 | 4.03 | 0.000 | 1.086 | 1.271 |
| Q3 | 1.119 | 0.046 | 2.75 | 0.006 | 1.033 | 1.213 |
| Q4 | 1.204 | 0.049 | 4.53 | 0.000 | 1.111 | 1.304 |
| Q5-most deprived | 1.283 | 0.053 | 5.97 | 0.000 | 1.182 | 1.392 |
|  |  |  |  |  |  |  |
| ethnicity2 | 1.074 | 0.056 | 1.36 | 0.174 | 0.969 | 1.190 |
| Alcohol_status3 | 1.013 | 0.036 | 0.36 | 0.721 | 0.945 | 1.085 |
|  |  |  |  |  |  |  |
| Smoking_status |  |  |  |  |  |  |
| current smoker | 1.447 | 0.058 | 9.19 | 0.000 | 1.337 | 1.565 |
| ex-smoker | 1.101 | 0.040 | 2.69 | 0.007 | 1.026 | 1.181 |
|  |  |  |  |  |  |  |
| BMI_2014_f | 0.985 | 0.003 | -5.53 | 0.000 | 0.980 | 0.990 |
| HT_b | 1.174 | 0.031 | 6.05 | 0.000 | 1.114 | 1.236 |
| Hyperlipidaemia_b | 1.137 | 0.031 | 4.65 | 0.000 | 1.077 | 1.201 |
|  |  |  |  |  |  |  |
| Diabetes_type_au_b |  |  |  |  |  |  |
| T1DM | 2.991 | 0.449 | 7.29 | 0.000 | 2.229 | 4.016 |
| T2DM | 1.521 | 0.048 | 13.34 | 0.000 | 1.430 | 1.618 |
| DM - nos | 1.817 | 0.187 | 5.79 | 0.000 | 1.484 | 2.224 |
|  |  |  |  |  |  |  |
| HF_b | 1.285 | 0.044 | 7.39 | 0.000 | 1.202 | 1.373 |
| AF_b | 1.569 | 0.051 | 13.75 | 0.000 | 1.471 | 1.673 |
| Heart_valve_dis_b | 1.415 | 0.138 | 3.55 | 0.000 | 1.168 | 1.713 |
| VT_VF_b | 1.197 | 0.127 | 1.69 | 0.090 | 0.972 | 1.473 |
| Cardiomyopathy_b | 1.216 | 0.135 | 1.76 | 0.078 | 0.978 | 1.511 |
| CV_procedures_b | 0.879 | 0.023 | -4.88 | 0.000 | 0.834 | 0.926 |
| TIA_stroke_b | 7.862 | 0.265 | 61.17 | 0.000 | 7.360 | 8.399 |
| PVD_b | 1.466 | 0.064 | 8.69 | 0.000 | 1.345 | 1.598 |
| CKD_b | 1.345 | 0.039 | 10.31 | 0.000 | 1.271 | 1.422 |
| Hypothyroidism_b | 1.043 | 0.043 | 1.02 | 0.309 | 0.962 | 1.130 |
| Liver_dis_b | 2.453 | 0.562 | 3.92 | 0.000 | 1.565 | 3.843 |
| Lupus_b | 1.331 | 0.290 | 1.31 | 0.189 | 0.869 | 2.040 |
| Erectile_dysfunction_b | 1.058 | 0.040 | 1.47 | 0.142 | 0.981 | 1.140 |
| Any_tumour_b | 1.075 | 0.034 | 2.31 | 0.021 | 1.011 | 1.143 |
| Menopause_b | 0.945 | 0.043 | -1.24 | 0.215 | 0.864 | 1.033 |
| Dementia_b | 3.438 | 0.203 | 20.88 | 0.000 | 3.062 | 3.861 |
| RA_b | 1.091 | 0.070 | 1.35 | 0.176 | 0.962 | 1.237 |
| num_ami_before_31_dec_14 | 0.997 | 0.001 | -2.35 | 0.019 | 0.994 | 0.999 |
| _cons | 0.001 | 0.000 | -42.92 | 0.000 | 0.001 | 0.001 |

**Performance in validation dataset (20% of practices) - Internal validation - audit AMI cases**

| AUROC (95% CI): 0.834 (0.826; 0.843) |  |
| --- | --- |
|  |  |
|  | % Correctly classified: 80.18 |

### 3) 5-year heart failure (HF)

|  | | | | | | |
| --- | --- | --- | --- | --- | --- | --- |
| _5year_HF | Odds ratio | Std. err. | z | P>|z| | [95% conf. interval] | |
| age | 1.065 | 0.001 | 47.91 | 0.000 | 1.062 | 1.067 |
|  |  |  |  |  |  |  |
| gender |  |  |  |  |  |  |
| Female | 1.010 | 0.031 | 0.32 | 0.750 | 0.951 | 1.072 |
|  |  |  |  |  |  |  |
| IMD |  |  |  |  |  |  |
| Q2 | 1.189 | 0.044 | 4.70 | 0.000 | 1.106 | 1.278 |
| Q3 | 1.302 | 0.049 | 7.07 | 0.000 | 1.210 | 1.400 |
| Q4 | 1.361 | 0.051 | 8.23 | 0.000 | 1.265 | 1.465 |
| Q5-most deprived | 1.471 | 0.056 | 10.13 | 0.000 | 1.365 | 1.585 |
|  |  |  |  |  |  |  |
| ethnicity2 | 1.170 | 0.054 | 3.37 | 0.001 | 1.068 | 1.282 |
| Alcohol_status3 | 0.830 | 0.027 | -5.73 | 0.000 | 0.778 | 0.884 |
|  |  |  |  |  |  |  |
| Smoking_status |  |  |  |  |  |  |
| current smoker | 1.521 | 0.056 | 11.41 | 0.000 | 1.415 | 1.635 |
| ex-smoker | 1.204 | 0.040 | 5.60 | 0.000 | 1.128 | 1.285 |
|  |  |  |  |  |  |  |
| BMI_2014_f | 1.018 | 0.003 | 7.09 | 0.000 | 1.013 | 1.023 |
| HT_b | 1.191 | 0.029 | 7.21 | 0.000 | 1.136 | 1.249 |
| Hyperlipidaemia_b | 0.983 | 0.025 | -0.68 | 0.499 | 0.935 | 1.034 |
|  |  |  |  |  |  |  |
| Diabetes_type_au_b |  |  |  |  |  |  |
| T1DM | 4.806 | 0.659 | 11.45 | 0.000 | 3.673 | 6.288 |
| T2DM | 1.650 | 0.048 | 17.21 | 0.000 | 1.558 | 1.746 |
| DM - nos | 2.885 | 0.284 | 10.75 | 0.000 | 2.378 | 3.500 |
|  |  |  |  |  |  |  |
| HF_b | 10.212 | 0.358 | 66.22 | 0.000 | 9.534 | 10.939 |
| AF_b | 2.248 | 0.072 | 25.23 | 0.000 | 2.111 | 2.394 |
| Heart_valve_dis_b | 1.715 | 0.174 | 5.30 | 0.000 | 1.405 | 2.093 |
| VT_VF_b | 1.687 | 0.166 | 5.31 | 0.000 | 1.391 | 2.047 |
| Cardiomyopathy_b | 2.410 | 0.267 | 7.94 | 0.000 | 1.940 | 2.994 |
| CV_procedures_b | 0.951 | 0.023 | -2.08 | 0.037 | 0.908 | 0.997 |
| TIA_stroke_b | 1.525 | 0.052 | 12.38 | 0.000 | 1.426 | 1.630 |
| PVD_b | 1.672 | 0.071 | 12.03 | 0.000 | 1.538 | 1.818 |
| CKD_b | 1.564 | 0.042 | 16.56 | 0.000 | 1.483 | 1.649 |
| Hypothyroidism_b | 1.007 | 0.040 | 0.18 | 0.861 | 0.932 | 1.088 |
| Liver_dis_b | 0.970 | 0.232 | -0.13 | 0.898 | 0.607 | 1.551 |
| Lupus_b | 1.649 | 0.315 | 2.62 | 0.009 | 1.134 | 2.397 |
| Erectile_dysfunction_b | 0.981 | 0.033 | -0.58 | 0.564 | 0.918 | 1.048 |
| Any_tumour_b | 1.182 | 0.035 | 5.64 | 0.000 | 1.115 | 1.252 |
| Menopause_b | 0.848 | 0.036 | -3.83 | 0.000 | 0.780 | 0.923 |
| Dementia_b | 2.201 | 0.132 | 13.12 | 0.000 | 1.956 | 2.476 |
| RA_b | 1.413 | 0.082 | 5.96 | 0.000 | 1.261 | 1.583 |
| num_ami_before_31_dec_14 | 1.000 | 0.001 | 0.38 | 0.702 | 0.998 | 1.003 |
| _cons | 0.001 | 0.000 | -49.49 | 0.000 | 0.001 | 0.001 |

**Performance in validation dataset (20% of practices) - Internal validation - audit AMI cases**

| AUROC (95% CI): 0.846 (0.839; 0.854) |  |
| --- | --- |
|  |  |
|  | % Correctly classified: 78.75 |

### 4) 5-year Recurrent MI

|  | | | | | | |
| --- | --- | --- | --- | --- | --- | --- |
| _5year_Recurrent_MI | Odds ratio | Std. err. | z | P>|z| | [95% conf. interval] | |
| age | 1.033 | 0.002 | 22.17 | 0.000 | 1.030 | 1.036 |
|  |  |  |  |  |  |  |
| gender |  |  |  |  |  |  |
| Female | 1.087 | 0.037 | 2.44 | 0.015 | 1.017 | 1.163 |
|  |  |  |  |  |  |  |
| IMD |  |  |  |  |  |  |
| Q2 | 1.112 | 0.048 | 2.46 | 0.014 | 1.022 | 1.210 |
| Q3 | 1.186 | 0.051 | 3.94 | 0.000 | 1.090 | 1.291 |
| Q4 | 1.233 | 0.053 | 4.84 | 0.000 | 1.133 | 1.342 |
| Q5-most deprived | 1.280 | 0.056 | 5.65 | 0.000 | 1.175 | 1.395 |
|  |  |  |  |  |  |  |
| ethnicity2 | 1.237 | 0.062 | 4.25 | 0.000 | 1.121 | 1.364 |
| Alcohol_status3 | 0.878 | 0.031 | -3.62 | 0.000 | 0.819 | 0.942 |
|  |  |  |  |  |  |  |
| Smoking_status |  |  |  |  |  |  |
| current smoker | 1.342 | 0.056 | 7.09 | 0.000 | 1.237 | 1.456 |
| ex-smoker | 1.001 | 0.038 | 0.02 | 0.988 | 0.929 | 1.078 |
|  |  |  |  |  |  |  |
| BMI_2014_f | 0.994 | 0.003 | -2.11 | 0.035 | 0.989 | 1.000 |
| HT_b | 1.170 | 0.033 | 5.61 | 0.000 | 1.107 | 1.235 |
| Hyperlipidaemia_b | 1.110 | 0.032 | 3.61 | 0.000 | 1.049 | 1.174 |
|  |  |  |  |  |  |  |
| Diabetes_type_au_b |  |  |  |  |  |  |
| T1DM | 3.486 | 0.474 | 9.18 | 0.000 | 2.671 | 4.551 |
| T2DM | 1.579 | 0.051 | 14.22 | 0.000 | 1.482 | 1.681 |
| DM - nos | 2.777 | 0.258 | 11.01 | 0.000 | 2.316 | 3.331 |
|  |  |  |  |  |  |  |
| HF_b | 1.314 | 0.046 | 7.82 | 0.000 | 1.227 | 1.408 |
| AF_b | 0.929 | 0.034 | -1.99 | 0.047 | 0.865 | 0.999 |
| Heart_valve_dis_b | 1.478 | 0.145 | 3.99 | 0.000 | 1.220 | 1.791 |
| VT_VF_b | 1.107 | 0.122 | 0.93 | 0.354 | 0.892 | 1.375 |
| Cardiomyopathy_b | 1.251 | 0.141 | 1.98 | 0.047 | 1.003 | 1.562 |
| CV_procedures_b | 0.978 | 0.027 | -0.80 | 0.424 | 0.927 | 1.033 |
| TIA_stroke_b | 1.378 | 0.050 | 8.83 | 0.000 | 1.283 | 1.479 |
| PVD_b | 1.799 | 0.077 | 13.72 | 0.000 | 1.654 | 1.956 |
| CKD_b | 1.457 | 0.044 | 12.37 | 0.000 | 1.373 | 1.547 |
| Hypothyroidism_b | 0.981 | 0.043 | -0.43 | 0.665 | 0.900 | 1.069 |
| Liver_dis_b | 1.039 | 0.277 | 0.14 | 0.887 | 0.615 | 1.753 |
| Lupus_b | 1.167 | 0.256 | 0.70 | 0.483 | 0.758 | 1.794 |
| Erectile_dysfunction_b | 1.006 | 0.039 | 0.16 | 0.870 | 0.932 | 1.086 |
| Any_tumour_b | 1.038 | 0.035 | 1.09 | 0.275 | 0.971 | 1.109 |
| Menopause_b | 0.809 | 0.040 | -4.25 | 0.000 | 0.734 | 0.892 |
| Dementia_b | 2.043 | 0.115 | 12.71 | 0.000 | 1.830 | 2.281 |
| RA_b | 1.388 | 0.088 | 5.17 | 0.000 | 1.226 | 1.572 |
| num_ami_before_31_dec_14 | 1.000 | 0.001 | 0.04 | 0.968 | 0.997 | 1.003 |
| _cons | 0.009 | 0.001 | -29.68 | 0.000 | 0.006 | 0.012 |

**Performance in validation dataset (20% of practices) - Internal validation - audit AMI cases**

| AUROC (95% CI): 0.716 (0.704; 0.729) |  |
| --- | --- |
|  |  |
|  | % Correctly classified: 72.63 |

### 5) 5-year CV mortality

|  | | | | | | |
| --- | --- | --- | --- | --- | --- | --- |
| _5year_CV_death | Odds ratio | Std. err. | z | P>|z| | [95% conf. interval] | |
| age | 1.088 | 0.002 | 43.38 | 0.000 | 1.084 | 1.092 |
|  |  |  |  |  |  |  |
| gender |  |  |  |  |  |  |
| Female | 0.934 | 0.036 | -1.78 | 0.076 | 0.866 | 1.007 |
|  |  |  |  |  |  |  |
| IMD |  |  |  |  |  |  |
| Q2 | 1.250 | 0.062 | 4.49 | 0.000 | 1.134 | 1.378 |
| Q3 | 1.328 | 0.066 | 5.67 | 0.000 | 1.204 | 1.465 |
| Q4 | 1.240 | 0.063 | 4.23 | 0.000 | 1.123 | 1.371 |
| Q5-most deprived | 1.471 | 0.075 | 7.52 | 0.000 | 1.330 | 1.627 |
|  |  |  |  |  |  |  |
| ethnicity2 | 0.847 | 0.057 | -2.48 | 0.013 | 0.742 | 0.966 |
| Alcohol_status3 | 0.900 | 0.038 | -2.53 | 0.011 | 0.829 | 0.976 |
|  |  |  |  |  |  |  |
| Smoking_status |  |  |  |  |  |  |
| current smoker | 1.670 | 0.083 | 10.35 | 0.000 | 1.515 | 1.840 |
| ex-smoker | 1.162 | 0.051 | 3.43 | 0.001 | 1.067 | 1.267 |
|  |  |  |  |  |  |  |
| BMI_2014_f | 0.991 | 0.003 | -2.79 | 0.005 | 0.984 | 0.997 |
| HT_b | 1.087 | 0.035 | 2.58 | 0.010 | 1.020 | 1.159 |
| Hyperlipidaemia_b | 1.083 | 0.036 | 2.36 | 0.018 | 1.014 | 1.156 |
|  |  |  |  |  |  |  |
| Diabetes_type_au_b |  |  |  |  |  |  |
| T1DM | 6.936 | 1.133 | 11.85 | 0.000 | 5.035 | 9.555 |
| T2DM | 1.963 | 0.073 | 18.20 | 0.000 | 1.825 | 2.110 |
| DM - nos | 3.520 | 0.381 | 11.62 | 0.000 | 2.847 | 4.353 |
|  |  |  |  |  |  |  |
| HF_b | 2.616 | 0.094 | 26.90 | 0.000 | 2.439 | 2.806 |
| AF_b | 1.490 | 0.055 | 10.83 | 0.000 | 1.386 | 1.601 |
| Heart_valve_dis_b | 1.822 | 0.188 | 5.83 | 0.000 | 1.489 | 2.229 |
| VT_VF_b | 1.487 | 0.174 | 3.39 | 0.001 | 1.182 | 1.870 |
| Cardiomyopathy_b | 2.595 | 0.284 | 8.72 | 0.000 | 2.094 | 3.215 |
| CV_procedures_b | 0.834 | 0.027 | -5.52 | 0.000 | 0.782 | 0.890 |
| TIA_stroke_b | 1.674 | 0.065 | 13.33 | 0.000 | 1.552 | 1.806 |
| PVD_b | 1.772 | 0.086 | 11.83 | 0.000 | 1.612 | 1.949 |
| CKD_b | 1.501 | 0.050 | 12.19 | 0.000 | 1.406 | 1.602 |
| Hypothyroidism_b | 1.005 | 0.048 | 0.10 | 0.920 | 0.914 | 1.104 |
| Liver_dis_b | 1.374 | 0.452 | 0.96 | 0.335 | 0.721 | 2.618 |
| Lupus_b | 1.337 | 0.358 | 1.08 | 0.279 | 0.790 | 2.261 |
| Erectile_dysfunction_b | 0.891 | 0.043 | -2.41 | 0.016 | 0.811 | 0.979 |
| Any_tumour_b | 0.978 | 0.036 | -0.61 | 0.545 | 0.909 | 1.051 |
| Menopause_b | 0.698 | 0.042 | -6.00 | 0.000 | 0.620 | 0.785 |
| Dementia_b | 3.137 | 0.180 | 19.94 | 0.000 | 2.803 | 3.510 |
| RA_b | 1.176 | 0.090 | 2.12 | 0.034 | 1.012 | 1.367 |
| num_ami_before_31_dec_14 | 0.992 | 0.002 | -4.11 | 0.000 | 0.988 | 0.996 |
| _cons | 0.000 | 0.000 | -44.00 | 0.000 | 0.000 | 0.000 |

**Performance in validation dataset (20% of practices) - Internal validation - audit AMI cases**

| AUROC (95% CI): 0.855 (0.845; 0.865) |  |
| --- | --- |
|  |  |
|  | % Correctly classified: 82.73 |

### 6) 5-year Composite 1 outcome (HF, stroke, recurrent MI, & all-cause mortality)

|  | | | | | | |
| --- | --- | --- | --- | --- | --- | --- |
| _5yr_Comp1_outcome | Odds ratio | Std. err. | z | P>|z| | [95% conf. interval] | |
| age | 1.063 | 0.001 | 53.33 | 0.000 | 1.061 | 1.066 |
|  |  |  |  |  |  |  |
| gender |  |  |  |  |  |  |
| Female | 1.047 | 0.030 | 1.62 | 0.106 | 0.990 | 1.108 |
|  |  |  |  |  |  |  |
| IMD |  |  |  |  |  |  |
| Q2 | 1.191 | 0.039 | 5.30 | 0.000 | 1.117 | 1.271 |
| Q3 | 1.268 | 0.043 | 7.07 | 0.000 | 1.187 | 1.354 |
| Q4 | 1.306 | 0.044 | 7.89 | 0.000 | 1.222 | 1.395 |
| Q5-most deprived | 1.457 | 0.050 | 10.94 | 0.000 | 1.362 | 1.559 |
|  |  |  |  |  |  |  |
| ethnicity2 | 1.079 | 0.045 | 1.81 | 0.070 | 0.994 | 1.171 |
| Alcohol_status3 | 0.869 | 0.027 | -4.55 | 0.000 | 0.818 | 0.923 |
|  |  |  |  |  |  |  |
| Smoking_status |  |  |  |  |  |  |
| current smoker | 1.578 | 0.052 | 13.78 | 0.000 | 1.479 | 1.684 |
| ex-smoker | 1.137 | 0.034 | 4.24 | 0.000 | 1.071 | 1.206 |
|  |  |  |  |  |  |  |
| BMI_2014_f | 1.000 | 0.002 | -0.18 | 0.858 | 0.995 | 1.004 |
| HT_b | 1.186 | 0.026 | 7.70 | 0.000 | 1.136 | 1.239 |
| Hyperlipidaemia_b | 1.025 | 0.024 | 1.06 | 0.291 | 0.979 | 1.074 |
|  |  |  |  |  |  |  |
| Diabetes_type_au_b |  |  |  |  |  |  |
| T1DM | 4.558 | 0.618 | 11.19 | 0.000 | 3.494 | 5.946 |
| T2DM | 1.549 | 0.043 | 15.87 | 0.000 | 1.467 | 1.635 |
| DM - nos | 2.295 | 0.231 | 8.25 | 0.000 | 1.884 | 2.795 |
|  |  |  |  |  |  |  |
| HF_b | 5.108 | 0.188 | 44.32 | 0.000 | 4.753 | 5.490 |
| AF_b | 1.894 | 0.064 | 18.90 | 0.000 | 1.773 | 2.024 |
| Heart_valve_dis_b | 1.501 | 0.161 | 3.79 | 0.000 | 1.216 | 1.852 |
| VT_VF_b | 1.588 | 0.151 | 4.86 | 0.000 | 1.318 | 1.914 |
| Cardiomyopathy_b | 1.884 | 0.213 | 5.61 | 0.000 | 1.510 | 2.351 |
| CV_procedures_b | 0.905 | 0.019 | -4.64 | 0.000 | 0.868 | 0.944 |
| TIA_stroke_b | 3.269 | 0.124 | 31.12 | 0.000 | 3.034 | 3.522 |
| PVD_b | 1.730 | 0.076 | 12.44 | 0.000 | 1.587 | 1.886 |
| CKD_b | 1.476 | 0.040 | 14.48 | 0.000 | 1.400 | 1.556 |
| Hypothyroidism_b | 1.064 | 0.041 | 1.63 | 0.104 | 0.987 | 1.147 |
| Liver_dis_b | 1.568 | 0.321 | 2.20 | 0.028 | 1.050 | 2.342 |
| Lupus_b | 1.575 | 0.284 | 2.52 | 0.012 | 1.105 | 2.244 |
| Erectile_dysfunction_b | 1.002 | 0.030 | 0.06 | 0.955 | 0.944 | 1.063 |
| Any_tumour_b | 1.368 | 0.039 | 10.97 | 0.000 | 1.293 | 1.446 |
| Menopause_b | 0.850 | 0.034 | -4.11 | 0.000 | 0.787 | 0.919 |
| Dementia_b | 4.240 | 0.365 | 16.79 | 0.000 | 3.582 | 5.018 |
| RA_b | 1.325 | 0.074 | 5.06 | 0.000 | 1.188 | 1.478 |
| num_ami_before_31_dec_14 | 0.998 | 0.001 | -2.24 | 0.025 | 0.996 | 1.000 |
| _cons | 0.004 | 0.001 | -44.22 | 0.000 | 0.003 | 0.005 |

**Performance in validation dataset (20% of practices) - Internal validation - audit AMI cases**

| AUROC (95% CI): 0.819 (0.812; 0.825) |  |
| --- | --- |
|  |  |
|  | % Correctly classified: 74.83 |

### 7) 5-year Composite 1 CV outcome (HF, stroke, recurrent MI, & CV mortality)

|  | | | | | | |
| --- | --- | --- | --- | --- | --- | --- |
| _5yr_Comp1_CV_outcome | Odds ratio | Std. err. | z | P>|z| | [95% conf. interval] | |
| age | 1.056 | 0.001 | 47.71 | 0.000 | 1.054 | 1.059 |
|  |  |  |  |  |  |  |
| gender |  |  |  |  |  |  |
| Female | 1.056 | 0.030 | 1.91 | 0.056 | 0.999 | 1.117 |
|  |  |  |  |  |  |  |
| IMD |  |  |  |  |  |  |
| Q2 | 1.108 | 0.037 | 3.09 | 0.002 | 1.038 | 1.183 |
| Q3 | 1.226 | 0.041 | 6.04 | 0.000 | 1.147 | 1.309 |
| Q4 | 1.254 | 0.043 | 6.67 | 0.000 | 1.173 | 1.340 |
| Q5-most deprived | 1.405 | 0.048 | 9.87 | 0.000 | 1.314 | 1.504 |
|  |  |  |  |  |  |  |
| ethnicity2 | 1.174 | 0.049 | 3.83 | 0.000 | 1.081 | 1.274 |
| Alcohol_status3 | 0.892 | 0.027 | -3.75 | 0.000 | 0.840 | 0.947 |
|  |  |  |  |  |  |  |
| Smoking_status |  |  |  |  |  |  |
| current smoker | 1.513 | 0.050 | 12.49 | 0.000 | 1.418 | 1.615 |
| ex-smoker | 1.119 | 0.034 | 3.73 | 0.000 | 1.055 | 1.188 |
|  |  |  |  |  |  |  |
| BMI_2014_f | 1.007 | 0.002 | 3.03 | 0.002 | 1.002 | 1.011 |
| HT_b | 1.221 | 0.027 | 9.01 | 0.000 | 1.169 | 1.275 |
| Hyperlipidaemia_b | 1.040 | 0.024 | 1.67 | 0.096 | 0.993 | 1.089 |
|  |  |  |  |  |  |  |
| Diabetes_type_au_b |  |  |  |  |  |  |
| T1DM | 3.849 | 0.512 | 10.14 | 0.000 | 2.966 | 4.995 |
| T2DM | 1.559 | 0.043 | 16.24 | 0.000 | 1.478 | 1.645 |
| DM - nos | 2.299 | 0.227 | 8.43 | 0.000 | 1.894 | 2.790 |
|  |  |  |  |  |  |  |
| HF_b | 5.934 | 0.213 | 49.50 | 0.000 | 5.530 | 6.368 |
| AF_b | 1.937 | 0.064 | 20.15 | 0.000 | 1.816 | 2.066 |
| Heart_valve_dis_b | 1.552 | 0.162 | 4.21 | 0.000 | 1.265 | 1.904 |
| VT_VF_b | 1.487 | 0.140 | 4.20 | 0.000 | 1.236 | 1.790 |
| Cardiomyopathy_b | 2.084 | 0.234 | 6.53 | 0.000 | 1.672 | 2.597 |
| CV_procedures_b | 0.949 | 0.021 | -2.40 | 0.016 | 0.910 | 0.991 |
| TIA_stroke_b | 3.596 | 0.132 | 34.83 | 0.000 | 3.346 | 3.864 |
| PVD_b | 1.796 | 0.077 | 13.60 | 0.000 | 1.651 | 1.955 |
| CKD_b | 1.466 | 0.039 | 14.49 | 0.000 | 1.392 | 1.544 |
| Hypothyroidism_b | 1.087 | 0.041 | 2.21 | 0.027 | 1.009 | 1.170 |
| Liver_dis_b | 1.287 | 0.267 | 1.21 | 0.225 | 0.857 | 1.933 |
| Lupus_b | 1.592 | 0.286 | 2.58 | 0.010 | 1.119 | 2.265 |
| Erectile_dysfunction_b | 0.994 | 0.030 | -0.20 | 0.843 | 0.937 | 1.055 |
| Any_tumour_b | 1.158 | 0.033 | 5.20 | 0.000 | 1.096 | 1.224 |
| Menopause_b | 0.855 | 0.034 | -3.98 | 0.000 | 0.791 | 0.923 |
| Dementia_b | 3.128 | 0.226 | 15.78 | 0.000 | 2.714 | 3.604 |
| RA_b | 1.267 | 0.070 | 4.28 | 0.000 | 1.137 | 1.412 |
| num_ami_before_31_dec_14 | 0.998 | 0.001 | -1.83 | 0.067 | 0.996 | 1.000 |
| _cons | 0.004 | 0.001 | -43.61 | 0.000 | 0.003 | 0.005 |

**Performance in validation dataset (20% of practices) - Internal validation - audit AMI cases**

| AUROC (95% CI): 0.815 (0.808; 0.822) |  |
| --- | --- |
|  |  |
|  | % Correctly classified: 74.82 |

### 8) 5-year Composite 2 outcome (HF, stroke, & all-cause mortality)

|  | | | | | | |
| --- | --- | --- | --- | --- | --- | --- |
| _5yr_Comp2_outcome | Odds ratio | Std. err. | z | P>|z| | [95% conf. interval] | |
| age | 1.072 | 0.001 | 57.40 | 0.000 | 1.069 | 1.074 |
|  |  |  |  |  |  |  |
| gender |  |  |  |  |  |  |
| Female | 1.065 | 0.031 | 2.16 | 0.031 | 1.006 | 1.128 |
|  |  |  |  |  |  |  |
| IMD |  |  |  |  |  |  |
| Q2 | 1.206 | 0.041 | 5.48 | 0.000 | 1.128 | 1.289 |
| Q3 | 1.279 | 0.044 | 7.10 | 0.000 | 1.195 | 1.369 |
| Q4 | 1.329 | 0.046 | 8.16 | 0.000 | 1.242 | 1.424 |
| Q5-most deprived | 1.512 | 0.054 | 11.64 | 0.000 | 1.410 | 1.621 |
|  |  |  |  |  |  |  |
| ethnicity2 | 1.050 | 0.046 | 1.11 | 0.265 | 0.964 | 1.143 |
| Alcohol_status3 | 0.896 | 0.028 | -3.49 | 0.000 | 0.842 | 0.953 |
|  |  |  |  |  |  |  |
| Smoking_status |  |  |  |  |  |  |
| current smoker | 1.655 | 0.057 | 14.71 | 0.000 | 1.548 | 1.770 |
| ex-smoker | 1.162 | 0.036 | 4.82 | 0.000 | 1.093 | 1.235 |
|  |  |  |  |  |  |  |
| BMI_2014_f | 1.001 | 0.002 | 0.39 | 0.697 | 0.996 | 1.005 |
| HT_b | 1.144 | 0.026 | 5.92 | 0.000 | 1.094 | 1.197 |
| Hyperlipidaemia_b | 1.014 | 0.025 | 0.58 | 0.562 | 0.967 | 1.063 |
|  |  |  |  |  |  |  |
| Diabetes_type_au_b |  |  |  |  |  |  |
| T1DM | 4.564 | 0.623 | 11.12 | 0.000 | 3.493 | 5.964 |
| T2DM | 1.616 | 0.046 | 17.03 | 0.000 | 1.529 | 1.708 |
| DM - nos | 2.479 | 0.253 | 8.91 | 0.000 | 2.030 | 3.027 |
|  |  |  |  |  |  |  |
| HF_b | 5.812 | 0.215 | 47.53 | 0.000 | 5.405 | 6.249 |
| AF_b | 1.994 | 0.068 | 20.30 | 0.000 | 1.866 | 2.132 |
| Heart_valve_dis_b | 1.592 | 0.172 | 4.30 | 0.000 | 1.288 | 1.969 |
| VT_VF_b | 1.597 | 0.155 | 4.84 | 0.000 | 1.321 | 1.931 |
| Cardiomyopathy_b | 2.104 | 0.240 | 6.52 | 0.000 | 1.682 | 2.631 |
| CV_procedures_b | 0.875 | 0.019 | -6.00 | 0.000 | 0.838 | 0.914 |
| TIA_stroke_b | 3.548 | 0.135 | 33.27 | 0.000 | 3.293 | 3.823 |
| PVD_b | 1.716 | 0.076 | 12.20 | 0.000 | 1.573 | 1.871 |
| CKD_b | 1.514 | 0.041 | 15.30 | 0.000 | 1.436 | 1.597 |
| Hypothyroidism_b | 1.052 | 0.041 | 1.30 | 0.193 | 0.975 | 1.135 |
| Liver_dis_b | 1.395 | 0.295 | 1.57 | 0.116 | 0.921 | 2.113 |
| Lupus_b | 1.764 | 0.325 | 3.09 | 0.002 | 1.230 | 2.530 |
| Erectile_dysfunction_b | 0.990 | 0.031 | -0.33 | 0.739 | 0.931 | 1.052 |
| Any_tumour_b | 1.360 | 0.039 | 10.63 | 0.000 | 1.285 | 1.440 |
| Menopause_b | 0.827 | 0.033 | -4.70 | 0.000 | 0.764 | 0.895 |
| Dementia_b | 4.363 | 0.370 | 17.37 | 0.000 | 3.694 | 5.152 |
| RA_b | 1.348 | 0.076 | 5.26 | 0.000 | 1.206 | 1.506 |
| num_ami_before_31_dec_14 | 0.997 | 0.001 | -2.56 | 0.011 | 0.995 | 0.999 |
| _cons | 0.002 | 0.000 | -48.63 | 0.000 | 0.001 | 0.002 |

**Performance in validation dataset (20% of practices) - Internal validation - audit AMI cases**

| AUROC (95% CI): 0.840 (0.833; 0.847) |  |
| --- | --- |
|  |  |
|  | % Correctly classified: 76.79 |

### 9) 5-year Composite 2 CV outcome (HF, stroke, & CV mortality)

|  | | | | | | |
| --- | --- | --- | --- | --- | --- | --- |
| _5yr_Comp2_CV_outcome | Odds ratio | Std. err. | z | P>|z| | [95% conf. interval] | |
| age | 1.066 | 0.001 | 52.58 | 0.000 | 1.063 | 1.068 |
|  |  |  |  |  |  |  |
| gender |  |  |  |  |  |  |
| Female | 1.087 | 0.032 | 2.85 | 0.004 | 1.026 | 1.151 |
|  |  |  |  |  |  |  |
| IMD |  |  |  |  |  |  |
| Q2 | 1.134 | 0.039 | 3.67 | 0.000 | 1.061 | 1.214 |
| Q3 | 1.221 | 0.043 | 5.73 | 0.000 | 1.141 | 1.308 |
| Q4 | 1.255 | 0.044 | 6.46 | 0.000 | 1.171 | 1.344 |
| Q5-most deprived | 1.419 | 0.051 | 9.80 | 0.000 | 1.323 | 1.522 |
|  |  |  |  |  |  |  |
| ethnicity2 | 1.114 | 0.049 | 2.46 | 0.014 | 1.022 | 1.213 |
| Alcohol_status3 | 0.892 | 0.028 | -3.64 | 0.000 | 0.838 | 0.948 |
|  |  |  |  |  |  |  |
| Smoking_status |  |  |  |  |  |  |
| current smoker | 1.508 | 0.052 | 11.94 | 0.000 | 1.410 | 1.613 |
| ex-smoker | 1.132 | 0.035 | 3.96 | 0.000 | 1.064 | 1.203 |
|  |  |  |  |  |  |  |
| BMI_2014_f | 1.007 | 0.002 | 3.10 | 0.002 | 1.003 | 1.012 |
| HT_b | 1.185 | 0.027 | 7.41 | 0.000 | 1.133 | 1.239 |
| Hyperlipidaemia_b | 1.019 | 0.025 | 0.77 | 0.440 | 0.972 | 1.069 |
|  |  |  |  |  |  |  |
| Diabetes_type_au_b |  |  |  |  |  |  |
| T1DM | 4.326 | 0.585 | 10.84 | 0.000 | 3.319 | 5.638 |
| T2DM | 1.552 | 0.044 | 15.63 | 0.000 | 1.469 | 1.640 |
| DM - nos | 2.606 | 0.262 | 9.53 | 0.000 | 2.140 | 3.173 |
|  |  |  |  |  |  |  |
| HF_b | 6.681 | 0.241 | 52.55 | 0.000 | 6.224 | 7.171 |
| AF_b | 2.053 | 0.068 | 21.72 | 0.000 | 1.924 | 2.191 |
| Heart_valve_dis_b | 1.612 | 0.170 | 4.54 | 0.000 | 1.312 | 1.982 |
| VT_VF_b | 1.707 | 0.165 | 5.55 | 0.000 | 1.413 | 2.062 |
| Cardiomyopathy_b | 2.149 | 0.242 | 6.80 | 0.000 | 1.724 | 2.678 |
| CV_procedures_b | 0.906 | 0.020 | -4.41 | 0.000 | 0.867 | 0.946 |
| TIA_stroke_b | 3.939 | 0.145 | 37.22 | 0.000 | 3.665 | 4.234 |
| PVD_b | 1.718 | 0.074 | 12.48 | 0.000 | 1.578 | 1.870 |
| CKD_b | 1.481 | 0.040 | 14.68 | 0.000 | 1.406 | 1.561 |
| Hypothyroidism_b | 1.027 | 0.039 | 0.70 | 0.485 | 0.953 | 1.108 |
| Liver_dis_b | 1.452 | 0.309 | 1.75 | 0.080 | 0.956 | 2.204 |
| Lupus_b | 1.785 | 0.328 | 3.15 | 0.002 | 1.245 | 2.560 |
| Erectile_dysfunction_b | 1.021 | 0.032 | 0.65 | 0.518 | 0.960 | 1.086 |
| Any_tumour_b | 1.170 | 0.034 | 5.45 | 0.000 | 1.106 | 1.238 |
| Menopause_b | 0.843 | 0.034 | -4.20 | 0.000 | 0.779 | 0.913 |
| Dementia_b | 3.000 | 0.213 | 15.48 | 0.000 | 2.611 | 3.448 |
| RA_b | 1.331 | 0.075 | 5.06 | 0.000 | 1.191 | 1.487 |
| num_ami_before_31_dec_14 | 0.998 | 0.001 | -1.54 | 0.123 | 0.996 | 1.000 |
| _cons | 0.002 | 0.000 | -47.70 | 0.000 | 0.001 | 0.002 |

**Performance in validation dataset (20% of practices) - Internal validation - audit AMI cases**

| AUROC (95% CI): 0.834 (0.828; 0.841) |  |
| --- | --- |
|  |  |
|  | % Correctly classified: 76.45 |

## External validation (CPRD GOLD) - without therapies

### 1) 5-year all-cause mortality

|  | | | | | | |
| --- | --- | --- | --- | --- | --- | --- |
| _5year_All_cause_death | Odds ratio | Std. err. | z | P>|z| | [95% conf. interval] | |
| age | 1.100 | 0.002 | 63.67 | 0.000 | 1.097 | 1.103 |
|  |  |  |  |  |  |  |
| gender |  |  |  |  |  |  |
| Female | 0.970 | 0.030 | -0.96 | 0.338 | 0.913 | 1.032 |
|  |  |  |  |  |  |  |
| IMD |  |  |  |  |  |  |
| Q2 | 1.284 | 0.049 | 6.50 | 0.000 | 1.191 | 1.385 |
| Q3 | 1.389 | 0.054 | 8.41 | 0.000 | 1.287 | 1.500 |
| Q4 | 1.496 | 0.059 | 10.23 | 0.000 | 1.385 | 1.616 |
| Q5-most deprived | 1.649 | 0.066 | 12.43 | 0.000 | 1.524 | 1.784 |
|  |  |  |  |  |  |  |
| ethnicity2 | 0.721 | 0.038 | -6.21 | 0.000 | 0.650 | 0.799 |
| Alcohol_status3 | 0.810 | 0.027 | -6.22 | 0.000 | 0.758 | 0.866 |
|  |  |  |  |  |  |  |
| Smoking_status |  |  |  |  |  |  |
| current smoker | 1.930 | 0.075 | 16.86 | 0.000 | 1.788 | 2.083 |
| ex-smoker | 1.208 | 0.042 | 5.44 | 0.000 | 1.129 | 1.293 |
|  |  |  |  |  |  |  |
| BMI_2014_f | 0.974 | 0.003 | -9.71 | 0.000 | 0.969 | 0.979 |
| HT_b | 1.049 | 0.027 | 1.89 | 0.058 | 0.998 | 1.103 |
| Hyperlipidaemia_b | 0.968 | 0.026 | -1.22 | 0.221 | 0.918 | 1.020 |
|  |  |  |  |  |  |  |
| Diabetes_type_au_b |  |  |  |  |  |  |
| T1DM | 4.458 | 0.666 | 10.00 | 0.000 | 3.325 | 5.975 |
| T2DM | 1.761 | 0.054 | 18.51 | 0.000 | 1.659 | 1.870 |
| DM - nos | 2.659 | 0.268 | 9.71 | 0.000 | 2.183 | 3.240 |
|  |  |  |  |  |  |  |
| HF_b | 1.921 | 0.062 | 20.13 | 0.000 | 1.803 | 2.047 |
| AF_b | 1.445 | 0.046 | 11.47 | 0.000 | 1.357 | 1.538 |
| Heart_valve_dis_b | 1.656 | 0.161 | 5.17 | 0.000 | 1.368 | 2.005 |
| VT_VF_b | 1.354 | 0.137 | 3.00 | 0.003 | 1.111 | 1.651 |
| Cardiomyopathy_b | 1.706 | 0.177 | 5.15 | 0.000 | 1.392 | 2.091 |
| CV_procedures_b | 0.793 | 0.020 | -9.22 | 0.000 | 0.754 | 0.833 |
| TIA_stroke_b | 1.546 | 0.053 | 12.73 | 0.000 | 1.446 | 1.653 |
| PVD_b | 1.757 | 0.075 | 13.19 | 0.000 | 1.616 | 1.910 |
| CKD_b | 1.542 | 0.042 | 15.90 | 0.000 | 1.462 | 1.627 |
| Hypothyroidism_b | 0.962 | 0.039 | -0.97 | 0.331 | 0.889 | 1.040 |
| Liver_dis_b | 1.505 | 0.373 | 1.65 | 0.099 | 0.926 | 2.445 |
| Lupus_b | 1.647 | 0.342 | 2.40 | 0.016 | 1.097 | 2.473 |
| Erectile_dysfunction_b | 0.851 | 0.031 | -4.39 | 0.000 | 0.792 | 0.915 |
| Any_tumour_b | 1.482 | 0.043 | 13.43 | 0.000 | 1.400 | 1.570 |
| Menopause_b | 0.766 | 0.034 | -5.96 | 0.000 | 0.701 | 0.836 |
| Dementia_b | 4.265 | 0.280 | 22.11 | 0.000 | 3.750 | 4.850 |
| RA_b | 1.247 | 0.076 | 3.65 | 0.000 | 1.108 | 1.404 |
| num_ami_before_31_dec_14 | 0.996 | 0.001 | -3.40 | 0.001 | 0.993 | 0.998 |
| _cons | 0.000 | 0.000 | -52.34 | 0.000 | 0.000 | 0.000 |

**Performance in validation dataset (CPRD GOLD) - External validation - audit AMI cases**

| AUROC (95% CI): 0.840 (0.830; 0.849) |  |
| --- | --- |
|  |  |
|  | % Correctly classified: 77.68 |

### 2) 5-year Stroke

|  | | | | | | |
| --- | --- | --- | --- | --- | --- | --- |
| _5year_Stroke | Odds ratio | Std. err. | z | P>|z| | [95% conf. interval] | |
| age | 1.068 | 0.002 | 44.30 | 0.000 | 1.064 | 1.071 |
|  |  |  |  |  |  |  |
| gender |  |  |  |  |  |  |
| Female | 1.161 | 0.038 | 4.58 | 0.000 | 1.089 | 1.238 |
|  |  |  |  |  |  |  |
| IMD |  |  |  |  |  |  |
| Q2 | 1.175 | 0.047 | 4.03 | 0.000 | 1.086 | 1.271 |
| Q3 | 1.119 | 0.046 | 2.75 | 0.006 | 1.033 | 1.213 |
| Q4 | 1.204 | 0.049 | 4.53 | 0.000 | 1.111 | 1.304 |
| Q5-most deprived | 1.283 | 0.053 | 5.97 | 0.000 | 1.182 | 1.392 |
|  |  |  |  |  |  |  |
| ethnicity2 | 1.074 | 0.056 | 1.36 | 0.174 | 0.969 | 1.190 |
| Alcohol_status3 | 1.013 | 0.036 | 0.36 | 0.721 | 0.945 | 1.085 |
|  |  |  |  |  |  |  |
| Smoking_status |  |  |  |  |  |  |
| current smoker | 1.447 | 0.058 | 9.19 | 0.000 | 1.337 | 1.565 |
| ex-smoker | 1.101 | 0.040 | 2.69 | 0.007 | 1.026 | 1.181 |
|  |  |  |  |  |  |  |
| BMI_2014_f | 0.985 | 0.003 | -5.53 | 0.000 | 0.980 | 0.990 |
| HT_b | 1.174 | 0.031 | 6.05 | 0.000 | 1.114 | 1.236 |
| Hyperlipidaemia_b | 1.137 | 0.031 | 4.65 | 0.000 | 1.077 | 1.201 |
|  |  |  |  |  |  |  |
| Diabetes_type_au_b |  |  |  |  |  |  |
| T1DM | 2.991 | 0.449 | 7.29 | 0.000 | 2.229 | 4.016 |
| T2DM | 1.521 | 0.048 | 13.34 | 0.000 | 1.430 | 1.618 |
| DM - nos | 1.817 | 0.187 | 5.79 | 0.000 | 1.484 | 2.224 |
|  |  |  |  |  |  |  |
| HF_b | 1.285 | 0.044 | 7.39 | 0.000 | 1.202 | 1.373 |
| AF_b | 1.569 | 0.051 | 13.75 | 0.000 | 1.471 | 1.673 |
| Heart_valve_dis_b | 1.415 | 0.138 | 3.55 | 0.000 | 1.168 | 1.713 |
| VT_VF_b | 1.197 | 0.127 | 1.69 | 0.090 | 0.972 | 1.473 |
| Cardiomyopathy_b | 1.216 | 0.135 | 1.76 | 0.078 | 0.978 | 1.511 |
| CV_procedures_b | 0.879 | 0.023 | -4.88 | 0.000 | 0.834 | 0.926 |
| TIA_stroke_b | 7.862 | 0.265 | 61.17 | 0.000 | 7.360 | 8.399 |
| PVD_b | 1.466 | 0.064 | 8.69 | 0.000 | 1.345 | 1.598 |
| CKD_b | 1.345 | 0.039 | 10.31 | 0.000 | 1.271 | 1.422 |
| Hypothyroidism_b | 1.043 | 0.043 | 1.02 | 0.309 | 0.962 | 1.130 |
| Liver_dis_b | 2.453 | 0.562 | 3.92 | 0.000 | 1.565 | 3.843 |
| Lupus_b | 1.331 | 0.290 | 1.31 | 0.189 | 0.869 | 2.040 |
| Erectile_dysfunction_b | 1.058 | 0.040 | 1.47 | 0.142 | 0.981 | 1.140 |
| Any_tumour_b | 1.075 | 0.034 | 2.31 | 0.021 | 1.011 | 1.143 |
| Menopause_b | 0.945 | 0.043 | -1.24 | 0.215 | 0.864 | 1.033 |
| Dementia_b | 3.438 | 0.203 | 20.88 | 0.000 | 3.062 | 3.861 |
| RA_b | 1.091 | 0.070 | 1.35 | 0.176 | 0.962 | 1.237 |
| num_ami_before_31_dec_14 | 0.997 | 0.001 | -2.35 | 0.019 | 0.994 | 0.999 |
| _cons | 0.001 | 0.000 | -42.92 | 0.000 | 0.001 | 0.001 |

**Performance in validation dataset (CPRD GOLD) - External validation - audit AMI cases**

| AUROC (95% CI): 0.816 (0.805; 0.826) |  |
| --- | --- |
|  |  |
|  | % Correctly classified: 77.08 |

### 3) 5-year heart failure (HF)

|  | | | | | | |
| --- | --- | --- | --- | --- | --- | --- |
| _5year_HF | Odds ratio | Std. err. | z | P>|z| | [95% conf. interval] | |
| age | 1.065 | 0.001 | 47.91 | 0.000 | 1.062 | 1.067 |
|  |  |  |  |  |  |  |
| gender |  |  |  |  |  |  |
| Female | 1.010 | 0.031 | 0.32 | 0.750 | 0.951 | 1.072 |
|  |  |  |  |  |  |  |
| IMD |  |  |  |  |  |  |
| Q2 | 1.189 | 0.044 | 4.70 | 0.000 | 1.106 | 1.278 |
| Q3 | 1.302 | 0.049 | 7.07 | 0.000 | 1.210 | 1.400 |
| Q4 | 1.361 | 0.051 | 8.23 | 0.000 | 1.265 | 1.465 |
| Q5-most deprived | 1.471 | 0.056 | 10.13 | 0.000 | 1.365 | 1.585 |
|  |  |  |  |  |  |  |
| ethnicity2 | 1.170 | 0.054 | 3.37 | 0.001 | 1.068 | 1.282 |
| Alcohol_status3 | 0.830 | 0.027 | -5.73 | 0.000 | 0.778 | 0.884 |
|  |  |  |  |  |  |  |
| Smoking_status |  |  |  |  |  |  |
| current smoker | 1.521 | 0.056 | 11.41 | 0.000 | 1.415 | 1.635 |
| ex-smoker | 1.204 | 0.040 | 5.60 | 0.000 | 1.128 | 1.285 |
|  |  |  |  |  |  |  |
| BMI_2014_f | 1.018 | 0.003 | 7.09 | 0.000 | 1.013 | 1.023 |
| HT_b | 1.191 | 0.029 | 7.21 | 0.000 | 1.136 | 1.249 |
| Hyperlipidaemia_b | 0.983 | 0.025 | -0.68 | 0.499 | 0.935 | 1.034 |
|  |  |  |  |  |  |  |
| Diabetes_type_au_b |  |  |  |  |  |  |
| T1DM | 4.806 | 0.659 | 11.45 | 0.000 | 3.673 | 6.288 |
| T2DM | 1.650 | 0.048 | 17.21 | 0.000 | 1.558 | 1.746 |
| DM - nos | 2.885 | 0.284 | 10.75 | 0.000 | 2.378 | 3.500 |
|  |  |  |  |  |  |  |
| HF_b | 10.212 | 0.358 | 66.22 | 0.000 | 9.534 | 10.939 |
| AF_b | 2.248 | 0.072 | 25.23 | 0.000 | 2.111 | 2.394 |
| Heart_valve_dis_b | 1.715 | 0.174 | 5.30 | 0.000 | 1.405 | 2.093 |
| VT_VF_b | 1.687 | 0.166 | 5.31 | 0.000 | 1.391 | 2.047 |
| Cardiomyopathy_b | 2.410 | 0.267 | 7.94 | 0.000 | 1.940 | 2.994 |
| CV_procedures_b | 0.951 | 0.023 | -2.08 | 0.037 | 0.908 | 0.997 |
| TIA_stroke_b | 1.525 | 0.052 | 12.38 | 0.000 | 1.426 | 1.630 |
| PVD_b | 1.672 | 0.071 | 12.03 | 0.000 | 1.538 | 1.818 |
| CKD_b | 1.564 | 0.042 | 16.56 | 0.000 | 1.483 | 1.649 |
| Hypothyroidism_b | 1.007 | 0.040 | 0.18 | 0.861 | 0.932 | 1.088 |
| Liver_dis_b | 0.970 | 0.232 | -0.13 | 0.898 | 0.607 | 1.551 |
| Lupus_b | 1.649 | 0.315 | 2.62 | 0.009 | 1.134 | 2.397 |
| Erectile_dysfunction_b | 0.981 | 0.033 | -0.58 | 0.564 | 0.918 | 1.048 |
| Any_tumour_b | 1.182 | 0.035 | 5.64 | 0.000 | 1.115 | 1.252 |
| Menopause_b | 0.848 | 0.036 | -3.83 | 0.000 | 0.780 | 0.923 |
| Dementia_b | 2.201 | 0.132 | 13.12 | 0.000 | 1.956 | 2.476 |
| RA_b | 1.413 | 0.082 | 5.96 | 0.000 | 1.261 | 1.583 |
| num_ami_before_31_dec_14 | 1.000 | 0.001 | 0.38 | 0.702 | 0.998 | 1.003 |
| _cons | 0.001 | 0.000 | -49.49 | 0.000 | 0.001 | 0.001 |

**Performance in validation dataset (CPRD GOLD) - External validation - audit AMI cases**

| AUROC (95% CI): 0.771 (0.760; 0.782) |  |
| --- | --- |
|  |  |
|  | % Correctly classified: 72.70 |

### 4) 5-year Recurrent MI

|  | | | | | | |
| --- | --- | --- | --- | --- | --- | --- |
| _5year_Recurrent_MI | Odds ratio | Std. err. | z | P>|z| | [95% conf. interval] | |
| age | 1.033 | 0.002 | 22.17 | 0.000 | 1.030 | 1.036 |
|  |  |  |  |  |  |  |
| gender |  |  |  |  |  |  |
| Female | 1.087 | 0.037 | 2.44 | 0.015 | 1.017 | 1.163 |
|  |  |  |  |  |  |  |
| IMD |  |  |  |  |  |  |
| Q2 | 1.112 | 0.048 | 2.46 | 0.014 | 1.022 | 1.210 |
| Q3 | 1.186 | 0.051 | 3.94 | 0.000 | 1.090 | 1.291 |
| Q4 | 1.233 | 0.053 | 4.84 | 0.000 | 1.133 | 1.342 |
| Q5-most deprived | 1.280 | 0.056 | 5.65 | 0.000 | 1.175 | 1.395 |
|  |  |  |  |  |  |  |
| ethnicity2 | 1.237 | 0.062 | 4.25 | 0.000 | 1.121 | 1.364 |
| Alcohol_status3 | 0.878 | 0.031 | -3.62 | 0.000 | 0.819 | 0.942 |
|  |  |  |  |  |  |  |
| Smoking_status |  |  |  |  |  |  |
| current smoker | 1.342 | 0.056 | 7.09 | 0.000 | 1.237 | 1.456 |
| ex-smoker | 1.001 | 0.038 | 0.02 | 0.988 | 0.929 | 1.078 |
|  |  |  |  |  |  |  |
| BMI_2014_f | 0.994 | 0.003 | -2.11 | 0.035 | 0.989 | 1.000 |
| HT_b | 1.170 | 0.033 | 5.61 | 0.000 | 1.107 | 1.235 |
| Hyperlipidaemia_b | 1.110 | 0.032 | 3.61 | 0.000 | 1.049 | 1.174 |
|  |  |  |  |  |  |  |
| Diabetes_type_au_b |  |  |  |  |  |  |
| T1DM | 3.486 | 0.474 | 9.18 | 0.000 | 2.671 | 4.551 |
| T2DM | 1.579 | 0.051 | 14.22 | 0.000 | 1.482 | 1.681 |
| DM - nos | 2.777 | 0.258 | 11.01 | 0.000 | 2.316 | 3.331 |
|  |  |  |  |  |  |  |
| HF_b | 1.314 | 0.046 | 7.82 | 0.000 | 1.227 | 1.408 |
| AF_b | 0.929 | 0.034 | -1.99 | 0.047 | 0.865 | 0.999 |
| Heart_valve_dis_b | 1.478 | 0.145 | 3.99 | 0.000 | 1.220 | 1.791 |
| VT_VF_b | 1.107 | 0.122 | 0.93 | 0.354 | 0.892 | 1.375 |
| Cardiomyopathy_b | 1.251 | 0.141 | 1.98 | 0.047 | 1.003 | 1.562 |
| CV_procedures_b | 0.978 | 0.027 | -0.80 | 0.424 | 0.927 | 1.033 |
| TIA_stroke_b | 1.378 | 0.050 | 8.83 | 0.000 | 1.283 | 1.479 |
| PVD_b | 1.799 | 0.077 | 13.72 | 0.000 | 1.654 | 1.956 |
| CKD_b | 1.457 | 0.044 | 12.37 | 0.000 | 1.373 | 1.547 |
| Hypothyroidism_b | 0.981 | 0.043 | -0.43 | 0.665 | 0.900 | 1.069 |
| Liver_dis_b | 1.039 | 0.277 | 0.14 | 0.887 | 0.615 | 1.753 |
| Lupus_b | 1.167 | 0.256 | 0.70 | 0.483 | 0.758 | 1.794 |
| Erectile_dysfunction_b | 1.006 | 0.039 | 0.16 | 0.870 | 0.932 | 1.086 |
| Any_tumour_b | 1.038 | 0.035 | 1.09 | 0.275 | 0.971 | 1.109 |
| Menopause_b | 0.809 | 0.040 | -4.25 | 0.000 | 0.734 | 0.892 |
| Dementia_b | 2.043 | 0.115 | 12.71 | 0.000 | 1.830 | 2.281 |
| RA_b | 1.388 | 0.088 | 5.17 | 0.000 | 1.226 | 1.572 |
| num_ami_before_31_dec_14 | 1.000 | 0.001 | 0.04 | 0.968 | 0.997 | 1.003 |
| _cons | 0.009 | 0.001 | -29.68 | 0.000 | 0.006 | 0.012 |

**Performance in validation dataset (CPRD GOLD) - External validation - audit AMI cases**

| AUROC (95% CI): 0.713 (0.698; 0.727) |  |
| --- | --- |
|  |  |
|  | % Correctly classified: 71.07 |

### 5) 5-year CV mortality

|  | | | | | | |
| --- | --- | --- | --- | --- | --- | --- |
| _5year_CV_death | Odds ratio | Std. err. | z | P>|z| | [95% conf. interval] | |
| age | 1.088 | 0.002 | 43.38 | 0.000 | 1.084 | 1.092 |
|  |  |  |  |  |  |  |
| gender |  |  |  |  |  |  |
| Female | 0.934 | 0.036 | -1.78 | 0.076 | 0.866 | 1.007 |
|  |  |  |  |  |  |  |
| IMD |  |  |  |  |  |  |
| Q2 | 1.250 | 0.062 | 4.49 | 0.000 | 1.134 | 1.378 |
| Q3 | 1.328 | 0.066 | 5.67 | 0.000 | 1.204 | 1.465 |
| Q4 | 1.240 | 0.063 | 4.23 | 0.000 | 1.123 | 1.371 |
| Q5-most deprived | 1.471 | 0.075 | 7.52 | 0.000 | 1.330 | 1.627 |
|  |  |  |  |  |  |  |
| ethnicity2 | 0.847 | 0.057 | -2.48 | 0.013 | 0.742 | 0.966 |
| Alcohol_status3 | 0.900 | 0.038 | -2.53 | 0.011 | 0.829 | 0.976 |
|  |  |  |  |  |  |  |
| Smoking_status |  |  |  |  |  |  |
| current smoker | 1.670 | 0.083 | 10.35 | 0.000 | 1.515 | 1.840 |
| ex-smoker | 1.162 | 0.051 | 3.43 | 0.001 | 1.067 | 1.267 |
|  |  |  |  |  |  |  |
| BMI_2014_f | 0.991 | 0.003 | -2.79 | 0.005 | 0.984 | 0.997 |
| HT_b | 1.087 | 0.035 | 2.58 | 0.010 | 1.020 | 1.159 |
| Hyperlipidaemia_b | 1.083 | 0.036 | 2.36 | 0.018 | 1.014 | 1.156 |
|  |  |  |  |  |  |  |
| Diabetes_type_au_b |  |  |  |  |  |  |
| T1DM | 6.936 | 1.133 | 11.85 | 0.000 | 5.035 | 9.555 |
| T2DM | 1.963 | 0.073 | 18.20 | 0.000 | 1.825 | 2.110 |
| DM - nos | 3.520 | 0.381 | 11.62 | 0.000 | 2.847 | 4.353 |
|  |  |  |  |  |  |  |
| HF_b | 2.616 | 0.094 | 26.90 | 0.000 | 2.439 | 2.806 |
| AF_b | 1.490 | 0.055 | 10.83 | 0.000 | 1.386 | 1.601 |
| Heart_valve_dis_b | 1.822 | 0.188 | 5.83 | 0.000 | 1.489 | 2.229 |
| VT_VF_b | 1.487 | 0.174 | 3.39 | 0.001 | 1.182 | 1.870 |
| Cardiomyopathy_b | 2.595 | 0.284 | 8.72 | 0.000 | 2.094 | 3.215 |
| CV_procedures_b | 0.834 | 0.027 | -5.52 | 0.000 | 0.782 | 0.890 |
| TIA_stroke_b | 1.674 | 0.065 | 13.33 | 0.000 | 1.552 | 1.806 |
| PVD_b | 1.772 | 0.086 | 11.83 | 0.000 | 1.612 | 1.949 |
| CKD_b | 1.501 | 0.050 | 12.19 | 0.000 | 1.406 | 1.602 |
| Hypothyroidism_b | 1.005 | 0.048 | 0.10 | 0.920 | 0.914 | 1.104 |
| Liver_dis_b | 1.374 | 0.452 | 0.96 | 0.335 | 0.721 | 2.618 |
| Lupus_b | 1.337 | 0.358 | 1.08 | 0.279 | 0.790 | 2.261 |
| Erectile_dysfunction_b | 0.891 | 0.043 | -2.41 | 0.016 | 0.811 | 0.979 |
| Any_tumour_b | 0.978 | 0.036 | -0.61 | 0.545 | 0.909 | 1.051 |
| Menopause_b | 0.698 | 0.042 | -6.00 | 0.000 | 0.620 | 0.785 |
| Dementia_b | 3.137 | 0.180 | 19.94 | 0.000 | 2.803 | 3.510 |
| RA_b | 1.176 | 0.090 | 2.12 | 0.034 | 1.012 | 1.367 |
| num_ami_before_31_dec_14 | 0.992 | 0.002 | -4.11 | 0.000 | 0.988 | 0.996 |
| _cons | 0.000 | 0.000 | -44.00 | 0.000 | 0.000 | 0.000 |

**Performance in validation dataset (CPRD GOLD) - External validation - audit AMI cases**

| AUROC (95% CI): 0.854 (0.843; 0.866) |  |
| --- | --- |
|  |  |
|  | % Correctly classified: 81.88 |

### 6) 5-year Composite 1 outcome (HF, stroke, recurrent MI, & all-cause mortality)

|  | | | | | | |
| --- | --- | --- | --- | --- | --- | --- |
| _5yr_Comp1_outcome | Odds ratio | Std. err. | z | P>|z| | [95% conf. interval] | |
| age | 1.063 | 0.001 | 53.33 | 0.000 | 1.061 | 1.066 |
|  |  |  |  |  |  |  |
| gender |  |  |  |  |  |  |
| Female | 1.047 | 0.030 | 1.62 | 0.106 | 0.990 | 1.108 |
|  |  |  |  |  |  |  |
| IMD |  |  |  |  |  |  |
| Q2 | 1.191 | 0.039 | 5.30 | 0.000 | 1.117 | 1.271 |
| Q3 | 1.268 | 0.043 | 7.07 | 0.000 | 1.187 | 1.354 |
| Q4 | 1.306 | 0.044 | 7.89 | 0.000 | 1.222 | 1.395 |
| Q5-most deprived | 1.457 | 0.050 | 10.94 | 0.000 | 1.362 | 1.559 |
|  |  |  |  |  |  |  |
| ethnicity2 | 1.079 | 0.045 | 1.81 | 0.070 | 0.994 | 1.171 |
| Alcohol_status3 | 0.869 | 0.027 | -4.55 | 0.000 | 0.818 | 0.923 |
|  |  |  |  |  |  |  |
| Smoking_status |  |  |  |  |  |  |
| current smoker | 1.578 | 0.052 | 13.78 | 0.000 | 1.479 | 1.684 |
| ex-smoker | 1.137 | 0.034 | 4.24 | 0.000 | 1.071 | 1.206 |
|  |  |  |  |  |  |  |
| BMI_2014_f | 1.000 | 0.002 | -0.18 | 0.858 | 0.995 | 1.004 |
| HT_b | 1.186 | 0.026 | 7.70 | 0.000 | 1.136 | 1.239 |
| Hyperlipidaemia_b | 1.025 | 0.024 | 1.06 | 0.291 | 0.979 | 1.074 |
|  |  |  |  |  |  |  |
| Diabetes_type_au_b |  |  |  |  |  |  |
| T1DM | 4.558 | 0.618 | 11.19 | 0.000 | 3.494 | 5.946 |
| T2DM | 1.549 | 0.043 | 15.87 | 0.000 | 1.467 | 1.635 |
| DM - nos | 2.295 | 0.231 | 8.25 | 0.000 | 1.884 | 2.795 |
|  |  |  |  |  |  |  |
| HF_b | 5.108 | 0.188 | 44.32 | 0.000 | 4.753 | 5.490 |
| AF_b | 1.894 | 0.064 | 18.90 | 0.000 | 1.773 | 2.024 |
| Heart_valve_dis_b | 1.501 | 0.161 | 3.79 | 0.000 | 1.216 | 1.852 |
| VT_VF_b | 1.588 | 0.151 | 4.86 | 0.000 | 1.318 | 1.914 |
| Cardiomyopathy_b | 1.884 | 0.213 | 5.61 | 0.000 | 1.510 | 2.351 |
| CV_procedures_b | 0.905 | 0.019 | -4.64 | 0.000 | 0.868 | 0.944 |
| TIA_stroke_b | 3.269 | 0.124 | 31.12 | 0.000 | 3.034 | 3.522 |
| PVD_b | 1.730 | 0.076 | 12.44 | 0.000 | 1.587 | 1.886 |
| CKD_b | 1.476 | 0.040 | 14.48 | 0.000 | 1.400 | 1.556 |
| Hypothyroidism_b | 1.064 | 0.041 | 1.63 | 0.104 | 0.987 | 1.147 |
| Liver_dis_b | 1.568 | 0.321 | 2.20 | 0.028 | 1.050 | 2.342 |
| Lupus_b | 1.575 | 0.284 | 2.52 | 0.012 | 1.105 | 2.244 |
| Erectile_dysfunction_b | 1.002 | 0.030 | 0.06 | 0.955 | 0.944 | 1.063 |
| Any_tumour_b | 1.368 | 0.039 | 10.97 | 0.000 | 1.293 | 1.446 |
| Menopause_b | 0.850 | 0.034 | -4.11 | 0.000 | 0.787 | 0.919 |
| Dementia_b | 4.240 | 0.365 | 16.79 | 0.000 | 3.582 | 5.018 |
| RA_b | 1.325 | 0.074 | 5.06 | 0.000 | 1.188 | 1.478 |
| num_ami_before_31_dec_14 | 0.998 | 0.001 | -2.24 | 0.025 | 0.996 | 1.000 |
| _cons | 0.004 | 0.001 | -44.22 | 0.000 | 0.003 | 0.005 |

**Performance in validation dataset (CPRD GOLD) - External validation - audit AMI cases**

| AUROC (95% CI): 0.775 (0.766; 0.785) |  |
| --- | --- |
|  |  |
|  | % Correctly classified: 70.38 |

### 7) 5-year Composite 1 CV outcome (HF, stroke, recurrent MI, & CV mortality)

|  | | | | | | |
| --- | --- | --- | --- | --- | --- | --- |
| _5yr_Comp1_CV_outcome | Odds ratio | Std. err. | z | P>|z| | [95% conf. interval] | |
| age | 1.056 | 0.001 | 47.71 | 0.000 | 1.054 | 1.059 |
|  |  |  |  |  |  |  |
| gender |  |  |  |  |  |  |
| Female | 1.056 | 0.030 | 1.91 | 0.056 | 0.999 | 1.117 |
|  |  |  |  |  |  |  |
| IMD |  |  |  |  |  |  |
| Q2 | 1.108 | 0.037 | 3.09 | 0.002 | 1.038 | 1.183 |
| Q3 | 1.226 | 0.041 | 6.04 | 0.000 | 1.147 | 1.309 |
| Q4 | 1.254 | 0.043 | 6.67 | 0.000 | 1.173 | 1.340 |
| Q5-most deprived | 1.405 | 0.048 | 9.87 | 0.000 | 1.314 | 1.504 |
|  |  |  |  |  |  |  |
| ethnicity2 | 1.174 | 0.049 | 3.83 | 0.000 | 1.081 | 1.274 |
| Alcohol_status3 | 0.892 | 0.027 | -3.75 | 0.000 | 0.840 | 0.947 |
|  |  |  |  |  |  |  |
| Smoking_status |  |  |  |  |  |  |
| current smoker | 1.513 | 0.050 | 12.49 | 0.000 | 1.418 | 1.615 |
| ex-smoker | 1.119 | 0.034 | 3.73 | 0.000 | 1.055 | 1.188 |
|  |  |  |  |  |  |  |
| BMI_2014_f | 1.007 | 0.002 | 3.03 | 0.002 | 1.002 | 1.011 |
| HT_b | 1.221 | 0.027 | 9.01 | 0.000 | 1.169 | 1.275 |
| Hyperlipidaemia_b | 1.040 | 0.024 | 1.67 | 0.096 | 0.993 | 1.089 |
|  |  |  |  |  |  |  |
| Diabetes_type_au_b |  |  |  |  |  |  |
| T1DM | 3.849 | 0.512 | 10.14 | 0.000 | 2.966 | 4.995 |
| T2DM | 1.559 | 0.043 | 16.24 | 0.000 | 1.478 | 1.645 |
| DM - nos | 2.299 | 0.227 | 8.43 | 0.000 | 1.894 | 2.790 |
|  |  |  |  |  |  |  |
| HF_b | 5.934 | 0.213 | 49.50 | 0.000 | 5.530 | 6.368 |
| AF_b | 1.937 | 0.064 | 20.15 | 0.000 | 1.816 | 2.066 |
| Heart_valve_dis_b | 1.552 | 0.162 | 4.21 | 0.000 | 1.265 | 1.904 |
| VT_VF_b | 1.487 | 0.140 | 4.20 | 0.000 | 1.236 | 1.790 |
| Cardiomyopathy_b | 2.084 | 0.234 | 6.53 | 0.000 | 1.672 | 2.597 |
| CV_procedures_b | 0.949 | 0.021 | -2.40 | 0.016 | 0.910 | 0.991 |
| TIA_stroke_b | 3.596 | 0.132 | 34.83 | 0.000 | 3.346 | 3.864 |
| PVD_b | 1.796 | 0.077 | 13.60 | 0.000 | 1.651 | 1.955 |
| CKD_b | 1.466 | 0.039 | 14.49 | 0.000 | 1.392 | 1.544 |
| Hypothyroidism_b | 1.087 | 0.041 | 2.21 | 0.027 | 1.009 | 1.170 |
| Liver_dis_b | 1.287 | 0.267 | 1.21 | 0.225 | 0.857 | 1.933 |
| Lupus_b | 1.592 | 0.286 | 2.58 | 0.010 | 1.119 | 2.265 |
| Erectile_dysfunction_b | 0.994 | 0.030 | -0.20 | 0.843 | 0.937 | 1.055 |
| Any_tumour_b | 1.158 | 0.033 | 5.20 | 0.000 | 1.096 | 1.224 |
| Menopause_b | 0.855 | 0.034 | -3.98 | 0.000 | 0.791 | 0.923 |
| Dementia_b | 3.128 | 0.226 | 15.78 | 0.000 | 2.714 | 3.604 |
| RA_b | 1.267 | 0.070 | 4.28 | 0.000 | 1.137 | 1.412 |
| num_ami_before_31_dec_14 | 0.998 | 0.001 | -1.83 | 0.067 | 0.996 | 1.000 |
| _cons | 0.004 | 0.001 | -43.61 | 0.000 | 0.003 | 0.005 |

**Performance in validation dataset (CPRD GOLD) - External validation - audit AMI cases**

| AUROC (95% CI): 0.759 (0.748; 0.771) |  |
| --- | --- |
|  |  |
|  | % Correctly classified: 69.84 |

### 8) 5-year Composite 2 outcome (HF, stroke, & all-cause mortality)

|  | | | | | | |
| --- | --- | --- | --- | --- | --- | --- |
| _5yr_Comp2_outcome | Odds ratio | Std. err. | z | P>|z| | [95% conf. interval] | |
| age | 1.072 | 0.001 | 57.40 | 0.000 | 1.069 | 1.074 |
|  |  |  |  |  |  |  |
| gender |  |  |  |  |  |  |
| Female | 1.065 | 0.031 | 2.16 | 0.031 | 1.006 | 1.128 |
|  |  |  |  |  |  |  |
| IMD |  |  |  |  |  |  |
| Q2 | 1.206 | 0.041 | 5.48 | 0.000 | 1.128 | 1.289 |
| Q3 | 1.279 | 0.044 | 7.10 | 0.000 | 1.195 | 1.369 |
| Q4 | 1.329 | 0.046 | 8.16 | 0.000 | 1.242 | 1.424 |
| Q5-most deprived | 1.512 | 0.054 | 11.64 | 0.000 | 1.410 | 1.621 |
|  |  |  |  |  |  |  |
| ethnicity2 | 1.050 | 0.046 | 1.11 | 0.265 | 0.964 | 1.143 |
[truncated: 65,542 more chars]
