# Supplementary material for: Risk prediction in people with acute myocardial infarction in England: a cohort study using data from 1521 general practices
Source: BMJ Open. 2025 Dec 5;15(12):e094961. doi: 10.1136/bmjopen-2024-094961 (PMC12684221; doi:10.1136/bmjopen-2024-094961)
Supplement: Supplementary file 3 [file bmjopen-2024-094961.reviewer_comments.pdf]

PEER REVIEW HISTORY

BMJ Open publishes all reviews undertaken for accepted manuscripts. Reviewers are asked to complete a checklist review form and are provided with free text boxes to elaborate on their assessment. These free text comments are reproduced below.

ARTICLE DETAILS

Title (Provisional)

Risk prediction in people with acute myocardial infarction in England: a cohort study using data from 1521 general practices

Authors

Kontopantelis, Evangelos; Arsene, Corneliu T; Chew, Nicholas WS; Zghebi, Salwa S; Zaman, Azfar; Wijeysundera, Harindra; Khunti, Kamlesh; Ashcroft, Darren; Carr, M; Parisi, Rosa; Mamas, Mamas

---

VERSION 1 - REVIEW

**Reviewer**	**1**
**Name**	**Chapman, Andrew**
**Affiliation**	**Royal Infirmary of Edinburgh, British Heart Foundation Center for Cardiovascular Science**
**Date**	**09-Jun-2025**
**COI**	**None**

Kontopantelis et al have applied logistic regression modelling to identify predictors of short term outcomes (1 year / 5 year) following a first presentation with myocardial infarction event (referred to as index) or in those who have had a previous MI (referred to as prevalent) using routine clinical care data.

The authors highlight the perceived gap in the evidence base for risk stratification in patients who survive acute myocardial infarction, and their rationale is clear. The authors should be commended for providing such a comprehensive, transparent data supplement.

I support publication but have some suggestions to improve clarity of reporting

1. I am not sure the nomenclature for index and prevalent models is optimal; this is an editorial decision but I wonder if models entitled 'index event' and 'all events' would be easier?

2. Did the authors consider comparing the performance of their new model against an established risk prediction tool for post MI CV risk (e.g. the GRACE score) as this would provide important context?

3. Did the authors consider (or have access to) additional predictive co-variates related to index infarct such as peak cardiac troponin concentration, BNP, LV function, coronary disease severity? commenting on this as a limitation would be helpful

4. The model is very good at predicting all cause mortality, one aspect of this will be frailty. They could consider adding the electronic frailty score or acknowledging frailty will influence risk prediction (and its effectiveness).

5. It would be helpful to state that the model applies only to patients who have experienced a type 1 myocardial infarction (due to atherosclerotic plaque rupture) rather than other subtypes, where competing risk of non-CV events is more likely to attenuate the observed CV event rate.

Minor comments

1. Please define the primary composite outcome in your abstract.

2. Please provide the total number of patients within the introductory results paragraph and provide denominators for proportions

3. Figure 1 and 2 is entitled 'Calibration performance summary' and reports AUC (area under the curve) which is a measure of discrimination. I would anticipate calibration to be determined by a plot of observed versus predicted, or a statistical test (noting the limitations of the HL test). Please clarify and revise.

4. It would be helpful to have Figure numbers in the data supplement for AUC and calibration plots to help direct the reader.

5. Page 7, Line 25; 'considerably' should be 'considerable'

**Reviewer**	**2**
**Name**	**Sayehmiri, kourosh**
**Affiliation**	**Ilam University of Medical Scienses**
**Date**	**04-Sep-2025**
**COI**	**None**

1- in intruduction authors did not justify gap of knowlege for this research.

2- In cohorst studies we have event time and outcome, approprate statistical analysis is survival models such as Cox PH, and parametric survival models, logistic regresion ignor event time and it is not approprate for this data.

3- Authors did not comprire effeicacy logistic regresion with othre models such as Random Forest (RF) , Decision Tree (DT) , k-Nearest Neighbors (KNN).

4- authors must compute effect of covariatee on AUC

5- Authors did not a practical models that clinician use it in decition making

• Support Vector Machine (SVM),

Reviewer **3**

Name **Kurth, Tobias**

Affiliation **Charité Universitätsmedizin Berlin, Institute of Public Health**

Date **03-Oct-2025**

COI **TK reports having received research grants from the German Federal Joint Committee (G-BA) and personal compensation from the North-East German Society for Gynecological Oncology (NOGGO), AbbVie, Eli Lilly & Company, Novartis, the BMJ Group, and Frontiers Media S.A.**

In this cohort study, using data from the Clinical Practice Research Datalink (CPRD Aurum database (N=1,018 general practices), internally validated in the remaining 20% (N=255) and externally validated in the CPRD GOLD database (N=248)), Kontopantelis and colleagues aimed to develop prediction models for short-term (1- and 5-year) outcomes (all-cause mortality, composite outcomes, and cause-specific mortality) immediately following a first myocardial infarction. Discrimination was evaluated using sensitivity, specificity and the area under the curve (AUC). Calibration was evaluated using calibration plots.

The findings of the study showed good or very good discrimination for the developed risk prediction models following incident myocardial infarction, but also for prevalent myocardial infarction, focusing on 1-year and 5-year mortality and composite CVD outcomes.

This study may provide useful information for clinicians treating patients with their first myocardial infarction and classifying them at risk of subsequent events. However, several aspects of the paper limit the interpretation of the data.

1. While the modelling approach is described in terms of covariates, performance measures, and validation, the manuscript does not outline the overall strategy for model development. It remains unclear whether predictors were fully pre-specified and how, whether any variable selection, shrinkage, or penalisation methods were considered, and how the final specification was determined. Without this information, it is impossible to evaluate the reported models and the risk of overfitting. I would encourage the authors to clarify their modelling strategy (e.g., pre-specification vs. data-driven selection, handling of nonlinearity,

and potential interactions) and to explain the rationale for arriving at the final models. This is also part of the TRIPOD guideline, which the authors indicate they have followed.

2. The authors conclude that their study supports closer monitoring and optimal secondary prevention strategies for people with their first myocardial infarction and that patients can be informed of the likelihood of future adverse outcomes and advised on modifiable risk factors. This, however, can only be concluded after impact studies of the prediction models.

3. The authors state that they also report on factors associated with poor outcomes. The aim of this part of the study, however, is not clear. Are the authors speaking of causal risk factors? As the authors aimed to develop prediction models, it is unclear what this information should add. In addition, I could not find a section where such factors are presented.

4. The authors state that they report accuracy, sensitivity, specificity, positive predictive value, negative predictive value, true positives, true negatives, false positives, and false negatives. However, these words can only be found in the methods section of the manuscript.

5. It is unfortunate that the authors opted not to use time-to-event modelling. I do not agree with their rationale that, since they focused on prediction rather than causal inference, the risk of bias is less of a concern. Also, prediction models should be as precise as possible.

6. It is unfortunate that the authors did not pre-register their study. This may have prevented showing endless tables and figures in the supplements. It remains unclear for whom such data overload would be useful.

7. Based on the provided contribution section, some authors do not fulfil the ICMJE criteria for authorship.

VERSION 1 - AUTHOR RESPONSE

Reviewer: 1
Dr. Andrew Chapman, Royal Infirmary of Edinburgh
Comments to the Authors:

Kontopantelis et al have applied logistic regression modelling to identify predictors of short term outcomes (1 year / 5 year) following a first presentation with myocardial infarction event (referred to as index) or in those who have had a previous MI (referred to as prevalent) using routine clinical care data.

The authors highlight the perceived gap in the evidence base for risk stratification in patients who survive acute myocardial infarction, and their rationale is clear. The authors should be commended for providing such a comprehensive, transparent data supplement.

I support publication but have some suggestions to improve clarity of reporting
Response:
Thank you for your supportive evaluation.

1. I am not sure the nomenclature for index and prevalent models is optimal; this is an editorial decision but I wonder if models entitled 'index event' and 'all events' would be easier?
Response:
We agree that this is debatable, and we struggled with this ourselves, originally calling these audit and index models. However, we are sure the reviewer acknowledges the subjective nature of this, and our preference would be a single word to label these models. Nevertheless, we are happy to follow the editor's guidance in this.

2. Did the authors consider comparing the performance of their new model against an established risk prediction tool for post MI CV risk (e.g. the GRACE score) as this would provide important context?
Response:
We appreciate the suggestion. Our aim was to develop a risk prediction tool specifically for use in primary care, using only data routinely available in that setting (excluding outcomes, which were obtained via linkage). The GRACE score, while well-established, relies on secondary care variables such as troponin levels, ECG findings, and Killip class, which are not typically recorded in primary care electronic health records. For this reason, we did not consider GRACE as a comparator. Secondly, the GRACE model is validated for use in the acute AMI setting, particularly NSTEMI, whereas our models were developed for use irrespective of acuity or type of AMI. We have now made this clearer in the limitations section as well:
"Many more covariates would be relevant to accurate risk prediction (e.g. troponin levels, ECG findings) but we aimed to develop a risk prediction model for use in primary care, where such information is not routinely available."

3. Did the authors consider (or have access to) additional predictive co-variates related to index infarct such as peak cardiac troponin concentration, BNP, LV function, coronary disease severity? commenting on this as a limitation would be helpful
Response:
Please see our response to the previous point (GRACE score). These covariates would definitely improve performance, but are not routinely available in primary care data.

4. The model is very good at predicting all-cause mortality, one aspect of this will be frailty. They could consider adding the electronic frailty score or acknowledging frailty will influence risk prediction (and its effectiveness).

Response:

Thank you for raising this. We chose not to include aggregate measures of multimorbidity or frailty, such as the electronic Frailty Index (eFI) or the Cambridge Multimorbidity Score, because our aim was to capture the individual predictive contributions of a wide range of conditions. Including both aggregate scores and their component conditions as binary covariates would introduce redundancy and potential multicollinearity. We therefore opted against dimensionality reduction, as we anticipated it could compromise model performance. We acknowledge that frailty is an important predictor of all-cause mortality, and we would expect it be indirectly captured in the list of included comorbidities, and we now discuss its relevance in the limitations section of the manuscript:
"Multimorbidity and frailty will be driving 1- and 5-year mortality, however, we opted not to use composite scores. Instead, we included a wide range of individual comorbidities to better capture distinct predictive contributions. Including both aggregate scores and their component conditions would introduce redundancy, and we anticipated that dimensionality reduction could compromise model performance."

5. It would be helpful to state that the model applies only to patients who have experienced a type 1 myocardial infarction (due to atherosclerotic plaque rupture) rather than other subtypes, where competing risk of non-CV events is more likely to attenuate the observed CV event rate.

Response:

We thank the reviewer for this important observation. Our study aimed to model risk following acute myocardial infarction recorded in primary care, but we did not explicitly differentiate between MI subtypes (e.g. Type 1 vs Type 2). The main reason being that these tools are aimed for primary care use and that information is not routinely available in primary care (not without linkage to Hospital Episode Statistics data). However, given that Type 1 MI is the predominant form captured in secondary care, we expect the majority of cases to be Type 1. We agree that competing risks are more prominent in other MI subtypes, and will now clarify this in the limitations section:
"We did not differentiate between AMI subtypes (e.g. Type 1 due to atherosclerotic plaque rupture vs Type 2 due to mismatch between myocardial oxygen supply and demand), as this information is not routinely available in primary care records. However, given that Type 1 is the predominant form captured in secondary care, we expect most cases to reflect this subtype. We acknowledge that competing risks, particularly from non-cardiovascular causes, may be more prominent in other AMI subtypes and could influence risk prediction."

Minor comments

1. Please define the primary composite outcome in your abstract.

Response:

Done

2. Please provide the total number of patients within the introductory results paragraph and provide denominators for proportions

Response:

Thank you, we have added the numbers for the training and external validation cohorts and selected outcomes, with details presented in Tables 1 and S1. We are aware of the preference to report numerators and denominators as per the STROBE statement, and we have addressed that in the tables. We feel the paper is too busy, with numerous cohorts and outcomes to be presenting numerators and denominators in the text.

3. Figure 1 and 2 is entitled 'Calibration performance summary' and reports AUC (area under the curve) which is a measure of discrimination. I would anticipate calibration to be determined by a plot of observed versus predicted, or a statistical test (noting the limitations of the HL test). Please clarify and revise.

Response:

Apologies for this mistake, this has now been corrected.

4. It would be helpful to have Figure numbers in the data supplement for AUC and calibration plots to help direct the reader.

Response:

The reviewer does have a point, but previously we organised by section (including a table and a multigraph) for each outcome and model type combination, to make results more accessible and aligned across both appendices. We still think this is preferable and it avoids a TOC that would be exceedingly long and figure labels that are terribly long, trying to include all the different parameters.

5. Page 7, Line 25; 'considerably' should be 'considerable'

Response:

Thank you, corrected.

Reviewer: 2

Dr. Kourosh Sayehmiri, Ilam University of Medical Sciences

Comments to the Authors:

1- In Introduction authors did not justify gap of knowledge for this research.

Response:

Thank you. Please see the second paragraph of the introduction section:

"Specific risk stratification tools for AMI survivors are limited, despite an estimated 1.4 million survivors after a first AMI in the UK in 2023. Following a first AMI event, 1-year mortality risk is 5.6% for males and 7.2% for females, while 7-year risk is 13.9% for males and 16.2% for females.25 Small differences have been reported in the risk factors for first and second AMIs, with females being more at risk for second AMI. The risk of a second AMI is the highest during the first year, in those who survive a first AMI, and the risk of all-cause death in survivors remains high for at least 7 years of follow-up compared to the general population without a history of AMI. Although these studies identified important risk factors and quantified risks for people who have had an AMI, they stopped short of delivering risk stratification tools that could be used in the primary care setting, where CVD is managed in most countries. Furthermore, only one of the previous studies used primary care data, and examined important coronary risk factors such as smoking status, blood pressure and cholesterol levels."

We would be very happy to revisit this if a more specific recommendation is made by the reviewer.

2- In cohort studies we have event time and outcome, appropriate statistical analysis is survival models such as Cox PH, and parametric survival models, logistic regression ignore event time and it is not appropriate for this data.

Response:

The reviewer is correct, as is reviewer #3 who raises the same point, and this is something we have already acknowledged and provided a rationale for in the limitations section. There we say:
"Finally, we decided not to use time-to-event models, so that we would be able to provide clear prediction metrics for specific time points (1-year and 5-years), while employing simpler and less computationally demanding models. Since we are not focusing on inference but developing risk prediction models, following multiple imputation and external validation, risk of bias is less of a concern, although we concede that model performance may be somewhat affected."

3- Authors did not compare effeicacy logistic regression with other models such as Random Forest (RF) , Decision Tree (DT) , k-Nearest Neighbors (KNN).

Response:

This is a good point, and we plan to deliver this in future work and compare with logistic regression. Considering the size of the manuscript and the appendices, we did not feel it would have been prudent to include machine learning approaches. The reviewer is also very likely aware that all these algorithms deal with binary classification and are not easily generalisable to time-to-event data, so we would not be able to deliver both. We have now added, in the relevant section:
"In addition, this approach will allow us to directly compare with machine learning methods in future work,…"

4- authors must compute effect of covariates on AUC

Response:

Thank you, the strength of associations is reported for all models in the respective regression tables, so they can be inferred from those. Considering the volume of the tables and figures presented, for a very large number of models, we feel that would not add much to the readers.

5- Authors did not a practical models that clinician use it in decision making

Response:

Thank you, the first step to making the models acceptable to clinicians is publishing this work to demonstrate clinical value following peer review. It would be very difficult to pursue implementation without that. In addition, our approach (logistic regression) makes implementation much easier (compared to a time-to-event approach) and the models could sit externally to the EHRs. We have added this to the rationale for using the current approach:
"…, while making it much easier to implement the models in primary care."

Reviewer: 3

Dr. Tobias Kurth, Charité Universitätsmedizin Berlin
Comments to the Authors:

In this cohort study, using data from the Clinical Practice Research Datalink (CPRD Aurum database (N=1,018 general practices), internally validated in the remaining 20% (N=255) and externally validated in the CPRD GOLD database (N=248)), Kontopantelis and colleagues aimed to develop prediction models for short-term (1- and 5-year) outcomes (all-cause mortality, composite outcomes, and cause-specific mortality) immediately following a first myocardial infarction. Discrimination was evaluated using sensitivity, specificity and the area under the curve (AUC). Calibration was evaluated using calibration plots.

The findings of the study showed good or very good discrimination for the developed risk prediction models following incident myocardial infarction, but also for prevalent myocardial infarction, focusing on 1-year and 5-year mortality and composite CVD outcomes.

This study may provide useful information for clinicians treating patients with their first myocardial infarction and classifying them at risk of subsequent events. However, several aspects of the paper limit the interpretation of the data.

Response:

Thank you for the honest assessment of our work.

1. While the modelling approach is described in terms of covariates, performance measures, and validation, the manuscript does not outline the overall strategy for model development. It remains unclear whether predictors were fully pre-specified and how, whether any variable selection, shrinkage, or penalisation methods were considered, and how the final specification was determined. Without this information, it is impossible to evaluate the reported models and the risk of overfitting. I would encourage the authors to clarify their modelling strategy (e.g., pre-specification vs. data-driven selection, handling of nonlinearity, and potential interactions) and to explain the rationale for arriving at the final models. This is also part of the TRIPOD guideline, which the authors indicate they have followed.

Response:

Thank you for raising this point. All covariates were fully pre-specified based on clinical relevance, and the model development process was not data-driven at any stage – had we used such methods, we would have described them in detail. As noted in the "Outcomes and predictors" section, baseline covariates were selected a-priori by the clinical expert in our team (MAM). That said, we acknowledge that our modelling strategy could have been more explicitly described, particularly regarding shrinkage, penalisation, nonlinearity, and interactions. To clarify, none of these techniques were applied, and we have now added the following statement to the "Statistical modelling" section: "All model creation steps used are explicitly stated in this section; no additional procedures were applied." Note that overfitting is not really relevant to logistic regression models, as evidenced by similar performance levels across training, internal and external validation cohorts.

2. The authors conclude that their study supports closer monitoring and optimal secondary prevention strategies for people with their first myocardial infarction and that patients can be informed of the likelihood of future adverse outcomes and advised on modifiable risk factors. This, however, can only be concluded after impact studies of the prediction models.

Response:

Thank you, the reviewer has a point there. We mean that the performance of the models was such (adequate or better than that) that made monitoring a possibility, which in conjunction with more aggressive management could improve outcomes for these patients. The relevant statement, in the abstract, we think, is: "This can inform monitoring strategies and secondary prevention, and guide patient counselling on modifiable risk factors." We think this statement is valid, considering the abstract word limit. We do expand on this in the "Conclusions" section of the main paper:
"The proposed CV prediction tools, derived from primary care data, can provide actionable information to help primary care physicians to identify patients with existing CVD, who are at higher risk for future adverse events. Thus, they can prove useful in guiding more aggressive secondary care interventions and/or risk factor management, enabling primary care physicians to discuss the impact of CV risk-factor modification on future CV events, as well as identify patients that may benefit from more aggressive secondary management." Additionally, it would be difficult to pursue implementation or impact studies of the prediction models before publishing this peer-reviewed work first.

3. The authors state that they also report on factors associated with poor outcomes. The aim of this part of the study, however, is not clear. Are the authors speaking of causal risk factors? As the authors aimed to develop prediction models, it is unclear what this information should add. In addition, I could not find a section where such factors are presented.
Response:
The reviewer is right. Due to the size of the paper and the numerous models we do not explicitly discuss these factors, but we do report the full models in the two supplementary files, with all covariates and the strengths of associations presented there. We have now made this clearer when the supplementary files are mentioned ("Additional models").

4. The authors state that they report accuracy, sensitivity, specificity, positive predictive value, negative predictive value, true positives, true negatives, false positives, and false negatives. However, these words can only be found in the methods section of the manuscript.
Response:
Same as in the previous point, thank you for highlighting. We have now made this clearer that these performance measures are reported in the supplementary files.

5. It is unfortunate that the authors opted not to use time-to-event modelling. I do not agree with their rationale that, since they focused on prediction rather than causal inference, the risk of bias is less of a concern. Also, prediction models should be as precise as possible.
Response:
The reviewer has every right to be critical on this and we did consider the decision carefully. We do provide a justification which we have now expanded to include future work and implementation, as two additional reasons driving this decision. It was a difficult decision to make, but the number of models involved, and the computational complexity also played a role. Just to clarify as well that we did not discuss causal inference, or lack of, as a reason – what we did was acknowledge the methodological approach was suboptimal, but despite that the models performed well or very well in the main, as demonstrated using external validation. Finally, it is known that for short-term outcomes, logistic regression is equivalent to time-to-event models, assuming censorship levels are

not massive (e.g. see https://pubmed.ncbi.nlm.nih.gov/2616941/) – although we acknowledge that is less likely to be the case for 5-year outcomes.

6. It is unfortunate that the authors did not pre-register their study. This may have prevented showing endless tables and figures in the supplements. It remains unclear for whom such data overload would be useful.

Response:

Again, a valid point, and the reviewer is right we would not do things the same way, were we given the opportunity to start over. We do hope that the wide array of outcomes assessed will give academics a starting when considering future studies, and we wanted to be fully transparent about the models and outcomes we used – perhaps to a fault.

7. Based on the provided contribution section, some authors do not fulfil the ICMJE criteria for authorship.

Response:

Thank you for bringing this to our attention, the Contributorship statement has now been amended to address that.

VERSION 2 - REVIEW

Reviewer **1**

Name **Chapman, Andrew**

Affiliation **Royal Infirmary of Edinburgh, British Heart Foundation Center for Cardiovascular Science**

Date **28-Oct-2025**

COI

Thank you for the very detailed response to my feedback and addressing my points. This is a very comprehensive piece of work and I have no further suggestions.

Reviewer **3**

Name **Kurth, Tobias**

Affiliation **Charité Universitätsmedizin Berlin, Institute of Public Health**

Date **30-Oct-2025**

COI

In this revised version, Kontopantelis and colleagues have incorporated the reviewers' comments, and I would like to thank the authors for responding to my prior concerns in detail.

After reviewing this revised version, I have a few more comments for the authors to consider:

1. In the Strengths and Limitations of this Study section, the authors mention in the second-to-last point, confounding. However, confounding cannot play a role in prediction studies but only in causal inference studies.

2. As the authors study the prediction of acute myocardial infarction events and information on these predictors has been collected prior to the outcome for the index model part, at least this part of the study is prospective. I understand that using these databases is sometimes referred to as retrospective. To avoid confusion, I suggest dropping the term retrospective.

3. Thank you for your consideration in not using a time-to-event modelling approach. However, I remain concerned that the rationale provided does not fully address the implications of this choice. As highlighted in the PROBAST Explanation and Elaboration document (Moons et al., Ann Intern Med 2019), appropriate handling of censoring and follow-up time is essential to reduce bias in prediction model studies. While logistic regression may approximate time-to-event models when follow-up is short and censoring is minimal, this equivalence does not hold for longer time horizons, such as 5-year outcomes. Even when the primary aim of a study is prediction rather than causal inference, neglecting time-to-event information can lead to biased and less precise estimates of risk. I encourage the authors to acknowledge this limitation more clearly and to discuss how it might have affected their model performance and interpretation.

VERSION 2 - AUTHOR RESPONSE

Reviewer: 1
Dr. Andrew Chapman, Royal Infirmary of Edinburgh
Comments to the Author:
Thank you for the very detailed response to my feedback and addressing my points. This is a very comprehensive piece of work and I have no further suggestions.

Reviewer: 3
Dr. Tobias Kurth, Charité Universitätsmedizin Berlin

Comments to the Author:
In this revised version, Kontopantelis and colleagues have incorporated the reviewers' comments, and I would like to thank the authors for responding to my prior concerns in detail.

After reviewing this revised version, I have a few more comments for the authors to consider:

1. In the Strengths and Limitations of this Study section, the authors mention in the second-to-last point, confounding. However, confounding cannot play a role in prediction studies but only in causal inference studies.
Response:
Thank you, we agree and we have rephrased to “The use of routinely collected clinical data may introduce coding inaccuracies and bias / poor performance due to omitted predictors.”

2. As the authors study the prediction of acute myocardial infarction events and information on these predictors has been collected prior to the outcome for the index model part, at least this part of the study is prospective. I understand that using these databases is sometimes referred to as retrospective. To avoid confusion, I suggest dropping the term retrospective.
Response:
Agreed, done.

3. Thank you for your consideration in not using a time-to-event modelling approach. However, I remain concerned that the rationale provided does not fully address the implications of this choice. As highlighted in the PROBAST Explanation and Elaboration document (Moons et al., Ann Intern Med 2019), appropriate handling of censoring and follow-up time is essential to reduce bias in prediction model studies. While logistic regression may approximate time-to-event models when follow-up is short and censoring is minimal, this equivalence does not hold for longer time horizons, such as 5-year outcomes. Even when the primary aim of a study is prediction rather than causal inference, neglecting time-to-event information can lead to biased and less precise estimates of risk. I encourage the authors to acknowledge this limitation more clearly and to discuss how it might have affected their model performance and interpretation.
Response:
We appreciate the reviewer’s concern. We have expanded the relevant section to address that. The discussion around this was not trivial, previously:
“Finally, we decided not to use time-to-event models, so that we would be able to provide clear prediction metrics for specific time points (1-year and 5-years), while employing simpler and less computationally demanding models. Since we are not focusing on inference but developing risk prediction models, following multiple imputation and external validation, risk of bias is less of a concern, although we concede that model performance may be somewhat affected. In addition, this approach will allow us to directly compare with machine learning methods in future work, while making it much easier to implement the models in primary care.”
We have now edited this to explicitly mention bias:
“Finally, we decided not to use time-to-event models, so that we would be able to provide clear prediction metrics for specific time points (1-year and 5-years), while employing simpler and less computationally demanding models. However, while logistic regression offers simplicity and interpretability, it does not account for censoring or varying follow-up times, which may introduce

bias in longer-term predictions, leading to poor performance. On the other hand, the current approach will allow us to directly compare with machine learning methods in future work, while making it much easier to implement the models in primary care.”
